# A proteomic strategy to identify novel serum biomarkers for liver cirrhosis and hepatocellular cancer in individuals with fatty liver disease

**DOI:** 10.1186/1471-2407-9-271

**Published:** 2009-08-05

**Authors:** Joe Gray, Dipankar Chattopadhyay, Gary S Beale, Gillian L Patman, Luca Miele, Barry P King, Stephen Stewart, Mark Hudson, Christopher P Day, Derek M Manas, Helen L Reeves

**Affiliations:** 1 Pinnacle Proteomics Laboratory, The Medical School, Newcastle University, Newcastle-upon-Tyne, UK; 2 Northern Institute for Cancer Research, The Medical School, Newcastle University, Newcastle-upon-Tyne, UK; 3 Department of Internal Medicine, Policlinico Gemelli Hospital and Catholic University of the Sacred Heart, Rome, Italy; 4 The Liver Unit, Freeman Hospital, Newcastle-upon-Tyne, UK; 5 Institute of Cellular Medicine, The Medical School, Newcastle University, Newcastle-upon-Tyne, UK

## Abstract

**Background:**

Non-alcoholic fatty liver disease (NAFLD) has a prevalence of over 20% in Western societies. Affected individuals are at risk of developing both cirrhosis and hepatocellular cancer (HCC). Presently there is no cost effective population based means of identifying cirrhotic individuals and even if there were, our ability to perform HCC surveillance in the at risk group is inadequate. We have performed a pilot proteomic study to assess this as a strategy for serum biomarker detection.

**Methods:**

2D Gel electrophoresis was performed on immune depleted sera from 3 groups of patients, namely those with (1) pre-cirrhotic NAFLD (2) cirrhotic NAFLD and (3) cirrhotic NAFLD with co-existing HCC. Five spots differentiating at least one of these three groups were characterised by mass spectroscopy. An ELISA assay was optimised and a cross sectional study assessing one of these serum spots was performed on serum from 45 patients with steatohepatitis related cirrhosis and HCC and compared to 77 patients with histologically staged steatohepatitis.

**Results:**

Four of the spots identified were apolipoprotein isoforms, the pattern of which was able to differentiate the three groups. The 5^th ^spot, seen in the serum of cirrhotic individuals and more markedly in those with HCC, was identified as CD5 antigen like (CD5L). By ELISA assay, although CD5L was markedly elevated in a number of cirrhotic individuals with HCC, its overall ability to distinguish non-cancer from cancer individuals as determined by AUC ROC analysis was poor. However, serum CD5L was dramatically increased, independently of age, sex, and the presence of necroinflammation, in the serum of individuals with NAFLD cirrhosis relative to those with pre-cirrhotic disease.

**Conclusion:**

This novel proteomic strategy has identified a number of candidate biomarkers which may have benefit in the surveillance and diagnosis of individuals with chronic liver disease and/or HCC.

## Background

Globally, viral infections such as Hepatitis B (HBV) and Hepatitis C (HCV) are the principal causes of chronic liver injury, while in western nations steatohepatitis secondary to alcoholic liver disease (ALD) or non-alcoholic fatty liver disease (NAFLD) contribute significantly. NAFLD is the liver manifestation of the metabolic syndrome, characterised by central obesity, insulin resistance, hypertension and atherogenic dyslipidaemia. It is now the commonest cause of chronic liver disease in western countries.[1] Whatever the insult, chronic injury generates a persistent wound healing response associated with a changing extracellular matrix (ECM) and the accumulation of fibrous, type I collagen rich, scar tissue. Cirrhosis describes the end stages of this process and is characterised by the disruption of normal liver architecture by both fibrotic bands and disorganised nodules of regenerating hepatocytes. There is presently no medical treatment to reverse the changes of cirrhosis, although it is hoped that improved therapies (eg. antiviral therapy or those targeting the metabolic syndrome and/or NAFLD) introduced at lesser stages of disease may have an impact on their rate of progression to cirrhosis.

As well as deteriorating liver function and significant clinical morbidity, cirrhosis is associated with a markedly increased risk of developing hepatocellular carcinoma (HCC). With more than 500,000 cases diagnosed annually, HCC itself is a major health problem.[2] It is frequently detected at an advanced, incurable stage [3] and the survival of those affected has not altered significantly in the last two decades.[2,4,5] It was hoped that surveillance of cirrhotic individuals would facilitate early diagnosis of HCC and improve survival. Surveillance using liver imaging with abdominal ultrasound (USS) in combination with serum alpha fetoprotein (AFP) measurement is performed 6 monthly in many centres. This strategy, however, has limited value. There is no safe and cost-effective population-based means to identify the at risk cirrhotic population requiring surveillance. Liver biopsy is presently the best means of diagnosing cirrhosis, but it carries a significant risk and has well recognised limitations such as sampling error. It is only performed if there is a clinical indication. There are panels of serum-based tests,[6] some in conjunction with clinical parameters, [7,8]now proposed as useful in diagnosing cirrhosis. These too are largely aimed at individuals in the clinical setting, rather than being advocated as population-based screening tools. Despite the prevalence of NAFLD being 20-25% of the population,[9] the reality is that many of those who progress and develop cirrhosis remain unaware of their disease until a complication, such as an HCC, develops. Secondly, and even more disheartening for those with known cirrhosis, the diagnostic performance of AFP for HCC detection is inadequate [10] as it is only elevated in 40-60% of positive cases. Abdominal USS is little better as it is difficult in cirrhotic nodular livers and is notoriously user dependent.[11] USS in NAFLD patients is particularly difficult owing to the frequent association with central obesity.

Improved non-invasive means of detecting both cirrhosis and HCC are urgently required if we are to have an impact on the survival of the increasing numbers of individuals affected by these diseases. Although a number of alternative biomarkers for HCC have been proposed, largely in individuals with HCV and some in combination with AFP, none has yet had an impact on clinical practice. Here we report our own pilot study in individuals with either ALD or NAFLD. We have used immune depleted and filtered serum from individuals with liver disease, with and without cirrhosis, and from cirrhotic individuals with and without cancer. This serum has been studied using gel-based proteomic techniques in conjunction with mass spectroscopy to identify novel biomarkers distinguishing the three conditions. We have identified 5 candidate biomarkers capable of discriminating at least one or other of the patient groups. One novel serum protein, CD5L, was selected for further characterisation in a larger patient group. We have confirmed that it is an independent predictor of cirrhosis in NAFLD, and may also identify individuals at greater risk of HCC development.

## Methods

### Patient serum samples

All patient serum and clinical information was collected with patient consent after approval by The Newcastle and North Tyneside Ethics Committee. The liver histology of patients with NAFLD (steatosis on biopsy and compatible clinical features in the absence of an alcohol intake greater than 14 units weekly for women and 21 weekly for men) was staged according to the Brunt scoring system.[12] A fibrosis score of 4 describes cirrhosis. Serum samples from patients with chronic liver disease (including both ALD and NAFLD patients) were taken at the time of liver biopsy. Individuals with HCC were diagnosed as per guidelines proposed by the European Association for the Study of the Liver. [11] The majority of these were not biopsied, but each of them had an underlying clinical cirrhosis in association with a hypervascular lesion visible on two imaging modalities and compatible with the diagnosis of HCC. Serum samples from the individuals with HCC were pre-treatment samples taken at the time of diagnosis. Details of the HCC patients and controls with biopsy proven cirrhosis are included in Table 1. All serum samples were separated by centrifugation within 4 h and subsequently stored at -80°C. The standard biochemical serum tests, including serum AFP, were measured using routine automated methods in the Biochemistry Laboratory at the Freeman Hospital, Newcastle upon Tyne. No patient positive for either HBsAg or HCV were included in this study.

**Table 1:** Clinical characteristics of cirrhotic patients with and without HCC.

	Cirrhosis	HCC
**Number**	49	45
**Age (years)**	56.73 ± 8.9	67.8 ± 7.4
**Male:Female**	35:14	37:8
**ALD:NAFLD**	28:21	28:17
**Childs-Pugh A:B:C**	30:14:05	24:16:05
**AFP (ng/ml)**	6.04 ± 2.68	6551 ± 13602
**Bilirubin (μmol/L)**	33.42 ± 30.81	22.87 ± 22.29
**Albumin (g/l)**	36.36 ± 7.76	35.71 ± 5.02
**Portal vein invasion**	NA	8
**Single nodule**	NA	19
**Two nodules**	NA	8
**≥ 3 nodules**	NA	18

The difference in age between patients with cirrhosis and HCC versus those with cirrhosis alone was statistically significant (p < 0.001). There was no significant difference between sex or Child-Pugh stage between the two groups.

### Proteomic studies

Serum samples from 5 patients with NAFLD but no significant fibrosis, 5 patients with NAFLD and biopsy proven cirrhosis, and 5 patients with NAFLD cirrhosis and advanced AFP negative HCC were prepared for study. The samples were immunodepleted by multiple affinity removal system (MARS HPLC column, 4.6 × 100 mm; Agilent technologies, UK) and de-salted using 5 K molecular weight cut off spin filters (Agilent technologies, UK). 500 μg of total protein was separated per 2-dimensional gel electrophoresis run. The prepared protein was first diluted into a rehydration Buffer comprising 7 M urea, 2 M thiourea, 4% (w/v) CHAPS, 0.5% 3-10 IPG Buffer (GE Amersham) and 40 mM DTT and applied to an 18 cm pH 3-10 NL (GE Amersham) IPG strip. Gel rehydration isoelectric focussing was carried out using an IPGPhor IEF (GE Amersham) apparatus following the manufacturers stated running conditions with a final step and hold of 19 kVh. Subsequently, gel strips were equilibrated in a buffer comprising 50 mM Tris-HCl, pH 8.8, 6 M urea, 30% glycerol, 2% SDS, 0.002% bromophenol blue, containing 100 mg DTT per 10 ml for 15 min, followed by incubation in the same solution, but replacing DTT with 250 mg iodoacetamide per 10 ml, for an additional 15 min. Focussed IPG gel strips were then placed on top of a 25 × 25 cm, 1 mm thick 12% polyacrylamide gel and overlaid with 0.5% agarose. Resolution in the second dimension was performed using a DALTsix electrophoresis system (GE Amersham) running at an initial 5 W per gel for 30 mins, followed by 17 W per gel for approximately 5 hours (max 100 Wh). Gels were stained using colloidal Coomassie blue (ProtoBlue Safe Stain, National Diagnostics) following the manufacturers instructions.

All samples were analysed using duplicate gels. Gel images after scanning were stored as TIFF files and analysed using Progenesis Software (Nonlinear Dynamics). Protein spots of interest were excised, destained, and digested with trypsin. Peptide mass fingerprinting was performed using a Voyager DE-STR MALDI-TOF mass spectrometer (Applied Biosystems Inc.) operated in positive ion reflectron mode with α-cyano-4-hydroxycinnamic acid as the matrix. Protein identifications were performed using the Mascot Peptide Mass Fingerprint search program (Matrix Science Ltd).

### Serum ELISA assay

A direct ELISA for novel candidate CD5L was optimised using different concentrations of serum (raw, 1;10, 1:100, 1:500 and 1:1000), primary anti-CD5L antibody (R&D systems) and recombinant CD5L protein (R&D systems). Serum dilutions of either 1:10 or 1:100 generated a linear absorbance response over a protein range of 0.01 - 1.0 ng/ml using a primary antibody dilution of 1:500. This antibody dilution was then used to analyse all subsequent patient serum samples in triplicate at a dilution of 1:10.

### Liver Tissues CD5L mRNA analysis

Liver biopsy tissues surplus to diagnostic requirements were available from 21 patients with histologically staged pre-cirrhotic NAFLD, collected with appropriate ethical approvals in either Newcastle Hospitals or the Policlinico Gemelli Hospital, Rome. Histological staging was as defined by Brunt [12], although no RNA yield sufficient for analysis was obtained from a stage 4/cirrhotic biopsy. In addition, liver tissues obtained at the time of liver resection or radiofrequency ablation for benign or malignant primary or secondary tumours was obtained in the Newcastle Hospitals, with ethical approval, from an additional 14 individuals. One of these had histologically confirmed HCC and steatohepatitis related cirrhosis. The other 13 had 'normal livers', although mild steatosis or a mild focal mononuclear cell infiltrate were occasionally noted on histological assessment post resection. Semi-quantitative real time PCR analysis was performed as previously described [13] using GAPDH as a control gene and the following CD5L primers: Forward: 5' CAA CAA GCA TGC CTA TGG CCG AAA, Reverse: 5' TCA CAT TCG ACC CAC GTG TCT TCA. CD5L expression was expressed relative to the control gene GAPDH and a normal liver sample from an individual patient undergoing resection of a benign focal nodular hyperplasia.

### Statistical analysis

Quantitative variables are expressed as mean and standard deviation. Comparison between groups were performed by Pearson Chi-square, Wilcoxon or Student's t-test, as appropriate. The diagnostic accuracy of CD5L was evaluated using receiver operating characteristic (ROC) curve analysis, reporting the area under the curve (AUC) and its 95% confidence interval (CI). Statistical analyses were performed with SPSS version 14.

## Results

### Novel candidate biomarkers identified using proteomic techniques

**Figure 1:**
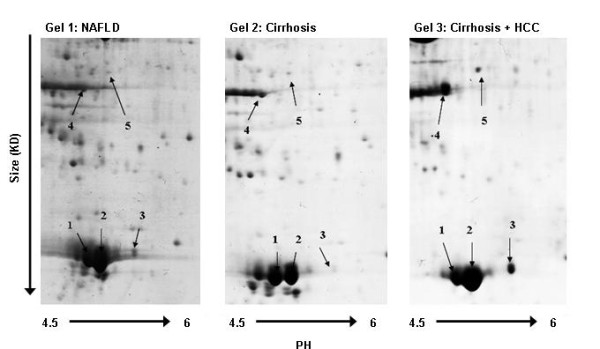
**Serum proteins separated by two dimensional gel electrophoresis in early and advanced NAFLD, with and without HCC**. These are representative blots comparing immune depleted and desalted serum from a patient with NAFLD and no fibrosis, a patient with NAFLD cirrhosis, and a patient with NAFLD cirrhosis and cancer. Serum profiles separated by 2D gel electrophoresis from 5 patients in each group, run in duplicate, were compared using Progenesis software. 5 spots were characterised by MS after excision. Spots 1,2 and 3 were isoforms of apolipoprotein A1, spot 4 was apolipoprotein A4, and spot 5 was identified as CD5 antigen like, or CD5L.

Figure 1 shows representative 2D gels of each of the three groups and identifies a number of spots, labelled 1-5. These spots, particularly in combination, enable the differentiation between the three patient groups, namely NAFLD without fibrosis, NAFLD with cirrhosis, and NAFLD with cirrhosis and HCC.

Protein identification of spots 1-5 was by Mascot search after mass spectroscopy. The identities and Mascot scores are summarised in Table 2. The individual mass spectra and protein summary information are available as additional files 1, 2, 3, 4, 5, 6, 7, 8, 9, 10, 11 in the supplementary information. Spots 1-3 were all identified after mass spectroscopy as apolipoprotein A1 (ApoA1). Spot 2 in particular was reduced in cirrhotic individuals relative to those with pre-cirrhotic NAFLD. In addition Pro-ApoA1, spot 3, was markedly present in all individuals with cirrhosis and HCC relative to only trace amounts in simple steatosis patients and cirrhotic patients without HCC. Protein spot 4 appeared lower in pre-cirrhotic NAFLD patients, compared to those with cirrhosis and in individuals with cirrhosis and HCC. This spot was identified as Apolipoprotein A4 (ApoA4). Protein spot 5 was identified as CD5 antigen like (CD5L). Relative to pre-cirrhotic NAFLD, this protein was identified in the serum of individuals with both cirrhosis and also those with cirrhosis and cancer, markedly so in the latter group.

**Table 2:** The identification of the differentiating protein spots on 2D gels.

Protein Spot	Protein Identified by Mascot Search	Accession Code (Entrez Protein)	Mascot Score (Expect value in brackets)*	Sequence Coverage %**
1	ApoA1 (human)	CAA00975	247 (6.5e^-19^)	80
2	ApoA1 (human)	CAA00975	236 (8.1e^-18^)	75
3	Pro-ApoA1 (human)***	CAA00975	195 (1.0e^-13^)	62
4	ApoA4 (human)	AAA51748	338 (5.1e^-28^)	64
**5**	CD5L/AIM (human)	AAD01446	218 (5.1e^-16^)	68

The protein spots are as numbered in Figure 1. The * Mascot score is shown as -10*Log(P), where P indicates the probability of a random match. Scores > 78 indicate that the protein identification is statistically significant (P < 0.05). ** Sequence coverage from matched peptides in spectra is provided in additional figures 1-10. *** Comparisons between zoomed mass spectra for spots 1 and 2 versus spot 3 are provided in additional figure 11. The pro-peptide sequence (RHFWQQDEPPQSPWDR...) generates an expected tryptic peptide at m/z = 2109.9 ([M+H]^+^). Mature ApoA1 sequence generates a predicted N-terminal tryptic peptide (DEPPQSPWDR...) of m/z = 1226.5 ([M+H]^+^). AIM = Apoptosis inhibitor expressed by Macrophages (alternate name for CD5L).

### CD5L determined by ELISA assay was very highly expressed in the serum of individuals with cirrhosis and HCC

**Figure 2:**
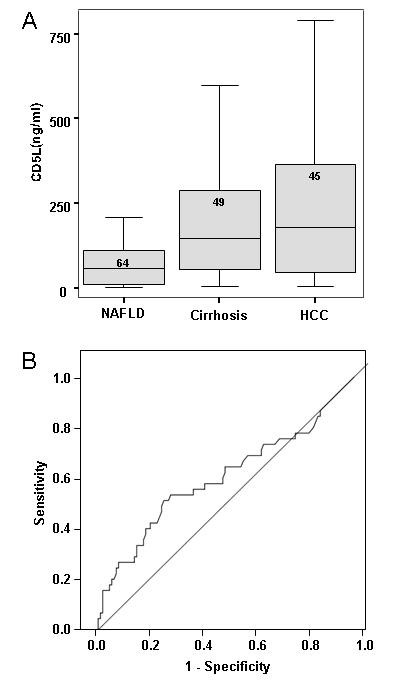
**CD5L discriminates poorly between cirrhotic patients with and without HCC**. Serum levels of CD5L were measured by ELISA assay in cirrhotic patients with (218 ± 221; n = 45) and without (194 ± 166; n = 49) HCC, as shown in 2A. Characteristics of the individuals are shown in Table 1. There was no significant difference between the two groups. ROC analyses is shown in 2B. The area under the curve is 0.495 (95% confidence intervals 0.376 and 0.614). A level > 400 ng/ml has a sensitivity of only 20%, but a specificity of 88%. Levels > 500 ng/ml have a specificity of 96%, > 600 ng/ml of 98% and > 700 ng/ml of 100%. For comparison, the mean CD5L serum level from patients without cirrhosis (detailed in figure 3) is also shown.

Serum CD5L was assessed in the serum of 45 individuals with steatohepatitis-related HCC and compared to levels in 49 individuals with biopsy proven steatohepatitis-related cirrhosis. Patient characteristics are shown in Table 1. The CD5L serum level was not significantly associated with age or sex. The box plots depicting the mean levels of the two groups of patients are shown in Figure 2 and are not significantly different (194 ± 167 without HCC versus 218 ± 221 with HCC). The AUC determined by ROC analysis (shown in Figure 2B) was 0.495 and indicated a poor serum CD5L discriminatory capacity between cirrhotic individuals with and without cancer. Some individuals with HCC did have, however, particularly high CD5L levels. A level greater than 400 ng/ml has a specificity for HCC of 88%. The sensitivity at this level was unacceptably poor (20%).

### CD5L ELISA in individuals with different stages of NAFLD was a good predictor of those with cirrhosis

**Figure 3:**
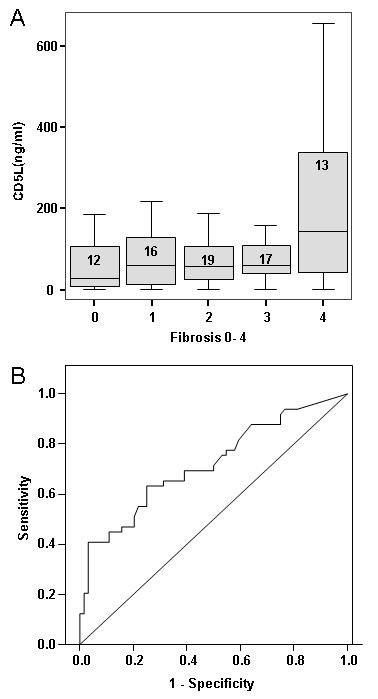
**CD5L discriminates between steatohepatitis patients with and without cirrhosis**. Serum levels of CD5L were measured in a total of 113 patients with either ALD or NAFLD. Fibrosis was scored histologically on liver biopsy as per the Brunt scoring system. The numbers of individuals in each group are shown within the boxes of chart 3A. The difference in CD5L levels between the different stages of fibrosis is statistically significant by univariate analysis (p = 0.004) controlled for both age and sex. This difference The ROC analyses for the identification of those individuals with stage 4 fibrosis (cirrhosis) is depicted in 3B. The area under the curve is 0.719 (95% confidence intervals 0.623 and 0.816), p < 0.0001. A level of CD5L 50 ng/ml has a sensitivity of 78% and a specificity of 46%, while a level of 100 ng/ml has a sensitivity of 63% and a specificity of 72%. A level greater than 200 ng/ml has a specificity of 95% (sensitivity 41%), and greater than 300 ng/ml of 97% (sensitivity 27%).

Serum from 77 patients, all of whom had biopsy proven NAFLD and had an alcohol intake of < 21 units per week for men and < 14 units for women, was studied. In this group of individuals the serum level of CD5L ng/ml in cirrhotic individuals (236 ± 223) was similar to those cirrhotic individuals with and without HCC described above. This level for stage 4 fibrosis/cirrhosis was significantly elevated compared to individuals with all other pre-cirrhotic stages (ANOVA p = 0.001, Bonferroni correction applied). Mean levels of CD5L were not significantly different between any other histologically defined group (steatosis or the presence or absence of necroinflammation). The level of CD5L was significantly associated with fibrosis independently of age, sex, steatosis or necroinflammation, as assessed by General Linear Model analysis (GLM, SPSS). The ROC analyses of CD5L as a predictor of stage 4 fibrosis in this independent group of patients with NAFLD is shown in Figure 3B. The area under the curve was 0.712 (95% CI 0.534-0.889).

### CD5L mRNA expression was elevated in individuals with NAFLD versus those with no underlying liver disease

**Figure 4:**
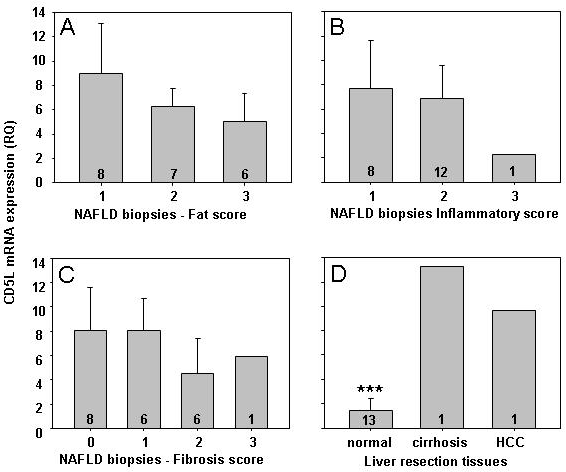
**CD5L mRNA expression is not altered in association with either fat, inflammation or fibrosis scores in pre-cirrhotic NAFLD liver tissues**. mRNA CD5L expression was quantified by real-time PCR, relative to GAPDH and a normal liver sample, in 21 pre-cirrhotic NAFLD biopsy tissues, 13 normal liver samples taken at the time of liver resection, one cirrhotic liver and one HCC. As shown in 4A-C, there was no difference in any pre-cirrhotic NAFLD biopsy tissues in association with the degree of fat, inflammation or fibrosis. There was a significant increase in the NAFLD tissues as a group (n = 21), compared with normal liver tissues (n = 13) as represented in 4D (6.945 ± 0.722 versus1.68 ± 0.269; p = 0.000, ***). The elevated CD5L mRNA expression in one cirrhotic and HCC tissue pair, obtained at the time of laparoscopic radiofrequency ablation, is also presented in 4C and is in keeping with the elevated serum CD5L levels identified in the larger cohort of patients studied.

CD5L mRNA expression was quantified in one set of liver biopsy tissues from patients with histologically staged NAFLD, and a set of normal liver tissues collected at the time of resection for benign or secondary cancers. (See methods) Data are expressed relative to the GAPDH control gene and a single normal liver sample from an individual undergoing resection for focal nodular hyperplasia. In the pre-cirrhotic NAFLD biopsy tissues, there was no significant difference in association with either fat, inflammatory or fibrosis score, as shown in Figure 4A-C. What was most notable, was that the liver mRNA expression of CD5L was significantly increased in the diseased NAFLD group as a whole (n = 21), relative to the normal liver group (n = 13) (6.945 ± 0.722 versus1.68 ± 0.269; p = 0.000). Although some of the 'normal' liver samples from patients undergoing resection for secondary malignancies had a 'mild steatosis' or a 'minimal focal mononuclear cell infiltrate' noted at the time of histological examination, there was no significant difference in CD5L expression in association with either of these minor changes (data not shown). The marked CD5L mRNA expression shown in the single cirrhotic tissue and HCC pair (Figure 4C) were in keeping with the serum ELISA assays from the much larger patient group.

## Discussion

The prevalence of chronic liver disease is increasing steadily in the UK, as is the population at risk of developing and dying from HCC. An increasing incidence of HCC has already been documented [5] and this is unfortunately compounded by the majority of tumours being diagnosed at a late stage when curative treatments are not possible. [14] Regular surveillance of high risk individuals is recommended but is presently hindered by the poor performance of the commonly used serum marker, AFP, [11] even in combination with abdominal USS. A significant effort has been and continues to be applied to the search for improved HCC biomarkers. As yet, none has proved superior to AFP in performance, but in combination some may have complimentary roles. [15,16] Our particular concern relates to the marked increase in the prevalence of ALD and NAFLD related HCC on our own unit. These individuals are often over 65 years of age and have cardiovascular co-morbidities precluding curative resection or transplantation. Much attention has been focused on validating quantitative fibrosis or cirrhosis markers in these individuals, with combinations of serum markers [8] showing encouraging if still suboptimal improvements in performance. Imaging methods such as the fibroscan may improve the efficiency of cirrhosis detection [17-19], but presently this technique needs to be interpreted with caution in obese individuals. [19-23] The need for further improvements in serum biomarkers for early detection of both advanced fibrosis and HCC grows ever more pressing.

We have applied a proteomic strategy using an optimised method of patient serum preparation in order to identify candidate biomarkers of either cirrhosis or HCC. This includes immune depletion prior to separation of serum by 2D gel electrophoresis. While this might remove some key biomarkers, we believe that this strategy improves the sensitivity of the technique applied to the remaining serum proteins. Having identified a number of spots able to discriminate between patients in at least one of our pre-defined disease groups, namely pre-cirrhotic NAFLD, cirrhotic NAFLD, and cirrhotic NAFLD with cancer, we selected candidates for identification by peptide mass spectroscopy.

The novel serum protein CD5L was identified in the serum of cirrhotic individuals with and without HCC by our proteomic strategy. CD5L, also known as Sp alpha, is a soluble protein belonging to group B of the scavenger receptor cysteine-rich (SRCR) superfamily for which little functional information is available.[24] It is expressed by macrophages present in lymphoid tissues (spleen, lymph node, thymus, and bone marrow), and it binds to myelomonocytic and lymphoid cells, suggesting that it may play an important role in the regulation of the innate and adaptive immune systems. It was recently identified in the sera of individuals with liver fibrosis related to HCV infection and, based on its proposed role in immune system regulation, was thought most likely associated with viral infection rather than cirrhosis.[25] While this may yet be true, as a mRNA transcript, it has been previously reported upregulated in HCC relative to dysplastic nodules.[26] We have investigated the potential of CD5L as a candidate biomarker for either advanced liver disease, or advanced liver disease and cancer. Our ELISA assay indicated a poor performance for CD5L as a surveillance tool for HCC, but again suggested value for cirrhosis detection. Our data clearly indicate, however, an association with cirrhosis in individuals without viral infection, which is independent of the presence or absence of histologically assessed inflammation. Our CD5L mRNA expression data from pre-cirrhotic NAFLD liver biopsies indicate an increase in association with fatty liver disease which, again, is not altered by the grade of either fat or inflammation. As CD5L does not increase incrementally with the level or stage of fibrosis, we propose that its dramatic increase in the serum of individuals with a background cirrhosis reflects hepatocyte regeneration, rather than the advanced fibrosis *per se*. The recent identification of CD5L by microarray as one of 30 mRNA transcripts expressed in patients with cirrhosis with a 'high risk' of HCC development [27] was one reason for our focus on this serum protein. Certainly some of our HCC patients had markedly high CD5L levels, while those with cirrhosis who had particularly high levels may be at high risk and may yet develop HCC. As a cirrhosis biomarker, the ROC analysis for CD5L indicates an accuracy of 72%. While this falls short of the accuracy of the European Liver Fibrosis (ELF) panel, recently validated in NAFLD patients and having an AUC of 0.9 for advanced fibrosis, CD5L is a single biomarker whose performance in conjunction with others may yet improve. In addition, it may have a value complementing that of AFP, which is predominantly elevated in patients with advanced HCC, in highlighting individuals with cirrhosis who are at greater risk of HCC development. At a level > 200 ng/ml, CD5L had a specificity for cirrhosis of 96% and a specificity for cancer of 60%.

Particularly prominent on our gel images were three spots identified as apolipoprotein A1 (ApoA1). ApoA1 is the major protein component of high density lipoprotein in plasma. It promotes cholesterol efflux from tissues to the liver for excretion and it is a cofactor for lecithin cholesterolacyltransferase which is responsible for the formation of most plasma cholesterol esters. It is not that surprising that this liver function associated protein alters in the serum of individuals with chronic liver disease and this has been previously reported in serum proteomic studies.[28] The mass of spots 1 and 2 (Figure 1) varied by approximately 30 daltons using MALDI analysis (Additional files 1 and 3) and are most likely attributable to post translational modification by oxidation. Notably, these two spots were reduced in cirrhotic individuals relative to those with pre-cirrhotic NAFLD, which is in keeping with previous reports and validates the clinical relevance of our methodology. In fact, a reduction of ApoA1 in the serum of patients with cirrhosis has remained a constitutive part of combined peptide panels used to predict fibrosis for a number of years. [6,29] In addition, however, we identified a novel isoform - spot 3. The difference in mass between spots 1 and 2 and spot 3 was much greater and in the order of 900 daltons (Additional files 1, 3 and 5). MALDI MS analysis of this spot has identified it as pro-Apolipoprotein A1 (Additional files 6 and 11), similarly detected and reported by an independent group of researchers studying patients with lung cancer. [30] Pro-Apolipoprotein A1 is proposed as a novel serum marker of brain metastases in lung cancer patients [30] and there may well be a rationale for its upregulation also in HCC patients. ApoA1 is secreted as the proprotein (pro-Apolipoprotein A1/spot 3) and is then cleaved, regulating its activation for lipid binding, by a metalloproteinase. One candidate metalloproteinase responsible is the c-terminal procollagen endoproteinase, Bone morphogenic protein 1 (BMP-1). [31] BMP-1 is secreted by the liver, but protease inhibitors, such as alpha-2-macroglobulin (A2M), are also secreted by the liver, often at elevated levels in inflammation or chronic disease.[32] Either a reduction in BMP-1, or an increase in inhibitors such as A2M - as reportedly occurs in HCC [33], could block the maturation of pro-Apolipoprotein A1, hence contributing to relative increase in this isoform (spot 3). In fact, the relative decrease in mature apoA1 in cirrhotic patients (spot 2) may also reflect increases in protease inhibition and A2M has similarly contributed significantly to serum diagnostic tests for fibrosis. [32,34] Further investigation has yet to determine the sensitivity and specificity of pro-Apolipoprotein A1 as a novel candidate biomarker in patients with chronic liver disease and HCC, but our pilot study suggests that its up-regulation is specifically a feature in the serum of patients with HCC.

## Conclusion

While non-hypothesis driven methodologies may be criticised by some as 'fishing expeditions', encouraging agreement is beginning to emerge when comparing data generated by proteomic techniques in the field of chronic liver disease, particularly when it is interpreted in the context of the ever growing academic literature generated by gene expression profiling. The data presented in this pilot study have identified a pattern of serum apolipoproteins which, in combination with CD5L, can discriminate pre-cirrhotic NAFLD, cirrhotic NAFLD, and cirrhotic NAFLD with HCC. These methodologies, even in small pilot studies requiring additional validation, contribute significantly to this field and provide the hope that improved serum biomarkers may yet become available as surveillance, diagnostic and prognostic tools in patients with chronic liver disease.

## Abbreviations

NAFLD: Non-alcoholic fatty liver disease; ALD: alcoholic liver disease; AFP: alpha alphafetoprotein; CD5L: CD5 molecule-like; HCC: hepatocellular cancer; HBV: hepatitis B; HCV: hepatitis C; USS: ultrasound scan; APO: apolipoprotein; MALDI-TOF MS: matrix-assisted laser desorption ionization time of flight mass spectroscopy.

## Competing interests

The authors declare that they have no competing interests.

## Authors' contributions

JG supervised the serum preparation and all proteomic studies and performed all the peptide mass analyses, DC has collected samples and with supervision performed the proteomic analyses, western blotting, ELISA assays, GSB has directly supervised the ELISA assay optimisation and data collection, GLP isolated RNA from tissue samples and performed real-time PCR analyses, LM helped in data interpretation, collected liver biopsy samples and collated histological staging enabling tissue studies, BPK reconfirmed the proteomic 2D Gel data, SS, MH, CPD have contributed to the study design and recruited patients to the study, DMM and HLR conceived the study, contributed to its design and co-ordination, HLR has completed final data analyses and written the manuscript. All authors read and approved the final manuscript.

## Pre-publication history

The pre-publication history for this paper can be accessed here:

http://www.biomedcentral.com/1471-2407/9/271/prepub

## Supplementary Material

Additional File 1 **Spot 1 mass spectra**. Maldi-TOF MS spectra for spot 1. Click here for file

Additional File 2 **Spot 1 is ApoA1**. The protein summary report for spot 1, generated using Mascot Peptide Mass Fingerprint search program (Matrix Science Ltd), identifies it as ApoA1. Click here for file

Additional File 3 **Spot 2 mass spectra**. Maldi-TOF MS spectra for spot 2. Click here for file

Additional File 4 **Spot 2 is also ApoA1**. The protein summary report for spot 2, generated using Mascot Peptide Mass Fingerprint search program (Matrix Science Ltd), identifies it as ApoA1. Click here for file

Additional File 5 **Spot 3 mass spectra**. Maldi-TOF MS spectra for spot 3. Click here for file

Additional File 6 **Spot 3 is Pro- ApoA1**. The protein summary report for spot 3, generated using Mascot Peptide Mass Fingerprint search program (Matrix Science Ltd), is compatible with it being Pro- ApoA1. Click here for file

Additional File 7 **Spot 4 mass spectra**. Maldi-TOF MS spectra for spot 4. Click here for file

Additional File 8 **Spot 4 is ApoA4**. The protein summary report for spot 4, generated using Mascot Peptide Mass Fingerprint search program (Matrix Science Ltd), identifies it as ApoA4. Click here for file

Additional File 9 **Spot 5 mass spectra**. Maldi-TOF MS spectra for spot 5. Click here for file

Additional File 10 **Spot 5 is CD5L**. The protein summary report for spot 5, generated using Mascot Peptide Mass Fingerprint search program (Matrix Science Ltd), identifies it as CD5L. Click here for file

Additional File 11 **Spot 3 is confirmed as Pro-ApoA1**. The Zoomed MALDI spectra confirming the peptide differences between spots 1 and 2 versus spot 3. Click here for file
