# Supplementary figures and images for: A proteomic strategy to identify novel serum biomarkers for liver cirrhosis and hepatocellular cancer in individuals with fatty liver disease
Source: BMC Cancer. 2009 Aug 5;9:271. doi: 10.1186/1471-2407-9-271 (PMC2729079; doi:10.1186/1471-2407-9-271)

Voyager Spec #1[BP = 1301.6, 4192]

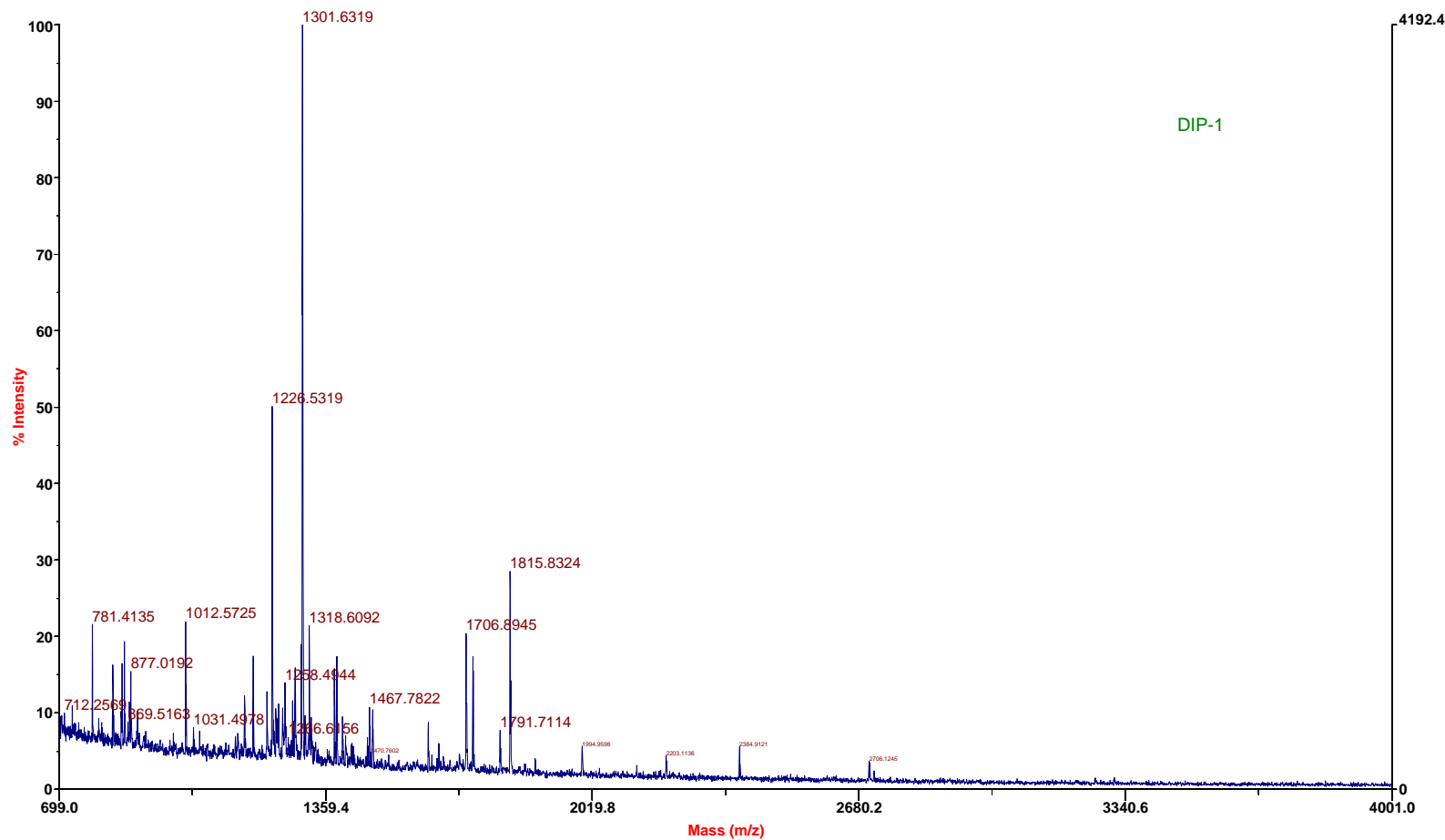

Voyager Spec #1=>AdvBC(32,0.5,0.1)=>NF0.7=>DI[BP = 1301.6, 35254]

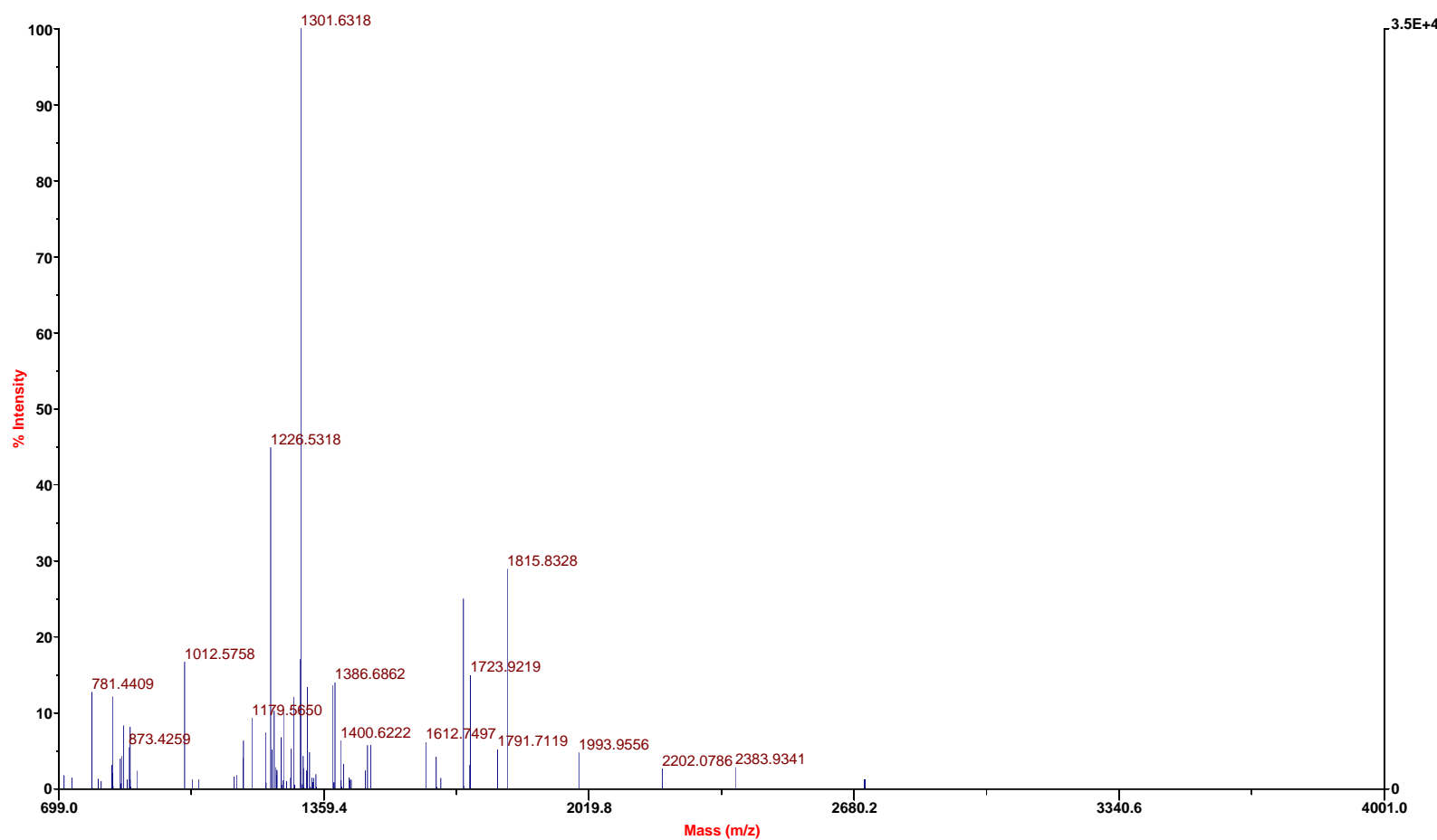

Supplement: Additional File 1 — Spot 1 mass spectra. Maldi-TOF MS spectra for spot 1. [file 1471-2407-9-271-S1.pdf]

Voyager Spec #1[BP = 1301.7, 6126]

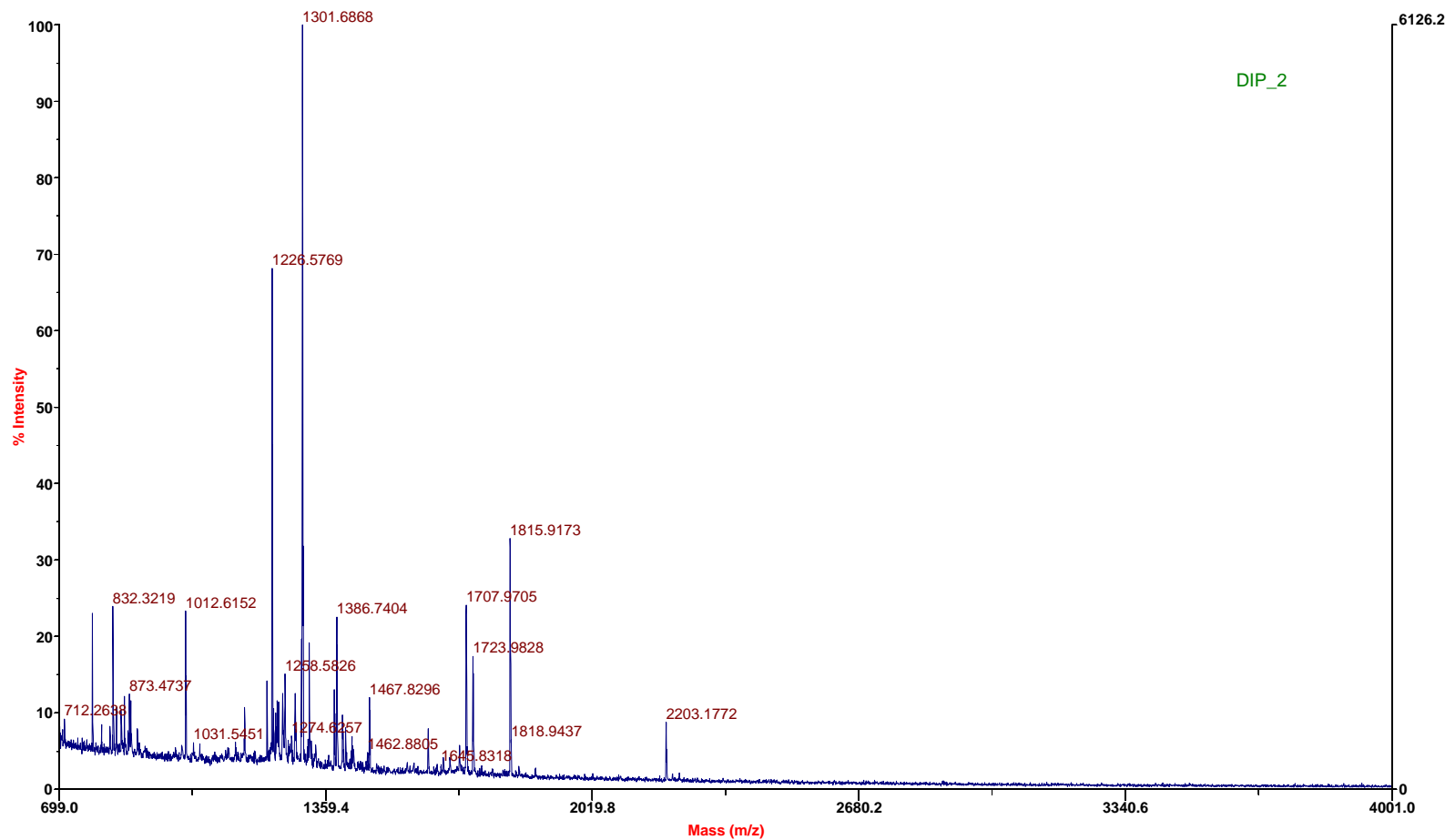

Voyager Spec #1=>AdvBC(32,0.5,0.1)=>NF0.7=>DI[BP = 1301.7, 49899]

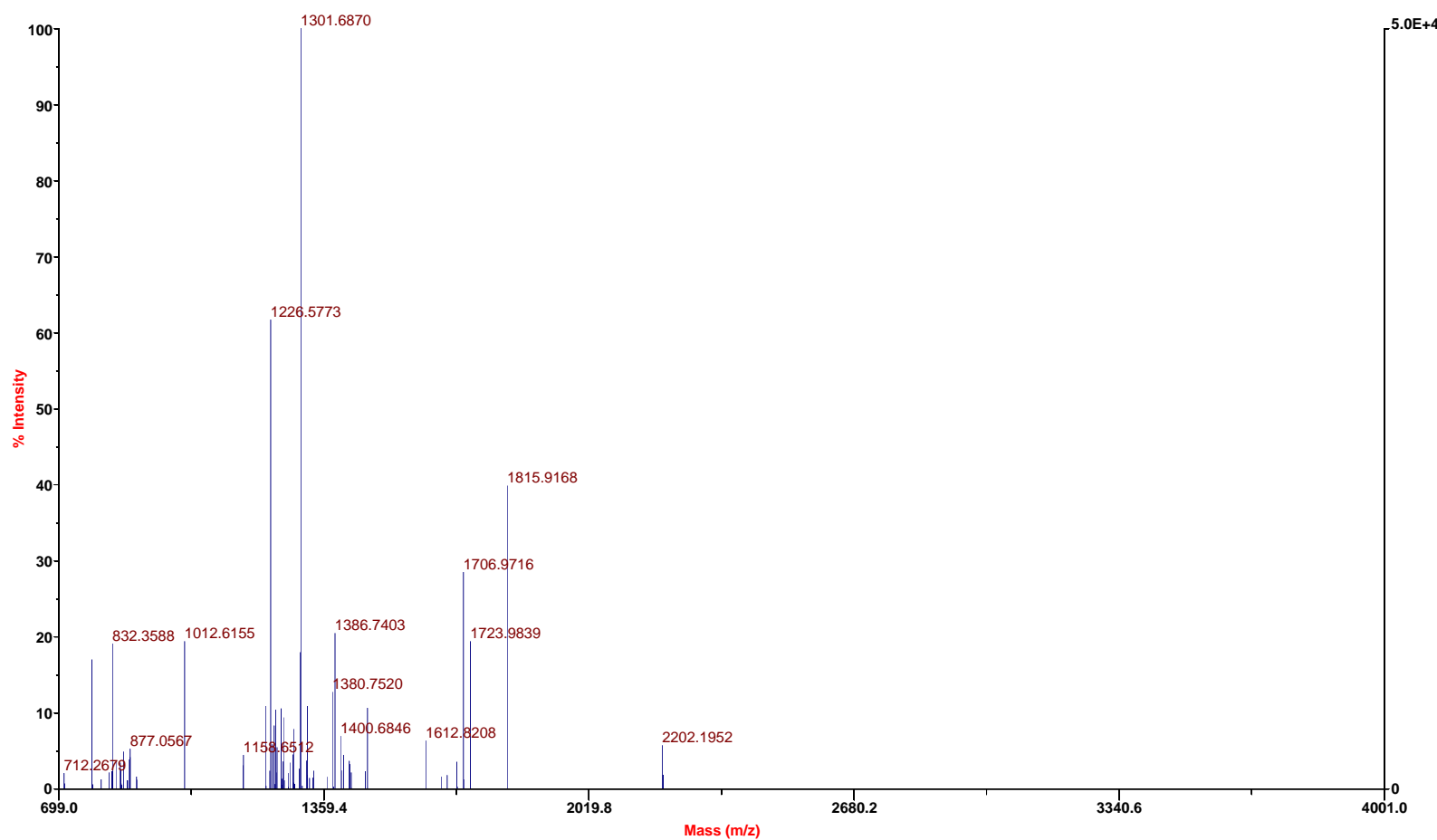

Supplement: Additional File 3 — Spot 2 mass spectra. Maldi-TOF MS spectra for spot 2. [file 1471-2407-9-271-S3.pdf]

# Voyager Spec #1[BP = 1301.7, 1420]

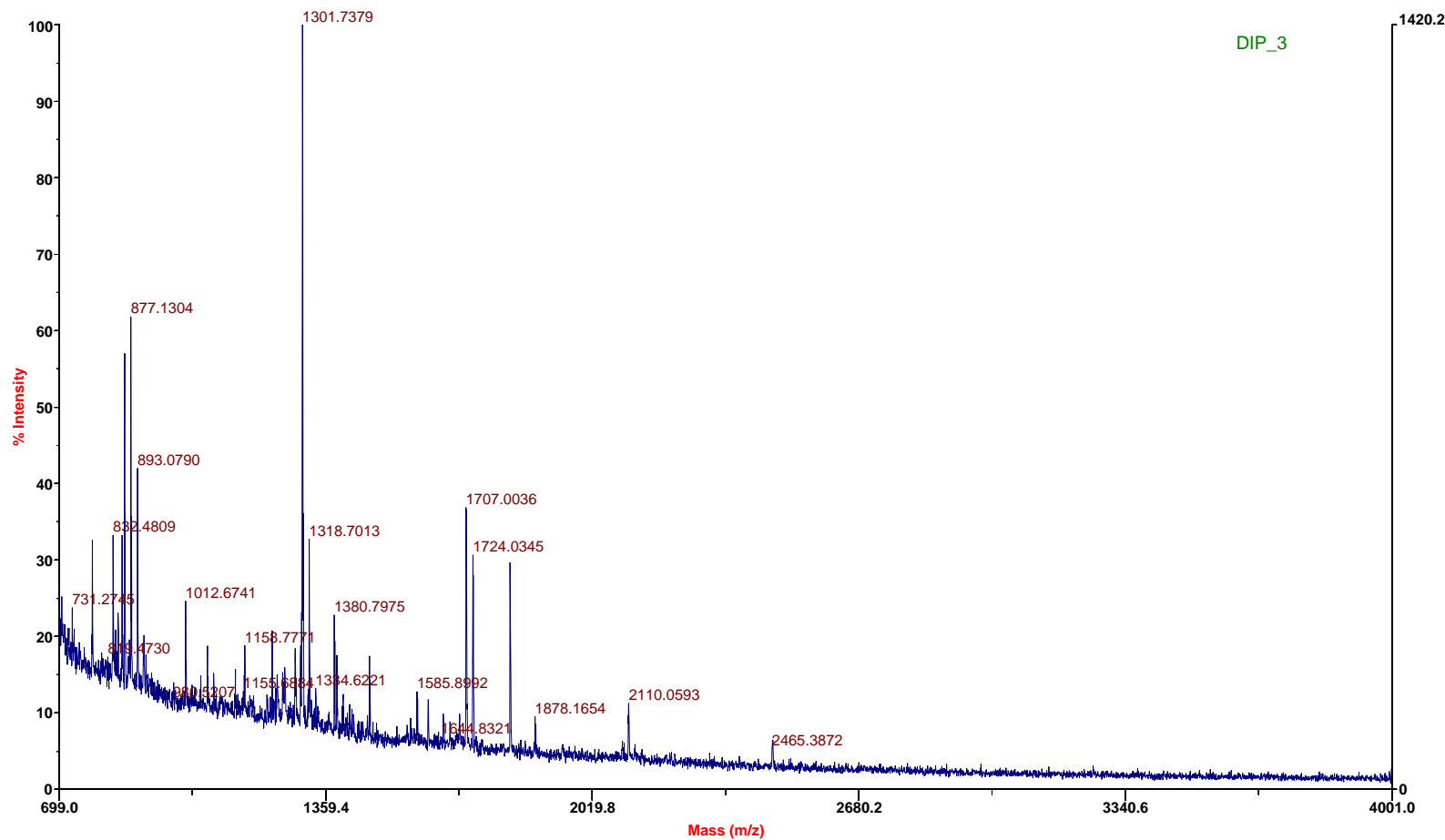

## Voyager Spec #1=>AdvBC(32,0.5,0.1)=>NF0.7=>DI[BP = 1301.7, 14140]

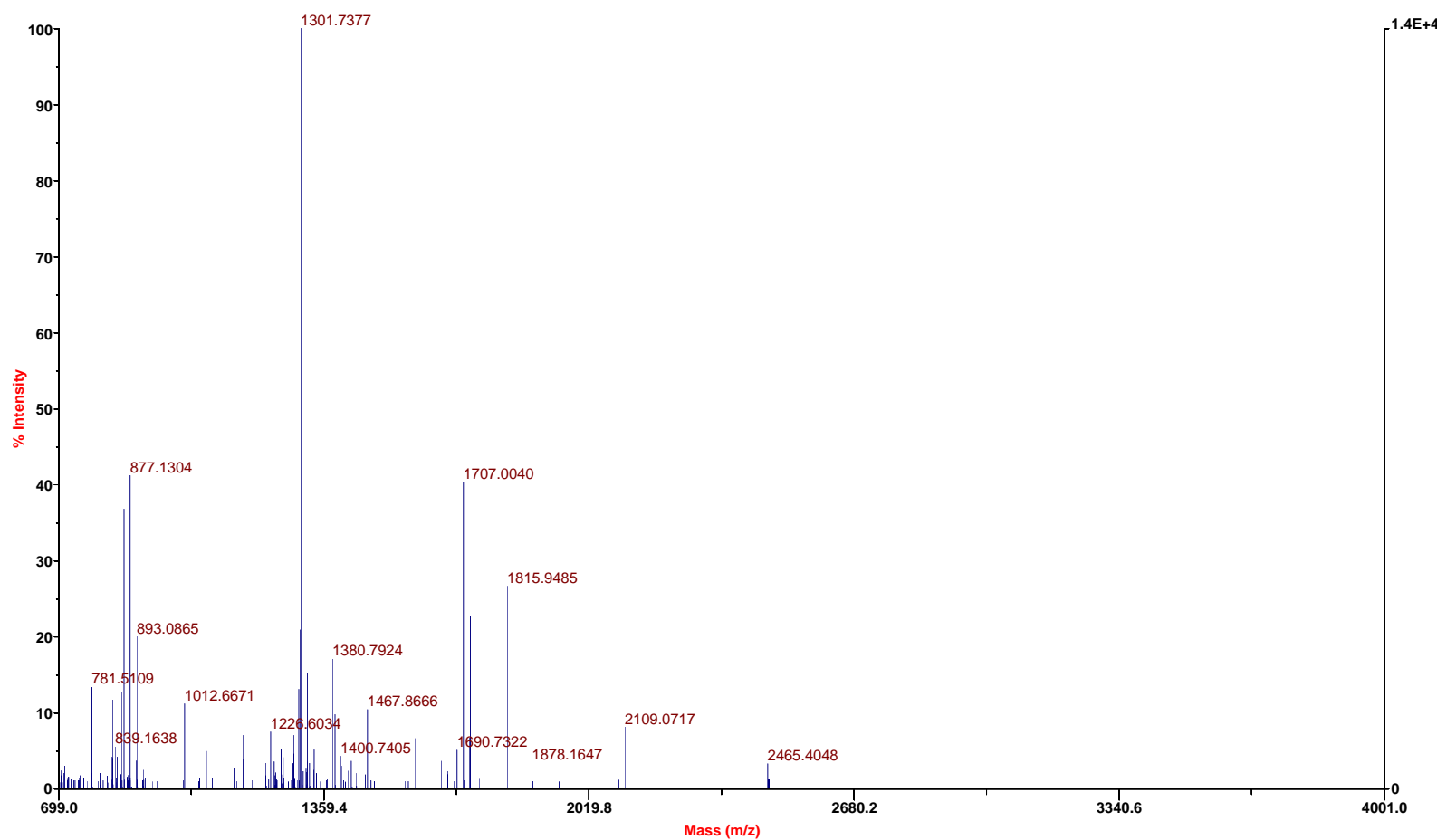

Supplement: Additional File 5 — Spot 3 mass spectra. Maldi-TOF MS spectra for spot 3. [file 1471-2407-9-271-S5.pdf]

# Voyager Spec #1[BP = 1104.5, 3944]

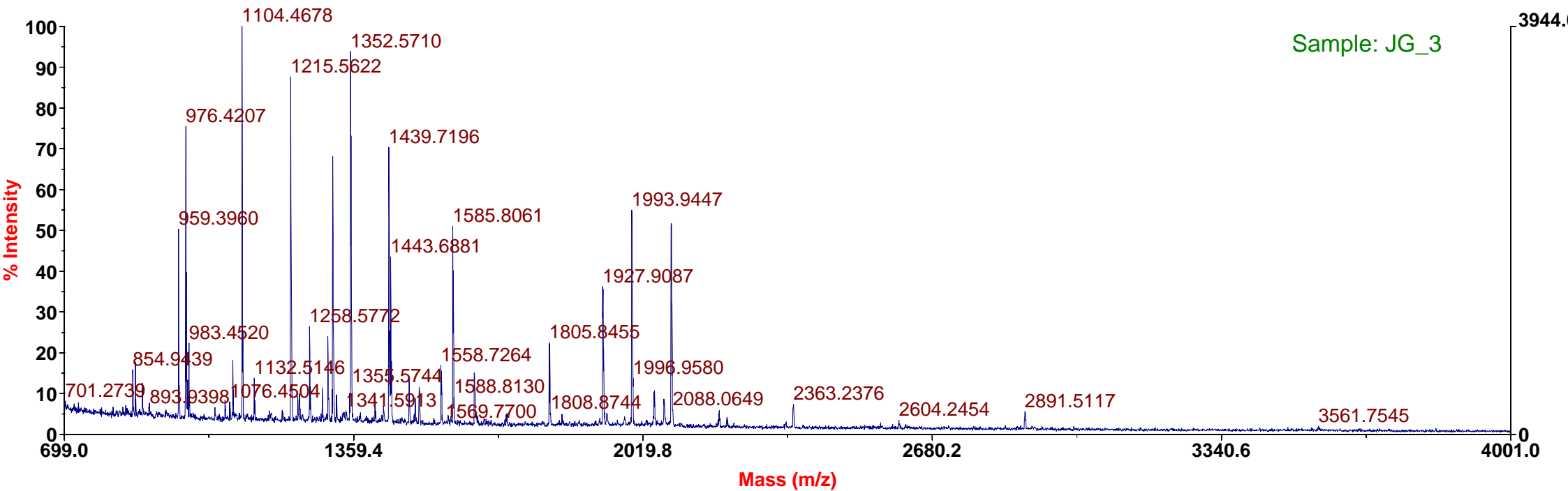

## Voyager Spec #1=>AdvBC(32,0.5,0.1)=>NF0.7=>DI[BP = 1215.6, 30138]

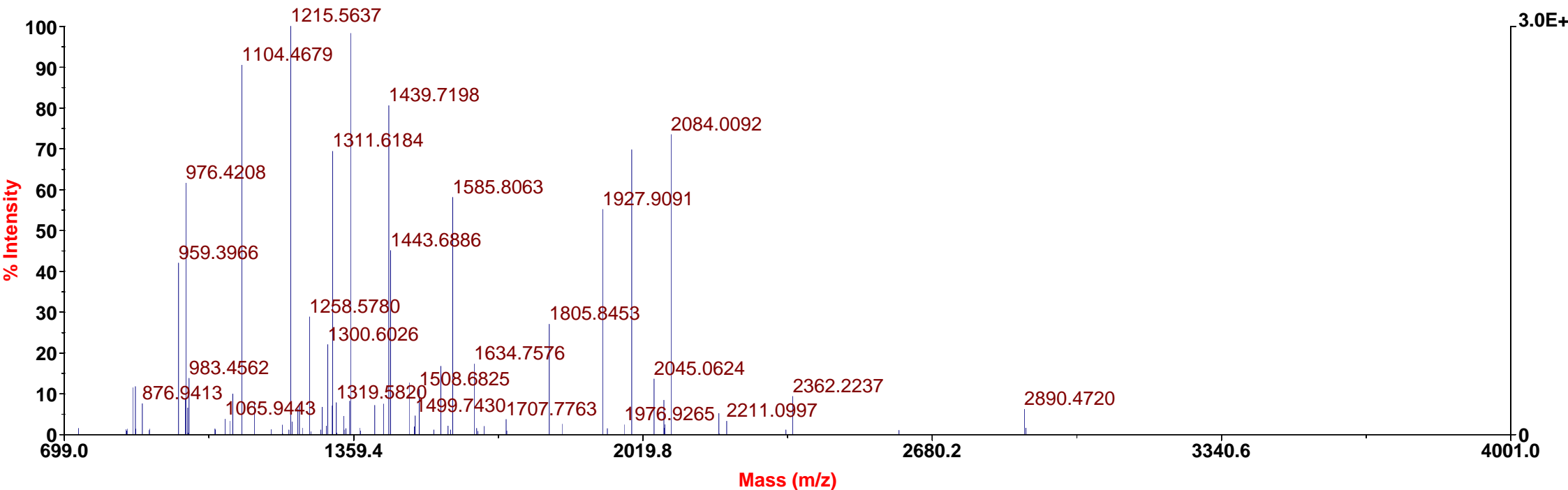

Supplement: Additional File 7 — Spot 4 mass spectra. Maldi-TOF MS spectra for spot 4. [file 1471-2407-9-271-S7.pdf]

# Voyager Spec #1[BP = 2064.8, 2890]

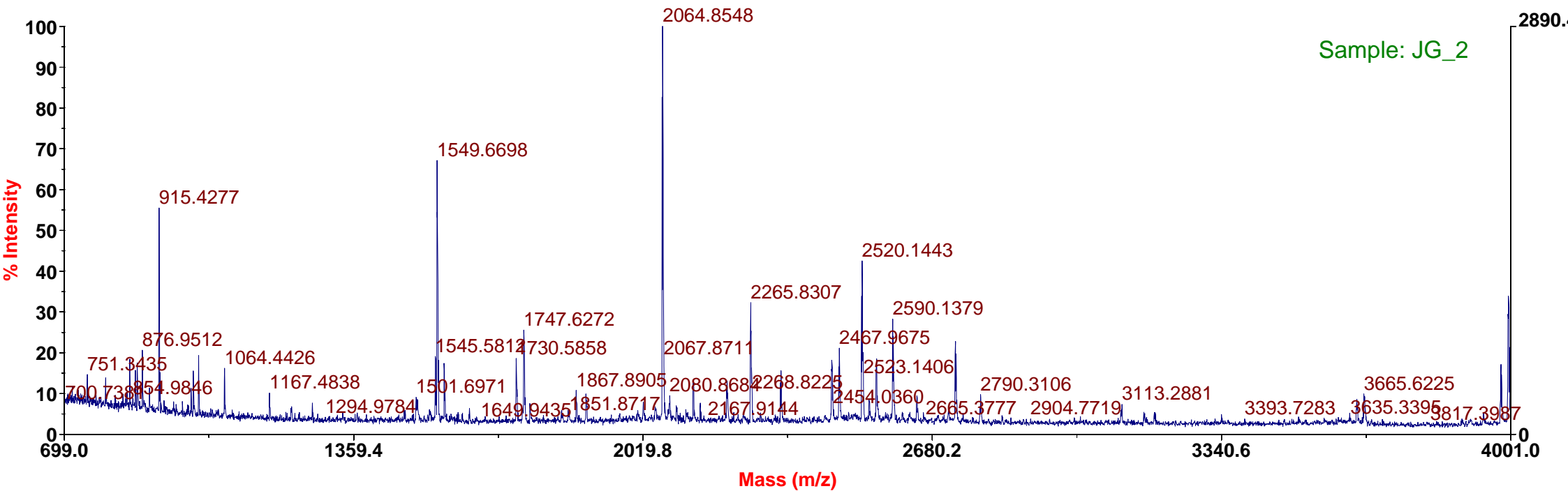

## Voyager Spec #1=>AdvBC(32,0.5,0.1)=>NF0.7=>DI[BP = 2063.9, 37000]

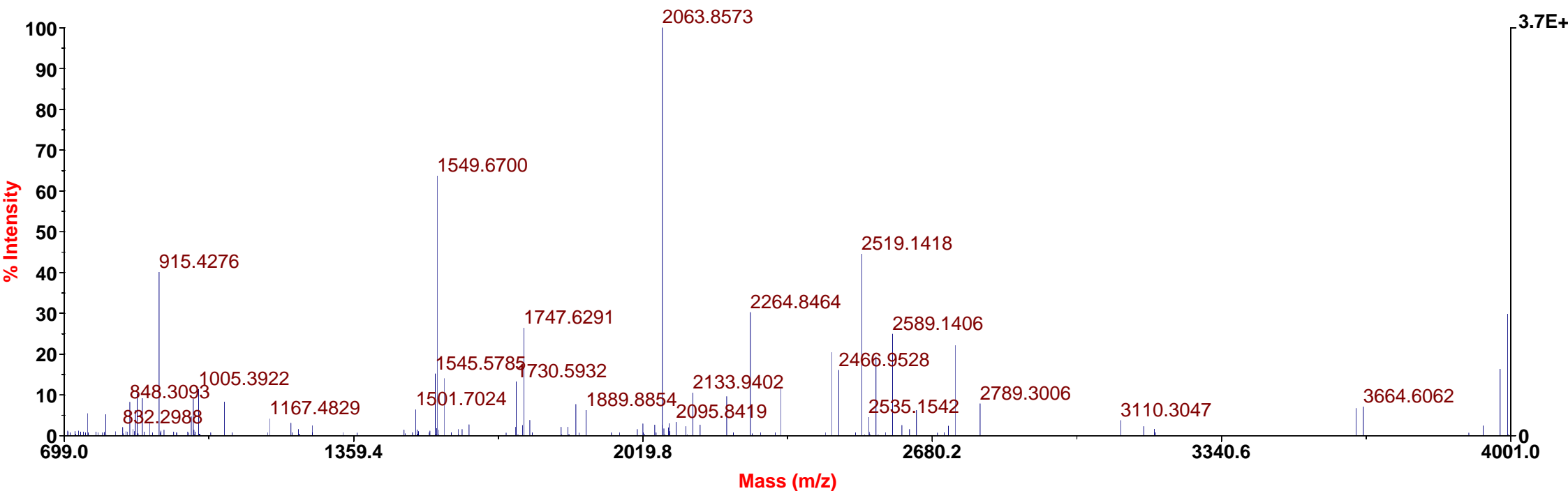

Supplement: Additional File 9 — Spot 5 mass spectra. Maldi-TOF MS spectra for spot 5. [file 1471-2407-9-271-S9.pdf]

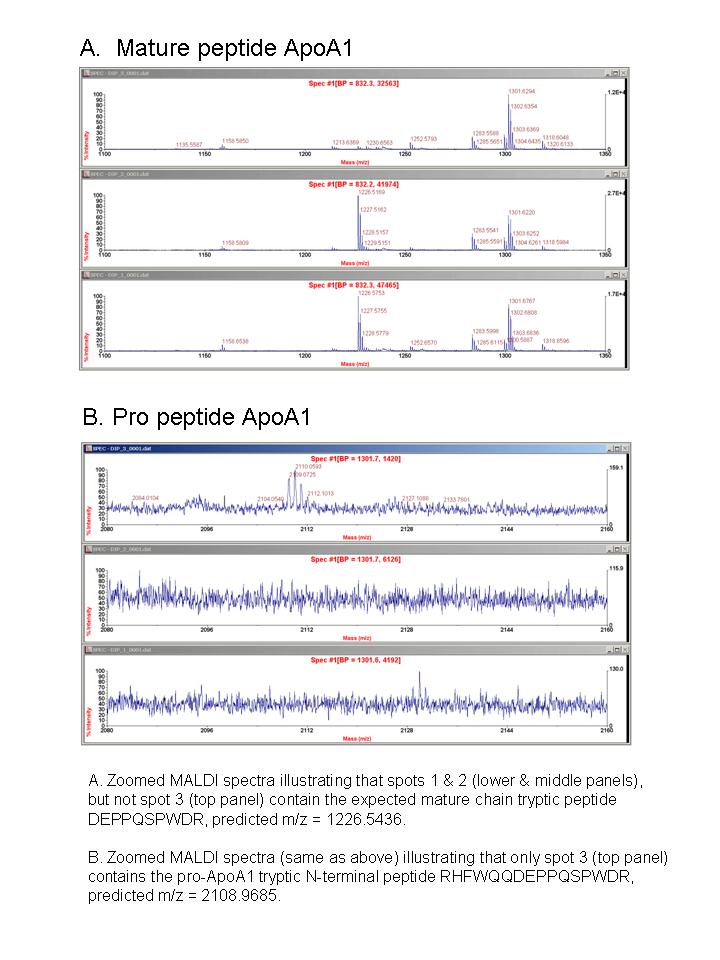

Supplement: Additional File 11 — Spot 3 is confirmed as Pro-ApoA1. The Zoomed MALDI spectra confirming the peptide differences between spots 1 and 2 versus spot 3. [file 1471-2407-9-271-S11.jpeg]
