# Supplementary material for: A proteomic strategy to identify novel serum biomarkers for liver cirrhosis and hepatocellular cancer in individuals with fatty liver disease
Source: BMC Cancer. 2009 Aug 5;9:271. doi: 10.1186/1471-2407-9-271 (PMC2729079; doi:10.1186/1471-2407-9-271)
Supplement: Additional File 2 — Spot 1 is ApoA1. The protein summary report for spot 1, generated using Mascot Peptide Mass Fingerprint search program (Matrix Science Ltd), identifies it as ApoA1. [file 1471-2407-9-271-S2.pdf]

Mascot Search Results

```
User          : Joe Gray
Email         : joe.gray@ncl.ac.uk
Search title  : DIP_1_0001.dat - SpecView
Database      : MSDB 20060831 (3239079 sequences; 1079594700 residues)
Timestamp     : 8 Jan 2007 at 11:27:46 GMT
Top Score     : 247 for CAA00975, APOA1 PROTEIN (FRAGMENT).- Homo sapiens (Human).
```

Probability Based Mowse Score

Protein score is -10*Log(P), where P is the probability that the observed match is a random event.
Protein scores greater than 78 are significant (p<0.05).

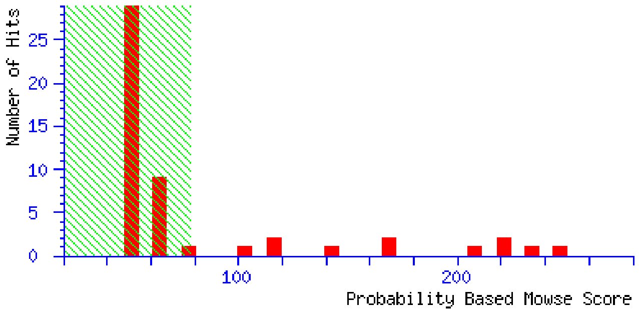

Protein Summary Report

Format As	Protein Summary	Help
	Significance threshold p< 0.05	Max. number of hits 50

Re-Search All   Search Unmatched

Index

	Accession	Mass	Score	Description
1.	CAA00975	28061	247	APOA1 PROTEIN (FRAGMENT).- Homo sapiens (Human).
2.	AAA51747	28944	229	HUMAPOAIC NID: - Homo sapiens
3.	LPHUA1	30759	220	apolipoprotein A-I precursor [validated] - human
4.	AAX42892	30872	220	AY890956 NID: - synthetic construct
5.	AAA35545	30745	204	HUMAPOAIP NID: - Homo sapiens
6.	CAA03490	23205	169	SEQUENCE 10 FROM PATENT WO9637608.- unidentified.
7.	1AV1A	23389	168	apolipoprotein a-i lipid-binding domain mutant N-TERMINAL MET, DEL(1-43), chain A - human
8.	Q8HZ97_PANTR	22075	137	Apolipoprotein A-I (Fragment).- Pan troglodytes (Chimpanzee).
9.	A26529	30700	115	apolipoprotein A-I precursor - crab-eating macaque
10.	JS0079	30716	115	apolipoprotein A-I precursor - baboon

11. Q8HZ95_PONPY	22116	105	Apolipoprotein A-I (Fragment).- Pongo pygmaeus (Orangutan).
12. CAD61352	18392	77	Sequence 2 from Patent WO02083898 precursor.- Homo sapiens (Human).
13. T47237	87729	64	myosin II heavy chain [imported] - Naegleria fowleri (fragment)
14. Q676V0_HPV62	73294	64	Putative replication protein E1.- Human papillomavirus type 62.
15. Q8TV91_METKA	29481	63	Predicted transcriptional regulator containing a DNA-binding HTH domain.- Methanopyrus kandleri.
16. O02568_9BILA	17537	62	Enolase (Fragment).- Cerebratulus cf. lacteus.
17. H87687	130186	62	helicase, UvrD/Rep family [imported] - Caulobacter crescentus
18. Q6DU55_PRUPE	67223	60	S-locus-like receptor protein kinase (Fragment).- Prunus persica (Peach).
19. Q7MS71_WOLSU	44173	59	Hypothetical protein hydC.- Wolinella succinogenes.
20. Q4IBZ3_GIBZE	31681	58	Hypothetical protein.- Gibberella zeae (Fusarium graminearum).
21. E96933	18193	57	hypothetical protein CAC0275 [imported] - Clostridium acetobutylicum
22. Q3KQC8_XENLA	28985	57	Hypothetical protein.- Xenopus laevis (African clawed frog).
23. Q9N0V7_RABIT	60174	56	Cystathionine beta-synthase (EC 4.2.1.22).- Oryctolagus cuniculus (Rabbit).
24. Q5NCF0_MOUSE	19181	56	Novel Sybindin-like family domain-containing protein.- Mus musculus (Mouse).
25. AAL97589	22480	56	AE010023 NID: - Streptococcus pyogenes MGAS8232
26. CAA40001	3140	55	BSDCIA NID: - Bacillus subtilis
27. Q219J6_RHOPB	54585	55	Transcriptional regulator, XRE family.- Rhodopseudomonas palustris (strain BisB18).
28. Q4LAF2_STAHJ	57597	55	Type I restriction-modification system DNA methylase.- Staphylococcus haemolyticus (strain JCSC1435).
29. AAM79212	22460	55	AE014074 NID: - Streptococcus pyogenes MGAS315
30. AAK33806	22479	54	AE006538 NID: - Streptococcus pyogenes M1 GAS
31. Q4WN94_ASPFU	42187	54	Actin binding protein, putative.- Aspergillus fumigatus (Sartorya fumigata).
32. Q2RK76_MOOTA	45037	54	UDP-N-acetylglucosamine 1-carboxyvinyltransferase.- Moorella thermoacetica (strain ATCC 39073).
33. Q8GC31_LEUCI	47411	53	Putative mobilization protein.- Leuconostoc citreum.
34. Q16V40_AEDAE	22771	53	Hypothetical protein.- Aedes aegypti (Yellowfever mosquito).
35. Q1EPB8_MUSAC	14866	53	Hypothetical protein.- Musa acuminata (Banana).
36. Q3WCG3_9ACTO	35265	53	Pyruvate dehydrogenase (Lipoamide) (EC 1.2.4.1).- Frankia sp. EAN1pec.
37. Q1RUA7_MEDTR	7463	53	Hypothetical protein.- Medicago truncatula (Barrel medic).
38. Q9C902_ARATH	86001	52	Protein kinase, putative; 19229-23534 (Hypothetical protein At3g06620).- Arabidopsis thaliana (Mouse-ear cress).
39. Q9TSS5_BOSIN	10093	52	BoLA-DRB3 protein (Fragment).- Bos indicus (Zebu).
40. Q5VU61_HUMAN	26265	52	Tropomyosin 3.- Homo sapiens (Human).
41. S11390	29003	52	tropomyosin 5 - mouse
42. Q54L29_DICDI	11417	52	Hypothetical protein.- Dictyostelium discoideum AX4.
43. Q5ZUP2_LEGPH	50366	51	Hypothetical protein.- Legionella pneumophila subsp. pneumophila (strain Philadelphia 1 / ATCC 33152 / DSM 7513).
44. A25530	29015	51	tropomyosin, fibroblast - human
45. Q4HE11_CAMCO	31370	51	Hypothetical protein.- Campylobacter coli RM2228.
46. Q8XVL4_RALSO	63203	51	Probable prolyl-trna synthetase protein (EC 6.1.1.15).- Ralstonia solanacearum (Pseudomonas solanacearum).
47. Q22LV9_TETTH	52292	51	Hypothetical protein.- Tetrahymena thermophila SB210.
48. Q9Y355_HUMAN	7429	51	Apolipoprotein A1 (Fragment).- Homo sapiens (Human).
49. T35704	16760	51	peptidylprolyl isomerase (EC 5.2.1.8) SC7H1.09 [similarity] - Streptomyces coelicolor
50. Q9KJG9_STRLI	16717	51	Peptidyl-prolyl cis-trans isomerase.- Streptomyces lividans.

Results List

1. CAA00975 **Mass:** 28061 **Score:** 247 **Expect:** 6.5e-19 **Queries matched:** 23

APOA1 PROTEIN (FRAGMENT).- Homo sapiens (Human).

Observed	Mr(expt)	Mr(calc)	Delta	Start	End	Miss	Peptide
781.4409	780.4336	780.4242	0.0094	154 -	160	0	R.AHVDALR.T
831.3855	830.3783	830.4286	-0.0503	189 -	195	0	R.LAEYHAK.A
873.4259	872.4186	872.4352	-0.0165	124 -	131	0	R.AELQEGAR.Q
1012.5758	1011.5686	1011.5713	-0.0027	207 -	215	0	K.AKPALEDLR.Q

1157.6034	1156.5962	1156.6200	-0.0238	178 - 188	1	R.LEALKENGGAR.L
1226.5318	1225.5245	1225.5364	-0.0118	1 - 10	0	-.DEPPQSPWDR.V
1230.6543	1229.6470	1229.7019	-0.0549	216 - 226	0	R.QGLLPVLESFK.V
1235.5913	1234.5840	1234.6809	-0.0969	13 - 23	0	K.DLATVYVDVLK.D
1252.6284	1251.6211	1251.6135	0.0076	97 - 106	0	K.VQPYLDDFQK.K
1283.5580	1282.5508	1282.5652	-0.0144	108 - 116	0	K.WQEEMELYR.Q
1299.5558	1298.5485	1298.5601	-0.0116	108 - 116	0	K.WQEEMELYR.Q + Oxidation (M)
1301.6318	1300.6245	1300.6411	-0.0166	161 - 171	0	R.THLAPYSDELR.Q
1307.6958	1306.6885	1306.6187	0.0698	78 - 88	1	K.ETEGLRQEMSK.D
1318.6086	1317.6013	1317.6347	-0.0334	141 - 151	1	K.LSPLGEEMRDR.A + Oxidation (M)
1323.6033	1322.5960	1322.6136	-0.0176	78 - 88	1	K.ETEGLRQEMSK.D + Oxidation (M)
1380.6922	1379.6849	1379.7085	-0.0236	97 - 107	1	K.VQPYLDDFQKK.W
1386.6862	1385.6789	1385.7078	-0.0289	227 - 238	0	K.VSFLSALEEYTK.K
1400.6222	1399.6149	1399.6619	-0.0470	28 - 40	0	R.DYVSQFEGSALGK.Q
1462.8200	1461.8127	1461.8442	-0.0315	11 - 23	1	R.VKDLATVYVDVLK.D
1467.7784	1466.7712	1466.7841	-0.0129	119 - 131	1	K.VEPLRAELQEGAR.Q
1612.7497	1611.7424	1611.7780	-0.0356	46 - 59	0	K.LLDNWDSVTSTFSK.L
1815.8328	1814.8255	1814.8434	-0.0179	24 - 40	1	K.DSGRDYVSQFEGSALGK.Q
2202.0786	2201.0713	2201.1116	-0.0403	60 - 77	1	K.LREQLGPVTQEFWDNLEK.E

No match to: 832.3686, 852.4759, 855.0295, 861.0514, 877.0207, 893.0028, 1158.5981, 1179.5649, 1213.6598, 1238.6094, 1240.5125, 1242.5535, 1258.5465, 1277.6779, 1308.6351, 1316.5630, 1317.6197, 1408.7039, 1475.7529, 1638.8350, 1706.8966, 1722.8620, 1723.9219, 1791.7119, 1993.9556, 2383.9341

2. AAA51747 **Mass:** 28944 **Score:** **229** **Expect:** 4.1e-17 **Queries matched:** 22

HUMAPOAIC NID: - Homo sapiens

Observed	Mr(expt)	Mr(calc)	Delta	Start End	Miss	Peptide
781.4409	780.4336	780.4242	0.0094	160 - 166	0	R.AHVDALR.T
831.3855	830.3783	830.4286	-0.0503	195 - 201	0	R.LAEYHAK.A
873.4259	872.4186	872.4352	-0.0165	130 - 137	0	R.AELQEGAR.Q
1012.5758	1011.5686	1011.5713	-0.0027	213 - 221	0	K.AKPALEDLR.Q
1157.6034	1156.5962	1156.6200	-0.0238	184 - 194	1	R.LEALKENGGAR.L
1230.6543	1229.6470	1229.7019	-0.0549	222 - 232	0	R.QGLLPVLESFK.V
1235.5913	1234.5840	1234.6809	-0.0969	19 - 29	0	K.DLATVYVDVLK.D
1252.6284	1251.6211	1251.6135	0.0076	103 - 112	0	K.VQPYLDDFQK.K
1283.5580	1282.5508	1282.5652	-0.0144	114 - 122	0	K.WQEEMELYR.Q
1299.5558	1298.5485	1298.5601	-0.0116	114 - 122	0	K.WQEEMELYR.Q + Oxidation (M)
1301.6318	1300.6245	1300.6411	-0.0166	167 - 177	0	R.THLAPYSDELR.Q
1307.6958	1306.6885	1306.6187	0.0698	84 - 94	1	K.ETEGLRQEMSK.D
1318.6086	1317.6013	1317.6347	-0.0334	147 - 157	1	K.LSPLGEEMRDR.A + Oxidation (M)
1323.6033	1322.5960	1322.6136	-0.0176	84 - 94	1	K.ETEGLRQEMSK.D + Oxidation (M)
1380.6922	1379.6849	1379.7085	-0.0236	103 - 113	1	K.VQPYLDDFQKK.W
1386.6862	1385.6789	1385.7078	-0.0289	233 - 244	0	K.VSFLSALEEYTK.K
1400.6222	1399.6149	1399.6619	-0.0470	34 - 46	0	R.DYVSQFEGSALGK.Q
1462.8200	1461.8127	1461.8442	-0.0315	17 - 29	1	R.VKDLATVYVDVLK.D
1467.7784	1466.7712	1466.7841	-0.0129	125 - 137	1	K.VEPLRAELQEGAR.Q
1612.7497	1611.7424	1611.7780	-0.0356	52 - 65	0	K.LLDNWDSVTSTFSK.L
1815.8328	1814.8255	1814.8434	-0.0179	30 - 46	1	K.DSGRDYVSQFEGSALGK.Q
2202.0786	2201.0713	2201.1116	-0.0403	66 - 83	1	K.LREQLGPVTQEFWDNLEK.E

No match to: 832.3686, 852.4759, 855.0295, 861.0514, 877.0207, 893.0028, 1158.5981, 1179.5649, 1213.6598, 1226.5318, 1238.6094, 1240.5125, 1242.5535, 1258.5465, 1277.6779, 1308.6351, 1316.5630, 1317.6197, 1408.7039, 1475.7529, 1638.8350, 1706.8966, 1722.8620, 1723.9219, 1791.7119, 1993.9556, 2383.9341

3. LPHUA1 **Mass:** 30759 **Score:** **220** **Expect:** 3.2e-16 **Queries matched:** 22

apolipoprotein A-I precursor [validated] - human

Observed	Mr(expt)	Mr(calc)	Delta	Start End	Miss	Peptide

781.4409	780.4336	780.4242	0.0094	178	184	0	R.AHVDALR.T
831.3855	830.3783	830.4286	-0.0503	213	219	0	R.LAEYHAK.A
873.4259	872.4186	872.4352	-0.0165	148	155	0	R.AELQEGAR.Q
1012.5758	1011.5686	1011.5713	-0.0027	231	239	0	K.AKPALEDLR.Q
1157.6034	1156.5962	1156.6200	-0.0238	202	212	1	R.LEALKENGGAR.L
1230.6543	1229.6470	1229.7019	-0.0549	240	250	0	R.QGLLPVLESFK.V
1235.5913	1234.5840	1234.6809	-0.0969	37	47	0	K.DLATVYVDVLK.D
1252.6284	1251.6211	1251.6135	0.0076	121	130	0	K.VQPYLDDFQK.K
1283.5580	1282.5508	1282.5652	-0.0144	132	140	0	K.WQEEMELYR.Q
1299.5558	1298.5485	1298.5601	-0.0116	132	140	0	K.WQEEMELYR.Q + Oxidation (M)
1301.6318	1300.6245	1300.6411	-0.0166	185	195	0	R.THLAPYSDELR.Q
1307.6958	1306.6885	1306.6187	0.0698	102	112	1	K.ETEGLRQEMSK.D
1318.6086	1317.6013	1317.6347	-0.0334	165	175	1	K.LSPLGEEMRDR.A + Oxidation (M)
1323.6033	1322.5960	1322.6136	-0.0176	102	112	1	K.ETEGLRQEMSK.D + Oxidation (M)
1380.6922	1379.6849	1379.7085	-0.0236	121	131	1	K.VQPYLDDFQKK.W
1386.6862	1385.6789	1385.7078	-0.0289	251	262	0	K.VSFLSALEEYTK.K
1400.6222	1399.6149	1399.6619	-0.0470	52	64	0	R.DYVSQFEGSALGK.Q
1462.8200	1461.8127	1461.8442	-0.0315	35	47	1	R.VKDLATVYVDVLK.D
1467.7784	1466.7712	1466.7841	-0.0129	143	155	1	K.VEPLRAELQEGAR.Q
1612.7497	1611.7424	1611.7780	-0.0356	70	83	0	K.LLDNWDSVTSTFSK.L
1815.8328	1814.8255	1814.8434	-0.0179	48	64	1	K.DSGRDYVSQFEGSALGK.Q
2202.0786	2201.0713	2201.1116	-0.0403	84	101	1	K.LREQLGPVTQEFWDNLEK.E

No match to: 832.3686, 852.4759, 855.0295, 861.0514, 877.0207, 893.0028, 1158.5981, 1179.5649, 1213.6598, 1226.5318, 1238.6094, 1240.5125, 1242.5535, 1258.5465, 1277.6779, 1308.6351, 1316.5630, 1317.6197, 1408.7039, 1475.7529, 1638.8350, 1706.8966, 1722.8620, 1723.9219, 1791.7119, 1993.9556, 2383.9341

4. AAX42892 **Mass:** 30872 **Score:** **220** **Expect:** 3.2e-16 **Queries matched:** 22

AY890956 NID: - synthetic construct

Observed	Mr(expt)	Mr(calc)	Delta	Start	End	Miss	Peptide
781.4409	780.4336	780.4242	0.0094	178	184	0	R.AHVDALR.T
831.3855	830.3783	830.4286	-0.0503	213	219	0	R.LAEYHAK.A
873.4259	872.4186	872.4352	-0.0165	148	155	0	R.AELQEGAR.Q
1012.5758	1011.5686	1011.5713	-0.0027	231	239	0	K.AKPALEDLR.Q
1157.6034	1156.5962	1156.6200	-0.0238	202	212	1	R.LEALKENGGAR.L
1230.6543	1229.6470	1229.7019	-0.0549	240	250	0	R.QGLLPVLESFK.V
1235.5913	1234.5840	1234.6809	-0.0969	37	47	0	K.DLATVYVDVLK.D
1252.6284	1251.6211	1251.6135	0.0076	121	130	0	K.VQPYLDDFQK.K
1283.5580	1282.5508	1282.5652	-0.0144	132	140	0	K.WQEEMELYR.Q
1299.5558	1298.5485	1298.5601	-0.0116	132	140	0	K.WQEEMELYR.Q + Oxidation (M)
1301.6318	1300.6245	1300.6411	-0.0166	185	195	0	R.THLAPYSDELR.Q
1307.6958	1306.6885	1306.6187	0.0698	102	112	1	K.ETEGLRQEMSK.D
1318.6086	1317.6013	1317.6347	-0.0334	165	175	1	K.LSPLGEEMRDR.A + Oxidation (M)
1323.6033	1322.5960	1322.6136	-0.0176	102	112	1	K.ETEGLRQEMSK.D + Oxidation (M)
1380.6922	1379.6849	1379.7085	-0.0236	121	131	1	K.VQPYLDDFQKK.W
1386.6862	1385.6789	1385.7078	-0.0289	251	262	0	K.VSFLSALEEYTK.K
1400.6222	1399.6149	1399.6619	-0.0470	52	64	0	R.DYVSQFEGSALGK.Q
1462.8200	1461.8127	1461.8442	-0.0315	35	47	1	R.VKDLATVYVDVLK.D
1467.7784	1466.7712	1466.7841	-0.0129	143	155	1	K.VEPLRAELQEGAR.Q
1612.7497	1611.7424	1611.7780	-0.0356	70	83	0	K.LLDNWDSVTSTFSK.L
1815.8328	1814.8255	1814.8434	-0.0179	48	64	1	K.DSGRDYVSQFEGSALGK.Q
2202.0786	2201.0713	2201.1116	-0.0403	84	101	1	K.LREQLGPVTQEFWDNLEK.E

No match to: 832.3686, 852.4759, 855.0295, 861.0514, 877.0207, 893.0028, 1158.5981, 1179.5649, 1213.6598, 1226.5318, 1238.6094, 1240.5125, 1242.5535, 1258.5465, 1277.6779, 1308.6351, 1316.5630, 1317.6197, 1408.7039, 1475.7529, 1638.8350, 1706.8966, 1722.8620, 1723.9219, 1791.7119, 1993.9556, 2383.9341

5. AAA35545 **Mass:** 30745 **Score:** **204** **Expect:** 1.3e-14 **Queries matched:** 21

HUMAPOAIP NID: - Homo sapiens

Observed	Mr(expt)	Mr(calc)	Delta	Start		End	Miss	Peptide
781.4409	780.4336	780.4242	0.0094	178	-	184	0	R.AHVDALR.T
831.3855	830.3783	830.4286	-0.0503	213	-	219	0	R.LAEYHAK.A
873.4259	872.4186	872.4352	-0.0165	148	-	155	0	R.AELQEGAR.Q
1012.5758	1011.5686	1011.5713	-0.0027	231	-	239	0	K.AKPALEDLR.Q
1157.6034	1156.5962	1156.6200	-0.0238	202	-	212	1	R.LEALKENGGAR.L
1230.6543	1229.6470	1229.7019	-0.0549	240	-	250	0	R.QGLLPVLESFK.V
1235.5913	1234.5840	1234.6809	-0.0969	37	-	47	0	K.DLATVYVDVLK.D
1252.6284	1251.6211	1251.6135	0.0076	121	-	130	0	K.VQPYLDDFQK.K
1283.5580	1282.5508	1282.5652	-0.0144	132	-	140	0	K.WQEEMELYR.Q
1299.5558	1298.5485	1298.5601	-0.0116	132	-	140	0	K.WQEEMELYR.Q + Oxidation (M)
1301.6318	1300.6245	1300.6411	-0.0166	185	-	195	0	R.THLAPYSDELR.Q
1307.6958	1306.6885	1306.6187	0.0698	102	-	112	1	K.ETEGLRQEMSK.D
1318.6086	1317.6013	1317.6347	-0.0334	165	-	175	1	K.LSPLGEEMRDR.A + Oxidation (M)
1323.6033	1322.5960	1322.6136	-0.0176	102	-	112	1	K.ETEGLRQEMSK.D + Oxidation (M)
1380.6922	1379.6849	1379.7085	-0.0236	121	-	131	1	K.VQPYLDDFQKK.W
1386.6862	1385.6789	1385.7078	-0.0289	251	-	262	0	K.VSFLSALEEYTK.K
1400.6222	1399.6149	1399.6619	-0.0470	52	-	64	0	R.DYVSQFEGSALGK.Q
1462.8200	1461.8127	1461.8442	-0.0315	35	-	47	1	R.VKDLATVYVDVLK.D
1612.7497	1611.7424	1611.7780	-0.0356	70	-	83	0	K.LLDNWDSVTSTFSK.L
1815.8328	1814.8255	1814.8434	-0.0179	48	-	64	1	K.DSGRDYVSQFEGSALGK.Q
2202.0786	2201.0713	2201.1116	-0.0403	84	-	101	1	K.LREQLGPVTQEFWDNLEK.E

No match to: 832.3686, 852.4759, 855.0295, 861.0514, 877.0207, 893.0028, 1158.5981, 1179.5649, 1213.6598, 1226.5318, 1238.6094, 1240.5125, 1242.5535, 1258.5465, 1277.6779, 1308.6351, 1316.5630, 1317.6197, 1408.7039, 1467.7784, 1475.7529, 1638.8350, 1706.8966, 1722.8620, 1723.9219, 1791.7119, 1993.9556, 2383.9341

6. CAA03490 **Mass:** 23205 **Score:** **169** **Expect:** 4.1e-11 **Queries matched:** 18

SEQUENCE 10 FROM PATENT WO9637608.- unidentified.

Observed	Mr(expt)	Mr(calc)	Delta	Start		End	Miss	Peptide
781.4409	780.4336	780.4242	0.0094	111	-	117	0	R.AHVDALR.T
831.3855	830.3783	830.4286	-0.0503	146	-	152	0	R.LAEYHAK.A
873.4259	872.4186	872.4352	-0.0165	81	-	88	0	R.AELQEGAR.Q
1012.5758	1011.5686	1011.5713	-0.0027	164	-	172	0	K.AKPALEDLR.Q
1157.6034	1156.5962	1156.6200	-0.0238	135	-	145	1	R.LEALKENGGAR.L
1226.5318	1225.5245	1225.5986	-0.0740	107	-	117	1	R.DCARAHVDALR.T
1230.6543	1229.6470	1229.7019	-0.0549	173	-	183	0	R.QGLLPVLESFK.V
1252.6284	1251.6211	1251.6135	0.0076	54	-	63	0	K.VQPYLDDFQK.K
1283.5580	1282.5508	1282.5652	-0.0144	65	-	73	0	K.WQEEMELYR.Q
1299.5558	1298.5485	1298.5601	-0.0116	65	-	73	0	K.WQEEMELYR.Q + Oxidation (M)
1301.6318	1300.6245	1300.6411	-0.0166	118	-	128	0	R.THLAPYSDELR.Q
1307.6958	1306.6885	1306.6187	0.0698	35	-	45	1	K.ETEGLRQEMSK.D
1323.6033	1322.5960	1322.6136	-0.0176	35	-	45	1	K.ETEGLRQEMSK.D + Oxidation (M)
1380.6922	1379.6849	1379.7085	-0.0236	54	-	64	1	K.VQPYLDDFQKK.W
1386.6862	1385.6789	1385.7078	-0.0289	184	-	195	0	K.VSFLSALEEYTK.K
1467.7784	1466.7712	1466.7841	-0.0129	76	-	88	1	K.VEPLRAELQEGAR.Q
1612.7497	1611.7424	1611.7780	-0.0356	3	-	16	0	K.LLDNWDSVTSTFSK.L
2202.0786	2201.0713	2201.1116	-0.0403	17	-	34	1	K.LREQLGPVTQEFWDNLEK.E

No match to: 832.3686, 852.4759, 855.0295, 861.0514, 877.0207, 893.0028, 1158.5981, 1179.5649, 1213.6598, 1235.5913, 1238.6094, 1240.5125, 1242.5535, 1258.5465, 1277.6779, 1308.6351, 1316.5630, 1317.6197, 1318.6086, 1400.6222, 1408.7039, 1462.8200, 1475.7529, 1638.8350, 1706.8966, 1722.8620, 1723.9219, 1791.7119, 1815.8328, 1993.9556, 2383.9341

7. 1AV1A **Mass:** 23389 **Score:** **168** **Expect:** 5.1e-11 **Queries matched:** 18

apolipoprotein a-i lipid-binding domain mutant N-TERMINAL MET, DEL(1-43), chain A - human

Observed	Mr(expt)	Mr(calc)	Delta	Start		End	Miss	Peptide
781.4409	780.4336	780.4242	0.0094	112	-	118	0	R.AHVDALR.T
831.3855	830.3783	830.4286	-0.0503	147	-	153	0	R.LAEYHAK.A
873.4259	872.4186	872.4352	-0.0165	82	-	89	0	R.AELQEGAR.Q
1012.5758	1011.5686	1011.5713	-0.0027	165	-	173	0	K.AKPALEDLR.Q
1157.6034	1156.5962	1156.6200	-0.0238	136	-	146	1	R.LEALKENGGAR.L
1230.6543	1229.6470	1229.7019	-0.0549	174	-	184	0	R.QGLLPVLESFK.V
1252.6284	1251.6211	1251.6135	0.0076	55	-	64	0	K.VQPYLDDFQK.K
1283.5580	1282.5508	1282.5652	-0.0144	66	-	74	0	K.WQEEMELYR.Q
1299.5558	1298.5485	1298.5601	-0.0116	66	-	74	0	K.WQEEMELYR.Q + Oxidation (M)
1301.6318	1300.6245	1300.6411	-0.0166	119	-	129	0	R.THLAPYSDELR.Q
1307.6958	1306.6885	1306.6187	0.0698	36	-	46	1	K.ETEGLRQEMSK.D
1318.6086	1317.6013	1317.6347	-0.0334	99	-	109	1	K.LSPLGEEMRDR.A + Oxidation (M)
1323.6033	1322.5960	1322.6136	-0.0176	36	-	46	1	K.ETEGLRQEMSK.D + Oxidation (M)
1380.6922	1379.6849	1379.7085	-0.0236	55	-	65	1	K.VQPYLDDFQKK.W
1386.6862	1385.6789	1385.7078	-0.0289	185	-	196	0	K.VSFLSALEEYTK.K
1467.7784	1466.7712	1466.7841	-0.0129	77	-	89	1	K.VEPLRAELQEGAR.Q
1612.7497	1611.7424	1611.7780	-0.0356	4	-	17	0	K.LLDNWDSVTSTFSK.L
2202.0786	2201.0713	2201.1116	-0.0403	18	-	35	1	K.LREQLGPVTQEFWDNLEK.E

No match to: 832.3686, 852.4759, 855.0295, 861.0514, 877.0207, 893.0028, 1158.5981, 1179.5649, 1213.6598, 1226.5318, 1235.5913, 1238.6094, 1240.5125, 1242.5535, 1258.5465, 1277.6779, 1308.6351, 1316.5630, 1317.6197, 1400.6222, 1408.7039, 1462.8200, 1475.7529, 1638.8350, 1706.8966, 1722.8620, 1723.9219, 1791.7119, 1815.8328, 1993.9556, 2383.9341

8. Q8HZ97_PANTR **Mass:** 22075 **Score:** **137** **Expect:** 6.5e-08 **Queries matched:** 15

Apolipoprotein A-I (Fragment).- Pan troglodytes (Chimpanzee).

Observed	Mr(expt)	Mr(calc)	Delta	Start		End	Miss	Peptide
781.4409	780.4336	780.4242	0.0094	141	-	147	0	R.AHVDALR.T
831.3855	830.3783	830.4286	-0.0503	176	-	182	0	R.LAEYHAK.A
873.4259	872.4186	872.4352	-0.0165	111	-	118	0	R.AELQEGAR.Q
1157.6034	1156.5962	1156.6200	-0.0238	165	-	175	1	R.LEALKENGGAR.L
1252.6284	1251.6211	1251.6135	0.0076	84	-	93	0	K.VQPYLDDFQK.K
1283.5580	1282.5508	1282.5652	-0.0144	95	-	103	0	K.WQEEMELYR.Q
1299.5558	1298.5485	1298.5601	-0.0116	95	-	103	0	K.WQEEMELYR.Q + Oxidation (M)
1301.6318	1300.6245	1300.6411	-0.0166	148	-	158	0	R.THLAPYSDELR.Q
1307.6958	1306.6885	1306.6187	0.0698	65	-	75	1	K.ETEGLRQEMSK.D
1323.6033	1322.5960	1322.6136	-0.0176	65	-	75	1	K.ETEGLRQEMSK.D + Oxidation (M)
1380.6922	1379.6849	1379.7085	-0.0236	84	-	94	1	K.VQPYLDDFQKK.W
1400.6222	1399.6149	1399.6619	-0.0470	15	-	27	0	R.DYVSQFEGSALGK.Q
1612.7497	1611.7424	1611.7780	-0.0356	33	-	46	0	K.LLDNWDSVTSTFSK.L
1815.8328	1814.8255	1814.8434	-0.0179	11	-	27	1	K.DSGRDYVSQFEGSALGK.Q
2202.0786	2201.0713	2201.1116	-0.0403	47	-	64	1	K.LREQLGPVTQEFWDNLEK.E

No match to: 832.3686, 852.4759, 855.0295, 861.0514, 877.0207, 893.0028, 1012.5758, 1158.5981, 1179.5649, 1213.6598, 1226.5318, 1230.6543, 1235.5913, 1238.6094, 1240.5125, 1242.5535, 1258.5465, 1277.6779, 1308.6351, 1316.5630, 1317.6197, 1318.6086, 1386.6862, 1408.7039, 1462.8200, 1467.7784, 1475.7529, 1638.8350, 1706.8966, 1722.8620, 1723.9219, 1791.7119, 1993.9556, 2383.9341

9. A26529 **Mass:** 30700 **Score:** **115** **Expect:** 1e-05 **Queries matched:** 15

apolipoprotein A-I precursor - crab-eating macaque

Observed	Mr(expt)	Mr(calc)	Delta	Start		End	Miss	Peptide
781.4409	780.4336	780.4242	0.0094	178	-	184	0	R.AHVDALR.T
831.3855	830.3783	830.4286	-0.0503	213	-	219	0	R.LAEYHAK.A
1012.5758	1011.5686	1011.5713	-0.0027	231	-	239	0	K.AKPALEDLR.Q
1157.6034	1156.5962	1156.6200	-0.0238	202	-	212	1	R.LEALKENGGAR.L
1230.6543	1229.6470	1229.7019	-0.0549	240	-	250	0	R.QGLLPVLESFK.V

1252.6284	1251.6211	1251.6135	0.0076	121 - 130	0	K.VQPYLDDFQK.K
1283.5580	1282.5508	1282.5652	-0.0144	132 - 140	0	K.WQEEMELYR.Q
1299.5558	1298.5485	1298.5601	-0.0116	132 - 140	0	K.WQEEMELYR.Q + Oxidation (M)
1301.6318	1300.6245	1300.6411	-0.0166	185 - 195	0	R.THLAPYSDELR.Q
1307.6958	1306.6885	1306.6187	0.0698	102 - 112	1	K.ETEGLRQEMSK.D
1323.6033	1322.5960	1322.6136	-0.0176	102 - 112	1	K.ETEGLRQEMSK.D + Oxidation (M)
1380.6922	1379.6849	1379.7085	-0.0236	121 - 131	1	K.VQPYLDDFQKK.W
1386.6862	1385.6789	1385.7078	-0.0289	251 - 262	0	K.VSFLSALEEYTK.K
1400.6222	1399.6149	1399.6619	-0.0470	52 - 64	0	K.DYVSQFEGSALGK.Q
2202.0786	2201.0713	2201.1116	-0.0403	84 - 101	1	K.LREQLGPVTQEFWDNLEK.E

No match to: 832.3686, 852.4759, 855.0295, 861.0514, 873.4259, 877.0207, 893.0028, 1158.5981, 1179.5649, 1213.6598, 1226.5318, 1235.5913, 1238.6094, 1240.5125, 1242.5535, 1258.5465, 1277.6779, 1308.6351, 1316.5630, 1317.6197, 1318.6086, 1408.7039, 1462.8200, 1467.7784, 1475.7529, 1612.7497, 1638.8350, 1706.8966, 1722.8620, 1723.9219, 1791.7119, 1815.8328, 1993.9556, 2383.9341

10. JS0079 **Mass:** 30716 **Score:** **115** **Expect:** 1e-05 **Queries matched:** 15

apolipoprotein A-I precursor - baboon

Observed	Mr(expt)	Mr(calc)	Delta	Start - End	Miss	Peptide
781.4409	780.4336	780.4242	0.0094	178 - 184	0	R.AHVDALR.T
831.3855	830.3783	830.4286	-0.0503	213 - 219	0	R.LAEYHAK.A
1012.5758	1011.5686	1011.5713	-0.0027	231 - 239	0	K.AKPALEDLR.Q
1157.6034	1156.5962	1156.6200	-0.0238	202 - 212	1	R.LEALKENGGAR.L
1230.6543	1229.6470	1229.7019	-0.0549	240 - 250	0	R.QGLLPVLESFK.V
1252.6284	1251.6211	1251.6135	0.0076	121 - 130	0	K.VQPYLDDFQK.K
1283.5580	1282.5508	1282.5652	-0.0144	132 - 140	0	K.WQEEMELYR.Q
1299.5558	1298.5485	1298.5601	-0.0116	132 - 140	0	K.WQEEMELYR.Q + Oxidation (M)
1301.6318	1300.6245	1300.6411	-0.0166	185 - 195	0	R.THLAPYSDELR.Q
1307.6958	1306.6885	1306.6187	0.0698	102 - 112	1	K.ETEGLRQEMSK.D
1323.6033	1322.5960	1322.6136	-0.0176	102 - 112	1	K.ETEGLRQEMSK.D + Oxidation (M)
1380.6922	1379.6849	1379.7085	-0.0236	121 - 131	1	K.VQPYLDDFQKK.W
1386.6862	1385.6789	1385.7078	-0.0289	251 - 262	0	K.VSFLSALEEYTK.K
1400.6222	1399.6149	1399.6619	-0.0470	52 - 64	0	K.DYVSQFEGSALGK.Q
2202.0786	2201.0713	2201.1116	-0.0403	84 - 101	1	K.LREQLGPVTQEFWDNLEK.E

No match to: 832.3686, 852.4759, 855.0295, 861.0514, 873.4259, 877.0207, 893.0028, 1158.5981, 1179.5649, 1213.6598, 1226.5318, 1235.5913, 1238.6094, 1240.5125, 1242.5535, 1258.5465, 1277.6779, 1308.6351, 1316.5630, 1317.6197, 1318.6086, 1408.7039, 1462.8200, 1467.7784, 1475.7529, 1612.7497, 1638.8350, 1706.8966, 1722.8620, 1723.9219, 1791.7119, 1815.8328, 1993.9556, 2383.9341

11. Q8HZ95_PONPY **Mass:** 22116 **Score:** **105** **Expect:** 0.0001 **Queries matched:** 13

Apolipoprotein A-I (Fragment).- Pongo pygmaeus (Orangutan).

Observed	Mr(expt)	Mr(calc)	Delta	Start - End	Miss	Peptide
781.4409	780.4336	780.4242	0.0094	141 - 147	0	R.AHVDALR.T
831.3855	830.3783	830.4286	-0.0503	176 - 182	0	R.LAEYHAK.A
873.4259	872.4186	872.4352	-0.0165	111 - 118	0	R.AELQEGAR.Q
1157.6034	1156.5962	1156.6200	-0.0238	165 - 175	1	R.LEALKENGGAR.L
1252.6284	1251.6211	1251.6135	0.0076	84 - 93	0	K.VQPYLDDFQK.K
1283.5580	1282.5508	1282.5652	-0.0144	95 - 103	0	K.WQEEMELYR.Q
1299.5558	1298.5485	1298.5601	-0.0116	95 - 103	0	K.WQEEMELYR.Q + Oxidation (M)
1307.6958	1306.6885	1306.6187	0.0698	65 - 75	1	K.ETEGLRQEMSK.D
1323.6033	1322.5960	1322.6136	-0.0176	65 - 75	1	K.ETEGLRQEMSK.D + Oxidation (M)
1380.6922	1379.6849	1379.7085	-0.0236	84 - 94	1	K.VQPYLDDFQKK.W
1400.6222	1399.6149	1399.6619	-0.0470	15 - 27	0	R.DYVSQFEGSALGK.Q
1815.8328	1814.8255	1814.8434	-0.0179	11 - 27	1	K.DSGRDYVSQFEGSALGK.Q
2202.0786	2201.0713	2201.1116	-0.0403	47 - 64	1	K.LREQLGPVTQEFWDNLEK.E

No match to: 832.3686, 852.4759, 855.0295, 861.0514, 877.0207, 893.0028, 1012.5758, 1158.5981, 1179.5649, 1213.6598, 1226.5318, 1230.6543, 1235.5913, 1238.6094, 1240.5125, 1242.5535, 1258.5465, 1277.6779, 1301.6318, 1308.6351, 1316.5630, 1317.6197, 1318.6086, 1386.6862, 1408.7039, 1462.8200, 1467.7784, 1475.7529, 1612.7497, 1638.8350, 1706.8966, 1722.8620, 1723.9219, 1791.7119, 1993.9556, 2383.9341

12. CAD61352 **Mass:** 18392 **Score:** 77 **Expect:** 0.062 **Queries matched:** 10

Sequence 2 from Patent WO02083898 precursor.- Homo sapiens (Human).

Observed	Mr(expt)	Mr(calc)	Delta	Start	End	Miss	Peptide
1235.5913	1234.5840	1234.6809	-0.0969	37 -	47	0	K.DLATVYVDVLK.D
1252.6284	1251.6211	1251.6135	0.0076	121 -	130	0	K.VQPYLDDFQK.K
1283.5580	1282.5508	1282.5652	-0.0144	132 -	140	0	K.WQEEMELYR.Q
1299.5558	1298.5485	1298.5601	-0.0116	132 -	140	0	K.WQEEMELYR.Q + Oxidation (M)
1307.6958	1306.6885	1306.6187	0.0698	102 -	112	1	K.ETEGLRQEMSK.D
1323.6033	1322.5960	1322.6136	-0.0176	102 -	112	1	K.ETEGLRQEMSK.D + Oxidation (M)
1380.6922	1379.6849	1379.7085	-0.0236	121 -	131	1	K.VQPYLDDFQKK.W
1400.6222	1399.6149	1399.6619	-0.0470	52 -	64	0	R.DYVSQFEGSALGK.Q
1462.8200	1461.8127	1461.8442	-0.0315	35 -	47	1	R.VKDLATVYVDVLK.D
1815.8328	1814.8255	1814.8434	-0.0179	48 -	64	1	K.DSGRDYVSQFEGSALGK.Q

No match to: 781.4409, 831.3855, 832.3686, 852.4759, 855.0295, 861.0514, 873.4259, 877.0207, 893.0028, 1012.5758, 1157.6034, 1158.5981, 1179.5649, 1213.6598, 1226.5318, 1230.6543, 1238.6094, 1240.5125, 1242.5535, 1258.5465, 1277.6779, 1301.6318, 1308.6351, 1316.5630, 1317.6197, 1318.6086, 1386.6862, 1408.7039, 1467.7784, 1475.7529, 1612.7497, 1638.8350, 1706.8966, 1722.8620, 1723.9219, 1791.7119, 1993.9556, 2202.0786, 2383.9341

13. T47237 **Mass:** 87729 **Score:** 64 **Expect:** 1.3 **Queries matched:** 15

myosin II heavy chain [imported] - Naegleria fowleri (fragment)

Observed	Mr(expt)	Mr(calc)	Delta	Start	End	Miss	Peptide
831.3855	830.3783	830.4497	-0.0715	488 -	494	1	R.VDVENKK.R
832.3686	831.3613	831.3835	-0.0221	271 -	277	1	K.KNDADNR.V
873.4259	872.4186	872.4715	-0.0529	597 -	604	1	R.AKLDEAAR.R
1226.5318	1225.5245	1225.4955	0.0291	524 -	533	0	K.SVEMECDELR.E + Oxidation (M)
1230.6543	1229.6470	1229.6251	0.0219	312 -	322	0	R.QLDESNNLVAK.L
1238.6094	1237.6021	1237.6224	-0.0202	111 -	121	1	K.KSTEMELSSVK.D
1252.6284	1251.6211	1251.6169	0.0042	468 -	477	0	R.EYEMQLAQLK.A
1283.5580	1282.5508	1282.5169	0.0338	524 -	533	0	K.SVEMECDELR.E + Carbamidomethyl (C); Oxidation (M)
1301.6318	1300.6245	1300.6120	0.0126	569 -	580	1	R.HSRASAEESATR.Q
1386.6862	1385.6789	1385.7262	-0.0473	311 -	322	1	K.RQLDESNNLVAK.L
1408.7039	1407.6966	1407.7180	-0.0214	467 -	477	1	R.REYEMQLAQLK.A
1462.8200	1461.8127	1461.6987	0.1140	733 -	746	0	K.ASPFSAGELIDDLQ.-
1638.8350	1637.8278	1637.7744	0.0534	200 -	214	1	R.SLSDKQNESTSLDSK.V
1722.8620	1721.8548	1721.7526	0.1022	406 -	419	1	K.QEMERVQSESENEK.S
1723.9219	1722.9146	1722.8094	0.1052	112 -	126	1	K.STEMELSSVKDDLNR.T

No match to: 781.4409, 852.4759, 855.0295, 861.0514, 877.0207, 893.0028, 1012.5758, 1157.6034, 1158.5981, 1179.5649, 1213.6598, 1235.5913, 1240.5125, 1242.5535, 1258.5465, 1277.6779, 1299.5558, 1307.6958, 1308.6351, 1316.5630, 1317.6197, 1318.6086, 1323.6033, 1380.6922, 1400.6222, 1467.7784, 1475.7529, 1612.7497, 1706.8966, 1791.7119, 1815.8328, 1993.9556, 2202.0786, 2383.9341

14. Q676V0_HPV62 **Mass:** 73294 **Score:** 64 **Expect:** 1.4 **Queries matched:** 14

Putative replication protein E1.- Human papillomavirus type 62.

Observed	Mr(expt)	Mr(calc)	Delta	Start	End	Miss	Peptide
873.4259	872.4186	872.3624	0.0563	171 -	179	0	R.GGGDGEPER.S
1157.6034	1156.5962	1156.6200	-0.0238	80 -	90	1	R.DDAAAVQALKR.K
1158.5981	1157.5908	1157.5863	0.0046	643 -	652	1	R.CVPGETNRLL.- + Carbamidomethyl (C)
1213.6598	1212.6525	1212.5750	0.0776	313 -	322	0	R.SAPCALYWFR.T
1230.6543	1229.6470	1229.5797	0.0673	419 -	428	0	R.MSMAQWIAHR.S
1235.5913	1234.5840	1234.5726	0.0114	403 -	412	1	K.DAITMCKYYK.R
1242.5535	1241.5462	1241.6148	-0.0686	400 -	409	1	K.YIKDAITMCK.Y + Carbamidomethyl (C)

1258.5465	1257.5392	1257.6097	-0.0705	400 - 409	1	K.YIKDAITMCK.Y + Carbamidomethyl (C); Oxidation (M)
1301.6318	1300.6245	1300.6737	-0.0491	450 - 460	0	K.IEFVTFMSALK.M + Oxidation (M)
1308.6351	1307.6279	1307.5890	0.0389	403 - 412	1	K.DAITMCKYYK.R + Carbamidomethyl (C); Oxidation (M)
1316.5630	1315.5558	1315.6732	-0.1174	537 - 548	0	R.SVLDGNQISIDR.K
1380.6922	1379.6849	1379.7269	-0.0420	113 - 124	1	R.LNAISLDRGHER.A
1386.6862	1385.6789	1385.6808	-0.0019	418 - 428	1	K.RMSMAQWIAHR.S
1467.7784	1466.7712	1466.7439	0.0273	469 - 482	1	K.KSCIVIYGPSDTGK.S

No match to: 781.4409, 831.3855, 832.3686, 852.4759, 855.0295, 861.0514, 877.0207, 893.0028, 1012.5758, 1179.5649, 1226.5318, 1238.6094, 1240.5125, 1252.6284, 1277.6779, 1283.5580, 1299.5558, 1307.6958, 1317.6197, 1318.6086, 1323.6033, 1400.6222, 1408.7039, 1462.8200, 1475.7529, 1612.7497, 1638.8350, 1706.8966, 1722.8620, 1723.9219, 1791.7119, 1815.8328, 1993.9556, 2202.0786, 2383.9341

15. Q8TV91_METKA **Mass:** 29481 **Score:** 63 **Expect:** 1.8 **Queries matched:** 9

Predicted transcriptional regulator containing a DNA-binding HTH domain.- Methanopyrus kandleri.

Observed	Mr(expt)	Mr(calc)	Delta	Start - End	Miss	Peptide
831.3855	830.3783	830.3956	-0.0173	1 - 7	0	-.MEEGPIR.V
1213.6598	1212.6525	1212.7264	-0.0738	240 - 250	0	K.ALVVITTPMLR.R
1301.6318	1300.6245	1300.6735	-0.0489	136 - 148	1	K.TGARAIAQADAEK.G
1307.6958	1306.6885	1306.6954	-0.0070	224 - 236	0	K.FGVVDGAIMAALK.G + Oxidation (M)
1323.6033	1322.5960	1322.6717	-0.0757	69 - 80	1	R.AEYVLTDKGAEK.A
1380.6922	1379.6849	1379.7408	-0.0559	2 - 13	1	M.EEGPIRVPNELK.D
1400.6222	1399.6149	1399.7381	-0.1231	211 - 223	0	R.AALNMLGIEPDLK.F + Oxidation (M)
1475.7529	1474.7456	1474.7602	-0.0146	103 - 115	1	R.MTWAAIAAEDIRK.G
1993.9556	1992.9483	1992.9390	0.0093	116 - 132	0	K.GEEIYLYMEDGLLYASK.S

No match to: 781.4409, 832.3686, 852.4759, 855.0295, 861.0514, 873.4259, 877.0207, 893.0028, 1012.5758, 1157.6034, 1158.5981, 1179.5649, 1226.5318, 1230.6543, 1235.5913, 1238.6094, 1240.5125, 1242.5535, 1252.6284, 1258.5465, 1277.6779, 1283.5580, 1299.5558, 1308.6351, 1316.5630, 1317.6197, 1318.6086, 1386.6862, 1408.7039, 1462.8200, 1467.7784, 1612.7497, 1638.8350, 1706.8966, 1722.8620, 1723.9219, 1791.7119, 1815.8328, 2202.0786, 2383.9341

16. O02568_9BILA **Mass:** 17537 **Score:** 62 **Expect:** 1.8 **Queries matched:** 7

Enolase (Fragment).- Cerebratulus cf. lacteus.

Observed	Mr(expt)	Mr(calc)	Delta	Start - End	Miss	Peptide
781.4409	780.4336	780.3766	0.0570	11 - 16	0	R.QIYDSR.G
1012.5758	1011.5686	1011.5825	-0.0140	2 - 10	1	M.STITKVHAR.Q
1308.6351	1307.6279	1307.6391	-0.0112	93 - 104	1	K.MLTLDGSDNKSK.F
1462.8200	1461.8127	1461.8013	0.0114	105 - 119	0	K.FGANAILGVSLAVCK.A
1723.9219	1722.9146	1722.9627	-0.0481	62 - 79	0	K.GVNTAVANVINSLGPAVK.G
1791.7119	1790.7047	1790.8798	-0.1752	17 - 33	0	R.GNPTVEVEVTTGQGTFR.A
1993.9556	1992.9483	1992.9310	0.0174	85 - 102	1	K.EQTAVDEKMLTLDGSDNK.S

No match to: 831.3855, 832.3686, 852.4759, 855.0295, 861.0514, 873.4259, 877.0207, 893.0028, 1157.6034, 1158.5981, 1179.5649, 1213.6598, 1226.5318, 1230.6543, 1235.5913, 1238.6094, 1240.5125, 1242.5535, 1252.6284, 1258.5465, 1277.6779, 1283.5580, 1299.5558, 1301.6318, 1307.6958, 1316.5630, 1317.6197, 1318.6086, 1323.6033, 1380.6922, 1386.6862, 1400.6222, 1408.7039, 1467.7784, 1475.7529, 1612.7497, 1638.8350, 1706.8966, 1722.8620, 1815.8328, 2202.0786, 2383.9341

17. H87687 **Mass:** 130186 **Score:** 62 **Expect:** 2 **Queries matched:** 15

helicase, UvrD/Rep family [imported] - Caulobacter crescentus

Observed	Mr(expt)	Mr(calc)	Delta	Start - End	Miss	Peptide
832.3686	831.3613	831.4086	-0.0473	905 - 911	0	K.ESQEALR.L
852.4759	851.4686	851.4977	-0.0291	1038 - 1044	1	R.RGELIHK.L
1157.6034	1156.5962	1156.6200	-0.0238	1123 - 1132	1	R.VDRLVVDDAR.V
1179.5649	1178.5577	1178.5931	-0.0354	961 - 971	0	R.TIGDGETAFLR.Y
1230.6543	1229.6470	1229.6186	0.0284	840 - 850	1	R.EVRVMTAHGSK.G + Oxidation (M)
1301.6318	1300.6245	1300.6160	0.0086	739 - 749	0	R.SEERPEWAAAR.A
1307.6958	1306.6885	1306.6517	0.0368	135 - 146	1	K.LVGEADARYDAK.S
1308.6351	1307.6279	1307.6258	0.0021	936 - 947	1	K.DDKVGGWYAAAR.A

1386.6862	1385.6789	1385.7190	-0.0401	1174 - 1186	0	R.TVEAALVWTDGPK.L
1400.6222	1399.6149	1399.7419	-0.1270	310 - 323	1	K.QDAKSAEALAAVAR.D
1475.7529	1474.7456	1474.7099	0.0357	258 - 271	1	R.CGGLAGAVSDAWRR.C + Carbamidomethyl (C)
1612.7497	1611.7424	1611.8416	-0.0991	43 - 56	1	R.LPHGGRIVMHDPQR.I
1638.8350	1637.8278	1637.9360	-0.1082	1187 - 1201	1	K.LMPVPEKVMALALAR.L
1815.8328	1814.8255	1815.0029	-0.1774	815 - 831	0	R.DLEALVADFAALDIIVK.R
2383.9341	2382.9268	2383.0927	-0.1659	587 - 606	1	R.ELPGEDREAWEKPLDEEGER.S

No match to: 781.4409, 831.3855, 855.0295, 861.0514, 873.4259, 877.0207, 893.0028, 1012.5758, 1158.5981, 1213.6598, 1226.5318, 1235.5913, 1238.6094, 1240.5125, 1242.5535, 1252.6284, 1258.5465, 1277.6779, 1283.5580, 1299.5558, 1316.5630, 1317.6197, 1318.6086, 1323.6033, 1380.6922, 1408.7039, 1462.8200, 1467.7784, 1706.8966, 1722.8620, 1723.9219, 1791.7119, 1993.9556, 2202.0786

18. Q6DU55_PRUPE **Mass:** 67223 **Score:** 60 **Expect:** 3.2 **Queries matched:** 12

S-locus-like receptor protein kinase (Fragment).- Prunus persica (Peach).

Observed	Mr(expt)	Mr(calc)	Delta	Start - End	Miss	Peptide
1012.5758	1011.5686	1011.5284	0.0402	357 - 364	0	K.INHMNLVR.T + Oxidation (M)
1158.5981	1157.5908	1157.6154	-0.0246	296 - 304	1	R.MYLYAELKK.A
1235.5913	1234.5840	1234.6557	-0.0717	194 - 204	0	R.SPTIEGSLYLR.L
1238.6094	1237.6021	1237.5873	0.0148	126 - 135	0	K.FKPTCSQSQR.V + Carbamidomethyl (C)
1299.5558	1298.5485	1298.5812	-0.0327	232 - 243	0	K.IGTSSMYDNPSK.T
1307.6958	1306.6885	1306.5876	0.1008	365 - 375	1	R.TWGFCSEGKHR.L
1318.6086	1317.6013	1317.6789	-0.0776	546 - 557	1	R.LEGQFGRNQAAK.M
1323.6033	1322.5960	1322.6579	-0.0618	53 - 63	1	R.RLTTDYDGNLR.L
1408.7039	1407.6966	1407.7292	-0.0326	126 - 137	1	K.FKPTCSQSQRVK.F
1467.7784	1466.7712	1466.7187	0.0524	276 - 288	1	R.SQGMSAPLVDKYR.L + Oxidation (M)
1723.9219	1722.9146	1722.7916	0.1230	558 - 572	1	K.MVEVGISCVEEDRNK.R + Oxidation (M)
1993.9556	1992.9483	1992.9244	0.0239	553 - 570	1	R.NQAAKMVEVGISCVEEDR.N + Oxidation (M)

No match to: 781.4409, 831.3855, 832.3686, 852.4759, 855.0295, 861.0514, 873.4259, 877.0207, 893.0028, 1157.6034, 1179.5649, 1213.6598, 1226.5318, 1230.6543, 1240.5125, 1242.5535, 1252.6284, 1258.5465, 1277.6779, 1283.5580, 1301.6318, 1308.6351, 1316.5630, 1317.6197, 1380.6922, 1386.6862, 1400.6222, 1462.8200, 1475.7529, 1612.7497, 1638.8350, 1706.8966, 1722.8620, 1791.7119, 1815.8328, 2202.0786, 2383.9341

19. Q7MS71_WOLSU **Mass:** 44173 **Score:** 59 **Expect:** 4.2 **Queries matched:** 9

Hypothetical protein hydC.- Wolinella succinogenes.

Observed	Mr(expt)	Mr(calc)	Delta	Start - End	Miss	Peptide
852.4759	851.4686	851.4388	0.0298	275 - 281	1	K.DAKEYVK.H
1012.5758	1011.5686	1011.5283	0.0402	1 - 9	1	-.MGRGEPLPR.I
1158.5981	1157.5908	1157.6022	-0.0113	83 - 90	0	R.YYFLHLFR.D
1213.6598	1212.6525	1212.6978	-0.0453	321 - 330	1	R.ISFIRELHAK.I
1301.6318	1300.6245	1300.6855	-0.0610	139 - 148	0	K.ILYFWGDFLK.K
1386.6862	1385.6789	1385.7489	-0.0700	263 - 274	1	K.IIMQDLKGHFGK.D
1612.7497	1611.7424	1611.7582	-0.0158	71 - 82	1	R.FSHFDFSWRDLR.Y
1815.8328	1814.8255	1814.9831	-0.1576	4 - 18	1	R.GEPLPRIYVWSPFVR.V
1993.9556	1992.9483	1993.0091	-0.0608	178 - 194	1	R.TRIIDSMVHGYKPYEGK.D

No match to: 781.4409, 831.3855, 832.3686, 855.0295, 861.0514, 873.4259, 877.0207, 893.0028, 1157.6034, 1179.5649, 1226.5318, 1230.6543, 1235.5913, 1238.6094, 1240.5125, 1242.5535, 1252.6284, 1258.5465, 1277.6779, 1283.5580, 1299.5558, 1307.6958, 1308.6351, 1316.5630, 1317.6197, 1318.6086, 1323.6033, 1380.6922, 1400.6222, 1408.7039, 1462.8200, 1467.7784, 1475.7529, 1638.8350, 1706.8966, 1722.8620, 1723.9219, 1791.7119, 2202.0786, 2383.9341

20. Q4IBZ3_GIBZE **Mass:** 31681 **Score:** 58 **Expect:** 4.9 **Queries matched:** 8

Hypothetical protein.- Gibberella zeae (Fusarium graminearum).

Observed	Mr(expt)	Mr(calc)	Delta	Start - End	Miss	Peptide
1230.6543	1229.6470	1229.6669	-0.0199	67 - 75	1	K.ISWQWNKLR.T
1258.5465	1257.5392	1257.5964	-0.0572	209 - 217	1	K.YCHEFKLYR.V
1308.6351	1307.6279	1307.6730	-0.0451	258 - 268	0	R.WSLMVVMSALR.M + Oxidation (M)

1408.7039	1407.6966	1407.6856	0.0110	223 - 234	0	K.VPNEYLAMASWK.E
1467.7784	1466.7712	1466.6976	0.0736	87 - 99	0	K.MFAVTEHSGLFGR.T + Oxidation (M)
1612.7497	1611.7424	1611.7740	-0.0315	275 - 289	0	R.SGQASLETNTSVQYK.-
1993.9556	1992.9483	1992.9767	-0.0284	223 - 239	1	K.VPNEYLAMASWKEAAWK.T
2383.9341	2382.9268	2383.1590	-0.2322	269 - 289	1	R.MWQLGRSGQASLETNTSVQYK.-

No match to: 781.4409, 831.3855, 832.3686, 852.4759, 855.0295, 861.0514, 873.4259, 877.0207, 893.0028, 1012.5758, 1157.6034, 1158.5981, 1179.5649, 1213.6598, 1226.5318, 1235.5913, 1238.6094, 1240.5125, 1242.5535, 1252.6284, 1277.6779, 1283.5580, 1299.5558, 1301.6318, 1307.6958, 1316.5630, 1317.6197, 1318.6086, 1323.6033, 1380.6922, 1386.6862, 1400.6222, 1462.8200, 1475.7529, 1638.8350, 1706.8966, 1722.8620, 1723.9219, 1791.7119, 1815.8328, 2202.0786

21. E96933 **Mass:** 18193 **Score:** 57 **Expect:** 5.9 **Queries matched:** 8

hypothetical protein CAC0275 [imported] - Clostridium acetobutylicum

Observed	Mr(expt)	Mr(calc)	Delta	Start End	Miss	Peptide
1012.5758	1011.5686	1011.5349	0.0337	144 - 152	0	K.ANHISTIEK.T
1157.6034	1156.5962	1156.6492	-0.0530	50 - 58	1	R.TIQYYKTLK.E
1242.5535	1241.5462	1241.6114	-0.0652	77 - 86	0	K.MSFLIDQFNK.K
1258.5465	1257.5392	1257.6063	-0.0671	77 - 86	0	K.MSFLIDQFNK.K + Oxidation (M)
1277.6779	1276.6706	1276.6411	0.0295	121 - 131	1	R.FVKNTSDTHTK.T
1283.5580	1282.5508	1282.5928	-0.0421	21 - 30	1	K.SEYEKIEEEK.H
1317.6197	1316.6124	1316.7122	-0.0997	38 - 49	1	R.VMSAVLVKEADR.T
1386.6862	1385.6789	1385.7013	-0.0224	77 - 87	1	K.MSFLIDQFNKK.T + Oxidation (M)

No match to: 781.4409, 831.3855, 832.3686, 852.4759, 855.0295, 861.0514, 873.4259, 877.0207, 893.0028, 1158.5981, 1179.5649, 1213.6598, 1226.5318, 1230.6543, 1235.5913, 1238.6094, 1240.5125, 1252.6284, 1299.5558, 1301.6318, 1307.6958, 1308.6351, 1316.5630, 1318.6086, 1323.6033, 1380.6922, 1400.6222, 1408.7039, 1462.8200, 1467.7784, 1475.7529, 1612.7497, 1638.8350, 1706.8966, 1722.8620, 1723.9219, 1791.7119, 1815.8328, 1993.9556, 2202.0786, 2383.9341

22. Q3KQC8_XENLA **Mass:** 28985 **Score:** 57 **Expect:** 6.8 **Queries matched:** 9

Hypothetical protein.- Xenopus laevis (African clawed frog).

Observed	Mr(expt)	Mr(calc)	Delta	Start End	Miss	Peptide
852.4759	851.4686	851.4211	0.0475	12 - 19	0	K.CGDLVFAK.M
1179.5649	1178.5577	1178.4687	0.0890	150 - 161	0	K.GNAEGSSDEEGK.L
1242.5535	1241.5462	1241.6127	-0.0665	20 - 29	1	K.MKGYPHWPAR.V
1258.5465	1257.5392	1257.6077	-0.0685	20 - 29	1	K.MKGYPHWPAR.V + Oxidation (M)
1307.6958	1306.6885	1306.5636	0.1248	149 - 161	1	K.KGNAEGSSDEEGK.L
1317.6197	1316.6124	1316.6096	0.0029	192 - 202	1	K.TKVEDDQEPEK.N
1386.6862	1385.6789	1385.7401	-0.0612	240 - 251	1	K.VEEKPVAEKETK.E
1612.7497	1611.7424	1611.7852	-0.0428	30 - 44	1	R.VDEVPEPNAKSSANR.Y
2202.0786	2201.0713	2200.9430	0.1283	203 - 221	1	K.NEKEDPQPEESVQMPDSDK.E

No match to: 781.4409, 831.3855, 832.3686, 855.0295, 861.0514, 873.4259, 877.0207, 893.0028, 1012.5758, 1157.6034, 1158.5981, 1213.6598, 1226.5318, 1230.6543, 1235.5913, 1238.6094, 1240.5125, 1252.6284, 1277.6779, 1283.5580, 1299.5558, 1301.6318, 1308.6351, 1316.5630, 1318.6086, 1323.6033, 1380.6922, 1400.6222, 1408.7039, 1462.8200, 1467.7784, 1475.7529, 1638.8350, 1706.8966, 1722.8620, 1723.9219, 1791.7119, 1815.8328, 1993.9556, 2383.9341

23. Q9N0V7_RABIT **Mass:** 60174 **Score:** 56 **Expect:** 7.6 **Queries matched:** 10

Cystathionine beta-synthase (EC 4.2.1.22).- Oryctolagus cuniculus (Rabbit).

Observed	Mr(expt)	Mr(calc)	Delta	Start End	Miss	Peptide
1157.6034	1156.5962	1156.6274	-0.0312	370 - 379	0	R.CVVILPDSVR.N + Carbamidomethyl (C)
1158.5981	1157.5908	1157.5175	0.0733	109 - 119	0	K.CEFFNAGGSVK.D
1235.5913	1234.5840	1234.6743	-0.0903	98 - 108	1	K.NFGLKCELLAK.C
1242.5535	1241.5462	1241.6040	-0.0578	326 - 336	1	K.STDKEAFAFAR.M
1308.6351	1307.6279	1307.6729	-0.0451	162 - 172	1	K.GYRCIIVMPEK.M
1323.6033	1322.5960	1322.6805	-0.0845	385 - 394	1	K.FLSDRWMLQK.G
1612.7497	1611.7424	1611.8005	-0.0580	212 - 224	0	K.QEIPNSHILDQYR.N
1723.9219	1722.9146	1722.8796	0.0350	370 - 384	1	R.CVVILPDSVRNYMSK.F

1815.8328	1814.8255	1814.8694	-0.0439	103	-	119	1	K.CELLAKCEFFNAGGSVK.D
2202.0786	2201.0713	2201.1547	-0.0834	337	-	359	0	R.MLIAQEGLLCGGSAGSAVAVAVK.A + Carbamidomethyl (C)

No match to: 781.4409, 831.3855, 832.3686, 852.4759, 855.0295, 861.0514, 873.4259, 877.0207, 893.0028, 1012.5758, 1179.5649, 1213.6598, 1226.5318, 1230.6543, 1238.6094, 1240.5125, 1252.6284, 1258.5465, 1277.6779, 1283.5580, 1299.5558, 1301.6318, 1307.6958, 1316.5630, 1317.6197, 1318.6086, 1380.6922, 1386.6862, 1400.6222, 1408.7039, 1462.8200, 1467.7784, 1475.7529, 1638.8350, 1706.8966, 1722.8620, 1791.7119, 1993.9556, 2383.9341

24. Q5NCF0_MOUSE **Mass:** 19181 **Score:** 56 **Expect:** 8.1 **Queries matched:** 8

Novel Sybindin-like family domain-containing protein.- Mus musculus (Mouse).

Observed	Mr(expt)	Mr(calc)	Delta	Start		End	Miss	Peptide
1157.6034	1156.5962	1156.5513	0.0449	59	-	68	0	K.DGFLSFQTSR.Y
1230.6543	1229.6470	1229.6300	0.0170	37	-	46	0	K.LMYGMLFSIR.S
1277.6779	1276.6706	1276.6564	0.0142	2	-	11	0	M.TVHNLYLFDR.N
1323.6033	1322.5960	1322.7241	-0.1281	142	-	152	0	R.RPTTHCLQVIR.M
1380.6922	1379.6849	1379.7456	-0.0607	142	-	152	0	R.RPTTHCLQVIR.M + Carbamidomethyl (C)
1386.6862	1385.6789	1385.7336	-0.0547	82	-	94	0	K.VVMNTDLGVGPIR.D + Oxidation (M)
1408.7039	1407.6966	1407.6969	-0.0002	1	-	11	0	-.MTVHNLYLFDR.N
1612.7497	1611.7424	1611.8297	-0.0872	69	-	81	1	R.YKLHYYETPTGIK.V

No match to: 781.4409, 831.3855, 832.3686, 852.4759, 855.0295, 861.0514, 873.4259, 877.0207, 893.0028, 1012.5758, 1158.5981, 1179.5649, 1213.6598, 1226.5318, 1235.5913, 1238.6094, 1240.5125, 1242.5535, 1252.6284, 1258.5465, 1283.5580, 1299.5558, 1301.6318, 1307.6958, 1308.6351, 1316.5630, 1317.6197, 1318.6086, 1400.6222, 1462.8200, 1467.7784, 1475.7529, 1638.8350, 1706.8966, 1722.8620, 1723.9219, 1791.7119, 1815.8328, 1993.9556, 2202.0786, 2383.9341

25. AAL97589 **Mass:** 22480 **Score:** 56 **Expect:** 8.7 **Queries matched:** 8

AE010023 NID: - Streptococcus pyogenes MGAS8232

Observed	Mr(expt)	Mr(calc)	Delta	Start		End	Miss	Peptide
1157.6034	1156.5962	1156.5434	0.0528	101	-	109	0	K.MIEEYLTSR.D + Oxidation (M)
1158.5981	1157.5908	1157.6516	-0.0608	54	-	64	1	K.NLARTSSKPGK.T
1235.5913	1234.5840	1234.7033	-0.1193	43	-	53	1	K.SSFINTILGRK.N
1301.6318	1300.6245	1300.6009	0.0236	128	-	137	0	K.EDIQMYDFLK.Y
1317.6197	1316.6124	1316.5958	0.0166	128	-	137	0	K.EDIQMYDFLK.Y + Oxidation (M)
1380.6922	1379.6849	1379.7700	-0.0851	138	-	149	0	K.YYDIPVIVVATK.A
1386.6862	1385.6789	1385.6826	-0.0037	174	-	185	0	K.SDTFIVFSSVER.I
1467.7784	1466.7712	1466.7405	0.0306	65	-	76	0	K.TQLLNFFNIDDK.L

No match to: 781.4409, 831.3855, 832.3686, 852.4759, 855.0295, 861.0514, 873.4259, 877.0207, 893.0028, 1012.5758, 1179.5649, 1213.6598, 1226.5318, 1230.6543, 1238.6094, 1240.5125, 1242.5535, 1252.6284, 1258.5465, 1277.6779, 1283.5580, 1299.5558, 1307.6958, 1308.6351, 1316.5630, 1318.6086, 1323.6033, 1400.6222, 1408.7039, 1462.8200, 1475.7529, 1612.7497, 1638.8350, 1706.8966, 1722.8620, 1723.9219, 1791.7119, 1815.8328, 1993.9556, 2202.0786, 2383.9341

26. CAA40001 **Mass:** 3140 **Score:** 55 **Expect:** 10 **Queries matched:** 4

BSDCIA NID: - Bacillus subtilis

Observed	Mr(expt)	Mr(calc)	Delta	Start		End	Miss	Peptide
1179.5649	1178.5577	1178.6183	-0.0606	15	-	24	0	K.YELLTEQVGK.Q
1252.6284	1251.6211	1251.5771	0.0440	1	-	10	0	-.PDVFDEVFER.T
1307.6958	1306.6885	1306.7132	-0.0247	14	-	24	1	R.KYELLTEQVGK.Q
1408.7039	1407.6966	1407.7245	-0.0279	15	-	26	1	K.YELLTEQVGKQT.-

No match to: 781.4409, 831.3855, 832.3686, 852.4759, 855.0295, 861.0514, 873.4259, 877.0207, 893.0028, 1012.5758, 1157.6034, 1158.5981, 1213.6598, 1226.5318, 1230.6543, 1235.5913, 1238.6094, 1240.5125, 1242.5535, 1258.5465, 1277.6779, 1283.5580, 1299.5558, 1301.6318, 1308.6351, 1316.5630, 1317.6197, 1318.6086, 1323.6033, 1380.6922, 1386.6862, 1400.6222, 1462.8200, 1467.7784, 1475.7529, 1612.7497, 1638.8350, 1706.8966, 1722.8620, 1723.9219, 1791.7119, 1815.8328, 1993.9556, 2202.0786, 2383.9341

27. Q219J6_RHOPB **Mass:** 54585 **Score:** 55 **Expect:** 10 **Queries matched:** 10

Transcriptional regulator, XRE family.- Rhodopseudomonas palustris (strain BisB18).

Observed	Mr(expt)	Mr(calc)	Delta	Start		End	Miss	Peptide

1213.6598	1212.6525	1212.6251	0.0275	117 - 126	1	R.LYAAYTEARR.G
1230.6543	1229.6470	1229.6516	-0.0046	143 - 152	1	R.FEANPIERVR.D
1238.6094	1237.6021	1237.6891	-0.0870	191 - 200	1	R.LREQHSIVTR.I
1299.5558	1298.5485	1298.6255	-0.0770	469 - 480	1	R.ASSFAFSNAREL.-
1307.6958	1306.6885	1306.5876	0.1008	304 - 314	0	R.FNAGFEQVCHR.L
1317.6197	1316.6124	1316.5714	0.0411	442 - 451	1	R.LCEREHCSQR.A + Carbamidomethyl (C)
1318.6086	1317.6013	1317.6459	-0.0446	126 - 137	1	R.RGETLAAQMADR.E
1408.7039	1407.6966	1407.8310	-0.1344	46 - 57	0	R.NQRPVTAQILLR.L
1467.7784	1466.7712	1466.8456	-0.0745	372 - 384	1	R.LLKQVIELPDGSR.Y
1638.8350	1637.8278	1637.8195	0.0083	102 - 116	0	R.DLAELCPGVTHSLQR.L

No match to: 781.4409, 831.3855, 832.3686, 852.4759, 855.0295, 861.0514, 873.4259, 877.0207, 893.0028, 1012.5758, 1157.6034, 1158.5981, 1179.5649, 1226.5318, 1235.5913, 1240.5125, 1242.5535, 1252.6284, 1258.5465, 1277.6779, 1283.5580, 1301.6318, 1308.6351, 1316.5630, 1323.6033, 1380.6922, 1386.6862, 1400.6222, 1462.8200, 1475.7529, 1612.7497, 1706.8966, 1722.8620, 1723.9219, 1791.7119, 1815.8328, 1993.9556, 2202.0786, 2383.9341

28. Q4LAF2_STAHJ **Mass:** 57597 **Score:** 55 **Expect:** 11 **Queries matched:** 10

Type I restriction-modification system DNA methylase.- Staphylococcus haemolyticus (strain JCSC1435).

Observed	Mr(expt)	Mr(calc)	Delta	Start - End	Miss	Peptide
831.3855	830.3783	830.4286	-0.0504	10 - 16	0	K.LWQAADK.L
1213.6598	1212.6525	1212.6713	-0.0188	104 - 114	1	K.ENESLKGVLPK.E
1240.5125	1239.5052	1239.5917	-0.0865	17 - 27	1	K.LRGSMDAAEYK.N
1252.6284	1251.6211	1251.6346	-0.0136	475 - 485	0	R.ITSELSEQFAK.S
1408.7039	1407.6966	1407.7357	-0.0391	474 - 485	1	K.RITSELSEQFAK.S
1467.7784	1466.7712	1466.7616	0.0095	475 - 487	1	R.ITSELSEQFAKSK.E
1475.7529	1474.7456	1474.7701	-0.0245	97 - 109	1	K.AMIAIEKENESLK.G
1706.8966	1705.8893	1705.8675	0.0218	2 - 16	1	M.ATIGFEEKLWQAADK.L
1722.8620	1721.8548	1721.8664	-0.0116	151 - 165	1	R.VYEYFIAKFASAEGK.N
1723.9219	1722.9146	1722.7559	0.1587	422 - 436	1	R.GTNDKYYEDIAGFCK.V

No match to: 781.4409, 832.3686, 852.4759, 855.0295, 861.0514, 873.4259, 877.0207, 893.0028, 1012.5758, 1157.6034, 1158.5981, 1179.5649, 1226.5318, 1230.6543, 1235.5913, 1238.6094, 1242.5535, 1258.5465, 1277.6779, 1283.5580, 1299.5558, 1301.6318, 1307.6958, 1308.6351, 1316.5630, 1317.6197, 1318.6086, 1323.6033, 1380.6922, 1386.6862, 1400.6222, 1462.8200, 1612.7497, 1638.8350, 1791.7119, 1815.8328, 1993.9556, 2202.0786, 2383.9341

29. AAM79212 **Mass:** 22460 **Score:** 55 **Expect:** 11 **Queries matched:** 8

AE014074 NID: - Streptococcus pyogenes MGAS315

Observed	Mr(expt)	Mr(calc)	Delta	Start - End	Miss	Peptide
1157.6034	1156.5962	1156.5434	0.0528	101 - 109	0	K.MIEEYLTSR.D + Oxidation (M)
1158.5981	1157.5908	1157.6516	-0.0608	54 - 64	1	K.NLARTSSKPGK.T
1235.5913	1234.5840	1234.7033	-0.1193	43 - 53	1	K.SSFINTILGRK.N
1301.6318	1300.6245	1300.6009	0.0236	128 - 137	0	K.EDIQMYDFLK.Y
1317.6197	1316.6124	1316.5958	0.0166	128 - 137	0	K.EDIQMYDFLK.Y + Oxidation (M)
1380.6922	1379.6849	1379.7700	-0.0851	138 - 149	0	K.YYDIPVIVVATK.A
1386.6862	1385.6789	1385.6826	-0.0037	174 - 185	0	K.SDTFIVFSSVER.I
1467.7784	1466.7712	1466.7405	0.0306	65 - 76	0	K.TQLLNFFNIDDK.L

No match to: 781.4409, 831.3855, 832.3686, 852.4759, 855.0295, 861.0514, 873.4259, 877.0207, 893.0028, 1012.5758, 1179.5649, 1213.6598, 1226.5318, 1230.6543, 1238.6094, 1240.5125, 1242.5535, 1252.6284, 1258.5465, 1277.6779, 1283.5580, 1299.5558, 1307.6958, 1308.6351, 1316.5630, 1318.6086, 1323.6033, 1400.6222, 1408.7039, 1462.8200, 1475.7529, 1612.7497, 1638.8350, 1706.8966, 1722.8620, 1723.9219, 1791.7119, 1815.8328, 1993.9556, 2202.0786, 2383.9341

30. AAK33806 **Mass:** 22479 **Score:** 54 **Expect:** 12 **Queries matched:** 8

AE006538 NID: - Streptococcus pyogenes M1 GAS

Observed	Mr(expt)	Mr(calc)	Delta	Start - End	Miss	Peptide
1157.6034	1156.5962	1156.5434	0.0528	101 - 109	0	K.MIEEYLTSR.D + Oxidation (M)
1158.5981	1157.5908	1157.6516	-0.0608	54 - 64	1	K.NLARTSSKPGK.T

1235.5913	1234.5840	1234.7033	-0.1193	43 - 53	1	K.SSFINTILGRK.N
1301.6318	1300.6245	1300.6009	0.0236	128 - 137	0	K.EDIQMYDFLK.Y
1317.6197	1316.6124	1316.5958	0.0166	128 - 137	0	K.EDIQMYDFLK.Y + Oxidation (M)
1380.6922	1379.6849	1379.7700	-0.0851	138 - 149	0	K.YYDIPVIVVATK.A
1386.6862	1385.6789	1385.6826	-0.0037	174 - 185	0	K.SDTFIVFSSVER.I
1467.7784	1466.7712	1466.7405	0.0306	65 - 76	0	K.TQLLNFFNIDDK.L

No match to: 781.4409, 831.3855, 832.3686, 852.4759, 855.0295, 861.0514, 873.4259, 877.0207, 893.0028, 1012.5758, 1179.5649, 1213.6598, 1226.5318, 1230.6543, 1238.6094, 1240.5125, 1242.5535, 1252.6284, 1258.5465, 1277.6779, 1283.5580, 1299.5558, 1307.6958, 1308.6351, 1316.5630, 1318.6086, 1323.6033, 1400.6222, 1408.7039, 1462.8200, 1475.7529, 1612.7497, 1638.8350, 1706.8966, 1722.8620, 1723.9219, 1791.7119, 1815.8328, 1993.9556, 2202.0786, 2383.9341

31. Q4WN94_ASPFU **Mass:** 42187 **Score:** 54 **Expect:** 14 **Queries matched:** 9

Actin binding protein, putative.- Aspergillus fumigatus (Sartorya fumigata).

Observed	Mr(expt)	Mr(calc)	Delta	Start - End	Miss	Peptide
832.3686	831.3613	831.4166	-0.0553	283 - 288	0	R.VYFFEK.E
1226.5318	1225.5245	1225.5840	-0.0594	37 - 46	1	K.RTDPYQFGSR.Y
1242.5535	1241.5462	1241.5789	-0.0326	107 - 116	1	K.NNTSNFFKDR.K
1252.6284	1251.6211	1251.5230	0.0981	269 - 277	0	R.YMDENFYVR.G + Oxidation (M)
1307.6958	1306.6885	1306.6153	0.0732	82 - 92	1	R.ESPVSDFDKQR.F
1317.6197	1316.6124	1316.6095	0.0029	354 - 366	1	R.ETPAAVEAEKADS.-
1318.6086	1317.6013	1317.6353	-0.0340	278 - 288	1	R.GDGTRVYFFEK.E
1467.7784	1466.7712	1466.7346	0.0365	96 - 106	1	K.DPAKWWNLFYK.N
1722.8620	1721.8548	1721.7467	0.1081	269 - 282	1	R.YMDENFYVRGDGTR.V

No match to: 781.4409, 831.3855, 852.4759, 855.0295, 861.0514, 873.4259, 877.0207, 893.0028, 1012.5758, 1157.6034, 1158.5981, 1179.5649, 1213.6598, 1230.6543, 1235.5913, 1238.6094, 1240.5125, 1258.5465, 1277.6779, 1283.5580, 1299.5558, 1301.6318, 1308.6351, 1316.5630, 1323.6033, 1380.6922, 1386.6862, 1400.6222, 1408.7039, 1462.8200, 1475.7529, 1612.7497, 1638.8350, 1706.8966, 1723.9219, 1791.7119, 1815.8328, 1993.9556, 2202.0786, 2383.9341

32. Q2RK76_MOOTA **Mass:** 45037 **Score:** 54 **Expect:** 14 **Queries matched:** 10

UDP-N-acetylglucosamine 1-carboxyvinyltransferase.- Moorella thermoacetica (strain ATCC 39073).

Observed	Mr(expt)	Mr(calc)	Delta	Start - End	Miss	Peptide
832.3686	831.3613	831.4385	-0.0771	266 - 272	1	K.LREMGAR.I
852.4759	851.4686	851.4725	-0.0039	333 - 339	1	K.HAAELRR.L
1012.5758	1011.5686	1011.4695	0.0991	220 - 227	1	R.ELKGCDYK.I + Carbamidomethyl (C)
1179.5649	1178.5577	1178.5754	-0.0177	223 - 232	1	K.GCDYKIIPDR.I
1213.6598	1212.6525	1212.6574	-0.0049	404 - 414	1	K.RLGDLGADIQR.L
1252.6284	1251.6211	1251.5838	0.0372	129 - 140	0	K.GLMAMGAEVTEK.L + Oxidation (M)
1301.6318	1300.6245	1300.7173	-0.0927	47 - 58	0	R.LQDVSVMAAVIR.S
1317.6197	1316.6124	1316.7122	-0.0997	47 - 58	0	R.LQDVSVMAAVIR.S + Oxidation (M)
1815.8328	1814.8255	1814.9382	-0.1127	23 - 40	0	K.NAALPIMAATLLATGECR.L
2202.0786	2201.0713	2201.1262	-0.0549	188 - 206	1	R.EPEIVDLQNFLNAMGARIR.G + Oxidation (M)

No match to: 781.4409, 831.3855, 855.0295, 861.0514, 873.4259, 877.0207, 893.0028, 1157.6034, 1158.5981, 1226.5318, 1230.6543, 1235.5913, 1238.6094, 1240.5125, 1242.5535, 1258.5465, 1277.6779, 1283.5580, 1299.5558, 1307.6958, 1308.6351, 1316.5630, 1318.6086, 1323.6033, 1380.6922, 1386.6862, 1400.6222, 1408.7039, 1462.8200, 1467.7784, 1475.7529, 1612.7497, 1638.8350, 1706.8966, 1722.8620, 1723.9219, 1791.7119, 1993.9556, 2383.9341

33. Q8GC31_LEUCI **Mass:** 47411 **Score:** 53 **Expect:** 16 **Queries matched:** 9

Putative mobilization protein.- Leuconostoc citreum.

Observed	Mr(expt)	Mr(calc)	Delta	Start - End	Miss	Peptide
852.4759	851.4686	851.3886	0.0800	23 - 29	0	K.TDHHTNK.E
1157.6034	1156.5962	1156.5369	0.0593	57 - 65	1	R.LNDVYCMKR.D + Oxidation (M)
1226.5318	1225.5245	1225.6302	-0.1057	349 - 358	1	K.LDHEELTKNK.K
1240.5125	1239.5052	1239.6247	-0.1195	191 - 200	1	K.KYNDQFNALK.N
1252.6284	1251.6211	1251.6723	-0.0512	10 - 21	0	R.GAVPGLAVHFER.K

1258.5465	1257.5392	1257.6201	-0.0809	373	383	1	R.EDPDTKISPTR.L
1299.5558	1298.5485	1298.5383	0.0102	314	323	0	R.EQQQMDYAMR.D
1301.6318	1300.6245	1300.6259	-0.0013	201	211	0	K.NELADVEENIR.A
1380.6922	1379.6849	1379.7673	-0.0824	10	22	1	R.GAVPGLAVHFERK.T

No match to: 781.4409, 831.3855, 832.3686, 855.0295, 861.0514, 873.4259, 877.0207, 893.0028, 1012.5758, 1158.5981, 1179.5649, 1213.6598, 1230.6543, 1235.5913, 1238.6094, 1242.5535, 1277.6779, 1283.5580, 1307.6958, 1308.6351, 1316.5630, 1317.6197, 1318.6086, 1323.6033, 1386.6862, 1400.6222, 1408.7039, 1462.8200, 1467.7784, 1475.7529, 1612.7497, 1638.8350, 1706.8966, 1722.8620, 1723.9219, 1791.7119, 1815.8328, 1993.9556, 2202.0786, 2383.9341

34. Q16V40_AEDAE **Mass:** 22771 **Score:** 53 **Expect:** 16 **Queries matched:** 8

Hypothetical protein.- Aedes aegypti (Yellowfever mosquito).

Observed	Mr(expt)	Mr(calc)	Delta	Start	End	Miss	Peptide
852.4759	851.4686	851.5229	-0.0542	83	90	0	R.ALTLIHGK.K
1158.5981	1157.5908	1157.5387	0.0522	27	35	1	R.TDMYRESIK.H + Oxidation (M)
1230.6543	1229.6470	1229.6186	0.0284	2	13	1	M.STTPPCNVRGAK.R
1283.5580	1282.5508	1282.6669	-0.1162	15	25	1	R.NVKSSYFSVPR.R
1307.6958	1306.6885	1306.6485	0.0399	41	52	1	R.NLCATRNIAMGK.A + Oxidation (M)
1612.7497	1611.7424	1611.7650	-0.0225	53	65	1	K.ACWNLNCRFTLSK.E + Carbamidomethyl (C)
1723.9219	1722.9146	1722.7752	0.1394	47	60	1	R.NIAMGKACWNLNCR.F + 2 Carbamidomethyl (C); Oxidation (M)
2383.9341	2382.9268	2383.1492	-0.2224	113	132	1	K.FAVVYKIFPGVNFCTCESFR.Y + Carbamidomethyl (C)

No match to: 781.4409, 831.3855, 832.3686, 855.0295, 861.0514, 873.4259, 877.0207, 893.0028, 1012.5758, 1157.6034, 1179.5649, 1213.6598, 1226.5318, 1235.5913, 1238.6094, 1240.5125, 1242.5535, 1252.6284, 1258.5465, 1277.6779, 1299.5558, 1301.6318, 1308.6351, 1316.5630, 1317.6197, 1318.6086, 1323.6033, 1380.6922, 1386.6862, 1400.6222, 1408.7039, 1462.8200, 1467.7784, 1475.7529, 1638.8350, 1706.8966, 1722.8620, 1791.7119, 1815.8328, 1993.9556, 2202.0786

35. Q1EPB8_MUSAC **Mass:** 14866 **Score:** 53 **Expect:** 16 **Queries matched:** 6

Hypothetical protein.- Musa acuminata (Banana).

Observed	Mr(expt)	Mr(calc)	Delta	Start	End	Miss	Peptide
852.4759	851.4686	851.4072	0.0614	17	23	1	R.QRFCGNK.S
1012.5758	1011.5686	1011.4906	0.0779	24	32	1	K.SEKSMSLSK.T + Oxidation (M)
1283.5580	1282.5508	1282.5538	-0.0030	8	18	1	R.EFDGSSDSRQR.F
1475.7529	1474.7456	1474.6841	0.0615	121	133	0	R.WHPTEAQSSSFAK.W
1638.8350	1637.8278	1637.8009	0.0269	2	16	1	M.ITLGVREFDGSSDSR.Q
1723.9219	1722.9146	1722.9199	-0.0052	93	108	1	R.CNRLSQAVAPPELGLR.A

No match to: 781.4409, 831.3855, 832.3686, 855.0295, 861.0514, 873.4259, 877.0207, 893.0028, 1157.6034, 1158.5981, 1179.5649, 1213.6598, 1226.5318, 1230.6543, 1235.5913, 1238.6094, 1240.5125, 1242.5535, 1252.6284, 1258.5465, 1277.6779, 1299.5558, 1301.6318, 1307.6958, 1308.6351, 1316.5630, 1317.6197, 1318.6086, 1323.6033, 1380.6922, 1386.6862, 1400.6222, 1408.7039, 1462.8200, 1467.7784, 1612.7497, 1706.8966, 1722.8620, 1791.7119, 1815.8328, 1993.9556, 2202.0786, 2383.9341

36. Q3WCG3_9ACTO **Mass:** 35265 **Score:** 53 **Expect:** 18 **Queries matched:** 8

Pyruvate dehydrogenase (Lipoamide) (EC 1.2.4.1).- Frankia sp. EAN1pec.

Observed	Mr(expt)	Mr(calc)	Delta	Start	End	Miss	Peptide
852.4759	851.4686	851.4865	-0.0179	73	80	0	R.GLHDLIGK.G
1157.6034	1156.5962	1156.5872	0.0090	17	25	0	R.LYELMTLMK.A + Oxidation (M)
1230.6543	1229.6470	1229.6629	-0.0158	6	16	1	R.TAGISGQVWRR.L
1240.5125	1239.5052	1239.6281	-0.1229	263	274	0	R.MAAAVEADPIPR.F
1277.6779	1276.6706	1276.5506	0.1200	322	332	1	R.DVYADPRNCPA.- + Carbamidomethyl (C)
1462.8200	1461.8127	1461.7068	0.1060	49	62	0	R.GQEAIAAAMGVCLR.S + Carbamidomethyl (C); Oxidation (M)
1706.8966	1705.8893	1705.8569	0.0324	230	245	1	R.ARSGGGPTLVECVTFR.F + Carbamidomethyl (C)
1723.9219	1722.9146	1722.7824	0.1323	248	262	0	R.GHYFGDPMAYIPAER.M

No match to: 781.4409, 831.3855, 832.3686, 855.0295, 861.0514, 873.4259, 877.0207, 893.0028, 1012.5758, 1158.5981, 1179.5649, 1213.6598, 1226.5318, 1235.5913, 1238.6094, 1242.5535, 1252.6284, 1258.5465, 1283.5580, 1299.5558, 1301.6318, 1307.6958, 1308.6351, 1316.5630, 1317.6197, 1318.6086, 1323.6033, 1380.6922, 1386.6862, 1400.6222, 1408.7039, 1467.7784, 1475.7529, 1612.7497, 1638.8350, 1722.8620, 1791.7119, 1815.8328, 1993.9556, 2202.0786, 2383.9341

37. Q1RUA7_MEDTR **Mass:** 7463 **Score:** 53 **Expect:** 18 **Queries matched:** 5

Hypothetical protein.- Medicago truncatula (Barrel medic).

Observed	Mr(expt)	Mr(calc)	Delta	Start	End	Miss	Peptide
852.4759	851.4686	851.4211	0.0475	12 -	18	0	K.LPTCQYK.C
873.4259	872.4186	872.4286	-0.0100	19 -	26	0	K.CGSTPRPR.H
1252.6284	1251.6211	1251.6897	-0.0686	1 -	11	0	-.MLDIPPLNVPK.L + Oxidation (M)
1318.6086	1317.6013	1317.6888	-0.0875	47 -	58	0	R.QTVSATSELLNR.C
1706.8966	1705.8893	1705.8392	0.0501	12 -	26	1	K.LPTCQYKCGSTPRPR.H

No match to: 781.4409, 831.3855, 832.3686, 855.0295, 861.0514, 877.0207, 893.0028, 1012.5758, 1157.6034, 1158.5981, 1179.5649, 1213.6598, 1226.5318, 1230.6543, 1235.5913, 1238.6094, 1240.5125, 1242.5535, 1258.5465, 1277.6779, 1283.5580, 1299.5558, 1301.6318, 1307.6958, 1308.6351, 1316.5630, 1317.6197, 1323.6033, 1380.6922, 1386.6862, 1400.6222, 1408.7039, 1462.8200, 1467.7784, 1475.7529, 1612.7497, 1638.8350, 1722.8620, 1723.9219, 1791.7119, 1815.8328, 1993.9556, 2202.0786, 2383.9341

38. Q9C902_ARATH **Mass:** 86001 **Score:** 52 **Expect:** 18 **Queries matched:** 12

Protein kinase, putative; 19229-23534 (Hypothetical protein At3g06620).- Arabidopsis thaliana (Mouse-ear cress).

Observed	Mr(expt)	Mr(calc)	Delta	Start	End	Miss	Peptide
852.4759	851.4686	851.4905	-0.0219	354 -	360	0	K.FVSKPFK.D
873.4259	872.4186	872.4545	-0.0358	388 -	394	0	K.GLSWPWK.G
1157.6034	1156.5962	1156.5699	0.0263	753 -	761	0	K.YMIQFQATR.A
1230.6543	1229.6470	1229.6377	0.0093	40 -	50	0	R.SHSVSPHRPAR.R
1235.5913	1234.5840	1234.6240	-0.0400	157 -	167	1	R.DAAFAMNIARR.C
1238.6094	1237.6021	1237.6165	-0.0144	125 -	134	0	R.IIFWNAMAEK.V + Oxidation (M)
1242.5535	1241.5462	1241.6265	-0.0802	51 -	61	1	R.RNIGEGAPSWR.K
1301.6318	1300.6245	1300.6598	-0.0352	752 -	761	1	R.KYMIQFQATR.A + Oxidation (M)
1386.6862	1385.6789	1385.7388	-0.0599	40 -	51	1	R.SHSVSPHRPARR.N
1400.6222	1399.6149	1399.6918	-0.0769	654 -	665	0	K.GTPQWMAPEVLR.N + Oxidation (M)
1467.7784	1466.7712	1466.7616	0.0095	13 -	24	0	K.ILELEESQEHLK.Q
1722.8620	1721.8548	1721.7541	0.1006	721 -	734	0	R.WISLMESCWHSDTK.L

No match to: 781.4409, 831.3855, 832.3686, 855.0295, 861.0514, 877.0207, 893.0028, 1012.5758, 1158.5981, 1179.5649, 1213.6598, 1226.5318, 1240.5125, 1252.6284, 1258.5465, 1277.6779, 1283.5580, 1299.5558, 1307.6958, 1308.6351, 1316.5630, 1317.6197, 1318.6086, 1323.6033, 1380.6922, 1408.7039, 1462.8200, 1475.7529, 1612.7497, 1638.8350, 1706.8966, 1723.9219, 1791.7119, 1815.8328, 1993.9556, 2202.0786, 2383.9341

39. Q9TSS5_BOSIN **Mass:** 10093 **Score:** 52 **Expect:** 19 **Queries matched:** 5

BoLA-DRB3 protein (Fragment).- Bos indicus (Zebu).

Observed	Mr(expt)	Mr(calc)	Delta	Start	End	Miss	Peptide
1158.5981	1157.5908	1157.4778	0.1131	32 -	40	0	R.FDSDWGEFR.A
1299.5558	1298.5485	1298.5931	-0.0446	73 -	83	0	R.HNYGVFESFTV.-
1638.8350	1637.8278	1637.7368	0.0910	5 -	17	1	K.SECHFFNGTERVR.Y + Carbamidomethyl (C)
1791.7119	1790.7047	1790.8223	-0.1177	18 -	31	1	R.YLDRYYTNGEENVR.F
2383.9341	2382.9268	2383.0141	-0.0873	22 -	40	1	R.YYTNGEENVRFDSDWGEFR.A

No match to: 781.4409, 831.3855, 832.3686, 852.4759, 855.0295, 861.0514, 873.4259, 877.0207, 893.0028, 1012.5758, 1157.6034, 1179.5649, 1213.6598, 1226.5318, 1230.6543, 1235.5913, 1238.6094, 1240.5125, 1242.5535, 1252.6284, 1258.5465, 1277.6779, 1283.5580, 1301.6318, 1307.6958, 1308.6351, 1316.5630, 1317.6197, 1318.6086, 1323.6033, 1380.6922, 1386.6862, 1400.6222, 1408.7039, 1462.8200, 1467.7784, 1475.7529, 1612.7497, 1706.8966, 1722.8620, 1723.9219, 1815.8328, 1993.9556, 2202.0786

40. Q5VU61_HUMAN **Mass:** 26265 **Score:** 52 **Expect:** 21 **Queries matched:** 10

Tropomyosin 3.- Homo sapiens (Human).

Observed	Mr(expt)	Mr(calc)	Delta	Start	End	Miss	Peptide
832.3686	831.3613	831.4338	-0.0724	74 -	80	1	R.ALKDEEK.M
1157.6034	1156.5962	1156.6564	-0.0602	43 -	52	1	R.EQRLATALQK.L
1158.5981	1157.5908	1157.6768	-0.0860	2 -	12	1	M.AGITTIEAVKR.K
1179.5649	1178.5577	1178.5172	0.0405	130 -	138	1	R.CREMDEQIR.L

```
1252.6284  1251.6211  1251.5336   0.0875   130 - 138   1  R.CREMDEQIR.L + Carbamidomethyl (C); Oxidation (M)
1308.6351  1307.6279  1307.6105   0.0173   174 - 184   1  K.EAETRAEFAER.S
1318.6086  1317.6013  1317.6664  -0.0650   189 - 199   1  K.LEKTIDDLEDK.L
1380.6922  1379.6849  1379.7184  -0.0335   161 - 171   1  K.YEEEIKILTDK.L
1467.7784  1466.7712  1466.6633   0.1079   213 - 224   0  R.MLDQTLLDLNEM.- + 2 Oxidation (M)
1475.7529  1474.7456  1474.7701  -0.0245    81 -  92   1  K.MELQEIQLKEAK.H + Oxidation (M)
```

No match to: 781.4409, 831.3855, 852.4759, 855.0295, 861.0514, 873.4259, 877.0207, 893.0028, 1012.5758, 1213.6598, 1226.5318, 1230.6543, 1235.5913, 1238.6094, 1240.5125, 1242.5535, 1258.5465, 1277.6779, 1283.5580, 1299.5558, 1301.6318, 1307.6958, 1316.5630, 1317.6197, 1323.6033, 1386.6862, 1400.6222, 1408.7039, 1462.8200, 1612.7497, 1638.8350, 1706.8966, 1722.8620, 1723.9219, 1791.7119, 1815.8328, 1993.9556, 2202.0786, 2383.9341

41. S11390 **Mass:** 29003 **Score:** 52 **Expect:** 21 **Queries matched:** 10

tropomyosin 5 - mouse

Observed	Mr(expt)	Mr(calc)	Delta	Start	End	Miss	Peptide
832.3686	831.3613	831.4338	-0.0724	98	104	1	R.ALKDEEK.M
1179.5649	1178.5577	1178.5172	0.0405	154	162	1	R.CREMDEQIR.L
1252.6284	1251.6211	1251.5336	0.0875	154	162	1	R.CREMDEQIR.L + Carbamidomethyl (C); Oxidation (M)
1277.6779	1276.6706	1276.6809	-0.0103	1	12	1	-.MAGTTTIEAVKR.K
1308.6351	1307.6279	1307.6105	0.0173	198	208	1	K.EAETRAEFAER.S
1316.5630	1315.5558	1315.6367	-0.0810	43	54	0	R.EQAEAEVASLNR.R
1318.6086	1317.6013	1317.6664	-0.0650	213	223	1	K.LEKTIDDLEDK.L
1380.6922	1379.6849	1379.7184	-0.0335	185	195	1	K.YEEEIKILTDK.L
1467.7784	1466.7712	1466.6633	0.1079	237	248	0	R.MLDQTLLDLNEM.- + 2 Oxidation (M)
1475.7529	1474.7456	1474.7701	-0.0245	105	116	1	K.MELQEIQLKEAK.H + Oxidation (M)

No match to: 781.4409, 831.3855, 852.4759, 855.0295, 861.0514, 873.4259, 877.0207, 893.0028, 1012.5758, 1157.6034, 1158.5981, 1213.6598, 1226.5318, 1230.6543, 1235.5913, 1238.6094, 1240.5125, 1242.5535, 1258.5465, 1283.5580, 1299.5558, 1301.6318, 1307.6958, 1317.6197, 1323.6033, 1386.6862, 1400.6222, 1408.7039, 1462.8200, 1612.7497, 1638.8350, 1706.8966, 1722.8620, 1723.9219, 1791.7119, 1815.8328, 1993.9556, 2202.0786, 2383.9341

42. Q54L29_DICDI **Mass:** 11417 **Score:** 52 **Expect:** 22 **Queries matched:** 6

Hypothetical protein.- Dictyostelium discoideum AX4.

Observed	Mr(expt)	Mr(calc)	Delta	Start	End	Miss	Peptide
832.3686	831.3613	831.3796	-0.0183	2	8	0	M.EENCLPK.K
1157.6034	1156.5962	1156.6968	-0.1006	22	31	1	R.LIKFPPSLSR.L
1277.6779	1276.6706	1276.5506	0.1200	12	21	1	K.RFEMSDSYSR.L
1301.6318	1300.6245	1300.7503	-0.1257	25	36	1	K.FPPSLSRLSIGK.E
1307.6958	1306.6885	1306.6306	0.0579	64	74	1	K.HFDGNFKDLSK.S
1475.7529	1474.7456	1474.7126	0.0330	13	24	1	R.FEMSDSYSRLIK.F

No match to: 781.4409, 831.3855, 852.4759, 855.0295, 861.0514, 873.4259, 877.0207, 893.0028, 1012.5758, 1158.5981, 1179.5649, 1213.6598, 1226.5318, 1230.6543, 1235.5913, 1238.6094, 1240.5125, 1242.5535, 1252.6284, 1258.5465, 1283.5580, 1299.5558, 1308.6351, 1316.5630, 1317.6197, 1318.6086, 1323.6033, 1380.6922, 1386.6862, 1400.6222, 1408.7039, 1462.8200, 1467.7784, 1612.7497, 1638.8350, 1706.8966, 1722.8620, 1723.9219, 1791.7119, 1815.8328, 1993.9556, 2202.0786, 2383.9341

43. Q5ZUP2_LEGPH **Mass:** 50366 **Score:** 51 **Expect:** 23 **Queries matched:** 9

Hypothetical protein.- Legionella pneumophila subsp. pneumophila (strain Philadelphia 1 / ATCC 33152 / DSM 7513).

Observed	Mr(expt)	Mr(calc)	Delta	Start	End	Miss	Peptide
832.3686	831.3613	831.3909	-0.0295	191	197	1	K.DEGKMPR.Y
1238.6094	1237.6021	1237.6666	-0.0645	251	261	0	R.QIEEALTHLGK.F
1240.5125	1239.5052	1239.5778	-0.0726	322	331	1	K.NCNSSLARYR.A + Carbamidomethyl (C)
1242.5535	1241.5462	1241.6114	-0.0652	262	271	1	K.FLPKTCSDFK.T + Carbamidomethyl (C)
1258.5465	1257.5392	1257.6564	-0.1172	284	294	0	K.AQIESNVLQEK.I
1323.6033	1322.5960	1322.7234	-0.1274	377	387	1	R.LLKTYFEANPK.V
1462.8200	1461.8127	1461.7133	0.0994	182	194	1	R.DNMQLIVGKDEGK.M + Oxidation (M)
1706.8966	1705.8893	1706.0355	-0.1462	94	108	1	K.NKQAILFLLGALLHR.Y

1723.9219 1722.9146 1722.9668 -0.0522 251 - 265 1 R.QIEEALTHLGKFLPK.T

No match to: 781.4409, 831.3855, 852.4759, 855.0295, 861.0514, 873.4259, 877.0207, 893.0028, 1012.5758, 1157.6034, 1158.5981, 1179.5649, 1213.6598, 1226.5318, 1230.6543, 1235.5913, 1252.6284, 1277.6779, 1283.5580, 1299.5558, 1301.6318, 1307.6958, 1308.6351, 1316.5630, 1317.6197, 1318.6086, 1380.6922, 1386.6862, 1400.6222, 1408.7039, 1467.7784, 1475.7529, 1612.7497, 1638.8350, 1722.8620, 1791.7119, 1815.8328, 1993.9556, 2202.0786, 2383.9341

44. A25530 **Mass:** 29015 **Score:** 51 **Expect:** 23 **Queries matched:** 10

tropomyosin, fibroblast - human

Observed	Mr(expt)	Mr(calc)	Delta	Start	End	Miss	Peptide
832.3686	831.3613	831.4338	-0.0724	98 -	104	1	R.ALKDEEK.M
1158.5981	1157.5908	1157.6768	-0.0860	2 -	12	1	M.AGITTIEAVKR.K
1179.5649	1178.5577	1178.5172	0.0405	154 -	162	1	R.CREMDEQIR.L
1252.6284	1251.6211	1251.5336	0.0875	154 -	162	1	R.CREMDEQIR.L + Carbamidomethyl (C); Oxidation (M)
1308.6351	1307.6279	1307.6105	0.0173	198 -	208	1	K.EAETRAEFAER.S
1316.5630	1315.5558	1315.6367	-0.0810	43 -	54	0	R.EQAEAEVASLNR.R
1318.6086	1317.6013	1317.6664	-0.0650	213 -	223	1	K.LEKTIDDLEDK.L
1380.6922	1379.6849	1379.7184	-0.0335	185 -	195	1	K.YEEEIKILTDK.L
1467.7784	1466.7712	1466.6633	0.1079	237 -	248	0	R.MLDQTLLDLNEM.- + 2 Oxidation (M)
1475.7529	1474.7456	1474.7701	-0.0245	105 -	116	1	K.MELQEIQLKEAK.H + Oxidation (M)

No match to: 781.4409, 831.3855, 852.4759, 855.0295, 861.0514, 873.4259, 877.0207, 893.0028, 1012.5758, 1157.6034, 1213.6598, 1226.5318, 1230.6543, 1235.5913, 1238.6094, 1240.5125, 1242.5535, 1258.5465, 1277.6779, 1283.5580, 1299.5558, 1301.6318, 1307.6958, 1317.6197, 1323.6033, 1386.6862, 1400.6222, 1408.7039, 1462.8200, 1612.7497, 1638.8350, 1706.8966, 1722.8620, 1723.9219, 1791.7119, 1815.8328, 1993.9556, 2202.0786, 2383.9341

45. Q4HE11_CAMCO **Mass:** 31370 **Score:** 51 **Expect:** 23 **Queries matched:** 8

Hypothetical protein.- Campylobacter coli RM2228.

Observed	Mr(expt)	Mr(calc)	Delta	Start	End	Miss	Peptide
831.3855	830.3783	830.3956	-0.0173	93 -	99	0	R.EVMHTSK.K
1158.5981	1157.5908	1157.5716	0.0192	84 -	92	1	K.YKEELSSFR.E
1242.5535	1241.5462	1241.6444	-0.0982	212 -	222	0	K.FIFGSNYPVAK.I
1283.5580	1282.5508	1282.6193	-0.0685	104 -	113	1	K.RLFEADFEEK.I
1316.5630	1315.5558	1315.5940	-0.0383	119 -	129	0	K.TFNIPFEACMK.N + Oxidation (M)
1318.6086	1317.6013	1317.6751	-0.0738	17 -	27	1	K.MPISWLKGNEK.L + Oxidation (M)
1722.8620	1721.8548	1721.9504	-0.0956	212 -	227	1	K.FIFGSNYPVAKIAPAK.W
1723.9219	1722.9146	1722.9079	0.0067	105 -	118	1	R.LFEADFEEKIEILK.T

No match to: 781.4409, 832.3686, 852.4759, 855.0295, 861.0514, 873.4259, 877.0207, 893.0028, 1012.5758, 1157.6034, 1179.5649, 1213.6598, 1226.5318, 1230.6543, 1235.5913, 1238.6094, 1240.5125, 1252.6284, 1258.5465, 1277.6779, 1299.5558, 1301.6318, 1307.6958, 1308.6351, 1317.6197, 1323.6033, 1380.6922, 1386.6862, 1400.6222, 1408.7039, 1462.8200, 1467.7784, 1475.7529, 1612.7497, 1638.8350, 1706.8966, 1791.7119, 1815.8328, 1993.9556, 2202.0786, 2383.9341

46. Q8XVL4_RALSO **Mass:** 63203 **Score:** 51 **Expect:** 23 **Queries matched:** 10

Probable prolyl-trna synthetase protein (EC 6.1.1.15).- Ralstonia solanacearum (Pseudomonas solanacearum).

Observed	Mr(expt)	Mr(calc)	Delta	Start	End	Miss	Peptide
852.4759	851.4686	851.4435	0.0251	147 -	153	1	R.FGIMRGR.E + Oxidation (M)
873.4259	872.4186	872.3664	0.0522	159 -	165	0	K.DAYSFDR.D
1213.6598	1212.6525	1212.5622	0.0903	391 -	402	0	R.NVVEGDPSPDGK.G
1238.6094	1237.6021	1237.6190	-0.0169	166 -	176	1	R.DAEGLKVSYEK.M
1400.6222	1399.6149	1399.7533	-0.1384	1 -	12	1	-.MKASQFFISTLK.E
1467.7784	1466.7712	1466.6863	0.0848	172 -	183	1	K.VSYEKMYGAYTR.I
1475.7529	1474.7456	1474.7820	-0.0364	330 -	344	0	R.AFGTPPGYLGPIGTK.Q
1638.8350	1637.8278	1637.8963	-0.0685	34 -	48	1	K.KLGAGLYTYMPVGLR.V
1706.8966	1705.8893	1705.7296	0.1597	438 -	452	0	K.TQPMMMGCYGIGITR.I + 3 Oxidation (M)
1815.8328	1814.8255	1814.9137	-0.0882	127 -	140	1	K.QMPVNFYQIQTKFR.D + Oxidation (M)

No match to: 781.4409, 831.3855, 832.3686, 855.0295, 861.0514, 877.0207, 893.0028, 1012.5758, 1157.6034, 1158.5981, 1179.5649, 1226.5318, 1230.6543, 1235.5913, 1240.5125, 1242.5535, 1252.6284, 1258.5465, 1277.6779, 1283.5580, 1299.5558, 1301.6318, 1307.6958, 1308.6351, 1316.5630, 1317.6197, 1318.6086, 1323.6033, 1380.6922, 1386.6862, 1408.7039, 1462.8200, 1612.7497, 1722.8620, 1723.9219, 1791.7119, 1993.9556, 2202.0786, 2383.9341

47. Q22LV9_TETTH **Mass:** 52292 **Score:** 51 **Expect:** 24 **Queries matched:** 9

Hypothetical protein.- Tetrahymena thermophila SB210.

Observed	Mr(expt)	Mr(calc)	Delta	Start	End	Miss	Peptide
1235.5913	1234.5840	1234.5836	0.0003	116 -	125	1	K.RMSQSQNQTR.V
1240.5125	1239.5052	1239.5771	-0.0719	322 -	331	1	R.VFPNKDYDDK.V
1299.5558	1298.5485	1298.6255	-0.0770	185 -	194	1	R.RDVDINYYNK.H
1308.6351	1307.6279	1307.6218	0.0061	356 -	366	0	K.NSSQIFNNTQR.E
1408.7039	1407.6966	1407.7510	-0.0544	9 -	20	1	K.QKVPQYLGNSFK.D
1467.7784	1466.7712	1466.7517	0.0194	394 -	406	0	R.LFGAEAQNYSLVR.A
1723.9219	1722.9146	1722.8325	0.0821	308 -	321	0	R.TNPQIEFLNNYSQR.V
1815.8328	1814.8255	1814.9202	-0.0947	419 -	433	1	R.NFNIISYADNKIEFK.K
2202.0786	2201.0713	2201.0065	0.0648	149 -	166	1	R.ESFFFTSVSRFVDDNNYK.M

No match to: 781.4409, 831.3855, 832.3686, 852.4759, 855.0295, 861.0514, 873.4259, 877.0207, 893.0028, 1012.5758, 1157.6034, 1158.5981, 1179.5649, 1213.6598, 1226.5318, 1230.6543, 1238.6094, 1242.5535, 1252.6284, 1258.5465, 1277.6779, 1283.5580, 1301.6318, 1307.6958, 1316.5630, 1317.6197, 1318.6086, 1323.6033, 1380.6922, 1386.6862, 1400.6222, 1462.8200, 1475.7529, 1612.7497, 1638.8350, 1706.8966, 1722.8620, 1791.7119, 1993.9556, 2383.9341

48. Q9Y355_HUMAN **Mass:** 7429 **Score:** 51 **Expect:** 26 **Queries matched:** 5

Apolipoprotein A1 (Fragment).- Homo sapiens (Human).

Observed	Mr(expt)	Mr(calc)	Delta	Start	End	Miss	Peptide
781.4409	780.4336	780.4242	0.0094	14 -	20	0	R.AHVDALR.T
831.3855	830.3783	830.4286	-0.0503	49 -	55	0	R.LAEYHAK.A
1157.6034	1156.5962	1156.6200	-0.0238	38 -	48	1	R.LEALKENGGAR.L
1301.6318	1300.6245	1300.6411	-0.0166	21 -	31	0	R.THLAPYSDELR.Q
1318.6086	1317.6013	1317.6347	-0.0334	1 -	11	1	-.LSPLGEEMRDR.A + Oxidation (M)

No match to: 832.3686, 852.4759, 855.0295, 861.0514, 873.4259, 877.0207, 893.0028, 1012.5758, 1158.5981, 1179.5649, 1213.6598, 1226.5318, 1230.6543, 1235.5913, 1238.6094, 1240.5125, 1242.5535, 1252.6284, 1258.5465, 1277.6779, 1283.5580, 1299.5558, 1307.6958, 1308.6351, 1316.5630, 1317.6197, 1323.6033, 1380.6922, 1386.6862, 1400.6222, 1408.7039, 1462.8200, 1467.7784, 1475.7529, 1612.7497, 1638.8350, 1706.8966, 1722.8620, 1723.9219, 1791.7119, 1815.8328, 1993.9556, 2202.0786, 2383.9341

49. T35704 **Mass:** 16760 **Score:** 51 **Expect:** 27 **Queries matched:** 6

peptidylprolyl isomerase (EC 5.2.1.8) SC7H1.09 [similarity] - Streptomyces coelicolor

Observed	Mr(expt)	Mr(calc)	Delta	Start	End	Miss	Peptide
832.3686	831.3613	831.4272	-0.0659	1 -	7	0	-.MAENVLR.G
873.4259	872.4186	872.4716	-0.0529	131 -	138	0	R.VTIAADQR.S
1158.5981	1157.5908	1157.6305	-0.0396	35 -	45	1	R.VPGFVVRGGDR.L
1252.6284	1251.6211	1251.5454	0.0756	23 -	33	0	R.GGFCDGTVFHR.R + Carbamidomethyl (C)
1386.6862	1385.6789	1385.7377	-0.0587	144 -	157	0	R.ALWPGGGTAMVTLI.-
1408.7039	1407.6966	1407.6465	0.0501	23 -	34	1	R.GGFCDGTVFHRR.V + Carbamidomethyl (C)

No match to: 781.4409, 831.3855, 852.4759, 855.0295, 861.0514, 877.0207, 893.0028, 1012.5758, 1157.6034, 1179.5649, 1213.6598, 1226.5318, 1230.6543, 1235.5913, 1238.6094, 1240.5125, 1242.5535, 1258.5465, 1277.6779, 1283.5580, 1299.5558, 1301.6318, 1307.6958, 1308.6351, 1316.5630, 1317.6197, 1318.6086, 1323.6033, 1380.6922, 1400.6222, 1462.8200, 1467.7784, 1475.7529, 1612.7497, 1638.8350, 1706.8966, 1722.8620, 1723.9219, 1791.7119, 1815.8328, 1993.9556, 2202.0786, 2383.9341

50. Q9KJG9_STRLI **Mass:** 16717 **Score:** 51 **Expect:** 27 **Queries matched:** 6

Peptidyl-prolyl cis-trans isomerase.- Streptomyces lividans.

Observed	Mr(expt)	Mr(calc)	Delta	Start	End	Miss	Peptide
832.3686	831.3613	831.4272	-0.0659	1 -	7	0	-.MAENVLR.G
873.4259	872.4186	872.4716	-0.0529	131 -	138	0	R.VTIAADQR.S

```
1158.5981  1157.5908  1157.6305  -0.0396    35 -  45   1  R.VPGFVVRGGDR.L
1252.6284  1251.6211  1251.5454   0.0756    23 -  33   0  R.GGFCDGTVFHR.R + Carbamidomethyl (C)
1386.6862  1385.6789  1385.7377  -0.0587   144 - 157   0  R.ALWPGGGTAMVTLI.-
1408.7039  1407.6966  1407.6465   0.0501    23 -  34   1  R.GGFCDGTVFHRR.V + Carbamidomethyl (C)
```

No match to: 781.4409, 831.3855, 852.4759, 855.0295, 861.0514, 877.0207, 893.0028, 1012.5758, 1157.6034, 1179.5649, 1213.6598, 1226.5318, 1230.6543, 1235.5913, 1238.6094, 1240.5125, 1242.5535, 1258.5465, 1277.6779, 1283.5580, 1299.5558, 1301.6318, 1307.6958, 1308.6351, 1316.5630, 1317.6197, 1318.6086, 1323.6033, 1380.6922, 1400.6222, 1462.8200, 1467.7784, 1475.7529, 1612.7497, 1638.8350, 1706.8966, 1722.8620, 1723.9219, 1791.7119, 1815.8328, 1993.9556, 2202.0786, 2383.9341

Search Parameters

```
Type of search         : Peptide Mass Fingerprint
Enzyme                 : Trypsin
Variable modifications : Carbamidomethyl (C),Oxidation (M)
Mass values            : Monoisotopic
Protein Mass           : Unrestricted
Peptide Mass Tolerance : ± 100 ppm
Peptide Charge State   : 1+
Max Missed Cleavages   : 1
Number of queries      : 49
```

Mascot: http://www.matrixscience.com/
