# Supplementary material for: A proteomic strategy to identify novel serum biomarkers for liver cirrhosis and hepatocellular cancer in individuals with fatty liver disease
Source: BMC Cancer. 2009 Aug 5;9:271. doi: 10.1186/1471-2407-9-271 (PMC2729079; doi:10.1186/1471-2407-9-271)
Supplement: Additional File 4 — Spot 2 is also ApoA1. The protein summary report for spot 2, generated using Mascot Peptide Mass Fingerprint search program (Matrix Science Ltd), identifies it as ApoA1. [file 1471-2407-9-271-S4.pdf]

Mascot Search Results

User : Joe Gray
Email : joe.gray@ncl.ac.uk
Search title : DIP_2_0001.dat - SpecView
Database : MSDB 20060831 (3239079 sequences; 1079594700 residues)
Timestamp : 8 Jan 2007 at 11:34:53 GMT
Top Score : 236 for CAA00975, APOA1 PROTEIN (FRAGMENT).- Homo sapiens (Human).

Probability Based Mowse Score

Protein score is -10*Log(P), where P is the probability that the observed match is a random event.
Protein scores greater than 78 are significant (p<0.05).

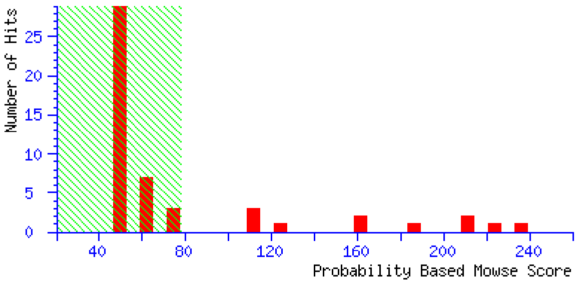

Protein Summary Report

Format As	Protein Summary	Help
	Significance threshold p< 0.05	Max. number of hits 50

Re-Search All | Search Unmatched

Index

	Accession	Mass	Score	Description
1.	CAA00975	28061	236	APOA1 PROTEIN (FRAGMENT).- Homo sapiens (Human).
2.	AAA51747	28944	218	HUMAPOAIC NID: - Homo sapiens
3.	LPHUA1	30759	208	apolipoprotein A-I precursor [validated] - human
4.	AAX42892	30872	207	AY890956 NID: - synthetic construct
5.	AAA35545	30745	192	HUMAPOAIP NID: - Homo sapiens
6.	CAA03490	23205	160	SEQUENCE 10 FROM PATENT WO9637608.- unidentified.
7.	1AV1A	23389	160	apolipoprotein a-i lipid-binding domain mutant N-TERMINAL MET, DEL(1-43), chain A - human
8.	Q8HZ97_PANTR	22075	130	Apolipoprotein A-I (Fragment).- Pan troglodytes (Chimpanzee).
9.	Q8HZ95_PONPY	22116	113	Apolipoprotein A-I (Fragment).- Pongo pygmaeus (Orangutan).
10.	A26529	30700	107	apolipoprotein A-I precursor - crab-eating macaque
11.	JS0079	30716	107	apolipoprotein A-I precursor - baboon
12.	CAD61352	18392	74	Sequence 2 from Patent WO02083898 precursor.- Homo sapiens (Human).
13.	Q31VN4_SHIBS	15504	69	Hypothetical protein yrfH.- Shigella boydii serotype 4 (strain Sb227).
14.	Q8ZLJ5_SALTY	15511	68	Heat shock protein.- Salmonella typhimurium.
15.	H86004	15486	61	ribosome-associated heat shock protein yrfH [similarity] - Escherichia coli (strain O157:H7, substrain EDL933)

16.	H97327	17433	59	transcription regulator, MarR/EmrR family [imported] - Clostridium acetobutylicum
17.	Q57IX7_SALCH	15557	59	Heat shock protein, predicted small RNA-binding protein.- Salmonella choleraesuis.
18.	Q5PLY1_SALPA	15465	58	Heat shock protein.- Salmonella paratyphi-a.
19.	Q7SD03_NEUCR	189062	57	Hypothetical protein NCU03060.1.- Neurospora crassa.
20.	Q5ZVU0_LEGPH	41536	56	L-lysine dehydrogenase.- Legionella pneumophila subsp. pneumophila (strain Philadelphia 1 / ATCC 33152 / DSM 7513).
21.	B72212	20862	56	translation elongation factor P - Thermotoga maritima (strain MSB8)
22.	Q19Y36_9CAUD	12275	55	Gp24.- Mycobacteriophage Wildcat.
23.	Q2WWQ7_9GAMM	12361	53	Hypothetical protein.- Shewanella sp. W3-18-1.
24.	Q2ZS56_SHEPU	12333	53	Hypothetical protein.- Shewanella putrefaciens CN-32.
25.	Q833F6_ENTFA	106075	53	Tape measure protein, putative.- Enterococcus faecalis (Streptococcus faecalis).
26.	AAL97589	22480	53	AE010023 NID: - Streptococcus pyogenes MGAS8232
27.	Q8I4X5_PLAF7	188045	53	Hypothetical protein.- Plasmodium falciparum (isolate 3D7).
28.	Q8K6Y4_STRP3	28197	53	Putative 1-acylglycerol-3-phosphate O-acyltransferase.- Streptococcus pyogenes serotype M3.
29.	Q84QB8_ORYSA	42907	52	Putative receptor ser/thr protein.- Oryza sativa (japonica cultivar-group).
30.	AAM79212	22460	52	AE014074 NID: - Streptococcus pyogenes MGAS315
31.	AAN72830	253967	52	AY157993 NID: - Tomato black ring virus
32.	AAK33806	22479	51	AE006538 NID: - Streptococcus pyogenes M1 GAS
33.	Q8T530_PLAFA	14687	51	Erythrocyte membrane protein 1 (Fragment).- Plasmodium falciparum.
34.	Q8GJA6_CAMJE	53327	51	Hypothetical protein Cjp49.- Campylobacter jejuni.
35.	Q2YVH6_STAAB	9035	51	Hypothetical protein.- Staphylococcus aureus (strain bovine RF122).
36.	Q9AH95_STRPN	24847	51	Wze (Tyrosine-protein kinase Wze) (EC 2.7.1.112).- Streptococcus pneumoniae.
37.	Q1RUA7_MEDTR	7463	51	Hypothetical protein.- Medicago truncatula (Barrel medic).
38.	Q54RC0_DICDI	45292	51	Hypothetical protein.- Dictyostelium discoideum AX4.
39.	Q8D787_VIBVU	11586	51	Hypothetical protein.- Vibrio vulnificus.
40.	O88180_MOUSE	65724	50	Guanine Nucleotide Regulatory Protein.- Mus musculus (Mouse).
41.	Q9FG41_ARATH	73235	50	Arabidopsis thaliana genomic DNA, chromosome 5, BAC clone:T25O11.- Arabidopsis thaliana (Mouse-ear cress).
42.	Q1IF78_9PSED	98036	50	Hypothetical protein.- Pseudomonas entomophila L48.
43.	Q4YWP1_PLABE	30166	50	Hypothetical protein (Fragment).- Plasmodium berghei.
44.	T35704	16760	50	peptidylprolyl isomerase (EC 5.2.1.8) SC7H1.09 [similarity] - Streptomyces coelicolor
45.	Q57U85_9TRYP	49924	50	Orotidine-5-phosphate decarboxylase/orotate phosphoribosyltransferase, putative (EC 2.4.2.10) (EC 4.1.1.23).- Trypanosoma brucei.
46.	Q4JCP4_SULAC	27674	49	Conserved protein.- Sulfolobus acidocaldarius.
47.	Q9Y355_HUMAN	7429	49	Apolipoprotein A1 (Fragment).- Homo sapiens (Human).
48.	C32055	17279	49	nifX protein - Azotobacter vinelandii
49.	Q5ZWH6_LEGPH	79435	49	Hypothetical protein.- Legionella pneumophila subsp. pneumophila (strain Philadelphia 1 / ATCC 33152 / DSM 7513).
50.	1DM9A	11873	49	hypothetical 15.5 kd protein in mrca-pcka intergenic region, chain A - bacteria

Results List

1. CAA00975 **Mass:** 28061 **Score:** 236 **Expect:** 8.1e-18 **Queries matched:** 22

APOA1 PROTEIN (FRAGMENT).- Homo sapiens (Human).

Observed	Mr(expt)	Mr(calc)	Delta	Start	End	Miss	Peptide
781.4592	780.4520	780.4242	0.0278	154 -	160	0	R.AHVDALR.T
831.4419	830.4346	830.4286	0.0060	189 -	195	0	R.LAEYHAK.A
873.4641	872.4568	872.4352	0.0217	124 -	131	0	R.AELQEGAR.Q
1012.6155	1011.6083	1011.5713	0.0370	207 -	215	0	K.AKPALEDLR.Q
1157.6270	1156.6198	1156.6200	-0.0002	178 -	188	1	R.LEALKENGGAR.L
1226.5773	1225.5700	1225.5364	0.0336	1 -	10	0	-.DEPPQSPWDR.V
1230.7044	1229.6971	1229.7019	-0.0048	216 -	226	0	R.QGLLPVLESFK.V
1235.6798	1234.6726	1234.6809	-0.0083	13 -	23	0	K.DLATVYVDVLK.D
1252.6795	1251.6722	1251.6135	0.0587	97 -	106	0	K.VQPYLDDFQK.K
1283.6177	1282.6105	1282.5652	0.0453	108 -	116	0	K.WQEEMELYR.Q
1299.6059	1298.5987	1298.5601	0.0386	108 -	116	0	K.WQEEMELYR.Q + Oxidation (M)
1301.6870	1300.6797	1300.6411	0.0385	161 -	171	0	R.THLAPYSDELR.Q
1318.6687	1317.6615	1317.6347	0.0268	141 -	151	1	K.LSPLGEEMRDR.A + Oxidation (M)
1380.7520	1379.7447	1379.7085	0.0362	97 -	107	1	K.VQPYLDDFQKK.W
1386.7403	1385.7330	1385.7078	0.0252	227 -	238	0	K.VSFLSALEEYTK.K

1400.6846	1399.6774	1399.6619	0.0155	28	- 40	0	R.DYVSQFEGSALGK.Q
1427.7243	1426.7170	1426.6551	0.0620	107	- 116	1	K.KWQEEMELYR.Q + Oxidation (M)
1462.8779	1461.8706	1461.8442	0.0264	11	- 23	1	R.VKDLATVYVDVLK.D
1467.8310	1466.8238	1466.7841	0.0397	119	- 131	1	K.VEPLRAELQEGAR.Q
1612.8208	1611.8135	1611.7780	0.0355	46	- 59	0	K.LLDNWDSVTSTFSK.L
1815.9168	1814.9096	1814.8434	0.0661	24	- 40	1	K.DSGRDYVSQFEGSALGK.Q
2202.1952	2201.1880	2201.1116	0.0763	60	- 77	1	K.LREQLGPVTQEFWDNLEK.E

No match to: 712.2679, 825.1370, 832.3588, 833.3959, 841.0980, 852.4849, 853.5010, 861.0837, 876.3626, 877.0567, 1158.6512, 1213.7102, 1224.6023, 1238.8028, 1240.5956, 1242.5818, 1257.6582, 1257.7440, 1258.5811, 1274.6199, 1282.5765, 1298.6028, 1315.6103, 1334.6603, 1402.7407, 1408.7587, 1422.7041, 1424.7200, 1690.6642, 1706.9716, 1723.9839

2. AAA51747 **Mass:** 28944 **Score:** **218** **Expect:** 5.1e-16 **Queries matched:** 21

HUMAPOAIC NID: - Homo sapiens

Observed	Mr(expt)	Mr(calc)	Delta	Start	End	Miss	Peptide
781.4592	780.4520	780.4242	0.0278	160	- 166	0	R.AHVDALR.T
831.4419	830.4346	830.4286	0.0060	195	- 201	0	R.LAEYHAK.A
873.4641	872.4568	872.4352	0.0217	130	- 137	0	R.AELQEGAR.Q
1012.6155	1011.6083	1011.5713	0.0370	213	- 221	0	K.AKPALEDLR.Q
1157.6270	1156.6198	1156.6200	-0.0002	184	- 194	1	R.LEALKENGGAR.L
1230.7044	1229.6971	1229.7019	-0.0048	222	- 232	0	R.QGLLPVLESFK.V
1235.6798	1234.6726	1234.6809	-0.0083	19	- 29	0	K.DLATVYVDVLK.D
1252.6795	1251.6722	1251.6135	0.0587	103	- 112	0	K.VQPYLDDFQK.K
1283.6177	1282.6105	1282.5652	0.0453	114	- 122	0	K.WQEEMELYR.Q
1299.6059	1298.5987	1298.5601	0.0386	114	- 122	0	K.WQEEMELYR.Q + Oxidation (M)
1301.6870	1300.6797	1300.6411	0.0385	167	- 177	0	R.THLAPYSDELR.Q
1318.6687	1317.6615	1317.6347	0.0268	147	- 157	1	K.LSPLGEEMRDR.A + Oxidation (M)
1380.7520	1379.7447	1379.7085	0.0362	103	- 113	1	K.VQPYLDDFQKK.W
1386.7403	1385.7330	1385.7078	0.0252	233	- 244	0	K.VSFLSALEEYTK.K
1400.6846	1399.6774	1399.6619	0.0155	34	- 46	0	R.DYVSQFEGSALGK.Q
1427.7243	1426.7170	1426.6551	0.0620	113	- 122	1	K.KWQEEMELYR.Q + Oxidation (M)
1462.8779	1461.8706	1461.8442	0.0264	17	- 29	1	R.VKDLATVYVDVLK.D
1467.8310	1466.8238	1466.7841	0.0397	125	- 137	1	K.VEPLRAELQEGAR.Q
1612.8208	1611.8135	1611.7780	0.0355	52	- 65	0	K.LLDNWDSVTSTFSK.L
1815.9168	1814.9096	1814.8434	0.0661	30	- 46	1	K.DSGRDYVSQFEGSALGK.Q
2202.1952	2201.1880	2201.1116	0.0763	66	- 83	1	K.LREQLGPVTQEFWDNLEK.E

No match to: 712.2679, 825.1370, 832.3588, 833.3959, 841.0980, 852.4849, 853.5010, 861.0837, 876.3626, 877.0567, 1158.6512, 1213.7102, 1224.6023, 1226.5773, 1238.8028, 1240.5956, 1242.5818, 1257.6582, 1257.7440, 1258.5811, 1274.6199, 1282.5765, 1298.6028, 1315.6103, 1334.6603, 1402.7407, 1408.7587, 1422.7041, 1424.7200, 1690.6642, 1706.9716, 1723.9839

3. LPHUA1 **Mass:** 30759 **Score:** **208** **Expect:** 5.1e-15 **Queries matched:** 21

apolipoprotein A-I precursor [validated] - human

Observed	Mr(expt)	Mr(calc)	Delta	Start	End	Miss	Peptide
781.4592	780.4520	780.4242	0.0278	178	- 184	0	R.AHVDALR.T
831.4419	830.4346	830.4286	0.0060	213	- 219	0	R.LAEYHAK.A
873.4641	872.4568	872.4352	0.0217	148	- 155	0	R.AELQEGAR.Q
1012.6155	1011.6083	1011.5713	0.0370	231	- 239	0	K.AKPALEDLR.Q
1157.6270	1156.6198	1156.6200	-0.0002	202	- 212	1	R.LEALKENGGAR.L
1230.7044	1229.6971	1229.7019	-0.0048	240	- 250	0	R.QGLLPVLESFK.V
1235.6798	1234.6726	1234.6809	-0.0083	37	- 47	0	K.DLATVYVDVLK.D
1252.6795	1251.6722	1251.6135	0.0587	121	- 130	0	K.VQPYLDDFQK.K
1283.6177	1282.6105	1282.5652	0.0453	132	- 140	0	K.WQEEMELYR.Q
1299.6059	1298.5987	1298.5601	0.0386	132	- 140	0	K.WQEEMELYR.Q + Oxidation (M)
1301.6870	1300.6797	1300.6411	0.0385	185	- 195	0	R.THLAPYSDELR.Q
1318.6687	1317.6615	1317.6347	0.0268	165	- 175	1	K.LSPLGEEMRDR.A + Oxidation (M)
1380.7520	1379.7447	1379.7085	0.0362	121	- 131	1	K.VQPYLDDFQKK.W
1386.7403	1385.7330	1385.7078	0.0252	251	- 262	0	K.VSFLSALEEYTK.K
1400.6846	1399.6774	1399.6619	0.0155	52	- 64	0	R.DYVSQFEGSALGK.Q
1427.7243	1426.7170	1426.6551	0.0620	131	- 140	1	K.KWQEEMELYR.Q + Oxidation (M)
1462.8779	1461.8706	1461.8442	0.0264	35	- 47	1	R.VKDLATVYVDVLK.D
1467.8310	1466.8238	1466.7841	0.0397	143	- 155	1	K.VEPLRAELQEGAR.Q

1612.8208	1611.8135	1611.7780	0.0355	70 -	83	0	K.LLDNWDSVTSTFSK.L
1815.9168	1814.9096	1814.8434	0.0661	48 -	64	1	K.DSGRDYVSQFEGSALGK.Q
2202.1952	2201.1880	2201.1116	0.0763	84 -	101	1	K.LREQLGPVTQEFWDNLEK.E

No match to: 712.2679, 825.1370, 832.3588, 833.3959, 841.0980, 852.4849, 853.5010, 861.0837, 876.3626, 877.0567, 1158.6512, 1213.7102, 1224.6023, 1226.5773, 1238.8028, 1240.5956, 1242.5818, 1257.6582, 1257.7440, 1258.5811, 1274.6199, 1282.5765, 1298.6028, 1315.6103, 1334.6603, 1402.7407, 1408.7587, 1422.7041, 1424.7200, 1690.6642, 1706.9716, 1723.9839

4. AAX42892 **Mass:** 30872 **Score:** 207 **Expect:** 6.5e-15 **Queries matched:** 21

AY890956 NID: - synthetic construct

Observed	Mr(expt)	Mr(calc)	Delta	Start	End	Miss	Peptide
781.4592	780.4520	780.4242	0.0278	178 -	184	0	R.AHVDALR.T
831.4419	830.4346	830.4286	0.0060	213 -	219	0	R.LAEYHAK.A
873.4641	872.4568	872.4352	0.0217	148 -	155	0	R.AELQEGAR.Q
1012.6155	1011.6083	1011.5713	0.0370	231 -	239	0	K.AKPALEDLR.Q
1157.6270	1156.6198	1156.6200	-0.0002	202 -	212	1	R.LEALKENGGAR.L
1230.7044	1229.6971	1229.7019	-0.0048	240 -	250	0	R.QGLLPVLESFK.V
1235.6798	1234.6726	1234.6809	-0.0083	37 -	47	0	K.DLATVYVDVLK.D
1252.6795	1251.6722	1251.6135	0.0587	121 -	130	0	K.VQPYLDDFQK.K
1283.6177	1282.6105	1282.5652	0.0453	132 -	140	0	K.WQEEMELYR.Q
1299.6059	1298.5987	1298.5601	0.0386	132 -	140	0	K.WQEEMELYR.Q + Oxidation (M)
1301.6870	1300.6797	1300.6411	0.0385	185 -	195	0	R.THLAPYSDELR.Q
1318.6687	1317.6615	1317.6347	0.0268	165 -	175	1	K.LSPLGEEMRDR.A + Oxidation (M)
1380.7520	1379.7447	1379.7085	0.0362	121 -	131	1	K.VQPYLDDFQKK.W
1386.7403	1385.7330	1385.7078	0.0252	251 -	262	0	K.VSFLSALEEYTK.K
1400.6846	1399.6774	1399.6619	0.0155	52 -	64	0	R.DYVSQFEGSALGK.Q
1427.7243	1426.7170	1426.6551	0.0620	131 -	140	1	K.KWQEEMELYR.Q + Oxidation (M)
1462.8779	1461.8706	1461.8442	0.0264	35 -	47	1	R.VKDLATVYVDVLK.D
1467.8310	1466.8238	1466.7841	0.0397	143 -	155	1	K.VEPLRAELQEGAR.Q
1612.8208	1611.8135	1611.7780	0.0355	70 -	83	0	K.LLDNWDSVTSTFSK.L
1815.9168	1814.9096	1814.8434	0.0661	48 -	64	1	K.DSGRDYVSQFEGSALGK.Q
2202.1952	2201.1880	2201.1116	0.0763	84 -	101	1	K.LREQLGPVTQEFWDNLEK.E

No match to: 712.2679, 825.1370, 832.3588, 833.3959, 841.0980, 852.4849, 853.5010, 861.0837, 876.3626, 877.0567, 1158.6512, 1213.7102, 1224.6023, 1226.5773, 1238.8028, 1240.5956, 1242.5818, 1257.6582, 1257.7440, 1258.5811, 1274.6199, 1282.5765, 1298.6028, 1315.6103, 1334.6603, 1402.7407, 1408.7587, 1422.7041, 1424.7200, 1690.6642, 1706.9716, 1723.9839

5. AAA35545 **Mass:** 30745 **Score:** 192 **Expect:** 2e-13 **Queries matched:** 20

HUMAPOAIP NID: - Homo sapiens

Observed	Mr(expt)	Mr(calc)	Delta	Start	End	Miss	Peptide
781.4592	780.4520	780.4242	0.0278	178 -	184	0	R.AHVDALR.T
831.4419	830.4346	830.4286	0.0060	213 -	219	0	R.LAEYHAK.A
873.4641	872.4568	872.4352	0.0217	148 -	155	0	R.AELQEGAR.Q
1012.6155	1011.6083	1011.5713	0.0370	231 -	239	0	K.AKPALEDLR.Q
1157.6270	1156.6198	1156.6200	-0.0002	202 -	212	1	R.LEALKENGGAR.L
1230.7044	1229.6971	1229.7019	-0.0048	240 -	250	0	R.QGLLPVLESFK.V
1235.6798	1234.6726	1234.6809	-0.0083	37 -	47	0	K.DLATVYVDVLK.D
1252.6795	1251.6722	1251.6135	0.0587	121 -	130	0	K.VQPYLDDFQK.K
1283.6177	1282.6105	1282.5652	0.0453	132 -	140	0	K.WQEEMELYR.Q
1299.6059	1298.5987	1298.5601	0.0386	132 -	140	0	K.WQEEMELYR.Q + Oxidation (M)
1301.6870	1300.6797	1300.6411	0.0385	185 -	195	0	R.THLAPYSDELR.Q
1318.6687	1317.6615	1317.6347	0.0268	165 -	175	1	K.LSPLGEEMRDR.A + Oxidation (M)
1380.7520	1379.7447	1379.7085	0.0362	121 -	131	1	K.VQPYLDDFQKK.W
1386.7403	1385.7330	1385.7078	0.0252	251 -	262	0	K.VSFLSALEEYTK.K
1400.6846	1399.6774	1399.6619	0.0155	52 -	64	0	R.DYVSQFEGSALGK.Q
1427.7243	1426.7170	1426.6551	0.0620	131 -	140	1	K.KWQEEMELYR.Q + Oxidation (M)
1462.8779	1461.8706	1461.8442	0.0264	35 -	47	1	R.VKDLATVYVDVLK.D
1612.8208	1611.8135	1611.7780	0.0355	70 -	83	0	K.LLDNWDSVTSTFSK.L
1815.9168	1814.9096	1814.8434	0.0661	48 -	64	1	K.DSGRDYVSQFEGSALGK.Q
2202.1952	2201.1880	2201.1116	0.0763	84 -	101	1	K.LREQLGPVTQEFWDNLEK.E

No match to: 712.2679, 825.1370, 832.3588, 833.3959, 841.0980, 852.4849, 853.5010, 861.0837, 876.3626, 877.0567, 1158.6512, 1213.7102, 1224.6023, 1226.5773, 1238.8028, 1240.5956, 1242.5818, 1257.6582, 1257.7440, 1258.5811, 1274.6199, 1282.5765, 1298.6028, 1315.6103, 1334.6603, 1402.7407, 1408.7587, 1422.7041, 1424.7200, 1467.8310, 1690.6642, 1706.9716, 1723.9839

6. CAA03490 **Mass:** 23205 **Score:** **160** **Expect:** 3.2e-10 **Queries matched:** 17

SEQUENCE 10 FROM PATENT WO9637608.- unidentified.

Observed	Mr(expt)	Mr(calc)	Delta	Start		End	Miss	Peptide
781.4592	780.4520	780.4242	0.0278	111	-	117	0	R.AHVDALR.T
831.4419	830.4346	830.4286	0.0060	146	-	152	0	R.LAEYHAK.A
873.4641	872.4568	872.4352	0.0217	81	-	88	0	R.AELQEGAR.Q
1012.6155	1011.6083	1011.5713	0.0370	164	-	172	0	K.AKPALEDLR.Q
1157.6270	1156.6198	1156.6200	-0.0002	135	-	145	1	R.LEALKENGGAR.L
1226.5773	1225.5700	1225.5986	-0.0286	107	-	117	1	R.DCARAHVDALR.T
1230.7044	1229.6971	1229.7019	-0.0048	173	-	183	0	R.QGLLPVLESFK.V
1252.6795	1251.6722	1251.6135	0.0587	54	-	63	0	K.VQPYLDDFQK.K
1283.6177	1282.6105	1282.6200	-0.0096	107	-	117	1	R.DCARAHVDALR.T + Carbamidomethyl (C)
1299.6059	1298.5987	1298.5601	0.0386	65	-	73	0	K.WQEEMELYR.Q + Oxidation (M)
1301.6870	1300.6797	1300.6411	0.0385	118	-	128	0	R.THLAPYSDELR.Q
1380.7520	1379.7447	1379.7085	0.0362	54	-	64	1	K.VQPYLDDFQKK.W
1386.7403	1385.7330	1385.7078	0.0252	184	-	195	0	K.VSFLSALEEYTK.K
1427.7243	1426.7170	1426.6551	0.0620	64	-	73	1	K.KWQEEMELYR.Q + Oxidation (M)
1467.8310	1466.8238	1466.7841	0.0397	76	-	88	1	K.VEPLRAELQEGAR.Q
1612.8208	1611.8135	1611.7780	0.0355	3	-	16	0	K.LLDNWDSVTSTFSK.L
2202.1952	2201.1880	2201.1116	0.0763	17	-	34	1	K.LREQLGPVTQEFWDNLEK.E

No match to: 712.2679, 825.1370, 832.3588, 833.3959, 841.0980, 852.4849, 853.5010, 861.0837, 876.3626, 877.0567, 1158.6512, 1213.7102, 1224.6023, 1235.6798, 1238.8028, 1240.5956, 1242.5818, 1257.6582, 1257.7440, 1258.5811, 1274.6199, 1282.5765, 1298.6028, 1315.6103, 1318.6687, 1334.6603, 1400.6846, 1402.7407, 1408.7587, 1422.7041, 1424.7200, 1462.8779, 1690.6642, 1706.9716, 1723.9839, 1815.9168

7. 1AV1A **Mass:** 23389 **Score:** **160** **Expect:** 3.2e-10 **Queries matched:** 17

apolipoprotein a-i lipid-binding domain mutant N-TERMINAL MET, DEL(1-43), chain A - human

Observed	Mr(expt)	Mr(calc)	Delta	Start		End	Miss	Peptide
781.4592	780.4520	780.4242	0.0278	112	-	118	0	R.AHVDALR.T
831.4419	830.4346	830.4286	0.0060	147	-	153	0	R.LAEYHAK.A
873.4641	872.4568	872.4352	0.0217	82	-	89	0	R.AELQEGAR.Q
1012.6155	1011.6083	1011.5713	0.0370	165	-	173	0	K.AKPALEDLR.Q
1157.6270	1156.6198	1156.6200	-0.0002	136	-	146	1	R.LEALKENGGAR.L
1230.7044	1229.6971	1229.7019	-0.0048	174	-	184	0	R.QGLLPVLESFK.V
1252.6795	1251.6722	1251.6135	0.0587	55	-	64	0	K.VQPYLDDFQK.K
1283.6177	1282.6105	1282.5652	0.0453	66	-	74	0	K.WQEEMELYR.Q
1299.6059	1298.5987	1298.5601	0.0386	66	-	74	0	K.WQEEMELYR.Q + Oxidation (M)
1301.6870	1300.6797	1300.6411	0.0385	119	-	129	0	R.THLAPYSDELR.Q
1318.6687	1317.6615	1317.6347	0.0268	99	-	109	1	K.LSPLGEEMRDR.A + Oxidation (M)
1380.7520	1379.7447	1379.7085	0.0362	55	-	65	1	K.VQPYLDDFQKK.W
1386.7403	1385.7330	1385.7078	0.0252	185	-	196	0	K.VSFLSALEEYTK.K
1427.7243	1426.7170	1426.6551	0.0620	65	-	74	1	K.KWQEEMELYR.Q + Oxidation (M)
1467.8310	1466.8238	1466.7841	0.0397	77	-	89	1	K.VEPLRAELQEGAR.Q
1612.8208	1611.8135	1611.7780	0.0355	4	-	17	0	K.LLDNWDSVTSTFSK.L
2202.1952	2201.1880	2201.1116	0.0763	18	-	35	1	K.LREQLGPVTQEFWDNLEK.E

No match to: 712.2679, 825.1370, 832.3588, 833.3959, 841.0980, 852.4849, 853.5010, 861.0837, 876.3626, 877.0567, 1158.6512, 1213.7102, 1224.6023, 1226.5773, 1235.6798, 1238.8028, 1240.5956, 1242.5818, 1257.6582, 1257.7440, 1258.5811, 1274.6199, 1282.5765, 1298.6028, 1315.6103, 1334.6603, 1400.6846, 1402.7407, 1408.7587, 1422.7041, 1424.7200, 1462.8779, 1690.6642, 1706.9716, 1723.9839, 1815.9168

8. Q8HZ97_PANTR **Mass:** 22075 **Score:** **130** **Expect:** 3.2e-07 **Queries matched:** 14

Apolipoprotein A-I (Fragment).- Pan troglodytes (Chimpanzee).

Observed	Mr(expt)	Mr(calc)	Delta	Start		End	Miss	Peptide
781.4592	780.4520	780.4242	0.0278	141	-	147	0	R.AHVDALR.T
831.4419	830.4346	830.4286	0.0060	176	-	182	0	R.LAEYHAK.A
873.4641	872.4568	872.4352	0.0217	111	-	118	0	R.AELQEGAR.Q
1157.6270	1156.6198	1156.6200	-0.0002	165	-	175	1	R.LEALKENGGAR.L
1252.6795	1251.6722	1251.6135	0.0587	84	-	93	0	K.VQPYLDDFQK.K

1283.6177	1282.6105	1282.5652	0.0453	95 -	103	0	K.WQEEMELYR.Q
1299.6059	1298.5987	1298.5601	0.0386	95 -	103	0	K.WQEEMELYR.Q + Oxidation (M)
1301.6870	1300.6797	1300.6411	0.0385	148 -	158	0	R.THLAPYSDELR.Q
1380.7520	1379.7447	1379.7085	0.0362	84 -	94	1	K.VQPYLDDFQKK.W
1400.6846	1399.6774	1399.6619	0.0155	15 -	27	0	R.DYVSQFEGSALGK.Q
1427.7243	1426.7170	1426.6551	0.0620	94 -	103	1	K.KWQEEMELYR.Q + Oxidation (M)
1612.8208	1611.8135	1611.7780	0.0355	33 -	46	0	K.LLDNWDSVTSTFSK.L
1815.9168	1814.9096	1814.8434	0.0661	11 -	27	1	K.DSGRDYVSQFEGSALGK.Q
2202.1952	2201.1880	2201.1116	0.0763	47 -	64	1	K.LREQLGPVTQEFWDNLEK.E

No match to: 712.2679, 825.1370, 832.3588, 833.3959, 841.0980, 852.4849, 853.5010, 861.0837, 876.3626, 877.0567, 1012.6155, 1158.6512, 1213.7102, 1224.6023, 1226.5773, 1230.7044, 1235.6798, 1238.8028, 1240.5956, 1242.5818, 1257.6582, 1257.7440, 1258.5811, 1274.6199, 1282.5765, 1298.6028, 1315.6103, 1318.6687, 1334.6603, 1386.7403, 1402.7407, 1408.7587, 1422.7041, 1424.7200, 1462.8779, 1467.8310, 1690.6642, 1706.9716, 1723.9839

9. Q8HZ95_PONPY **Mass:** 22116 **Score:** 113 **Expect:** 1.6e-05 **Queries matched:** 13

Apolipoprotein A-I (Fragment).- Pongo pygmaeus (Orangutan).

Observed	Mr(expt)	Mr(calc)	Delta	Start	End	Miss	Peptide
781.4592	780.4520	780.4242	0.0278	141 -	147	0	R.AHVDALR.T
831.4419	830.4346	830.4286	0.0060	176 -	182	0	R.LAEYHAK.A
873.4641	872.4568	872.4352	0.0217	111 -	118	0	R.AELQEGAR.Q
1157.6270	1156.6198	1156.6200	-0.0002	165 -	175	1	R.LEALKENGGAR.L
1252.6795	1251.6722	1251.6135	0.0587	84 -	93	0	K.VQPYLDDFQK.K
1283.6177	1282.6105	1282.5652	0.0453	95 -	103	0	K.WQEEMELYR.Q
1299.6059	1298.5987	1298.5601	0.0386	95 -	103	0	K.WQEEMELYR.Q + Oxidation (M)
1315.6103	1314.6030	1314.6568	-0.0538	148 -	158	0	R.THLAPYTDELR.Q
1380.7520	1379.7447	1379.7085	0.0362	84 -	94	1	K.VQPYLDDFQKK.W
1400.6846	1399.6774	1399.6619	0.0155	15 -	27	0	R.DYVSQFEGSALGK.Q
1427.7243	1426.7170	1426.6551	0.0620	94 -	103	1	K.KWQEEMELYR.Q + Oxidation (M)
1815.9168	1814.9096	1814.8434	0.0661	11 -	27	1	K.DSGRDYVSQFEGSALGK.Q
2202.1952	2201.1880	2201.1116	0.0763	47 -	64	1	K.LREQLGPVTQEFWDNLEK.E

No match to: 712.2679, 825.1370, 832.3588, 833.3959, 841.0980, 852.4849, 853.5010, 861.0837, 876.3626, 877.0567, 1012.6155, 1158.6512, 1213.7102, 1224.6023, 1226.5773, 1230.7044, 1235.6798, 1238.8028, 1240.5956, 1242.5818, 1257.6582, 1257.7440, 1258.5811, 1274.6199, 1282.5765, 1298.6028, 1301.6870, 1318.6687, 1334.6603, 1386.7403, 1402.7407, 1408.7587, 1422.7041, 1424.7200, 1462.8779, 1467.8310, 1612.8208, 1690.6642, 1706.9716, 1723.9839

10. A26529 **Mass:** 30700 **Score:** 107 **Expect:** 6.5e-05 **Queries matched:** 14

apolipoprotein A-I precursor - crab-eating macaque

Observed	Mr(expt)	Mr(calc)	Delta	Start	End	Miss	Peptide
781.4592	780.4520	780.4242	0.0278	178 -	184	0	R.AHVDALR.T
831.4419	830.4346	830.4286	0.0060	213 -	219	0	R.LAEYHAK.A
1012.6155	1011.6083	1011.5713	0.0370	231 -	239	0	K.AKPALEDLR.Q
1157.6270	1156.6198	1156.6200	-0.0002	202 -	212	1	R.LEALKENGGAR.L
1230.7044	1229.6971	1229.7019	-0.0048	240 -	250	0	R.QGLLPVLESFK.V
1252.6795	1251.6722	1251.6135	0.0587	121 -	130	0	K.VQPYLDDFQK.K
1283.6177	1282.6105	1282.5652	0.0453	132 -	140	0	K.WQEEMELYR.Q
1299.6059	1298.5987	1298.5601	0.0386	132 -	140	0	K.WQEEMELYR.Q + Oxidation (M)
1301.6870	1300.6797	1300.6411	0.0385	185 -	195	0	R.THLAPYSDELR.Q
1380.7520	1379.7447	1379.7085	0.0362	121 -	131	1	K.VQPYLDDFQKK.W
1386.7403	1385.7330	1385.7078	0.0252	251 -	262	0	K.VSFLSALEEYTK.K
1400.6846	1399.6774	1399.6619	0.0155	52 -	64	0	K.DYVSQFEGSALGK.Q
1427.7243	1426.7170	1426.6551	0.0620	131 -	140	1	K.KWQEEMELYR.Q + Oxidation (M)
2202.1952	2201.1880	2201.1116	0.0763	84 -	101	1	K.LREQLGPVTQEFWDNLEK.E

No match to: 712.2679, 825.1370, 832.3588, 833.3959, 841.0980, 852.4849, 853.5010, 861.0837, 873.4641, 876.3626, 877.0567, 1158.6512, 1213.7102, 1224.6023, 1226.5773, 1235.6798, 1238.8028, 1240.5956, 1242.5818, 1257.6582, 1257.7440, 1258.5811, 1274.6199, 1282.5765, 1298.6028, 1315.6103, 1318.6687, 1334.6603, 1402.7407, 1408.7587, 1422.7041, 1424.7200, 1462.8779, 1467.8310, 1612.8208, 1690.6642, 1706.9716, 1723.9839, 1815.9168

11. JS0079 **Mass:** 30716 **Score:** 107 **Expect:** 6.5e-05 **Queries matched:** 14

apolipoprotein A-I precursor - baboon

Observed	Mr(expt)	Mr(calc)	Delta	Start	End	Miss	Peptide
781.4592	780.4520	780.4242	0.0278	178 -	184	0	R.AHVDALR.T
831.4419	830.4346	830.4286	0.0060	213 -	219	0	R.LAEYHAK.A
1012.6155	1011.6083	1011.5713	0.0370	231 -	239	0	K.AKPALEDLR.Q

Observed	Mr(expt)	Mr(calc)	Delta	Start	End	Miss	Peptide
1157.6270	1156.6198	1156.6200	-0.0002	202 -	212	1	R.LEALKENGGAR.L
1230.7044	1229.6971	1229.7019	-0.0048	240 -	250	0	R.QGLLPVLESFK.V
1252.6795	1251.6722	1251.6135	0.0587	121 -	130	0	K.VQPYLDDFQK.K
1283.6177	1282.6105	1282.5652	0.0453	132 -	140	0	K.WQEEMELYR.Q
1299.6059	1298.5987	1298.5601	0.0386	132 -	140	0	K.WQEEMELYR.Q + Oxidation (M)
1301.6870	1300.6797	1300.6411	0.0385	185 -	195	0	R.THLAPYSDELR.Q
1380.7520	1379.7447	1379.7085	0.0362	121 -	131	1	K.VQPYLDDFQKK.W
1386.7403	1385.7330	1385.7078	0.0252	251 -	262	0	K.VSFLSALEEYTK.K
1400.6846	1399.6774	1399.6619	0.0155	52 -	64	0	K.DYVSQFEGSALGK.Q
1427.7243	1426.7170	1426.6551	0.0620	131 -	140	1	K.KWQEEMELYR.Q + Oxidation (M)
2202.1952	2201.1880	2201.1116	0.0763	84 -	101	1	K.LREQLGPVTQEFWDNLEK.E

No match to: 712.2679, 825.1370, 832.3588, 833.3959, 841.0980, 852.4849, 853.5010, 861.0837, 873.4641, 876.3626, 877.0567, 1158.6512, 1213.7102, 1224.6023, 1226.5773, 1235.6798, 1238.8028, 1240.5956, 1242.5818, 1257.6582, 1257.7440, 1258.5811, 1274.6199, 1282.5765, 1298.6028, 1315.6103, 1318.6687, 1334.6603, 1402.7407, 1408.7587, 1422.7041, 1424.7200, 1462.8779, 1467.8310, 1612.8208, 1690.6642, 1706.9716, 1723.9839, 1815.9168

12. CAD61352 **Mass:** 18392 **Score:** 74 **Expect:** 0.14 **Queries matched:** 9

Sequence 2 from Patent WO02083898 precursor.- Homo sapiens (Human).

Observed	Mr(expt)	Mr(calc)	Delta	Start	End	Miss	Peptide
1235.6798	1234.6726	1234.6809	-0.0083	37 -	47	0	K.DLATVYVDVLK.D
1252.6795	1251.6722	1251.6135	0.0587	121 -	130	0	K.VQPYLDDFQK.K
1283.6177	1282.6105	1282.5652	0.0453	132 -	140	0	K.WQEEMELYR.Q
1299.6059	1298.5987	1298.5601	0.0386	132 -	140	0	K.WQEEMELYR.Q + Oxidation (M)
1380.7520	1379.7447	1379.7085	0.0362	121 -	131	1	K.VQPYLDDFQKK.W
1400.6846	1399.6774	1399.6619	0.0155	52 -	64	0	R.DYVSQFEGSALGK.Q
1427.7243	1426.7170	1426.6551	0.0620	131 -	140	1	K.KWQEEMELYR.Q + Oxidation (M)
1462.8779	1461.8706	1461.8442	0.0264	35 -	47	1	R.VKDLATVYVDVLK.D
1815.9168	1814.9096	1814.8434	0.0661	48 -	64	1	K.DSGRDYVSQFEGSALGK.Q

No match to: 712.2679, 781.4592, 825.1370, 831.4419, 832.3588, 833.3959, 841.0980, 852.4849, 853.5010, 861.0837, 873.4641, 876.3626, 877.0567, 1012.6155, 1157.6270, 1158.6512, 1213.7102, 1224.6023, 1226.5773, 1230.7044, 1238.8028, 1240.5956, 1242.5818, 1257.6582, 1257.7440, 1258.5811, 1274.6199, 1282.5765, 1298.6028, 1301.6870, 1315.6103, 1318.6687, 1334.6603, 1386.7403, 1402.7407, 1408.7587, 1422.7041, 1424.7200, 1467.8310, 1612.8208, 1690.6642, 1706.9716, 1723.9839, 2202.1952

13. Q31VN4_SHIBS **Mass:** 15504 **Score:** 69 **Expect:** 0.44 **Queries matched:** 10

Hypothetical protein yrfH.- Shigella boydii serotype 4 (strain Sb227).

Observed	Mr(expt)	Mr(calc)	Delta	Start	End	Miss	Peptide
852.4849	851.4776	851.3993	0.0783	96 -	102	1	R.EKMAMAR.K + Oxidation (M)
873.4641	872.4568	872.4253	0.0316	36 -	42	0	K.VHYNGQR.S
1158.6512	1157.6440	1157.6345	0.0094	11 -	19	1	R.LDKWLWAAR.F
1240.5956	1239.5884	1239.6553	-0.0669	14 -	22	1	K.WLWAARFYK.T
1257.6582	1256.6509	1256.7452	-0.0943	65 -	75	1	R.TVIVKAITEQR.R
1257.7440	1256.7367	1256.7452	-0.0085	65 -	75	1	R.TVIVKAITEQR.R
1258.5811	1257.5738	1257.6677	-0.0939	59 -	69	1	R.QGNDERTVIVK.A
1283.6177	1282.6105	1282.7244	-0.1140	3 -	13	1	K.EKPAVEVRLDK.W
1400.6846	1399.6774	1399.7320	-0.0546	36 -	47	1	K.VHYNGQRSKPSK.I
1408.7587	1407.7515	1407.7292	0.0222	103 -	114	1	R.KLNALTMPHPDR.R + Oxidation (M)

No match to: 712.2679, 781.4592, 825.1370, 831.4419, 832.3588, 833.3959, 841.0980, 853.5010, 861.0837, 876.3626, 877.0567, 1012.6155, 1157.6270, 1213.7102, 1224.6023, 1226.5773, 1230.7044, 1235.6798, 1238.8028, 1242.5818, 1252.6795, 1274.6199, 1282.5765, 1298.6028, 1299.6059, 1301.6870, 1315.6103, 1318.6687, 1334.6603, 1380.7520, 1386.7403, 1402.7407, 1422.7041, 1424.7200, 1427.7243, 1462.8779, 1467.8310, 1612.8208, 1690.6642, 1706.9716, 1723.9839, 1815.9168, 2202.1952

14. Q8ZLJ5_SALTY **Mass:** 15511 **Score:** 68 **Expect:** 0.5 **Queries matched:** 10

Heat shock protein.- Salmonella typhimurium.

Observed	Mr(expt)	Mr(calc)	Delta	Start	End	Miss	Peptide
833.3959	832.3886	832.4225	-0.0339	96 -	102	1	R.EKMAQAR.K
873.4641	872.4568	872.4253	0.0316	36 -	42	0	K.VHYNGQR.S
1158.6512	1157.6440	1157.6345	0.0094	11 -	19	1	R.LDKWLWAAR.F
1224.6023	1223.5950	1223.5638	0.0313	25 -	35	1	R.AMAREMIEGGK.V + 2 Oxidation (M)
1240.5956	1239.5884	1239.6553	-0.0669	14 -	22	1	K.WLWAARFYK.T
1257.6582	1256.6509	1256.7452	-0.0943	65 -	75	1	R.TVIVKAITEQR.R
1257.7440	1256.7367	1256.7452	-0.0085	65 -	75	1	R.TVIVKAITEQR.R

```
1258.5811  1257.5738  1257.6677  -0.0939    59 -  69   1  R.QGNDERTVIVK.A
1400.6846  1399.6774  1399.7320  -0.0546    36 -  47   1  K.VHYNGQRSKPSK.I
1408.7587  1407.7515  1407.7292   0.0222   103 - 114   1  R.KLNALTMPHPDR.R + Oxidation (M)
```

No match to: 712.2679, 781.4592, 825.1370, 831.4419, 832.3588, 841.0980, 852.4849, 853.5010, 861.0837, 876.3626, 877.0567, 1012.6155, 1157.6270, 1213.7102, 1226.5773, 1230.7044, 1235.6798, 1238.8028, 1242.5818, 1252.6795, 1274.6199, 1282.5765, 1283.6177, 1298.6028, 1299.6059, 1301.6870, 1315.6103, 1318.6687, 1334.6603, 1380.7520, 1386.7403, 1402.7407, 1422.7041, 1424.7200, 1427.7243, 1462.8779, 1467.8310, 1612.8208, 1690.6642, 1706.9716, 1723.9839, 1815.9168, 2202.1952

15. H86004 **Mass:** 15486 **Score:** 61 **Expect:** 2.7 **Queries matched:** 9

ribosome-associated heat shock protein yrfH [similarity] - Escherichia coli (strain O157:H7, substrain EDL933)

Observed	Mr(expt)	Mr(calc)	Delta	Start	End	Miss	Peptide
873.4641	872.4568	872.4253	0.0316	36 -	42	0	K.VHYNGQR.S
1158.6512	1157.6440	1157.6345	0.0094	11 -	19	1	R.LDKWLWAAR.F
1240.5956	1239.5884	1239.6553	-0.0669	14 -	22	1	K.WLWAARFYK.T
1257.6582	1256.6509	1256.7452	-0.0943	65 -	75	1	R.TVIVKAITEQR.R
1257.7440	1256.7367	1256.7452	-0.0085	65 -	75	1	R.TVIVKAITEQR.R
1258.5811	1257.5738	1257.6677	-0.0939	59 -	69	1	R.QGNDERTVIVK.A
1283.6177	1282.6105	1282.7244	-0.1140	3 -	13	1	K.EKPAVEVRLDK.W
1400.6846	1399.6774	1399.7320	-0.0546	36 -	47	1	K.VHYNGQRSKPSK.I
1408.7587	1407.7515	1407.7292	0.0222	103 -	114	1	R.KLNALTMPHPDR.R + Oxidation (M)

No match to: 712.2679, 781.4592, 825.1370, 831.4419, 832.3588, 833.3959, 841.0980, 852.4849, 853.5010, 861.0837, 876.3626, 877.0567, 1012.6155, 1157.6270, 1213.7102, 1224.6023, 1226.5773, 1230.7044, 1235.6798, 1238.8028, 1242.5818, 1252.6795, 1274.6199, 1282.5765, 1298.6028, 1299.6059, 1301.6870, 1315.6103, 1318.6687, 1334.6603, 1380.7520, 1386.7403, 1402.7407, 1422.7041, 1424.7200, 1427.7243, 1462.8779, 1467.8310, 1612.8208, 1690.6642, 1706.9716, 1723.9839, 1815.9168, 2202.1952

16. H97327 **Mass:** 17433 **Score:** 59 **Expect:** 3.9 **Queries matched:** 7

transcription regulator, MarR/EmrR family [imported] - Clostridium acetobutylicum

Observed	Mr(expt)	Mr(calc)	Delta	Start	End	Miss	Peptide
832.3588	831.3515	831.3835	-0.0320	2 -	8	0	M.NNNTQNK.T
1158.6512	1157.6440	1157.5352	0.1087	121 -	129	1	K.EKESAFYER.I
1213.7102	1212.7029	1212.6138	0.0891	123 -	132	1	K.ESAFYERIAK.W
1224.6023	1223.5950	1223.5928	0.0022	70 -	80	1	K.SIAMDRSSVSR.L + Oxidation (M)
1230.7044	1229.6971	1229.7091	-0.0120	76 -	86	1	R.SSVSRLVNQLK.N
1318.6687	1317.6615	1317.6021	0.0593	2 -	12	1	M.NNNTQNKTDNR.I
1422.7041	1421.6968	1421.7700	-0.0732	81 -	92	1	R.LVNQLKNMGYVK.S + Oxidation (M)

No match to: 712.2679, 781.4592, 825.1370, 831.4419, 833.3959, 841.0980, 852.4849, 853.5010, 861.0837, 873.4641, 876.3626, 877.0567, 1012.6155, 1157.6270, 1226.5773, 1235.6798, 1238.8028, 1240.5956, 1242.5818, 1252.6795, 1257.6582, 1257.7440, 1258.5811, 1274.6199, 1282.5765, 1283.6177, 1298.6028, 1299.6059, 1301.6870, 1315.6103, 1334.6603, 1380.7520, 1386.7403, 1400.6846, 1402.7407, 1408.7587, 1424.7200, 1427.7243, 1462.8779, 1467.8310, 1612.8208, 1690.6642, 1706.9716, 1723.9839, 1815.9168, 2202.1952

17. Q57IX7_SALCH **Mass:** 15557 **Score:** 59 **Expect:** 4.2 **Queries matched:** 10

Heat shock protein, predicted small RNA-binding protein.- Salmonella choleraesuis.

Observed	Mr(expt)	Mr(calc)	Delta	Start	End	Miss	Peptide
833.3959	832.3886	832.4225	-0.0339	96 -	102	1	R.EKMAQAR.K
873.4641	872.4568	872.4253	0.0316	36 -	42	0	K.VHYNGQR.S
1158.6512	1157.6440	1157.6345	0.0094	11 -	19	1	R.LDKWLWAAR.F
1240.5956	1239.5884	1239.6553	-0.0669	14 -	22	1	K.WLWAARFYK.T
1257.6582	1256.6509	1256.7452	-0.0943	65 -	75	1	R.TVIVKAITEQR.R
1257.7440	1256.7367	1256.7452	-0.0085	65 -	75	1	R.TVIVKAITEQR.R
1258.5811	1257.5738	1257.6677	-0.0939	59 -	69	1	R.QGNDERTVIVK.A
1400.6846	1399.6774	1399.7320	-0.0546	36 -	47	1	K.VHYNGQRSKPSK.I
1408.7587	1407.7515	1407.7292	0.0222	103 -	114	1	R.KLNSLTMPHPDR.R
1424.7200	1423.7128	1423.7242	-0.0114	103 -	114	1	R.KLNSLTMPHPDR.R + Oxidation (M)

No match to: 712.2679, 781.4592, 825.1370, 831.4419, 832.3588, 841.0980, 852.4849, 853.5010, 861.0837, 876.3626, 877.0567, 1012.6155, 1157.6270, 1213.7102, 1224.6023, 1226.5773, 1230.7044, 1235.6798, 1238.8028, 1242.5818, 1252.6795, 1274.6199, 1282.5765, 1283.6177, 1298.6028, 1299.6059, 1301.6870, 1315.6103, 1318.6687, 1334.6603, 1380.7520, 1386.7403, 1402.7407, 1422.7041, 1427.7243, 1462.8779, 1467.8310, 1612.8208, 1690.6642, 1706.9716, 1723.9839, 1815.9168, 2202.1952

18. Q5PLY1_SALPA **Mass:** 15465 **Score:** 58 **Expect:** 4.8 **Queries matched:** 9

Heat shock protein.- Salmonella paratyphi-a.

Observed	Mr(expt)	Mr(calc)	Delta	Start		End	Miss	Peptide
833.3959	832.3886	832.4225	-0.0339	96	-	102	1	R.EKMAQAR.K
873.4641	872.4568	872.4253	0.0316	36	-	42	0	K.VHYNGQR.S
1158.6512	1157.6440	1157.6345	0.0094	11	-	19	1	R.LDKWLWAAR.F
1240.5956	1239.5884	1239.6553	-0.0669	14	-	22	1	K.WLWAARFYK.T
1257.6582	1256.6509	1256.7452	-0.0943	65	-	75	1	R.TVIVKAITEQR.R
1257.7440	1256.7367	1256.7452	-0.0085	65	-	75	1	R.TVIVKAITEQR.R
1258.5811	1257.5738	1257.6677	-0.0939	59	-	69	1	R.QGNDERTVIVK.A
1400.6846	1399.6774	1399.7320	-0.0546	36	-	47	1	K.VHYNGQRSKPSK.I
1408.7587	1407.7515	1407.7292	0.0222	103	-	114	1	R.KLNALTMPHPDR.R + Oxidation (M)

No match to: 712.2679, 781.4592, 825.1370, 831.4419, 832.3588, 841.0980, 852.4849, 853.5010, 861.0837, 876.3626, 877.0567, 1012.6155, 1157.6270, 1213.7102, 1224.6023, 1226.5773, 1230.7044, 1235.6798, 1238.8028, 1242.5818, 1252.6795, 1274.6199, 1282.5765, 1283.6177, 1298.6028, 1299.6059, 1301.6870, 1315.6103, 1318.6687, 1334.6603, 1380.7520, 1386.7403, 1402.7407, 1422.7041, 1424.7200, 1427.7243, 1462.8779, 1467.8310, 1612.8208, 1690.6642, 1706.9716, 1723.9839, 1815.9168, 2202.1952

19. Q7SD03_NEUCR **Mass:** 189062 **Score:** 57 **Expect:** 6.6 **Queries matched:** 20

Hypothetical protein NCU03060.1.- Neurospora crassa.

Observed	Mr(expt)	Mr(calc)	Delta	Start		End	Miss	Peptide
831.4419	830.4346	830.4359	-0.0013	138	-	144	0	R.SSRPTQR.R
1012.6155	1011.6083	1011.5461	0.0622	1618	-	1626	1	R.DIDRGAPIR.H
1157.6270	1156.6198	1156.5948	0.0249	1171	-	1180	1	R.AVDANNERLR.E
1224.6023	1223.5950	1223.6220	-0.0269	813	-	822	0	K.MLDILGDYLR.V + Oxidation (M)
1226.5773	1225.5700	1225.5947	-0.0247	1533	-	1542	1	R.LQRMFGDLCK.K + Oxidation (M)
1230.7044	1229.6971	1229.6615	0.0356	913	-	922	1	K.QTIEEEVVKR.A
1235.6798	1234.6726	1234.7172	-0.0447	1319	-	1329	0	R.SEYLLSVLLAK.H
1242.5818	1241.5745	1241.5896	-0.0151	1464	-	1473	1	K.ANMERIMYSK.E
1258.5811	1257.5738	1257.5845	-0.0107	1464	-	1473	1	K.ANMERIMYSK.E + Oxidation (M)
1274.6199	1273.6126	1273.5795	0.0332	1464	-	1473	1	K.ANMERIMYSK.E + 2 Oxidation (M)
1283.6177	1282.6105	1282.6162	-0.0057	1533	-	1542	1	R.LQRMFGDLCK.K + Carbamidomethyl (C); Oxidation (M)
1298.6028	1297.5955	1297.6989	-0.1035	1181	-	1192	1	R.EEEARAGKPLAK.K
1318.6687	1317.6615	1317.5684	0.0930	1099	-	1108	0	R.EEQEELEEQR.A
1402.7407	1401.7335	1401.6120	0.1214	659	-	670	1	K.GPTDEEQDEARR.I
1408.7587	1407.7515	1407.6590	0.0925	97	-	110	0	R.SAQSNEVVSASSSR.T
1422.7041	1421.6968	1421.7667	-0.0698	490	-	501	0	K.TVQSVSFLSWLR.N
1424.7200	1423.7128	1423.6983	0.0145	550	-	561	1	R.DYELFVDGNPKK.T
1612.8208	1611.8135	1611.8580	-0.0445	1357	-	1373	1	R.RGEASSISGSPAPLGVK.K
1815.9168	1814.9096	1814.8758	0.0337	1557	-	1572	1	K.KPSRQDLEDGEIASDR.D
2202.1952	2201.1880	2200.9847	0.2032	1276	-	1292	1	R.DDPELQMHDKFFLEEHR.V + Oxidation (M)

No match to: 712.2679, 781.4592, 825.1370, 832.3588, 833.3959, 841.0980, 852.4849, 853.5010, 861.0837, 873.4641, 876.3626, 877.0567, 1158.6512, 1213.7102, 1238.8028, 1240.5956, 1252.6795, 1257.6582, 1257.7440, 1282.5765, 1299.6059, 1301.6870, 1315.6103, 1334.6603, 1380.7520, 1386.7403, 1400.6846, 1427.7243, 1462.8779, 1467.8310, 1690.6642, 1706.9716, 1723.9839

20. Q5ZVU0_LEGPH **Mass:** 41536 **Score:** 56 **Expect:** 7.6 **Queries matched:** 9

L-lysine dehydrogenase.- Legionella pneumophila subsp. pneumophila (strain Philadelphia 1 / ATCC 33152 / DSM 7513).

Observed	Mr(expt)	Mr(calc)	Delta	Start		End	Miss	Peptide
832.3588	831.3515	831.4061	-0.0546	257	-	263	1	R.YPGHCKK.M
1224.6023	1223.5950	1223.6584	-0.0633	2	-	12	0	M.IICVGLSSFTK.E + Carbamidomethyl (C)
1242.5818	1241.5745	1241.6880	-0.1135	363	-	372	1	K.FKLSHVLENR.F
1298.6028	1297.5955	1297.6774	-0.0819	1	-	12	0	-.MIICVGLSSFTK.E
1299.6059	1298.5987	1298.7095	-0.1108	365	-	375	1	K.LSHVLENRFGK.Y
1402.7407	1401.7335	1401.6090	0.1244	15	-	27	0	K.DCMYNVMITGAGK.I
1424.7200	1423.7128	1423.7744	-0.0617	2	-	14	1	M.IICVGLSSFTKEK.D
1612.8208	1611.8135	1611.8364	-0.0229	1	-	14	1	-.MIICVGLSSFTKEK.D + Carbamidomethyl (C)
1723.9839	1722.9766	1723.0495	-0.0728	59	-	74	1	R.LLTALPEIKTVALDVK.D

No match to: 712.2679, 781.4592, 825.1370, 831.4419, 833.3959, 841.0980, 852.4849, 853.5010, 861.0837, 873.4641, 876.3626, 877.0567, 1012.6155, 1157.6270, 1158.6512, 1213.7102, 1226.5773, 1230.7044, 1235.6798, 1238.8028, 1240.5956, 1252.6795, 1257.6582, 1257.7440, 1258.5811, 1274.6199, 1282.5765, 1283.6177, 1301.6870, 1315.6103, 1318.6687, 1334.6603, 1380.7520, 1386.7403, 1400.6846, 1408.7587, 1422.7041, 1427.7243, 1462.8779, 1467.8310, 1690.6642, 1706.9716, 1815.9168, 2202.1952

21. B72212 **Mass:** 20862 **Score:** 56 **Expect:** 8.3 **Queries matched:** 7

translation elongation factor P - Thermotoga maritima (strain MSB8)

Observed	Mr(expt)	Mr(calc)	Delta	Start	End	Miss	Peptide
781.4592	780.4520	780.3766	0.0754	178 -	184	0	R.TGEYVGR.A
831.4419	830.4346	830.4974	-0.0628	33 -	40	1	R.GSGLIRTK.L
852.4849	851.4776	851.4137	0.0640	178 -	185	1	R.TGEYVGRA.-
1230.7044	1229.6971	1229.6451	0.0520	28 -	38	1	K.HFMGRGSGLIR.T
1252.6795	1251.6722	1251.6207	0.0515	174 -	184	1	K.VDTRTGEYVGR.A
1274.6199	1273.6126	1273.6601	-0.0474	22 -	32	1	R.VLEASKHFMGR.G
1380.7520	1379.7447	1379.7336	0.0111	160 -	171	0	K.ITVPYFIEVGDK.I

No match to: 712.2679, 825.1370, 832.3588, 833.3959, 841.0980, 853.5010, 861.0837, 873.4641, 876.3626, 877.0567, 1012.6155, 1157.6270, 1158.6512, 1213.7102, 1224.6023, 1226.5773, 1235.6798, 1238.8028, 1240.5956, 1242.5818, 1257.6582, 1257.7440, 1258.5811, 1282.5765, 1283.6177, 1298.6028, 1299.6059, 1301.6870, 1315.6103, 1318.6687, 1334.6603, 1386.7403, 1400.6846, 1402.7407, 1408.7587, 1422.7041, 1424.7200, 1427.7243, 1462.8779, 1467.8310, 1612.8208, 1690.6642, 1706.9716, 1723.9839, 1815.9168, 2202.1952

22. Q19Y36_9CAUD **Mass:** 12275 **Score:** 55 **Expect:** 11 **Queries matched:** 6

Gp24.- Mycobacteriophage Wildcat.

Observed	Mr(expt)	Mr(calc)	Delta	Start	End	Miss	Peptide
853.5010	852.4937	852.4857	0.0080	63 -	69	1	R.KLFNFGK.S
1226.5773	1225.5700	1225.6302	-0.0602	35 -	45	0	K.SSVANLELEHK.A
1235.6798	1234.6726	1234.6193	0.0532	70 -	80	0	K.SPVFTTEDLAR.A
1282.5765	1281.5692	1281.6465	-0.0773	83 -	92	1	R.VVYFERAQDR.L
1386.7403	1385.7330	1385.7554	-0.0224	5 -	17	0	K.AIVQLPDGSYVPK.H
1462.8779	1461.8706	1461.7576	0.1130	70 -	82	1	K.SPVFTTEDLARAR.V

No match to: 712.2679, 781.4592, 825.1370, 831.4419, 832.3588, 833.3959, 841.0980, 852.4849, 861.0837, 873.4641, 876.3626, 877.0567, 1012.6155, 1157.6270, 1158.6512, 1213.7102, 1224.6023, 1230.7044, 1238.8028, 1240.5956, 1242.5818, 1252.6795, 1257.6582, 1257.7440, 1258.5811, 1274.6199, 1283.6177, 1298.6028, 1299.6059, 1301.6870, 1315.6103, 1318.6687, 1334.6603, 1380.7520, 1400.6846, 1402.7407, 1408.7587, 1422.7041, 1424.7200, 1427.7243, 1467.8310, 1612.8208, 1690.6642, 1706.9716, 1723.9839, 1815.9168, 2202.1952

23. Q2WWQ7_9GAMM **Mass:** 12361 **Score:** 53 **Expect:** 15 **Queries matched:** 7

Hypothetical protein.- Shewanella sp. W3-18-1.

Observed	Mr(expt)	Mr(calc)	Delta	Start	End	Miss	Peptide
833.3959	832.3886	832.4225	-0.0339	32 -	38	1	R.NKQQMGK.Y
852.4849	851.4776	851.4977	-0.0201	65 -	71	1	K.KHQLAQK.E
853.5010	852.4937	852.4341	0.0596	42 -	48	0	K.SLDYSLR.R
1230.7044	1229.6971	1229.6364	0.0607	23 -	33	1	K.VSADLEQARNK.Q
1257.6582	1256.6509	1256.6765	-0.0255	39 -	48	1	K.YIKSLDYSLR.R
1257.7440	1256.7367	1256.6765	0.0602	39 -	48	1	K.YIKSLDYSLR.R
2202.1952	2201.1880	2201.0375	0.1504	2 -	21	0	M.TTDIDLQSAIAALDEYGYDK.K

No match to: 712.2679, 781.4592, 825.1370, 831.4419, 832.3588, 841.0980, 861.0837, 873.4641, 876.3626, 877.0567, 1012.6155, 1157.6270, 1158.6512, 1213.7102, 1224.6023, 1226.5773, 1235.6798, 1238.8028, 1240.5956, 1242.5818, 1252.6795, 1258.5811, 1274.6199, 1282.5765, 1283.6177, 1298.6028, 1299.6059, 1301.6870, 1315.6103, 1318.6687, 1334.6603, 1380.7520, 1386.7403, 1400.6846, 1402.7407, 1408.7587, 1422.7041, 1424.7200, 1427.7243, 1462.8779, 1467.8310, 1612.8208, 1690.6642, 1706.9716, 1723.9839, 1815.9168

24. Q2ZS56_SHEPU **Mass:** 12333 **Score:** 53 **Expect:** 15 **Queries matched:** 7

Hypothetical protein.- Shewanella putrefaciens CN-32.

Observed	Mr(expt)	Mr(calc)	Delta	Start	End	Miss	Peptide
833.3959	832.3886	832.4225	-0.0339	32 -	38	1	R.NKQQMGK.Y
852.4849	851.4776	851.4977	-0.0201	65 -	71	1	K.KHQLAQK.E
853.5010	852.4937	852.4341	0.0596	42 -	48	0	K.SLDYSLR.R
1230.7044	1229.6971	1229.6364	0.0607	23 -	33	1	K.VSADLEQARNK.Q
1257.6582	1256.6509	1256.6765	-0.0255	39 -	48	1	K.YIKSLDYSLR.R
1257.7440	1256.7367	1256.6765	0.0602	39 -	48	1	K.YIKSLDYSLR.R
2202.1952	2201.1880	2201.0375	0.1504	2 -	21	0	M.TTDIDLQSAIAALDEYGYDK.K

No match to: 712.2679, 781.4592, 825.1370, 831.4419, 832.3588, 841.0980, 861.0837, 873.4641, 876.3626, 877.0567, 1012.6155, 1157.6270, 1158.6512, 1213.7102, 1224.6023, 1226.5773, 1235.6798, 1238.8028, 1240.5956, 1242.5818, 1252.6795, 1258.5811, 1274.6199, 1282.5765, 1283.6177, 1298.6028, 1299.6059, 1301.6870, 1315.6103, 1318.6687, 1334.6603, 1380.7520, 1386.7403, 1400.6846, 1402.7407, 1408.7587, 1422.7041, 1424.7200, 1427.7243, 1462.8779, 1467.8310, 1612.8208, 1690.6642, 1706.9716, 1723.9839, 1815.9168

25. Q833F6_ENTFA **Mass:** 106075 **Score:** 53 **Expect:** 16 **Queries matched:** 13

Tape measure protein, putative.- Enterococcus faecalis (Streptococcus faecalis).

Observed	Mr(expt)	Mr(calc)	Delta	Start		End	Miss	Peptide
1230.7044	1229.6971	1229.7053	-0.0082	566	-	576	0	K.MIAENVVTILK.N
1235.6798	1234.6726	1234.7146	-0.0420	844	-	855	1	K.IKGALGIHSPSR.W
1252.6795	1251.6722	1251.6467	0.0255	548	-	558	1	K.NAFKMVVNAMK.A
1257.6582	1256.6509	1256.5495	0.1014	442	-	452	0	K.EAFSNAGTWMK.E + Oxidation (M)
1258.5811	1257.5738	1257.6353	-0.0615	753	-	763	0	K.DGIINGWENLK.Q
1274.6199	1273.6126	1273.7176	-0.1050	255	-	266	1	K.TGLANMKTAIVR.G
1315.6103	1314.6030	1314.6244	-0.0214	471	-	481	0	K.EFFSGLWNSTK.E
1386.7403	1385.7330	1385.7514	-0.0184	234	-	247	1	K.EGGGGLKSLEQIAK.D
1402.7407	1401.7335	1401.7463	-0.0128	128	-	141	0	K.GVQLIASSTNDLGK.S
1408.7587	1407.7515	1407.8166	-0.0651	654	-	664	0	K.LFFTLLWIDIK.Y
1422.7041	1421.6968	1421.7184	-0.0215	248	-	261	1	K.DSTAGIKTGLANMK.T + Oxidation (M)
1427.7243	1426.7170	1426.7204	-0.0034	453	-	465	1	K.EVPGNAADWVKNK.W
1467.8310	1466.8238	1466.7564	0.0673	846	-	858	1	K.GALGIHSPSRWMR.D

No match to: 712.2679, 781.4592, 825.1370, 831.4419, 832.3588, 833.3959, 841.0980, 852.4849, 853.5010, 861.0837, 873.4641, 876.3626, 877.0567, 1012.6155, 1157.6270, 1158.6512, 1213.7102, 1224.6023, 1226.5773, 1238.8028, 1240.5956, 1242.5818, 1257.7440, 1282.5765, 1283.6177, 1298.6028, 1299.6059, 1301.6870, 1318.6687, 1334.6603, 1380.7520, 1400.6846, 1424.7200, 1462.8779, 1612.8208, 1690.6642, 1706.9716, 1723.9839, 1815.9168, 2202.1952

26. AAL97589 **Mass:** 22480 **Score:** 53 **Expect:** 17 **Queries matched:** 7

AE010023 NID: - Streptococcus pyogenes MGAS8232

Observed	Mr(expt)	Mr(calc)	Delta	Start		End	Miss	Peptide
1157.6270	1156.6198	1156.5434	0.0764	101	-	109	0	K.MIEEYLTSR.D + Oxidation (M)
1158.6512	1157.6440	1157.6516	-0.0077	54	-	64	1	K.NLARTSSKPGK.T
1235.6798	1234.6726	1234.7033	-0.0308	43	-	53	1	K.SSFINTILGRK.N
1301.6870	1300.6797	1300.6009	0.0788	128	-	137	0	K.EDIQMYDFLK.Y
1380.7520	1379.7447	1379.7700	-0.0253	138	-	149	0	K.YYDIPVIVVATK.A
1386.7403	1385.7330	1385.6826	0.0503	174	-	185	0	K.SDTFIVFSSVER.I
1467.8310	1466.8238	1466.7405	0.0833	65	-	76	0	K.TQLLNFFNIDDK.L

No match to: 712.2679, 781.4592, 825.1370, 831.4419, 832.3588, 833.3959, 841.0980, 852.4849, 853.5010, 861.0837, 873.4641, 876.3626, 877.0567, 1012.6155, 1213.7102, 1224.6023, 1226.5773, 1230.7044, 1238.8028, 1240.5956, 1242.5818, 1252.6795, 1257.6582, 1257.7440, 1258.5811, 1274.6199, 1282.5765, 1283.6177, 1298.6028, 1299.6059, 1315.6103, 1318.6687, 1334.6603, 1400.6846, 1402.7407, 1408.7587, 1422.7041, 1424.7200, 1427.7243, 1462.8779, 1612.8208, 1690.6642, 1706.9716, 1723.9839, 1815.9168, 2202.1952

27. Q8I4X5_PLAF7 **Mass:** 188045 **Score:** 53 **Expect:** 17 **Queries matched:** 18

Hypothetical protein.- Plasmodium falciparum (isolate 3D7).

Observed	Mr(expt)	Mr(calc)	Delta	Start		End	Miss	Peptide
832.3588	831.3515	831.4086	-0.0571	1008	-	1014	1	K.DDVNKNK.N
833.3959	832.3886	832.3311	0.0575	234	-	240	0	K.DNNDNNK.D
853.5010	852.4937	852.4817	0.0120	223	-	229	1	K.INKVHDK.I
1012.6155	1011.6083	1011.5475	0.0608	1336	-	1343	1	K.HIRNHAHK.D
1157.6270	1156.6198	1156.5764	0.0434	1097	-	1106	0	K.STVFSNFDLK.E
1213.7102	1212.7029	1212.7077	-0.0048	756	-	765	1	K.IDIIKEQLNK.S
1226.5773	1225.5700	1225.6091	-0.0391	1138	-	1147	0	K.NLNSNYFINK.I
1242.5818	1241.5745	1241.6768	-0.1022	130	-	140	0	K.LVIDTFHVGNK.S
1252.6795	1251.6722	1251.6394	0.0329	916	-	925	1	K.FNLTSLKCNR.K + Carbamidomethyl (C)
1257.6582	1256.6509	1256.6724	-0.0215	1107	-	1116	1	K.ENLDRQIIEK.N
1257.7440	1256.7367	1256.6724	0.0643	1107	-	1116	1	K.ENLDRQIIEK.N
1283.6177	1282.6105	1282.5578	0.0527	1197	-	1206	0	K.EYNNTYNTHK.Y
1334.6603	1333.6530	1333.7354	-0.0823	1262	-	1272	1	K.EINNKSITFLR.Q
1380.7520	1379.7447	1379.7296	0.0151	615	-	626	0	K.LQYLNETASTIK.F
1408.7587	1407.7515	1407.6816	0.0699	74	-	84	0	K.NVEEYINVICR.I + Carbamidomethyl (C)
1422.7041	1421.6968	1421.6786	0.0182	1399	-	1410	0	K.ENTNDVNTFIQK.E
1467.8310	1466.8238	1466.7477	0.0761	821	-	832	1	K.KQENEEHALTIR.N
2202.1952	2201.1880	2201.0461	0.1419	975	-	993	1	K.INIPDDNNNNNNNNKISFK.S

No match to: 712.2679, 781.4592, 825.1370, 831.4419, 841.0980, 852.4849, 861.0837, 873.4641, 876.3626, 877.0567, 1158.6512, 1224.6023, 1230.7044, 1235.6798, 1238.8028, 1240.5956, 1258.5811, 1274.6199, 1282.5765, 1298.6028, 1299.6059, 1301.6870, 1315.6103, 1318.6687, 1386.7403, 1400.6846, 1402.7407, 1424.7200, 1427.7243, 1462.8779, 1612.8208, 1690.6642, 1706.9716, 1723.9839, 1815.9168

28. Q8K6Y4_STRP3 **Mass:** 28197 **Score:** 53 **Expect:** 17 **Queries matched:** 10

Putative 1-acylglycerol-3-phosphate O-acyltransferase.- Streptococcus pyogenes serotype M3.

Observed	Mr(expt)	Mr(calc)	Delta	Start		End	Miss	Peptide
832.3588	831.3515	831.4279	-0.0764	2	-	7	0	M.FYAYLR.G
1012.6155	1011.6083	1011.5575	0.0508	59	-	66	1	K.QFIFMAKK.E
1318.6687	1317.6615	1317.5983	0.0632	174	-	185	0	R.MNDEGIAEVANR.I
1334.6603	1333.6530	1333.5932	0.0599	174	-	185	0	R.MNDEGIAEVANR.I + Oxidation (M)
1408.7587	1407.7515	1407.6890	0.0625	138	-	150	0	K.IMPAAYQGPMSVK.G + Oxidation (M)
1422.7041	1421.6968	1421.7990	-0.1021	119	-	132	1	R.HSQEVKGGVAVIAK.M
1424.7200	1423.7128	1423.6839	0.0289	138	-	150	0	K.IMPAAYQGPMSVK.G + 2 Oxidation (M)
1467.8310	1466.8238	1466.7299	0.0938	106	-	118	1	K.SNRSLLMFPSGSR.H + Oxidation (M)
1723.9839	1722.9766	1722.9245	0.0521	67	-	79	1	K.ELFTNRLFAWWIK.M
1815.9168	1814.9096	1814.8814	0.0282	44	-	58	0	R.TFWDPVYMAFAARPK.Q + Oxidation (M)

No match to: 712.2679, 781.4592, 825.1370, 831.4419, 833.3959, 841.0980, 852.4849, 853.5010, 861.0837, 873.4641, 876.3626, 877.0567, 1157.6270, 1158.6512, 1213.7102, 1224.6023, 1226.5773, 1230.7044, 1235.6798, 1238.8028, 1240.5956, 1242.5818, 1252.6795, 1257.6582, 1257.7440, 1258.5811, 1274.6199, 1282.5765, 1283.6177, 1298.6028, 1299.6059, 1301.6870, 1315.6103, 1380.7520, 1386.7403, 1400.6846, 1402.7407, 1427.7243, 1462.8779, 1612.8208, 1690.6642, 1706.9716, 2202.1952

29. Q84QB8_ORYSA **Mass:** 42907 **Score:** 52 **Expect:** 19 **Queries matched:** 11

Putative receptor ser/thr protein.- Oryza sativa (japonica cultivar-group).

Observed	Mr(expt)	Mr(calc)	Delta	Start		End	Miss	Peptide
873.4641	872.4568	872.4967	-0.0399	184	-	191	0	K.ASNILLDK.K
1157.6270	1156.6198	1156.6175	0.0022	147	-	155	1	R.CIPLNWQKR.A
1224.6023	1223.5950	1223.4998	0.0953	54	-	63	0	R.NFHMMNCVGR.G + Oxidation (M)
1240.5956	1239.5884	1239.4947	0.0937	54	-	63	0	R.NFHMMNCVGR.G + 2 Oxidation (M)
1257.6582	1256.6509	1256.7088	-0.0579	72	-	83	1	K.GNLKDGTQIAIK.K
1257.7440	1256.7367	1256.7088	0.0279	72	-	83	1	K.GNLKDGTQIAIK.K
1258.5811	1257.5738	1257.6061	-0.0323	16	-	28	0	R.GNQNGSAAVTSPR.S
1301.6870	1300.6797	1300.6710	0.0087	145	-	154	1	R.SRCIPLNWQK.R + Carbamidomethyl (C)
1400.6846	1399.6774	1399.7460	-0.0686	205	-	216	0	K.LFPDTITHISTR.V
1706.9716	1705.9644	1705.7963	0.1681	50	-	63	1	K.LATRNFHMMNCVGR.G + Carbamidomethyl (C)
2202.1952	2201.1880	2201.1844	0.0036	197	-	216	1	K.IGDFGLAKLFPDTITHISTR.V

No match to: 712.2679, 781.4592, 825.1370, 831.4419, 832.3588, 833.3959, 841.0980, 852.4849, 853.5010, 861.0837, 876.3626, 877.0567, 1012.6155, 1158.6512, 1213.7102, 1226.5773, 1230.7044, 1235.6798, 1238.8028, 1242.5818, 1252.6795, 1274.6199, 1282.5765, 1283.6177, 1298.6028, 1299.6059, 1315.6103, 1318.6687, 1334.6603, 1380.7520, 1386.7403, 1402.7407, 1408.7587, 1422.7041, 1424.7200, 1427.7243, 1462.8779, 1467.8310, 1612.8208, 1690.6642, 1723.9839, 1815.9168

30. AAM79212 **Mass:** 22460 **Score:** 52 **Expect:** 21 **Queries matched:** 7

AE014074 NID: - Streptococcus pyogenes MGAS315

Observed	Mr(expt)	Mr(calc)	Delta	Start		End	Miss	Peptide
1157.6270	1156.6198	1156.5434	0.0764	101	-	109	0	K.MIEEYLTSR.D + Oxidation (M)
1158.6512	1157.6440	1157.6516	-0.0077	54	-	64	1	K.NLARTSSKPGK.T
1235.6798	1234.6726	1234.7033	-0.0308	43	-	53	1	K.SSFINTILGRK.N
1301.6870	1300.6797	1300.6009	0.0788	128	-	137	0	K.EDIQMYDFLK.Y
1380.7520	1379.7447	1379.7700	-0.0253	138	-	149	0	K.YYDIPVIVVATK.A
1386.7403	1385.7330	1385.6826	0.0503	174	-	185	0	K.SDTFIVFSSVER.I
1467.8310	1466.8238	1466.7405	0.0833	65	-	76	0	K.TQLLNFFNIDDK.L

No match to: 712.2679, 781.4592, 825.1370, 831.4419, 832.3588, 833.3959, 841.0980, 852.4849, 853.5010, 861.0837, 873.4641, 876.3626, 877.0567, 1012.6155, 1213.7102, 1224.6023, 1226.5773, 1230.7044, 1238.8028, 1240.5956, 1242.5818, 1252.6795, 1257.6582, 1257.7440, 1258.5811, 1274.6199, 1282.5765, 1283.6177, 1298.6028, 1299.6059, 1315.6103, 1318.6687, 1334.6603, 1400.6846, 1402.7407, 1408.7587, 1422.7041, 1424.7200, 1427.7243, 1462.8779, 1612.8208, 1690.6642, 1706.9716, 1723.9839, 1815.9168, 2202.1952

31. AAN72830 **Mass:** 253967 **Score:** 52 **Expect:** 22 **Queries matched:** 21

AY157993 NID: - Tomato black ring virus

Observed	Mr(expt)	Mr(calc)	Delta	Start		End	Miss	Peptide
832.3588	831.3515	831.4095	-0.0580	2205	-	2211	0	K.VPCMVAR.Q + Carbamidomethyl (C)
852.4849	851.4776	851.4137	0.0639	19	-	25	0	K.YNNSLNK.Y
853.5010	852.4937	852.4705	0.0232	753	-	759	0	K.DLVELHK.R
873.4641	872.4568	872.5153	-0.0585	1416	-	1424	0	R.VVGMLVAGK.D
1012.6155	1011.6083	1011.5396	0.0687	1269	-	1276	1	R.MTRHQALR.F
1224.6023	1223.5950	1223.5063	0.0887	2010	-	2019	0	R.FMHMSEQGNK.A + Oxidation (M)
1240.5956	1239.5884	1239.6459	-0.0575	1495	-	1505	1	K.EPAVLTKDDPR.C
1242.5818	1241.5745	1241.6438	-0.0692	1476	-	1486	0	K.TNMVPVPESLR.V

1258.5811	1257.5738	1257.6387	-0.0649	1476 - 1486	0	K.TNMVPVPESLR.V + Oxidation (M)
1274.6199	1273.6126	1273.6423	-0.0297	2205 - 2214	1	K.VPCMVARQWK.S + Carbamidomethyl (C)
1282.5765	1281.5692	1281.6573	-0.0880	419 - 430	1	R.ANVFMKAMAGVK.N + Oxidation (M)
1298.6028	1297.5955	1297.6522	-0.0567	419 - 430	1	R.ANVFMKAMAGVK.N + 2 Oxidation (M)
1301.6870	1300.6797	1300.6735	0.0062	127 - 138	1	R.REVAEASAEALR.L
1318.6687	1317.6615	1317.6104	0.0511	639 - 650	1	K.IGAACHSMRMGK.E + Carbamidomethyl (C)
1334.6603	1333.6530	1333.6448	0.0082	1689 - 1700	0	R.LPCQVGTNPYSR.E
1380.7520	1379.7447	1379.6074	0.1373	2009 - 2019	1	K.RFMHMSEQGNK.A + Oxidation (M)
1386.7403	1385.7330	1385.7336	-0.0006	1475 - 1486	1	K.KTNMVPVPESLR.V + Oxidation (M)
1427.7243	1426.7170	1426.6762	0.0408	788 - 800	0	K.SNFMSTLDNALAK.H + Oxidation (M)
1462.8779	1461.8706	1461.7252	0.1454	50 - 61	1	K.KYNVLSSFFSDR.V
1723.9839	1722.9766	1722.8664	0.1103	771 - 783	1	R.RCEPVWIYLFGQR.H + Carbamidomethyl (C)
1815.9168	1814.9096	1814.8747	0.0349	1675 - 1688	1	K.TCAFTQFLQHNRHR.L + Carbamidomethyl (C)

No match to: 712.2679, 781.4592, 825.1370, 831.4419, 833.3959, 841.0980, 861.0837, 876.3626, 877.0567, 1157.6270, 1158.6512, 1213.7102, 1226.5773, 1230.7044, 1235.6798, 1238.8028, 1252.6795, 1257.6582, 1257.7440, 1283.6177, 1299.6059, 1315.6103, 1400.6846, 1402.7407, 1408.7587, 1422.7041, 1424.7200, 1467.8310, 1612.8208, 1690.6642, 1706.9716, 2202.1952

32. AAK33806 **Mass:** 22479 **Score:** 51 **Expect:** 23 **Queries matched:** 7

AE006538 NID: - Streptococcus pyogenes M1 GAS

Observed	Mr(expt)	Mr(calc)	Delta	Start - End	Miss	Peptide
1157.6270	1156.6198	1156.5434	0.0764	101 - 109	0	K.MIEEYLTSR.D + Oxidation (M)
1158.6512	1157.6440	1157.6516	-0.0077	54 - 64	1	K.NLARTSSKPGK.T
1235.6798	1234.6726	1234.7033	-0.0308	43 - 53	1	K.SSFINTILGRK.N
1301.6870	1300.6797	1300.6009	0.0788	128 - 137	0	K.EDIQMYDFLK.Y
1380.7520	1379.7447	1379.7700	-0.0253	138 - 149	0	K.YYDIPVIVVATK.A
1386.7403	1385.7330	1385.6826	0.0503	174 - 185	0	K.SDTFIVFSSVER.I
1467.8310	1466.8238	1466.7405	0.0833	65 - 76	0	K.TQLLNFFNIDDK.L

No match to: 712.2679, 781.4592, 825.1370, 831.4419, 832.3588, 833.3959, 841.0980, 852.4849, 853.5010, 861.0837, 873.4641, 876.3626, 877.0567, 1012.6155, 1213.7102, 1224.6023, 1226.5773, 1230.7044, 1238.8028, 1240.5956, 1242.5818, 1252.6795, 1257.6582, 1257.7440, 1258.5811, 1274.6199, 1282.5765, 1283.6177, 1298.6028, 1299.6059, 1315.6103, 1318.6687, 1334.6603, 1400.6846, 1402.7407, 1408.7587, 1422.7041, 1424.7200, 1427.7243, 1462.8779, 1612.8208, 1690.6642, 1706.9716, 1723.9839, 1815.9168, 2202.1952

33. Q8T530_PLAFA **Mass:** 14687 **Score:** 51 **Expect:** 25 **Queries matched:** 6

Erythrocyte membrane protein 1 (Fragment).- Plasmodium falciparum.

Observed	Mr(expt)	Mr(calc)	Delta	Start - End	Miss	Peptide
831.4419	830.4346	830.4498	-0.0152	25 - 31	0	K.QLQDSLK.N
1012.6155	1011.6083	1011.5097	0.0985	37 - 45	0	K.IHSEVTNGR.T
1282.5765	1281.5692	1281.6717	-0.1024	8 - 18	1	R.GKDLFIGYNQK.D
1298.6028	1297.5955	1297.6738	-0.0783	37 - 48	1	K.IHSEVTNGRTGK.N
1301.6870	1300.6797	1300.5684	0.1113	58 - 68	0	K.GDTDGNYYQLR.E
1427.7243	1426.7170	1426.6510	0.0660	82 - 95	1	K.AITCGAGEGDRYSK.Y

No match to: 712.2679, 781.4592, 825.1370, 832.3588, 833.3959, 841.0980, 852.4849, 853.5010, 861.0837, 873.4641, 876.3626, 877.0567, 1157.6270, 1158.6512, 1213.7102, 1224.6023, 1226.5773, 1230.7044, 1235.6798, 1238.8028, 1240.5956, 1242.5818, 1252.6795, 1257.6582, 1257.7440, 1258.5811, 1274.6199, 1283.6177, 1299.6059, 1315.6103, 1318.6687, 1334.6603, 1380.7520, 1386.7403, 1400.6846, 1402.7407, 1408.7587, 1422.7041, 1424.7200, 1462.8779, 1467.8310, 1612.8208, 1690.6642, 1706.9716, 1723.9839, 1815.9168, 2202.1952

34. Q8GJA6_CAMJE **Mass:** 53327 **Score:** 51 **Expect:** 26 **Queries matched:** 10

Hypothetical protein Cjp49.- Campylobacter jejuni.

Observed	Mr(expt)	Mr(calc)	Delta	Start - End	Miss	Peptide
1230.7044	1229.6971	1229.6404	0.0567	107 - 116	1	K.IHFNTKDDIK.D
1235.6798	1234.6726	1234.6227	0.0498	320 - 330	0	K.NTMEANTILTK.L
1283.6177	1282.6105	1282.6293	-0.0188	156 - 166	0	K.STLYLDDIDTK.K
1299.6059	1298.5987	1298.6441	-0.0454	278 - 287	1	K.NLKNFVDFMR.K + Oxidation (M)
1318.6687	1317.6615	1317.6962	-0.0348	168 - 178	0	K.DLSDMILNIIR.K + Oxidation (M)
1400.6846	1399.6774	1399.7558	-0.0784	191 - 202	0	K.IDILNNESLELK.Q
1422.7041	1421.6968	1421.6575	0.0393	113 - 123	1	K.DDIKDFWNDVR.S
1427.7243	1426.7170	1426.7667	-0.0497	52 - 63	1	K.ENPTEKNLEILK.K
1612.8208	1611.8135	1611.6987	0.1148	29 - 41	1	K.DWKMDFSNQENAK.D
1706.9716	1705.9644	1705.9402	0.0241	400 - 413	1	K.NKIPLNAYIIQEYK.K

No match to: 712.2679, 781.4592, 825.1370, 831.4419, 832.3588, 833.3959, 841.0980, 852.4849, 853.5010, 861.0837, 873.4641, 876.3626, 877.0567, 1012.6155, 1157.6270, 1158.6512, 1213.7102, 1224.6023, 1226.5773, 1238.8028, 1240.5956, 1242.5818, 1252.6795, 1257.6582, 1257.7440, 1258.5811, 1274.6199, 1282.5765, 1298.6028, 1301.6870, 1315.6103, 1334.6603, 1380.7520, 1386.7403, 1402.7407, 1408.7587, 1424.7200, 1462.8779, 1467.8310, 1690.6642, 1723.9839, 1815.9168, 2202.1952

35. Q2YVH6_STAAB **Mass:** 9035 **Score:** 51 **Expect:** 26 **Queries matched:** 7

Hypothetical protein.- Staphylococcus aureus (strain bovine RF122).

Observed	Mr(expt)	Mr(calc)	Delta	Start	End	Miss	Peptide
832.3588	831.3515	831.4252	-0.0737	2 -	7	0	M.HHHFVR.C
1224.6023	1223.5950	1223.6081	-0.0130	51 -	60	1	R.DCISTFLRNR.L
1334.6603	1333.6530	1333.6826	-0.0295	2 -	11	1	M.HHHFVRCLTK.Y + Carbamidomethyl (C)
1380.7520	1379.7447	1379.7812	-0.0365	61 -	72	0	R.LQLQIGGYYVVK.L
1402.7407	1401.7335	1401.6711	0.0624	48 -	58	1	K.YERDCISTFLR.N
1408.7587	1407.7515	1407.7016	0.0499	1 -	11	1	-.MHHHFVRCLTK.Y
1424.7200	1423.7128	1423.6965	0.0163	1 -	11	1	-.MHHHFVRCLTK.Y + Oxidation (M)

No match to: 712.2679, 781.4592, 825.1370, 831.4419, 833.3959, 841.0980, 852.4849, 853.5010, 861.0837, 873.4641, 876.3626, 877.0567, 1012.6155, 1157.6270, 1158.6512, 1213.7102, 1226.5773, 1230.7044, 1235.6798, 1238.8028, 1240.5956, 1242.5818, 1252.6795, 1257.6582, 1257.7440, 1258.5811, 1274.6199, 1282.5765, 1283.6177, 1298.6028, 1299.6059, 1301.6870, 1315.6103, 1318.6687, 1386.7403, 1400.6846, 1422.7041, 1427.7243, 1462.8779, 1467.8310, 1612.8208, 1690.6642, 1706.9716, 1723.9839, 1815.9168, 2202.1952

36. Q9AH95_STRPN **Mass:** 24847 **Score:** 51 **Expect:** 26 **Queries matched:** 7

Wze (Tyrosine-protein kinase Wze) (EC 2.7.1.112).- Streptococcus pneumoniae.

Observed	Mr(expt)	Mr(calc)	Delta	Start	End	Miss	Peptide
1158.6512	1157.6440	1157.6478	-0.0038	1 -	10	1	-.MPTLEIAQKK.L
1252.6795	1251.6722	1251.5917	0.0805	134 -	143	0	K.NFNDMIETLR.K
1299.6059	1298.5987	1298.5567	0.0420	215 -	226	0	K.YGAYGSYGNYGK.K
1380.7520	1379.7447	1379.6867	0.0580	134 -	144	1	K.NFNDMIETLRK.Y
1422.7041	1421.6968	1421.7514	-0.0546	63 -	75	1	R.AGYKTLLIDGDTR.Q
1427.7243	1426.7170	1426.6516	0.0654	215 -	227	1	K.YGAYGSYGNYGKK.-
1467.8310	1466.8238	1466.7518	0.0720	50 -	62	0	K.TTTSINIAWSFAR.A

No match to: 712.2679, 781.4592, 825.1370, 831.4419, 832.3588, 833.3959, 841.0980, 852.4849, 853.5010, 861.0837, 873.4641, 876.3626, 877.0567, 1012.6155, 1157.6270, 1213.7102, 1224.6023, 1226.5773, 1230.7044, 1235.6798, 1238.8028, 1240.5956, 1242.5818, 1257.6582, 1257.7440, 1258.5811, 1274.6199, 1282.5765, 1283.6177, 1298.6028, 1301.6870, 1315.6103, 1318.6687, 1334.6603, 1386.7403, 1400.6846, 1402.7407, 1408.7587, 1424.7200, 1462.8779, 1612.8208, 1690.6642, 1706.9716, 1723.9839, 1815.9168, 2202.1952

37. Q1RUA7_MEDTR **Mass:** 7463 **Score:** 51 **Expect:** 26 **Queries matched:** 5

Hypothetical protein.- Medicago truncatula (Barrel medic).

Observed	Mr(expt)	Mr(calc)	Delta	Start	End	Miss	Peptide
852.4849	851.4776	851.4211	0.0565	12 -	18	0	K.LPTCQYK.C
873.4641	872.4568	872.4286	0.0282	19 -	26	0	K.CGSTPRPR.H
1252.6795	1251.6722	1251.6897	-0.0174	1 -	11	0	-.MLDIPPLNVPK.L + Oxidation (M)
1318.6687	1317.6615	1317.6888	-0.0274	47 -	58	0	R.QTVSATSELLNR.C
1706.9716	1705.9644	1705.8392	0.1252	12 -	26	1	K.LPTCQYKCGSTPRPR.H

No match to: 712.2679, 781.4592, 825.1370, 831.4419, 832.3588, 833.3959, 841.0980, 853.5010, 861.0837, 876.3626, 877.0567, 1012.6155, 1157.6270, 1158.6512, 1213.7102, 1224.6023, 1226.5773, 1230.7044, 1235.6798, 1238.8028, 1240.5956, 1242.5818, 1257.6582, 1257.7440, 1258.5811, 1274.6199, 1282.5765, 1283.6177, 1298.6028, 1299.6059, 1301.6870, 1315.6103, 1334.6603, 1380.7520, 1386.7403, 1400.6846, 1402.7407, 1408.7587, 1422.7041, 1424.7200, 1427.7243, 1462.8779, 1467.8310, 1612.8208, 1690.6642, 1723.9839, 1815.9168, 2202.1952

38. Q54RC0_DICDI **Mass:** 45292 **Score:** 51 **Expect:** 28 **Queries matched:** 9

Hypothetical protein.- Dictyostelium discoideum AX4.

Observed	Mr(expt)	Mr(calc)	Delta	Start	End	Miss	Peptide
781.4592	780.4520	780.4718	-0.0199	86 -	91	1	K.KPRQPR.Q
831.4419	830.4346	830.4035	0.0311	320 -	327	0	R.NPFGGSPR.N
876.3626	875.3553	875.3621	-0.0067	123 -	130	0	R.GEDSPSER.D
1224.6023	1223.5950	1223.5279	0.0672	273 -	283	1	R.NSGDRDQGGYR.G
1226.5773	1225.5700	1225.5587	0.0112	160 -	170	1	R.EGGFNRGGYNR.G
1252.6795	1251.6722	1251.5592	0.1130	278 -	288	1	R.DQGGYRGTQDR.E
1299.6059	1298.5987	1298.5751	0.0235	215 -	226	1	R.EGGYNRGGYGNR.D
1422.7041	1421.6968	1421.6422	0.0546	356 -	368	0	R.NPSEDVSTNAFNK.Q
1706.9716	1705.9644	1705.8019	0.1624	356 -	370	1	R.NPSEDVSTNAFNKQR.K

No match to: 712.2679, 825.1370, 832.3588, 833.3959, 841.0980, 852.4849, 853.5010, 861.0837, 873.4641, 877.0567, 1012.6155, 1157.6270, 1158.6512, 1213.7102, 1230.7044, 1235.6798, 1238.8028, 1240.5956, 1242.5818, 1257.6582, 1257.7440, 1258.5811, 1274.6199, 1282.5765, 1283.6177, 1298.6028, 1301.6870, 1315.6103, 1318.6687, 1334.6603, 1380.7520, 1386.7403, 1400.6846, 1402.7407, 1408.7587, 1424.7200, 1427.7243, 1462.8779, 1467.8310, 1612.8208, 1690.6642, 1723.9839, 1815.9168, 2202.1952

39. Q8D787_VIBVU **Mass:** 11586 **Score:** 51 **Expect:** 28 **Queries matched:** 6

Hypothetical protein.- Vibrio vulnificus.

Observed	Mr(expt)	Mr(calc)	Delta	Start		End	Miss	Peptide
831.4419	830.4346	830.4432	-0.0086	68	-	75	0	R.AAIGICQR.A
1318.6687	1317.6615	1317.6711	-0.0096	1	-	11	0	-.MILENKPNTSR.Q + Oxidation (M)
1334.6603	1333.6530	1333.7201	-0.0671	38	-	50	1	K.AQTNSTVTISGKK.D
1386.7403	1385.7330	1385.6074	0.1256	21	-	30	0	K.QCDFIYFHEK.T + Carbamidomethyl (C)
1400.6846	1399.6774	1399.7605	-0.0831	68	-	80	1	R.AAIGICQRALQEK.D
1723.9839	1722.9766	1722.8610	0.1156	76	-	90	1	R.ALQEKDFTVDASLMR.I

No match to: 712.2679, 781.4592, 825.1370, 832.3588, 833.3959, 841.0980, 852.4849, 853.5010, 861.0837, 873.4641, 876.3626, 877.0567, 1012.6155, 1157.6270, 1158.6512, 1213.7102, 1224.6023, 1226.5773, 1230.7044, 1235.6798, 1238.8028, 1240.5956, 1242.5818, 1252.6795, 1257.6582, 1257.7440, 1258.5811, 1274.6199, 1282.5765, 1283.6177, 1298.6028, 1299.6059, 1301.6870, 1315.6103, 1380.7520, 1402.7407, 1408.7587, 1422.7041, 1424.7200, 1427.7243, 1462.8779, 1467.8310, 1612.8208, 1690.6642, 1706.9716, 1815.9168, 2202.1952

40. O88180_MOUSE **Mass:** 65724 **Score:** 50 **Expect:** 29 **Queries matched:** 12

Guanine Nucleotide Regulatory Protein.- Mus musculus (Mouse).

Observed	Mr(expt)	Mr(calc)	Delta	Start		End	Miss	Peptide
1226.5773	1225.5700	1225.6376	-0.0676	408	-	418	1	K.YKDMGTVVLGK.L + Oxidation (M)
1230.7044	1229.6971	1229.5750	0.1221	75	-	85	0	R.MEWGAPVEPSK.D
1235.6798	1234.6726	1234.6458	0.0267	251	-	261	0	K.HFTILDAPGHK.S
1240.5956	1239.5884	1239.6169	-0.0285	557	-	567	0	R.TAGTICLETFK.D + Carbamidomethyl (C)
1258.5811	1257.5738	1257.6612	-0.0874	547	-	556	1	K.QDQVCIARLR.T + Carbamidomethyl (C)
1298.6028	1297.5955	1297.6561	-0.0606	295	-	306	1	K.GGQTREHAMLAK.T
1315.6103	1314.6030	1314.6527	-0.0497	2	-	14	1	M.AAAEAAEAEAQRK.H
1318.6687	1317.6615	1317.5983	0.0632	1	-	13	0	-.MAAAEAAEAEAQR.K
1334.6603	1333.6530	1333.5932	0.0599	1	-	13	0	-.MAAAEAAEAEAQR.K + Oxidation (M)
1386.7403	1385.7330	1385.6761	0.0569	74	-	85	1	K.RMEWGAPVEPSK.D
1402.7407	1401.7335	1401.6710	0.0624	74	-	85	1	K.RMEWGAPVEPSK.D + Oxidation (M)
1422.7041	1421.6968	1421.8242	-0.1273	395	-	407	1	R.SIDGPIRLPIVDK.Y

No match to: 712.2679, 781.4592, 825.1370, 831.4419, 832.3588, 833.3959, 841.0980, 852.4849, 853.5010, 861.0837, 873.4641, 876.3626, 877.0567, 1012.6155, 1157.6270, 1158.6512, 1213.7102, 1224.6023, 1238.8028, 1242.5818, 1252.6795, 1257.6582, 1257.7440, 1274.6199, 1282.5765, 1283.6177, 1299.6059, 1301.6870, 1380.7520, 1400.6846, 1408.7587, 1424.7200, 1427.7243, 1462.8779, 1467.8310, 1612.8208, 1690.6642, 1706.9716, 1723.9839, 1815.9168, 2202.1952

41. Q9FG41_ARATH **Mass:** 73235 **Score:** 50 **Expect:** 32 **Queries matched:** 12

Arabidopsis thaliana genomic DNA, chromosome 5, BAC clone:T25O11.- Arabidopsis thaliana (Mouse-ear cress).

Observed	Mr(expt)	Mr(calc)	Delta	Start		End	Miss	Peptide
831.4419	830.4346	830.4385	-0.0039	542	-	548	0	R.DLEDIVK.K
1158.6512	1157.6440	1157.5717	0.0723	263	-	271	1	R.DKNLYNYTK.V
1224.6023	1223.5950	1223.6583	-0.0633	398	-	408	1	R.ISDSMFKVLGK.V
1230.7044	1229.6971	1229.7053	-0.0082	384	-	394	0	R.ELQALLISCLK.Q
1240.5956	1239.5884	1239.5917	-0.0034	395	-	404	1	K.QERISDSMFK.V
1298.6028	1297.5955	1297.6778	-0.0823	265	-	274	1	K.NLYNYTKVQR.H
1301.6870	1300.6797	1300.7000	-0.0203	64	-	74	1	K.LYRHSSNGVIR.F
1334.6603	1333.6530	1333.7241	-0.0711	286	-	296	1	K.DFVIESKEIVR.K
1402.7407	1401.7335	1401.7649	-0.0315	22	-	33	1	K.GLEKIVEQLSMR.Q
1408.7587	1407.7515	1407.7721	-0.0207	13	-	25	1	R.EVLATPSHKGLEK.I
1424.7200	1423.7128	1423.6799	0.0329	1	-	12	1	-.MEESSNLMLKAR.E + Oxidation (M)
1690.6642	1689.6569	1689.7780	-0.1211	321	-	335	1	K.DDGAEYVRGLCDHLK.T

No match to: 712.2679, 781.4592, 825.1370, 832.3588, 833.3959, 841.0980, 852.4849, 853.5010, 861.0837, 873.4641, 876.3626, 877.0567, 1012.6155, 1157.6270, 1213.7102, 1226.5773, 1235.6798, 1238.8028, 1242.5818, 1252.6795, 1257.6582, 1257.7440, 1258.5811, 1274.6199, 1282.5765, 1283.6177, 1299.6059, 1315.6103, 1318.6687, 1380.7520, 1386.7403, 1400.6846, 1422.7041, 1427.7243, 1462.8779, 1467.8310, 1612.8208, 1706.9716, 1723.9839, 1815.9168, 2202.1952

42. Q1IF78_9PSED **Mass:** 98036 **Score:** 50 **Expect:** 33 **Queries matched:** 12

Hypothetical protein.- Pseudomonas entomophila L48.

Observed	Mr(expt)	Mr(calc)	Delta	Start		End	Miss	Peptide

832.3588	831.3515	831.4086	-0.0571	157 - 163	1	R.VDKEEGR.K
833.3959	832.3886	832.4265	-0.0379	401 - 407	0	R.MPYAVPR.R
1158.6512	1157.6440	1157.6842	-0.0402	690 - 699	1	K.LGEQLMLVKK.Q
1230.7044	1229.6971	1229.7165	-0.0194	145 - 156	0	K.VCVVISTVAALR.V
1235.6798	1234.6726	1234.6921	-0.0195	816 - 825	1	K.DSRLLIVEYK.G
1282.5765	1281.5692	1281.6750	-0.1058	849 - 860	1	K.SAGKGLYLMAQK.K + Oxidation (M)
1298.6028	1297.5955	1297.6150	-0.0195	324 - 334	0	R.HLIDTDNVDEK.A
1334.6603	1333.6530	1333.6448	0.0082	100 - 110	0	K.MQTLEAFQNPR.H
1422.7041	1421.6968	1421.7514	-0.0546	12 - 24	1	R.ALGSLEKYLDASR.L
1427.7243	1426.7170	1426.7779	-0.0609	867 - 879	1	R.GVREQLLAVIENS.-
1690.6642	1689.6569	1689.8065	-0.1497	562 - 575	0	R.LCVMEQGELELVDR.G + Carbamidomethyl (C)
1706.9716	1705.9644	1705.9879	-0.0235	673 - 687	1	R.GFSLSQLLKGQFILR.R

No match to: 712.2679, 781.4592, 825.1370, 831.4419, 841.0980, 852.4849, 853.5010, 861.0837, 873.4641, 876.3626, 877.0567, 1012.6155, 1157.6270, 1213.7102, 1224.6023, 1226.5773, 1238.8028, 1240.5956, 1242.5818, 1252.6795, 1257.6582, 1257.7440, 1258.5811, 1274.6199, 1283.6177, 1299.6059, 1301.6870, 1315.6103, 1318.6687, 1380.7520, 1386.7403, 1400.6846, 1402.7407, 1408.7587, 1424.7200, 1462.8779, 1467.8310, 1612.8208, 1723.9839, 1815.9168, 2202.1952

43. Q4YWP1_PLABE **Mass:** 30166 **Score:** 50 **Expect:** 34 **Queries matched:** 9

Hypothetical protein (Fragment).- Plasmodium berghei.

Observed	Mr(expt)	Mr(calc)	Delta	Start - End	Miss	Peptide
831.4419	830.4346	830.4861	-0.0516	221 - 227	1	K.KDTNLLK.S
832.3588	831.3515	831.4160	-0.0645	231 - 237	0	K.MNPITEK.K
1274.6199	1273.6126	1273.6012	0.0114	202 - 211	0	K.YISYVNDLCK.M + Carbamidomethyl (C)
1282.5765	1281.5692	1281.6573	-0.0881	1 - 11	1	-.KIYANCLICGK.L + Carbamidomethyl (C)
1334.6603	1333.6530	1333.7241	-0.0711	156 - 166	1	K.INENVDYVKIK.E
1400.6846	1399.6774	1399.6772	0.0002	65 - 75	0	K.YYFDPLNNNLK.K
1402.7407	1401.7335	1401.6962	0.0373	201 - 211	1	K.KYISYVNDLCK.M + Carbamidomethyl (C)
1422.7041	1421.6968	1421.6820	0.0148	84 - 95	1	K.MLKNSVNEENTK.I + Oxidation (M)
1424.7200	1423.7128	1423.6255	0.0872	167 - 177	0	K.EFSEYLNEHEK.K

No match to: 712.2679, 781.4592, 825.1370, 833.3959, 841.0980, 852.4849, 853.5010, 861.0837, 873.4641, 876.3626, 877.0567, 1012.6155, 1157.6270, 1158.6512, 1213.7102, 1224.6023, 1226.5773, 1230.7044, 1235.6798, 1238.8028, 1240.5956, 1242.5818, 1252.6795, 1257.6582, 1257.7440, 1258.5811, 1283.6177, 1298.6028, 1299.6059, 1301.6870, 1315.6103, 1318.6687, 1380.7520, 1386.7403, 1408.7587, 1427.7243, 1462.8779, 1467.8310, 1612.8208, 1690.6642, 1706.9716, 1723.9839, 1815.9168, 2202.1952

44. T35704 **Mass:** 16760 **Score:** 50 **Expect:** 34 **Queries matched:** 8

peptidylprolyl isomerase (EC 5.2.1.8) SC7H1.09 [similarity] - Streptomyces coelicolor

Observed	Mr(expt)	Mr(calc)	Delta	Start - End	Miss	Peptide
832.3588	831.3515	831.4272	-0.0757	1 - 7	0	-.MAENVLR.G
873.4641	872.4568	872.4716	-0.0147	131 - 138	0	R.VTIAADQR.S
1158.6512	1157.6440	1157.6305	0.0135	35 - 45	1	R.VPGFVVRGGDR.L
1257.6582	1256.6509	1256.6837	-0.0328	128 - 138	1	R.LDRVTIAADQR.S
1257.7440	1256.7367	1256.6837	0.0530	128 - 138	1	R.LDRVTIAADQR.S
1386.7403	1385.7330	1385.7377	-0.0047	144 - 157	0	R.ALWPGGGTAMVTLI.-
1402.7407	1401.7335	1401.7326	0.0009	144 - 157	0	R.ALWPGGGTAMVTLI.- + Oxidation (M)
1408.7587	1407.7515	1407.6465	0.1049	23 - 34	1	R.GGFCDGTVFHRR.V + Carbamidomethyl (C)

No match to: 712.2679, 781.4592, 825.1370, 831.4419, 833.3959, 841.0980, 852.4849, 853.5010, 861.0837, 876.3626, 877.0567, 1012.6155, 1157.6270, 1213.7102, 1224.6023, 1226.5773, 1230.7044, 1235.6798, 1238.8028, 1240.5956, 1242.5818, 1252.6795, 1258.5811, 1274.6199, 1282.5765, 1283.6177, 1298.6028, 1299.6059, 1301.6870, 1315.6103, 1318.6687, 1334.6603, 1380.7520, 1400.6846, 1422.7041, 1424.7200, 1427.7243, 1462.8779, 1467.8310, 1612.8208, 1690.6642, 1706.9716, 1723.9839, 1815.9168, 2202.1952

45. Q57U85_9TRYP **Mass:** 49924 **Score:** 50 **Expect:** 36 **Queries matched:** 9

Orotidine-5-phosphate decarboxylase/orotate phosphoribosyltransferase, putative (EC 2.4.2.10) (EC 4.1.1.23).- Trypanosoma brucei.

Observed	Mr(expt)	Mr(calc)	Delta	Start - End	Miss	Peptide
1224.6023	1223.5950	1223.5670	0.0281	88 - 99	0	R.GDIADTAEAYAK.S
1230.7044	1229.6971	1229.6478	0.0493	127 - 137	1	K.YSSKGVFVLCK.T
1235.6798	1234.6726	1234.7332	-0.0606	294 - 303	1	R.RLVTHPLIMR.L
1252.6795	1251.6722	1251.6645	0.0077	131 - 141	1	K.GVFVLCKTSNK.G + Carbamidomethyl (C)
1258.5811	1257.5738	1257.6751	-0.1013	15 - 25	0	K.TTLLCIGLDPR.A + Carbamidomethyl (C)
1318.6687	1317.6615	1317.6710	-0.0096	26 - 38	1	R.ADTAAAAVLECKR.I
1334.6603	1333.6530	1333.6626	-0.0095	357 - 368	1	K.GLIEGDFQKGDR.V
1380.7520	1379.7447	1379.6681	0.0767	87 - 99	1	K.RGDIADTAEAYAK.S

1400.6846	1399.6774	1399.7857	-0.1083	13	-	25	1	R.AKTTLLCIGLDPR.A

No match to: 712.2679, 781.4592, 825.1370, 831.4419, 832.3588, 833.3959, 841.0980, 852.4849, 853.5010, 861.0837, 873.4641, 876.3626, 877.0567, 1012.6155, 1157.6270, 1158.6512, 1213.7102, 1226.5773, 1238.8028, 1240.5956, 1242.5818, 1257.6582, 1257.7440, 1274.6199, 1282.5765, 1283.6177, 1298.6028, 1299.6059, 1301.6870, 1315.6103, 1386.7403, 1402.7407, 1408.7587, 1422.7041, 1424.7200, 1427.7243, 1462.8779, 1467.8310, 1612.8208, 1690.6642, 1706.9716, 1723.9839, 1815.9168, 2202.1952

46. Q4JCP4_SULAC **Mass:** 27674 **Score:** 49 **Expect:** 37 **Queries matched:** 8

Conserved protein.- Sulfolobus acidocaldarius.

Observed	Mr(expt)	Mr(calc)	Delta	Start		End	Miss	Peptide
833.3959	832.3886	832.4403	-0.0517	169	-	176	0	K.ALTSGQTR.F
1274.6199	1273.6126	1273.7354	-0.1227	165	-	176	1	K.LSLKALTSGQTR.F
1298.6028	1297.5955	1297.6666	-0.0711	106	-	116	0	K.LFDQFLTASTR.E
1299.6059	1298.5987	1298.7081	-0.1095	219	-	230	0	K.EVVTALLESNPK.T
1318.6687	1317.6615	1317.5811	0.0803	13	-	23	0	R.GFNFFVENCNK.D
1400.6846	1399.6774	1399.6442	0.0332	93	-	104	0	K.LTYCAYNNPDVK.K
1690.6642	1689.6569	1689.7627	-0.1059	127	-	140	1	R.RDEVEVNNECQEVK.L
1815.9168	1814.9096	1814.9447	-0.0352	214	-	230	1	K.CDGLKEVVTALLESNPK.T

No match to: 712.2679, 781.4592, 825.1370, 831.4419, 832.3588, 841.0980, 852.4849, 853.5010, 861.0837, 873.4641, 876.3626, 877.0567, 1012.6155, 1157.6270, 1158.6512, 1213.7102, 1224.6023, 1226.5773, 1230.7044, 1235.6798, 1238.8028, 1240.5956, 1242.5818, 1252.6795, 1257.6582, 1257.7440, 1258.5811, 1282.5765, 1283.6177, 1301.6870, 1315.6103, 1334.6603, 1380.7520, 1386.7403, 1402.7407, 1408.7587, 1422.7041, 1424.7200, 1427.7243, 1462.8779, 1467.8310, 1612.8208, 1706.9716, 1723.9839, 2202.1952

47. Q9Y355_HUMAN **Mass:** 7429 **Score:** 49 **Expect:** 37 **Queries matched:** 5

Apolipoprotein A1 (Fragment).- Homo sapiens (Human).

Observed	Mr(expt)	Mr(calc)	Delta	Start		End	Miss	Peptide
781.4592	780.4520	780.4242	0.0278	14	-	20	0	R.AHVDALR.T
831.4419	830.4346	830.4286	0.0060	49	-	55	0	R.LAEYHAK.A
1157.6270	1156.6198	1156.6200	-0.0002	38	-	48	1	R.LEALKENGGAR.L
1301.6870	1300.6797	1300.6411	0.0385	21	-	31	0	R.THLAPYSDELR.Q
1318.6687	1317.6615	1317.6347	0.0268	1	-	11	1	-.LSPLGEEMRDR.A + Oxidation (M)

No match to: 712.2679, 825.1370, 832.3588, 833.3959, 841.0980, 852.4849, 853.5010, 861.0837, 873.4641, 876.3626, 877.0567, 1012.6155, 1158.6512, 1213.7102, 1224.6023, 1226.5773, 1230.7044, 1235.6798, 1238.8028, 1240.5956, 1242.5818, 1252.6795, 1257.6582, 1257.7440, 1258.5811, 1274.6199, 1282.5765, 1283.6177, 1298.6028, 1299.6059, 1315.6103, 1334.6603, 1380.7520, 1386.7403, 1400.6846, 1402.7407, 1408.7587, 1422.7041, 1424.7200, 1427.7243, 1462.8779, 1467.8310, 1612.8208, 1690.6642, 1706.9716, 1723.9839, 1815.9168, 2202.1952

48. C32055 **Mass:** 17279 **Score:** 49 **Expect:** 38 **Queries matched:** 6

nifX protein - Azotobacter vinelandii

Observed	Mr(expt)	Mr(calc)	Delta	Start		End	Miss	Peptide
832.3588	831.3515	831.4021	-0.0506	140	-	146	1	R.TRGPDMR.R
852.4849	851.4776	851.4137	0.0639	22	-	29	0	K.VAFASSDR.E
1213.7102	1212.7029	1212.6900	0.0129	97	-	107	0	R.QLMAIGVQPIK.V + Oxidation (M)
1252.6795	1251.6722	1251.6247	0.0475	41	-	51	0	R.SFAIYGVNPER.S
1274.6199	1273.6126	1273.6051	0.0076	30	-	40	0	R.ELVDQHFGSSR.S
2202.1952	2201.1880	2201.1175	0.0704	2	-	21	1	M.SSPTRQLQVLDSEDDGTLLK.V

No match to: 712.2679, 781.4592, 825.1370, 831.4419, 833.3959, 841.0980, 853.5010, 861.0837, 873.4641, 876.3626, 877.0567, 1012.6155, 1157.6270, 1158.6512, 1224.6023, 1226.5773, 1230.7044, 1235.6798, 1238.8028, 1240.5956, 1242.5818, 1257.6582, 1257.7440, 1258.5811, 1282.5765, 1283.6177, 1298.6028, 1299.6059, 1301.6870, 1315.6103, 1318.6687, 1334.6603, 1380.7520, 1386.7403, 1400.6846, 1402.7407, 1408.7587, 1422.7041, 1424.7200, 1427.7243, 1462.8779, 1467.8310, 1612.8208, 1690.6642, 1706.9716, 1723.9839, 1815.9168

49. Q5ZWH6_LEGPH **Mass:** 79435 **Score:** 49 **Expect:** 38 **Queries matched:** 12

Hypothetical protein.- Legionella pneumophila subsp. pneumophila (strain Philadelphia 1 / ATCC 33152 / DSM 7513).

Observed	Mr(expt)	Mr(calc)	Delta	Start		End	Miss	Peptide
1157.6270	1156.6198	1156.6451	-0.0254	647	-	656	0	K.SLERPEVSLK.Y
1224.6023	1223.5950	1223.7060	-0.1109	333	-	342	1	R.HDILNLMIKK.L
1235.6798	1234.6726	1234.6669	0.0056	322	-	332	1	K.QLALFASDSKR.H
1240.5956	1239.5884	1239.6036	-0.0152	539	-	547	1	K.DNKFWDIFR.N
1252.6795	1251.6722	1251.7121	-0.0399	332	-	341	1	K.RHDILNLMIK.K
1299.6059	1298.5987	1298.6771	-0.0784	72	-	81	1	R.KNLFDFVFNR.T
1386.7403	1385.7330	1385.7164	0.0166	507	-	517	1	K.TINHRSFIENR.K
1400.6846	1399.6774	1399.7248	-0.0474	73	-	83	1	K.NLFDFVFNRTK.T

1408.7587	1407.7515	1407.7245	0.0270	456 -	467	0	R.ELNATTYLINEK.K
1422.7041	1421.6968	1421.7952	-0.0983	353 -	365	1	K.LASVMPYATTKIK.I
1427.7243	1426.7170	1426.7107	0.0063	49 -	59	0	R.FIFTFSLFCFR.A
1815.9168	1814.9096	1814.7693	0.1402	193 -	208	0	K.DENESSSTVGEEYIEK.L

No match to: 712.2679, 781.4592, 825.1370, 831.4419, 832.3588, 833.3959, 841.0980, 852.4849, 853.5010, 861.0837, 873.4641, 876.3626, 877.0567, 1012.6155, 1158.6512, 1213.7102, 1226.5773, 1230.7044, 1238.8028, 1242.5818, 1257.6582, 1257.7440, 1258.5811, 1274.6199, 1282.5765, 1283.6177, 1298.6028, 1301.6870, 1315.6103, 1318.6687, 1334.6603, 1380.7520, 1402.7407, 1424.7200, 1462.8779, 1467.8310, 1612.8208, 1690.6642, 1706.9716, 1723.9839, 2202.1952

50. 1DM9A **Mass:** 11873 **Score:** 49 **Expect:** 40 **Queries matched:** 7

hypothetical 15.5 kd protein in mrca-pcka intergenic region, chain A - bacteria

Observed	Mr(expt)	Mr(calc)	Delta	Start	End	Miss	Peptide
873.4641	872.4568	872.4253	0.0316	32 -	38	0	K.VHYNGQR.S
1158.6512	1157.6440	1157.6345	0.0094	7 -	15	1	R.LDKWLWAAR.F
1240.5956	1239.5884	1239.6553	-0.0669	10 -	18	1	K.WLWAARFYK.T
1257.6582	1256.6509	1256.7452	-0.0943	61 -	71	1	R.TVIVKAITEQR.R
1257.7440	1256.7367	1256.7452	-0.0085	61 -	71	1	R.TVIVKAITEQR.R
1258.5811	1257.5738	1257.6677	-0.0939	55 -	65	1	R.QGNDERTVIVK.A
1400.6846	1399.6774	1399.7320	-0.0546	32 -	43	1	K.VHYNGQRSKPSK.I

No match to: 712.2679, 781.4592, 825.1370, 831.4419, 832.3588, 833.3959, 841.0980, 852.4849, 853.5010, 861.0837, 876.3626, 877.0567, 1012.6155, 1157.6270, 1213.7102, 1224.6023, 1226.5773, 1230.7044, 1235.6798, 1238.8028, 1242.5818, 1252.6795, 1274.6199, 1282.5765, 1283.6177, 1298.6028, 1299.6059, 1301.6870, 1315.6103, 1318.6687, 1334.6603, 1380.7520, 1386.7403, 1402.7407, 1408.7587, 1422.7041, 1424.7200, 1427.7243, 1462.8779, 1467.8310, 1612.8208, 1690.6642, 1706.9716, 1723.9839, 1815.9168, 2202.1952

Search Parameters

Type of search : Peptide Mass Fingerprint
Enzyme : Trypsin
Variable modifications : Carbamidomethyl (C),Oxidation (M)
Mass values : Monoisotopic
Protein Mass : Unrestricted
Peptide Mass Tolerance : ± 100 ppm
Peptide Charge State : 1+
Max Missed Cleavages : 1
Number of queries : 53

Mascot: http://www.matrixscience.com/
