# Supplementary material for: A proteomic strategy to identify novel serum biomarkers for liver cirrhosis and hepatocellular cancer in individuals with fatty liver disease
Source: BMC Cancer. 2009 Aug 5;9:271. doi: 10.1186/1471-2407-9-271 (PMC2729079; doi:10.1186/1471-2407-9-271)
Supplement: Additional File 6 — Spot 3 is Pro- ApoA1. The protein summary report for spot 3, generated using Mascot Peptide Mass Fingerprint search program (Matrix Science Ltd), is compatible with it being Pro- ApoA1. [file 1471-2407-9-271-S6.pdf]

Mascot Search Results

User : Joe Gray
Email : joe.gray@ncl.ac.uk
Search title : DIP_3_0001.dat - SpecView
Database : MSDB 20060831 (3239079 sequences; 1079594700 residues)
Timestamp : 8 Jan 2007 at 11:54:21 GMT
Top Score : 195 for CAA00975, APOA1 PROTEIN (FRAGMENT).- Homo sapiens (Human).

Probability Based Mowse Score

Protein score is -10*Log(P), where P is the probability that the observed match is a random event.
Protein scores greater than 78 are significant (p<0.05).

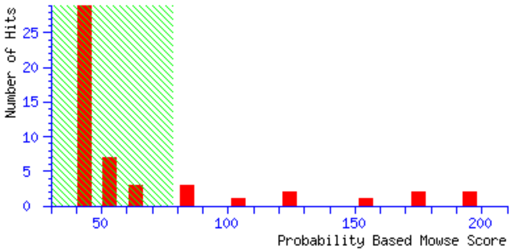

Protein Summary Report

Format As | Protein Summary | Help
Significance threshold p< 0.05
Max. number of hits 50

Re-Search All | Search Unmatched

Index

	Accession	Mass	Score	Description
1.	CAA00975	28061	195	APOA1 PROTEIN (FRAGMENT).- Homo sapiens (Human).
2.	AAA51747	28944	195	HUMAPOAIC NID: - Homo sapiens
3.	LPHUA1	30759	172	apolipoprotein A-I precursor [validated] - human
4.	AAX42892	30872	171	AY890956 NID: - synthetic construct
5.	AAA35545	30745	157	HUMAPOAIP NID: - Homo sapiens
6.	CAA03490	23205	122	SEQUENCE 10 FROM PATENT WO9637608.- unidentified.
7.	1AV1A	23389	122	apolipoprotein a-i lipid-binding domain mutant N-TERMINAL MET, DEL(1-43), chain A - human
8.	Q8HZ97_PANTR	22075	106	Apolipoprotein A-I (Fragment).- Pan troglodytes (Chimpanzee).
9.	CAD61352	18392	88	Sequence 2 from Patent WO02083898 precursor.- Homo sapiens (Human).
10.	A26529	30700	87	apolipoprotein A-I precursor - crab-eating macaque
11.	JS0079	30716	87	apolipoprotein A-I precursor - baboon
12.	Q8HZ95_PONPY	22116	65	Apolipoprotein A-I (Fragment).- Pongo pygmaeus (Orangutan).
13.	Q5ZWV5_LEGPH	35482	65	Putative integrase.- Legionella pneumophila subsp. pneumophila (strain Philadelphia 1 / ATCC 33152 / DSM 7513).
14.	Q390G9_BURS3	26860	61	Short-chain dehydrogenase/reductase SDR (EC 1.1.1.184).- Burkholderia sp. (strain 383) (Burkholderia cepacia (strain ATCC 17760 / NCIB 9086 / R18194)).
15.	Q6BIV2_DEBHA	29600	58	Similar to ca|CA5499|CaRPS4A Candida albicans CaRPS4A ribosomal protein S4.- Debaryomyces hansenii (Yeast) (Torulaspora hansenii).
16.	Q385S6_9TRYP	30688	55	Hypothetical protein.- Trypanosoma brucei.
17.	Q2NC11_9SPHN	26146	54	Transcription terminating nucleic-acid-binding protein.- Erythrobacter litoralis HTCC2594.
18.	Q9AH95_STRPN	24847	52	Wze (Tyrosine-protein kinase Wze) (EC 2.7.1.112).- Streptococcus pneumoniae.
19.	Q5JE97_PYRKO	128609	50	Predicted endonuclease-methyltransferase fusion protein.- Pyrococcus kodakaraensis (Thermococcus kodakaraensis).
20.	Q9DAF2_MOUSE	27632	49	Adult male testis cDNA, RIKEN full-length enriched library, clone:1700011O04 product:SHIPPO 1, full insert sequence (Shippo 1).- Mus musculus (Mouse).
21.	Q1FY16_9GAMM	40803	48	Hypothetical protein.- Psychromonas ingrahamii 37.
22.	Q6BMR2_DEBHA	29600	48	Similar to CA5499|CaRPS4A Candida albicans CaRPS4A ribosomal protein S4.- Debaryomyces hansenii (Yeast) (Torulaspora hansenii).

**23. Q2TBH0_BOVIN**	27616	48	Hypothetical protein.- Bos taurus (Bovine).
**24. Q920N1_MOUSE**	27678	47	Shippo 1.- Mus musculus (Mouse).
**25. E1260070**	5665	46	SEQUENCE 12 FROM PATENT WO9703197.- unidentified.
**26. Q650G1_BACFR**	33704	46	TraP.- Bacteroides fragilis.
**27. Q9AJH0_VIBPR**	12007	46	Hypothetical protein (Fragment).- Vibrio proteolyticus (Aeromonas proteolytica).
**28. Q2H0H7_CHAGB**	24347	46	Hypothetical protein.- Chaetomium globosum CBS 148.51.
**29. Q173X5_AEDAE**	77749	45	Estradiol 17 beta-dehydrogenase.- Aedes aegypti (Yellowfever mosquito).
**30. BAC46077**	33679	45	BA000040 NID: - Bradyrhizobium japonicum USDA 110
**31. Q52SQ8_SIVCZ**	33013	45	Gag protein (Fragment).- Simian immunodeficiency virus (isolate CPZ GAB1) (SIV-cpz) (Chimpanzee immunodeficiency virus).
**32. G75111**	29590	45	hypothetical protein PAB1906 - Pyrococcus abyssi (strain Orsay)
**33. Q1V8I3_VIBAL**	10688	44	Hypothetical protein.- Vibrio alginolyticus 12G01.
**34. Q2AF50_9FIRM**	23685	44	Thymidylate kinase.- Halothermothrix orenii H 168.
**35. Q7NWW0_CHRVO**	25013	44	Probable two-component response regulator, LuxR family.- Chromobacterium violaceum.
**36. Q4X0H5_ASPFU**	153863	44	Dynactin, putative.- Aspergillus fumigatus (Sartorya fumigata).
**37. AAL97589**	22480	44	AE010023 NID: - Streptococcus pyogenes MGAS8232
**38. Q4R1H7_PIG**	47693	44	S-adenosylhomocysteine hydrolase.- Sus scrofa (Pig).
**39. Q5H5A1_XANOR**	18629	44	IS1404 transposase.- Xanthomonas oryzae pv. oryzae.
**40. AAF47775**	144797	44	AE003478 NID: - Drosophila melanogaster
**41. Q3IJD7_PSEHT**	19651	44	Hypothetical protein.- Pseudoalteromonas haloplanktis (strain TAC 125).
**42. Q4LGD1_9BURK**	20049	44	Ribosomal protein L5:Ribosomal protein L5.- Burkholderia cenocepacia HI2424.
**43. Q1INB3_ACIBL**	14018	44	PilT protein-like.- Acidobacteria bacterium (strain Ellin345).
**44. Q2WJV1_CLOBE**	38673	44	Glycosyltransferase.- Clostridium beijerincki NCIMB 8052.
**45. AAM79212**	22460	44	AE014074 NID: - Streptococcus pyogenes MGAS315
**46. B70318**	51423	43	chaperone HslU - Aquifex aeolicus
**47. P79372_PIG**	11271	43	Na+/Ca2+ exchanger isoform NACA3 protein (Fragment).- Sus scrofa (Pig).
**48. E69296**	20123	43	transcription initiation factor IID homolog - Archaeoglobus fulgidus
**49. Q8T530_PLAFA**	14687	43	Erythrocyte membrane protein 1 (Fragment).- Plasmodium falciparum.
**50. Q1JM76_STRPC**	22493	43	GTP-binding protein YihA.- Streptococcus pyogenes serotype M3 (strain MGAS9429).

Results List

1. CAA00975 **Mass:** 28061 **Score:** 195 **Expect:** 1e-13 **Queries matched:** 19

APOA1 PROTEIN (FRAGMENT).- Homo sapiens (Human).

Observed	Mr(expt)	Mr(calc)	Delta	Start	End	Miss	Peptide
1012.6671	1011.6598	1011.5713	0.0886	207	215	0	K.AKPALEDLR.Q
1157.7036	1156.6964	1156.6200	0.0764	178	188	1	R.LEALKENGGAR.L
1226.6034	1225.5961	1225.5364	0.0598	1	10	0	-.DEPPQSPWDR.V
1235.6752	1234.6679	1234.6809	-0.0130	13	23	0	K.DLATVYVDVLK.D
1252.6824	1251.6752	1251.6135	0.0616	97	106	0	K.VQPYLDDFQK.K
1283.6610	1282.6537	1282.5652	0.0885	108	116	0	K.WQEEMELYR.Q
1299.6611	1298.6538	1298.5601	0.0937	108	116	0	K.WQEEMELYR.Q + Oxidation (M)
1301.7377	1300.7304	1300.6411	0.0892	161	171	0	R.THLAPYSDELR.Q
1318.7017	1317.6944	1317.6347	0.0598	141	151	1	K.LSPLGEEMRDR.A + Oxidation (M)
1323.6815	1322.6742	1322.6136	0.0606	78	88	1	K.ETEGLRQEMSK.D + Oxidation (M)
1380.7924	1379.7851	1379.7085	0.0766	97	107	1	K.VQPYLDDFQKK.W
1386.7853	1385.7780	1385.7078	0.0702	227	238	0	K.VSFLSALEEYTK.K
1400.7405	1399.7332	1399.6619	0.0713	28	40	0	R.DYVSQFEGSALGK.Q
1427.7313	1426.7240	1426.6551	0.0690	107	116	1	K.KWQEEMELYR.Q + Oxidation (M)
1467.8666	1466.8594	1466.7841	0.0753	119	131	1	K.VEPLRAELQEGAR.Q
1585.9024	1584.8952	1584.8008	0.0943	161	173	1	R.THLAPYSDELRQR.L
1612.8562	1611.8489	1611.7780	0.0709	46	59	0	K.LLDNWDSVTSTFSK.L
1650.8940	1649.8868	1649.8624	0.0244	13	27	1	K.DLATVYVDVLKDSGR.D
1815.9485	1814.9412	1814.8434	0.0978	24	40	1	K.DSGRDYVSQFEGSALGK.Q

No match to: 700.3201, 713.4158, 731.2732, 781.5109, 831.5215, 832.3782, 832.4806, 833.5146, 839.1638, 845.1403, 845.2394, 855.1207, 861.1503, 877.1304, 892.4161, 893.0865, 1066.1318, 1158.7027, 1213.7329, 1257.6881, 1282.6333, 1284.7370, 1296.7999, 1317.6990, 1334.6923, 1690.7322, 1707.0040, 1723.0042, 1724.0353, 1878.1647, 2109.0717, 2465.4048

2. AAA51747 **Mass:** 28944 **Score:** 195 **Expect:** 1e-13 **Queries matched:** 19

HUMAPOAIC NID: - Homo sapiens

Observed	Mr(expt)	Mr(calc)	Delta	Start	End	Miss	Peptide
1012.6671	1011.6598	1011.5713	0.0886	213	221	0	K.AKPALEDLR.Q
1157.7036	1156.6964	1156.6200	0.0764	184	194	1	R.LEALKENGGAR.L
1235.6752	1234.6679	1234.6809	-0.0130	19	29	0	K.DLATVYVDVLK.D
1252.6824	1251.6752	1251.6135	0.0616	103	112	0	K.VQPYLDDFQK.K
1283.6610	1282.6537	1282.5652	0.0885	114	122	0	K.WQEEMELYR.Q
1299.6611	1298.6538	1298.5601	0.0937	114	122	0	K.WQEEMELYR.Q + Oxidation (M)

1301.7377	1300.7304	1300.6411	0.0892	167 - 177	0	R.THLAPYSDELR.Q
1318.7017	1317.6944	1317.6347	0.0598	147 - 157	1	K.LSPLGEEMRDR.A + Oxidation (M)
1323.6815	1322.6742	1322.6136	0.0606	84 - 94	1	K.ETEGLRQEMSK.D + Oxidation (M)
1380.7924	1379.7851	1379.7085	0.0766	103 - 113	1	K.VQPYLDDFQKK.W
1386.7853	1385.7780	1385.7078	0.0702	233 - 244	0	K.VSFLSALEEYTK.K
1400.7405	1399.7332	1399.6619	0.0713	34 - 46	0	R.DYVSQFEGSALGK.Q
1427.7313	1426.7240	1426.6551	0.0690	113 - 122	1	K.KWQEEMELYR.Q + Oxidation (M)
1467.8666	1466.8594	1466.7841	0.0753	125 - 137	1	K.VEPLRAELQEGAR.Q
1585.9024	1584.8952	1584.8008	0.0943	167 - 179	1	R.THLAPYSDELRQR.L
1612.8562	1611.8489	1611.7780	0.0709	52 - 65	0	K.LLDNWDSVTSTFSK.L
1650.8940	1649.8868	1649.8624	0.0244	19 - 33	1	K.DLATVYVDVLKDSGR.D
1815.9485	1814.9412	1814.8434	0.0978	30 - 46	1	K.DSGRDYVSQFEGSALGK.Q
2109.0717	2108.0644	2107.9613	0.1032	1 - 16	1	-.RHFWQQDEPPQSPWDR.V

No match to: 700.3201, 713.4158, 731.2732, 781.5109, 831.5215, 832.3782, 832.4806, 833.5146, 839.1638, 845.1403, 845.2394, 855.1207, 861.1503, 877.1304, 892.4161, 893.0865, 1066.1318, 1158.7027, 1213.7329, 1226.6034, 1257.6881, 1282.6333, 1284.7370, 1296.7999, 1317.6990, 1334.6923, 1690.7322, 1707.0040, 1723.0042, 1724.0353, 1878.1647, 2465.4048

3. LPHUA1 **Mass:** 30759 **Score:** 172 **Expect:** 2e-11 **Queries matched:** 18

apolipoprotein A-I precursor [validated] - human

Observed	Mr(expt)	Mr(calc)	Delta	Start - End	Miss	Peptide
1012.6671	1011.6598	1011.5713	0.0886	231 - 239	0	K.AKPALEDLR.Q
1157.7036	1156.6964	1156.6200	0.0764	202 - 212	1	R.LEALKENGGAR.L
1235.6752	1234.6679	1234.6809	-0.0130	37 - 47	0	K.DLATVYVDVLK.D
1252.6824	1251.6752	1251.6135	0.0616	121 - 130	0	K.VQPYLDDFQK.K
1283.6610	1282.6537	1282.5652	0.0885	132 - 140	0	K.WQEEMELYR.Q
1299.6611	1298.6538	1298.5601	0.0937	132 - 140	0	K.WQEEMELYR.Q + Oxidation (M)
1301.7377	1300.7304	1300.6411	0.0892	185 - 195	0	R.THLAPYSDELR.Q
1318.7017	1317.6944	1317.6347	0.0598	165 - 175	1	K.LSPLGEEMRDR.A + Oxidation (M)
1323.6815	1322.6742	1322.6136	0.0606	102 - 112	1	K.ETEGLRQEMSK.D + Oxidation (M)
1380.7924	1379.7851	1379.7085	0.0766	121 - 131	1	K.VQPYLDDFQKK.W
1386.7853	1385.7780	1385.7078	0.0702	251 - 262	0	K.VSFLSALEEYTK.K
1400.7405	1399.7332	1399.6619	0.0713	52 - 64	0	R.DYVSQFEGSALGK.Q
1427.7313	1426.7240	1426.6551	0.0690	131 - 140	1	K.KWQEEMELYR.Q + Oxidation (M)
1467.8666	1466.8594	1466.7841	0.0753	143 - 155	1	K.VEPLRAELQEGAR.Q
1585.9024	1584.8952	1584.8008	0.0943	185 - 197	1	R.THLAPYSDELRQR.L
1612.8562	1611.8489	1611.7780	0.0709	70 - 83	0	K.LLDNWDSVTSTFSK.L
1650.8940	1649.8868	1649.8624	0.0244	37 - 51	1	K.DLATVYVDVLKDSGR.D
1815.9485	1814.9412	1814.8434	0.0978	48 - 64	1	K.DSGRDYVSQFEGSALGK.Q

No match to: 700.3201, 713.4158, 731.2732, 781.5109, 831.5215, 832.3782, 832.4806, 833.5146, 839.1638, 845.1403, 845.2394, 855.1207, 861.1503, 877.1304, 892.4161, 893.0865, 1066.1318, 1158.7027, 1213.7329, 1226.6034, 1257.6881, 1282.6333, 1284.7370, 1296.7999, 1317.6990, 1334.6923, 1690.7322, 1707.0040, 1723.0042, 1724.0353, 1878.1647, 2109.0717, 2465.4048

4. AAX42892 **Mass:** 30872 **Score:** 171 **Expect:** 2.6e-11 **Queries matched:** 18

AY890956 NID: - synthetic construct

Observed	Mr(expt)	Mr(calc)	Delta	Start - End	Miss	Peptide
1012.6671	1011.6598	1011.5713	0.0886	231 - 239	0	K.AKPALEDLR.Q
1157.7036	1156.6964	1156.6200	0.0764	202 - 212	1	R.LEALKENGGAR.L
1235.6752	1234.6679	1234.6809	-0.0130	37 - 47	0	K.DLATVYVDVLK.D
1252.6824	1251.6752	1251.6135	0.0616	121 - 130	0	K.VQPYLDDFQK.K
1283.6610	1282.6537	1282.5652	0.0885	132 - 140	0	K.WQEEMELYR.Q
1299.6611	1298.6538	1298.5601	0.0937	132 - 140	0	K.WQEEMELYR.Q + Oxidation (M)
1301.7377	1300.7304	1300.6411	0.0892	185 - 195	0	R.THLAPYSDELR.Q
1318.7017	1317.6944	1317.6347	0.0598	165 - 175	1	K.LSPLGEEMRDR.A + Oxidation (M)
1323.6815	1322.6742	1322.6136	0.0606	102 - 112	1	K.ETEGLRQEMSK.D + Oxidation (M)
1380.7924	1379.7851	1379.7085	0.0766	121 - 131	1	K.VQPYLDDFQKK.W
1386.7853	1385.7780	1385.7078	0.0702	251 - 262	0	K.VSFLSALEEYTK.K
1400.7405	1399.7332	1399.6619	0.0713	52 - 64	0	R.DYVSQFEGSALGK.Q
1427.7313	1426.7240	1426.6551	0.0690	131 - 140	1	K.KWQEEMELYR.Q + Oxidation (M)
1467.8666	1466.8594	1466.7841	0.0753	143 - 155	1	K.VEPLRAELQEGAR.Q
1585.9024	1584.8952	1584.8008	0.0943	185 - 197	1	R.THLAPYSDELRQR.L
1612.8562	1611.8489	1611.7780	0.0709	70 - 83	0	K.LLDNWDSVTSTFSK.L
1650.8940	1649.8868	1649.8624	0.0244	37 - 51	1	K.DLATVYVDVLKDSGR.D
1815.9485	1814.9412	1814.8434	0.0978	48 - 64	1	K.DSGRDYVSQFEGSALGK.Q

No match to: 700.3201, 713.4158, 731.2732, 781.5109, 831.5215, 832.3782, 832.4806, 833.5146, 839.1638, 845.1403, 845.2394, 855.1207, 861.1503, 877.1304, 892.4161, 893.0865, 1066.1318, 1158.7027, 1213.7329, 1226.6034, 1257.6881, 1282.6333, 1284.7370, 1296.7999, 1317.6990, 1334.6923, 1690.7322, 1707.0040, 1723.0042, 1724.0353, 1878.1647, 2109.0717, 2465.4048

5. AAA35545 **Mass:** 30745 **Score:** 157 **Expect:** 6.5e-10 **Queries matched:** 17

HUMAPOAIP NID: - Homo sapiens

Observed	Mr(expt)	Mr(calc)	Delta	Start		End	Miss	Peptide
1012.6671	1011.6598	1011.5713	0.0886	231	-	239	0	K.AKPALEDLR.Q
1157.7036	1156.6964	1156.6200	0.0764	202	-	212	1	R.LEALKENGGAR.L
1235.6752	1234.6679	1234.6809	-0.0130	37	-	47	0	K.DLATVYVDVLK.D
1252.6824	1251.6752	1251.6135	0.0616	121	-	130	0	K.VQPYLDDFQK.K
1283.6610	1282.6537	1282.5652	0.0885	132	-	140	0	K.WQEEMELYR.Q
1299.6611	1298.6538	1298.5601	0.0937	132	-	140	0	K.WQEEMELYR.Q + Oxidation (M)
1301.7377	1300.7304	1300.6411	0.0892	185	-	195	0	R.THLAPYSDELR.Q
1318.7017	1317.6944	1317.6347	0.0598	165	-	175	1	K.LSPLGEEMRDR.A + Oxidation (M)
1323.6815	1322.6742	1322.6136	0.0606	102	-	112	1	K.ETEGLRQEMSK.D + Oxidation (M)
1380.7924	1379.7851	1379.7085	0.0766	121	-	131	1	K.VQPYLDDFQKK.W
1386.7853	1385.7780	1385.7078	0.0702	251	-	262	0	K.VSFLSALEEYTK.K
1400.7405	1399.7332	1399.6619	0.0713	52	-	64	0	R.DYVSQFEGSALGK.Q
1427.7313	1426.7240	1426.6551	0.0690	131	-	140	1	K.KWQEEMELYR.Q + Oxidation (M)
1585.9024	1584.8952	1584.8008	0.0943	185	-	197	1	R.THLAPYSDELRQR.L
1612.8562	1611.8489	1611.7780	0.0709	70	-	83	0	K.LLDNWDSVTSTFSK.L
1650.8940	1649.8868	1649.8624	0.0244	37	-	51	1	K.DLATVYVDVLKDSGR.D
1815.9485	1814.9412	1814.8434	0.0978	48	-	64	1	K.DSGRDYVSQFEGSALGK.Q

No match to: 700.3201, 713.4158, 731.2732, 781.5109, 831.5215, 832.3782, 832.4806, 833.5146, 839.1638, 845.1403, 845.2394, 855.1207, 861.1503, 877.1304, 892.4161, 893.0865, 1066.1318, 1158.7027, 1213.7329, 1226.6034, 1257.6881, 1282.6333, 1284.7370, 1296.7999, 1317.6990, 1334.6923, 1467.8666, 1690.7322, 1707.0040, 1723.0042, 1724.0353, 1878.1647, 2109.0717, 2465.4048

6. CAA03490 **Mass:** 23205 **Score:** **122** **Expect:** 2e-06 **Queries matched:** 14

SEQUENCE 10 FROM PATENT WO9637608.- unidentified.

Observed	Mr(expt)	Mr(calc)	Delta	Start		End	Miss	Peptide
1012.6671	1011.6598	1011.5713	0.0886	164	-	172	0	K.AKPALEDLR.Q
1157.7036	1156.6964	1156.6200	0.0764	135	-	145	1	R.LEALKENGGAR.L
1226.6034	1225.5961	1225.5986	-0.0024	107	-	117	1	R.DCARAHVDALR.T
1252.6824	1251.6752	1251.6135	0.0616	54	-	63	0	K.VQPYLDDFQK.K
1283.6610	1282.6537	1282.6200	0.0337	107	-	117	1	R.DCARAHVDALR.T + Carbamidomethyl (C)
1299.6611	1298.6538	1298.5601	0.0937	65	-	73	0	K.WQEEMELYR.Q + Oxidation (M)
1301.7377	1300.7304	1300.6411	0.0892	118	-	128	0	R.THLAPYSDELR.Q
1323.6815	1322.6742	1322.6136	0.0606	35	-	45	1	K.ETEGLRQEMSK.D + Oxidation (M)
1380.7924	1379.7851	1379.7085	0.0766	54	-	64	1	K.VQPYLDDFQKK.W
1386.7853	1385.7780	1385.7078	0.0702	184	-	195	0	K.VSFLSALEEYTK.K
1427.7313	1426.7240	1426.6551	0.0690	64	-	73	1	K.KWQEEMELYR.Q + Oxidation (M)
1467.8666	1466.8594	1466.7841	0.0753	76	-	88	1	K.VEPLRAELQEGAR.Q
1585.9024	1584.8952	1584.8008	0.0943	118	-	130	1	R.THLAPYSDELRQR.L
1612.8562	1611.8489	1611.7780	0.0709	3	-	16	0	K.LLDNWDSVTSTFSK.L

No match to: 700.3201, 713.4158, 731.2732, 781.5109, 831.5215, 832.3782, 832.4806, 833.5146, 839.1638, 845.1403, 845.2394, 855.1207, 861.1503, 877.1304, 892.4161, 893.0865, 1066.1318, 1158.7027, 1213.7329, 1235.6752, 1257.6881, 1282.6333, 1284.7370, 1296.7999, 1317.6990, 1318.7017, 1334.6923, 1400.7405, 1650.8940, 1690.7322, 1707.0040, 1723.0042, 1724.0353, 1815.9485, 1878.1647, 2109.0717, 2465.4048

7. 1AV1A **Mass:** 23389 **Score:** **122** **Expect:** 2e-06 **Queries matched:** 14

apolipoprotein a-i lipid-binding domain mutant N-TERMINAL MET, DEL(1-43), chain A - human

Observed	Mr(expt)	Mr(calc)	Delta	Start		End	Miss	Peptide
1012.6671	1011.6598	1011.5713	0.0886	165	-	173	0	K.AKPALEDLR.Q
1157.7036	1156.6964	1156.6200	0.0764	136	-	146	1	R.LEALKENGGAR.L
1252.6824	1251.6752	1251.6135	0.0616	55	-	64	0	K.VQPYLDDFQK.K
1283.6610	1282.6537	1282.5652	0.0885	66	-	74	0	K.WQEEMELYR.Q
1299.6611	1298.6538	1298.5601	0.0937	66	-	74	0	K.WQEEMELYR.Q + Oxidation (M)
1301.7377	1300.7304	1300.6411	0.0892	119	-	129	0	R.THLAPYSDELR.Q
1318.7017	1317.6944	1317.6347	0.0598	99	-	109	1	K.LSPLGEEMRDR.A + Oxidation (M)
1323.6815	1322.6742	1322.6136	0.0606	36	-	46	1	K.ETEGLRQEMSK.D + Oxidation (M)
1380.7924	1379.7851	1379.7085	0.0766	55	-	65	1	K.VQPYLDDFQKK.W
1386.7853	1385.7780	1385.7078	0.0702	185	-	196	0	K.VSFLSALEEYTK.K
1427.7313	1426.7240	1426.6551	0.0690	65	-	74	1	K.KWQEEMELYR.Q + Oxidation (M)
1467.8666	1466.8594	1466.7841	0.0753	77	-	89	1	K.VEPLRAELQEGAR.Q
1585.9024	1584.8952	1584.8008	0.0943	119	-	131	1	R.THLAPYSDELRQR.L
1612.8562	1611.8489	1611.7780	0.0709	4	-	17	0	K.LLDNWDSVTSTFSK.L

No match to: 700.3201, 713.4158, 731.2732, 781.5109, 831.5215, 832.3782, 832.4806, 833.5146, 839.1638, 845.1403, 845.2394, 855.1207, 861.1503, 877.1304, 892.4161, 893.0865, 1066.1318, 1158.7027, 1213.7329, 1226.6034, 1235.6752, 1257.6881, 1282.6333, 1284.7370, 1296.7999, 1317.6990, 1334.6923, 1400.7405, 1650.8940, 1690.7322, 1707.0040, 1723.0042, 1724.0353, 1815.9485, 1878.1647, 2109.0717, 2465.4048

8. Q8HZ97_PANTR **Mass:** 22075 **Score:** **106** **Expect:** 8.1e-05 **Queries matched:** 12

Apolipoprotein A-I (Fragment).- Pan troglodytes (Chimpanzee).

Observed	Mr(expt)	Mr(calc)	Delta	Start		End	Miss	Peptide
1157.7036	1156.6964	1156.6200	0.0764	165	-	175	1	R.LEALKENGGAR.L

Observed	Mr(expt)	Mr(calc)	Delta	Start	End	Miss	Peptide
1252.6824	1251.6752	1251.6135	0.0616	84	93	0	K.VQPYLDDFQK.K
1283.6610	1282.6537	1282.5652	0.0885	95	103	0	K.WQEEMELYR.Q
1299.6611	1298.6538	1298.5601	0.0937	95	103	0	K.WQEEMELYR.Q + Oxidation (M)
1301.7377	1300.7304	1300.6411	0.0892	148	158	0	R.THLAPYSDELR.Q
1323.6815	1322.6742	1322.6136	0.0606	65	75	1	K.ETEGLRQEMSK.D + Oxidation (M)
1380.7924	1379.7851	1379.7085	0.0766	84	94	1	K.VQPYLDDFQKK.W
1400.7405	1399.7332	1399.6619	0.0713	15	27	0	R.DYVSQFEGSALGK.Q
1427.7313	1426.7240	1426.6551	0.0690	94	103	1	K.KWQEEMELYR.Q + Oxidation (M)
1585.9024	1584.8952	1584.8008	0.0943	148	160	1	R.THLAPYSDELRQR.L
1612.8562	1611.8489	1611.7780	0.0709	33	46	0	K.LLDNWDSVTSTFSK.L
1815.9485	1814.9412	1814.8434	0.0978	11	27	1	K.DSGRDYVSQFEGSALGK.Q

No match to: 700.3201, 713.4158, 731.2732, 781.5109, 831.5215, 832.3782, 832.4806, 833.5146, 839.1638, 845.1403, 845.2394, 855.1207, 861.1503, 877.1304, 892.4161, 893.0865, 1012.6671, 1066.1318, 1158.7027, 1213.7329, 1226.6034, 1235.6752, 1257.6881, 1282.6333, 1284.7370, 1296.7999, 1317.6990, 1318.7017, 1334.6923, 1386.7853, 1467.8666, 1650.8940, 1690.7322, 1707.0040, 1723.0042, 1724.0353, 1878.1647, 2109.0717, 2465.4048

9. CAD61352 **Mass:** 18392 **Score:** **88** **Expect:** 0.0047 **Queries matched:** 10

Sequence 2 from Patent WO02083898 precursor.- Homo sapiens (Human).

Observed	Mr(expt)	Mr(calc)	Delta	Start	End	Miss	Peptide
1235.6752	1234.6679	1234.6809	-0.0130	37	47	0	K.DLATVYVDVLK.D
1252.6824	1251.6752	1251.6135	0.0616	121	130	0	K.VQPYLDDFQK.K
1283.6610	1282.6537	1282.5652	0.0885	132	140	0	K.WQEEMELYR.Q
1299.6611	1298.6538	1298.5601	0.0937	132	140	0	K.WQEEMELYR.Q + Oxidation (M)
1323.6815	1322.6742	1322.6136	0.0606	102	112	1	K.ETEGLRQEMSK.D + Oxidation (M)
1380.7924	1379.7851	1379.7085	0.0766	121	131	1	K.VQPYLDDFQKK.W
1400.7405	1399.7332	1399.6619	0.0713	52	64	0	R.DYVSQFEGSALGK.Q
1427.7313	1426.7240	1426.6551	0.0690	131	140	1	K.KWQEEMELYR.Q + Oxidation (M)
1650.8940	1649.8868	1649.8624	0.0244	37	51	1	K.DLATVYVDVLKDSGR.D
1815.9485	1814.9412	1814.8434	0.0978	48	64	1	K.DSGRDYVSQFEGSALGK.Q

No match to: 700.3201, 713.4158, 731.2732, 781.5109, 831.5215, 832.3782, 832.4806, 833.5146, 839.1638, 845.1403, 845.2394, 855.1207, 861.1503, 877.1304, 892.4161, 893.0865, 1012.6671, 1066.1318, 1157.7036, 1158.7027, 1213.7329, 1226.6034, 1257.6881, 1282.6333, 1284.7370, 1296.7999, 1301.7377, 1317.6990, 1318.7017, 1334.6923, 1386.7853, 1467.8666, 1585.9024, 1612.8562, 1690.7322, 1707.0040, 1723.0042, 1724.0353, 1878.1647, 2109.0717, 2465.4048

10. A26529 **Mass:** 30700 **Score:** **87** **Expect:** 0.0062 **Queries matched:** 12

apolipoprotein A-I precursor - crab-eating macaque

Observed	Mr(expt)	Mr(calc)	Delta	Start	End	Miss	Peptide
1012.6671	1011.6598	1011.5713	0.0886	231	239	0	K.AKPALEDLR.Q
1157.7036	1156.6964	1156.6200	0.0764	202	212	1	R.LEALKENGGAR.L
1252.6824	1251.6752	1251.6135	0.0616	121	130	0	K.VQPYLDDFQK.K
1283.6610	1282.6537	1282.5652	0.0885	132	140	0	K.WQEEMELYR.Q
1299.6611	1298.6538	1298.5601	0.0937	132	140	0	K.WQEEMELYR.Q + Oxidation (M)
1301.7377	1300.7304	1300.6411	0.0892	185	195	0	R.THLAPYSDELR.Q
1323.6815	1322.6742	1322.6136	0.0606	102	112	1	K.ETEGLRQEMSK.D + Oxidation (M)
1380.7924	1379.7851	1379.7085	0.0766	121	131	1	K.VQPYLDDFQKK.W
1386.7853	1385.7780	1385.7078	0.0702	251	262	0	K.VSFLSALEEYTK.K
1400.7405	1399.7332	1399.6619	0.0713	52	64	0	K.DYVSQFEGSALGK.Q
1427.7313	1426.7240	1426.6551	0.0690	131	140	1	K.KWQEEMELYR.Q + Oxidation (M)
1585.9024	1584.8952	1584.8008	0.0943	185	197	1	R.THLAPYSDELRQR.L

No match to: 700.3201, 713.4158, 731.2732, 781.5109, 831.5215, 832.3782, 832.4806, 833.5146, 839.1638, 845.1403, 845.2394, 855.1207, 861.1503, 877.1304, 892.4161, 893.0865, 1066.1318, 1158.7027, 1213.7329, 1226.6034, 1235.6752, 1257.6881, 1282.6333, 1284.7370, 1296.7999, 1317.6990, 1318.7017, 1334.6923, 1467.8666, 1612.8562, 1650.8940, 1690.7322, 1707.0040, 1723.0042, 1724.0353, 1815.9485, 1878.1647, 2109.0717, 2465.4048

11. JS0079 **Mass:** 30716 **Score:** **87** **Expect:** 0.0062 **Queries matched:** 12

apolipoprotein A-I precursor - baboon

Observed	Mr(expt)	Mr(calc)	Delta	Start	End	Miss	Peptide
1012.6671	1011.6598	1011.5713	0.0886	231	239	0	K.AKPALEDLR.Q
1157.7036	1156.6964	1156.6200	0.0764	202	212	1	R.LEALKENGGAR.L
1252.6824	1251.6752	1251.6135	0.0616	121	130	0	K.VQPYLDDFQK.K
1283.6610	1282.6537	1282.5652	0.0885	132	140	0	K.WQEEMELYR.Q
1299.6611	1298.6538	1298.5601	0.0937	132	140	0	K.WQEEMELYR.Q + Oxidation (M)
1301.7377	1300.7304	1300.6411	0.0892	185	195	0	R.THLAPYSDELR.Q
1323.6815	1322.6742	1322.6136	0.0606	102	112	1	K.ETEGLRQEMSK.D + Oxidation (M)
1380.7924	1379.7851	1379.7085	0.0766	121	131	1	K.VQPYLDDFQKK.W
1386.7853	1385.7780	1385.7078	0.0702	251	262	0	K.VSFLSALEEYTK.K
1400.7405	1399.7332	1399.6619	0.0713	52	64	0	K.DYVSQFEGSALGK.Q
1427.7313	1426.7240	1426.6551	0.0690	131	140	1	K.KWQEEMELYR.Q + Oxidation (M)
1585.9024	1584.8952	1584.8008	0.0943	185	197	1	R.THLAPYSDELRQR.L

No match to: 700.3201, 713.4158, 731.2732, 781.5109, 831.5215, 832.3782, 832.4806, 833.5146, 839.1638, 845.1403, 845.2394, 855.1207, 861.1503, 877.1304, 892.4161, 893.0865, 1066.1318, 1158.7027, 1213.7329, 1226.6034, 1235.6752, 1257.6881, 1282.6333, 1284.7370, 1296.7999, 1317.6990, 1318.7017, 1334.6923, 1467.8666, 1612.8562, 1650.8940, 1690.7322, 1707.0040, 1723.0042, 1724.0353, 1815.9485, 1878.1647, 2109.0717, 2465.4048

12. Q8HZ95_PONPY **Mass:** 22116 **Score:** 65 **Expect:** 0.98 **Queries matched:** 9

Apolipoprotein A-I (Fragment).- Pongo pygmaeus (Orangutan).

Observed	Mr(expt)	Mr(calc)	Delta	Start	End	Miss	Peptide
1157.7036	1156.6964	1156.6200	0.0764	165 -	175	1	R.LEALKENGGAR.L
1252.6824	1251.6752	1251.6135	0.0616	84 -	93	0	K.VQPYLDDFQK.K
1283.6610	1282.6537	1282.5652	0.0885	95 -	103	0	K.WQEEMELYR.Q
1299.6611	1298.6538	1298.5601	0.0937	95 -	103	0	K.WQEEMELYR.Q + Oxidation (M)
1323.6815	1322.6742	1322.6136	0.0606	65 -	75	1	K.ETEGLRQEMSK.D + Oxidation (M)
1380.7924	1379.7851	1379.7085	0.0766	84 -	94	1	K.VQPYLDDFQKK.W
1400.7405	1399.7332	1399.6619	0.0713	15 -	27	0	R.DYVSQFEGSALGK.Q
1427.7313	1426.7240	1426.6551	0.0690	94 -	103	1	K.KWQEEMELYR.Q + Oxidation (M)
1815.9485	1814.9412	1814.8434	0.0978	11 -	27	1	K.DSGRDYVSQFEGSALGK.Q

No match to: 700.3201, 713.4158, 731.2732, 781.5109, 831.5215, 832.3782, 832.4806, 833.5146, 839.1638, 845.1403, 845.2394, 855.1207, 861.1503, 877.1304, 892.4161, 893.0865, 1012.6671, 1066.1318, 1158.7027, 1213.7329, 1226.6034, 1235.6752, 1257.6881, 1282.6333, 1284.7370, 1296.7999, 1301.7377, 1317.6990, 1318.7017, 1334.6923, 1386.7853, 1467.8666, 1585.9024, 1612.8562, 1650.8940, 1690.7322, 1707.0040, 1723.0042, 1724.0353, 1878.1647, 2109.0717, 2465.4048

13. Q5ZWV5_LEGPH **Mass:** 35482 **Score:** 65 **Expect:** 1 **Queries matched:** 11

Putative integrase.- Legionella pneumophila subsp. pneumophila (strain Philadelphia 1 / ATCC 33152 / DSM 7513).

Observed	Mr(expt)	Mr(calc)	Delta	Start	End	Miss	Peptide
1158.7027	1157.6954	1157.5797	0.1157	231 -	240	1	K.MGLSKCHGLR.H + Carbamidomethyl (C)
1226.6034	1225.5961	1225.6415	-0.0453	288 -	298	0	R.EIISQSLGHSR.L
1252.6824	1251.6752	1251.6724	0.0028	160 -	170	0	K.IVLSHAWQGNK.L
1282.6333	1281.6260	1281.6023	0.0237	16 -	26	0	K.NAQYSINECIK.K
1283.6610	1282.6537	1282.6193	0.0343	248 -	257	1	R.YHEITKSYDK.T
1296.7999	1295.7926	1295.7812	0.0114	95 -	106	0	K.VASVLNKPELVK.E
1299.6611	1298.6538	1298.6904	-0.0365	2 -	13	1	M.ILYGGISMSKSK.L + Oxidation (M)
1323.6815	1322.6742	1322.7135	-0.0393	67 -	76	0	K.HIYILVDHWK.A
1467.8666	1466.8594	1466.7187	0.1406	16 -	27	1	K.NAQYSINECIKK.I + Carbamidomethyl (C)
1585.9024	1584.8952	1584.7970	0.0982	49 -	61	0	K.DLHELGYMITHIK.G + Oxidation (M)
2109.0717	2108.0644	2108.1452	-0.0807	49 -	66	1	K.DLHELGYMITHIKGLKPK.H + Oxidation (M)

No match to: 700.3201, 713.4158, 731.2732, 781.5109, 831.5215, 832.3782, 832.4806, 833.5146, 839.1638, 845.1403, 845.2394, 855.1207, 861.1503, 877.1304, 892.4161, 893.0865, 1012.6671, 1066.1318, 1157.7036, 1213.7329, 1235.6752, 1257.6881, 1284.7370, 1301.7377, 1317.6990, 1318.7017, 1334.6923, 1380.7924, 1386.7853, 1400.7405, 1427.7313, 1612.8562, 1650.8940, 1690.7322, 1707.0040, 1723.0042, 1724.0353, 1815.9485, 1878.1647, 2465.4048

14. Q390G9_BURS3 **Mass:** 26860 **Score:** 61 **Expect:** 2.7 **Queries matched:** 8

Short-chain dehydrogenase/reductase SDR (EC 1.1.1.184).- Burkholderia sp. (strain 383) (Burkholderia cepacia (strain ATCC 17760 / NCIB 9086 / R18194)).

Observed	Mr(expt)	Mr(calc)	Delta	Start	End	Miss	Peptide
833.5146	832.5074	832.4476	0.0597	165 -	172	0	K.AAVVNMTK.A
1226.6034	1225.5961	1225.6012	-0.0051	1 -	11	0	-.MATNLFDLTGK.I + Oxidation (M)
1235.6752	1234.6679	1234.5692	0.0987	122 -	132	0	R.GYFFMSVEAGK.L
1318.7017	1317.6944	1317.6863	0.0081	173 -	184	1	K.AFAKECGPLGIR.V + Carbamidomethyl (C)
1400.7405	1399.7332	1399.7095	0.0237	71 -	82	0	R.LDDIAATFEHIR.G
1585.9024	1584.8952	1584.8260	0.0692	71 -	84	1	R.LDDIAATFEHIRGK.H
1724.0353	1723.0280	1722.9338	0.0942	157 -	172	1	R.QGIYSITKAAVVNMTK.A
2465.4048	2464.3975	2464.2281	0.1694	61 -	82	1	R.AEALACHVGRLDDIAATFEHIR.G + Carbamidomethyl (C)

No match to: 700.3201, 713.4158, 731.2732, 781.5109, 831.5215, 832.3782, 832.4806, 839.1638, 845.1403, 845.2394, 855.1207, 861.1503, 877.1304, 892.4161, 893.0865, 1012.6671, 1066.1318, 1157.7036, 1158.7027, 1213.7329, 1252.6824, 1257.6881, 1282.6333, 1283.6610, 1284.7370, 1296.7999, 1299.6611, 1301.7377, 1317.6990, 1323.6815, 1334.6923, 1380.7924, 1386.7853, 1427.7313, 1467.8666, 1612.8562, 1650.8940, 1690.7322, 1707.0040, 1723.0042, 1815.9485, 1878.1647, 2109.0717

15. Q6BIV2_DEBHA **Mass:** 29600 **Score:** 58 **Expect:** 5.4 **Queries matched:** 9

Similar to ca|CA5499|CaRPS4A Candida albicans CaRPS4A ribosomal protein S4.- Debaryomyces hansenii (Yeast) (Torulaspora hansenii).

Observed	Mr(expt)	Mr(calc)	Delta	Start	End	Miss	Peptide
781.5109	780.5036	780.4606	0.0430	192 -	198	0	R.VGVITHR.E
832.4806	831.4734	831.4636	0.0097	180 -	187	0	R.LVMVTGGR.N
833.5146	832.5074	832.4290	0.0784	247 -	253	0	K.LSISEER.D
1213.7329	1212.7256	1212.6251	0.1005	135 -	145	0	R.GIPYVVTHDGR.T
1257.6881	1256.6809	1256.6037	0.0772	169 -	179	0	K.VTDFISFDTGR.L
1284.7370	1283.7297	1283.6332	0.0965	12 -	22	0	R.LAAPSHWMLDK.L + Oxidation (M)
1317.6990	1316.6917	1316.6935	-0.0018	156 -	168	1	R.ANDSVKVDLATGK.V
1323.6815	1322.6742	1322.7081	-0.0339	114 -	125	1	R.ITAEEASYKLAK.V
1467.8666	1466.8594	1466.7915	0.0679	63 -	75	1	K.AILMQEHVKVDGK.V

No match to: 700.3201, 713.4158, 731.2732, 831.5215, 832.3782, 839.1638, 845.1403, 845.2394, 855.1207, 861.1503, 877.1304, 892.4161, 893.0865, 1012.6671, 1066.1318, 1157.7036, 1158.7027, 1226.6034, 1235.6752, 1252.6824, 1282.6333, 1283.6610, 1296.7999, 1299.6611, 1301.7377, 1318.7017, 1334.6923, 1380.7924, 1386.7853, 1400.7405, 1427.7313, 1585.9024, 1612.8562, 1650.8940, 1690.7322, 1707.0040, 1723.0042, 1724.0353, 1815.9485, 1878.1647, 2109.0717, 2465.4048

16. Q385S6_9TRYP **Mass:** 30688 **Score:** 55 **Expect:** 11 **Queries matched:** 9

Hypothetical protein.- Trypanosoma brucei.

Observed	Mr(expt)	Mr(calc)	Delta	Start	End	Miss	Peptide
700.3201	699.3128	699.3816	-0.0688	101 -	106	0	K.FHGALR.T
713.4158	712.4085	712.4231	-0.0146	222 -	228	0	R.ASLPAVR.N
892.4161	891.4088	891.3821	0.0267	143 -	150	0	R.EESDDVAK.K
1235.6752	1234.6679	1234.6094	0.0585	126 -	135	1	K.DFVHRYAEAK.M
1257.6881	1256.6809	1256.6037	0.0772	152 -	162	0	K.VATEAYTEAFR.K
1299.6611	1298.6538	1298.6077	0.0461	212 -	221	0	K.YVTCPNQVYR.A + Carbamidomethyl (C)
1301.7377	1300.7304	1300.6987	0.0317	19 -	30	0	R.TDLLDTVAQLGR.H
1427.7313	1426.7240	1426.7027	0.0214	211 -	221	1	R.KYVTCPNQVYR.A + Carbamidomethyl (C)
2109.0717	2108.0644	2108.0208	0.0436	39 -	58	0	K.AVTTADAPLPSCTFNTTVSR.S + Carbamidomethyl (C)

No match to: 731.2732, 781.5109, 831.5215, 832.3782, 832.4806, 833.5146, 839.1638, 845.1403, 845.2394, 855.1207, 861.1503, 877.1304, 893.0865, 1012.6671, 1066.1318, 1157.7036, 1158.7027, 1213.7329, 1226.6034, 1252.6824, 1282.6333, 1283.6610, 1284.7370, 1296.7999, 1317.6990, 1318.7017, 1323.6815, 1334.6923, 1380.7924, 1386.7853, 1400.7405, 1467.8666, 1585.9024, 1612.8562, 1650.8940, 1690.7322, 1707.0040, 1723.0042, 1724.0353, 1815.9485, 1878.1647, 2465.4048

17. Q2NC11_9SPHN **Mass:** 26146 **Score:** 54 **Expect:** 12 **Queries matched:** 9

Transcription terminating nucleic-acid-binding protein.- Erythrobacter litoralis HTCC2594.

Observed	Mr(expt)	Mr(calc)	Delta	Start	End	Miss	Peptide
713.4158	712.4085	712.3504	0.0581	3 -	8	0	R.TPPNER.V
831.5215	830.5142	830.4610	0.0532	236 -	243	1	K.DSLAAAKR.H
832.3782	831.3709	831.4450	-0.0741	212 -	218	0	R.VEQTLSR.L
832.4806	831.4734	831.4450	0.0283	212 -	218	0	R.VEQTLSR.L
833.5146	832.5074	832.4919	0.0155	87 -	94	1	R.GALARAFK.G
1226.6034	1225.5961	1225.6679	-0.0718	61 -	72	1	R.APGRGAWIGVSR.A
1235.6752	1234.6679	1234.6153	0.0526	9 -	20	1	R.VSSDISDGTKAR.T
1650.8940	1649.8868	1649.8988	-0.0120	45 -	60	0	R.LAISPDGDVLPDVLAR.A
1724.0353	1723.0280	1722.9529	0.0751	212 -	226	1	R.VEQTLSRLLHFAGPR.E

No match to: 700.3201, 731.2732, 781.5109, 839.1638, 845.1403, 845.2394, 855.1207, 861.1503, 877.1304, 892.4161, 893.0865, 1012.6671, 1066.1318, 1157.7036, 1158.7027, 1213.7329, 1252.6824, 1257.6881, 1282.6333, 1283.6610, 1284.7370, 1296.7999, 1299.6611, 1301.7377, 1317.6990, 1318.7017, 1323.6815, 1334.6923, 1380.7924, 1386.7853, 1400.7405, 1427.7313, 1467.8666, 1585.9024, 1612.8562, 1690.7322, 1707.0040, 1723.0042, 1815.9485, 1878.1647, 2109.0717, 2465.4048

18. Q9AH95_STRPN **Mass:** 24847 **Score:** 52 **Expect:** 20 **Queries matched:** 7

Wze (Tyrosine-protein kinase Wze) (EC 2.7.1.112).- Streptococcus pneumoniae.

Observed	Mr(expt)	Mr(calc)	Delta	Start	End	Miss	Peptide
1158.7027	1157.6954	1157.6478	0.0476	1 -	10	1	-.MPTLEIAQKK.L
1252.6824	1251.6752	1251.5917	0.0834	134 -	143	0	K.NFNDMIETLR.K
1299.6611	1298.6538	1298.5567	0.0971	215 -	226	0	K.YGAYGSYGNYGK.K
1380.7924	1379.7851	1379.6867	0.0984	134 -	144	1	K.NFNDMIETLRK.Y
1427.7313	1426.7240	1426.6516	0.0724	215 -	227	1	K.YGAYGSYGNYGKK.-
1467.8666	1466.8594	1466.7518	0.1076	50 -	62	0	K.TTTSINIAWSFAR.A
1690.7322	1689.7249	1689.8719	-0.1470	169 -	184	1	K.CDASILITATGEVNKR.D

No match to: 700.3201, 713.4158, 731.2732, 781.5109, 831.5215, 832.3782, 832.4806, 833.5146, 839.1638, 845.1403, 845.2394, 855.1207, 861.1503, 877.1304, 892.4161, 893.0865, 1012.6671, 1066.1318, 1157.7036, 1213.7329, 1226.6034, 1235.6752, 1257.6881, 1282.6333, 1283.6610, 1284.7370, 1296.7999, 1301.7377, 1317.6990, 1318.7017, 1323.6815, 1334.6923, 1386.7853, 1400.7405, 1585.9024, 1612.8562, 1650.8940, 1707.0040, 1723.0042, 1724.0353, 1815.9485, 1878.1647, 2109.0717, 2465.4048

19. Q5JE97_PYRKO **Mass:** 128609 **Score:** 50 **Expect:** 32 **Queries matched:** 13

Predicted endonuclease-methyltransferase fusion protein.- Pyrococcus kodakaraensis (Thermococcus kodakaraensis).

Observed	Mr(expt)	Mr(calc)	Delta	Start	End	Miss	Peptide
1158.7027	1157.6954	1157.6669	0.0285	349 -	357	1	K.RLYQNLVPR.E
1226.6034	1225.5961	1225.5437	0.0524	194 -	202	0	R.TEMLFEDWR.R
1235.6752	1234.6679	1234.6532	0.0147	529 -	538	0	K.GQFIVYLHMK.L
1257.6881	1256.6809	1256.6005	0.0803	632 -	641	1	K.RDMSMLFVAR.T + 2 Oxidation (M)
1317.6990	1316.6917	1316.6347	0.0571	1076 -	1086	0	R.ELEEELAEVEK.K
1380.7924	1379.7851	1379.7521	0.0330	28 -	39	1	R.HNVSKVLDEIAR.E
1400.7405	1399.7332	1399.7935	-0.0603	350 -	360	1	R.LYQNLVPREIR.H
1585.9024	1584.8952	1584.9239	-0.0287	833 -	846	1	K.VVETDLIFPVLRGK.N
1612.8562	1611.8489	1611.9130	-0.0641	153 -	166	1	R.GLTRKPLDAEMLLR.D
1650.8940	1649.8868	1649.8446	0.0421	510 -	523	1	K.LMDKVFQAIEDGLR.R + Oxidation (M)
1724.0353	1723.0280	1722.8940	0.1340	85 -	99	0	K.QAFNSALDQVINYIK.S
2109.0717	2108.0644	2108.1041	-0.0397	544 -	561	1	R.LSDDTIDAISVLYEKLWK.L
2465.4048	2464.3975	2464.3060	0.0914	463 -	485	1	R.VDDITIPVYLADSITVSTKTSAR.G

No match to: 700.3201, 713.4158, 731.2732, 781.5109, 831.5215, 832.3782, 832.4806, 833.5146, 839.1638, 845.1403, 845.2394, 855.1207, 861.1503, 877.1304, 892.4161, 893.0865, 1012.6671, 1066.1318, 1157.7036, 1213.7329, 1252.6824, 1282.6333, 1283.6610, 1284.7370, 1296.7999, 1299.6611, 1301.7377, 1318.7017, 1323.6815, 1334.6923, 1386.7853, 1427.7313, 1467.8666, 1690.7322, 1707.0040, 1723.0042, 1815.9485, 1878.1647

20. Q9DAF2_MOUSE **Mass:** 27632 **Score:** 49 **Expect:** 45 **Queries matched:** 9

Adult male testis cDNA, RIKEN full-length enriched library, clone:1700011O04 product:SHIPPO 1, full insert sequence (Shippo 1).- Mus musculus (Mouse).

Observed	Mr(expt)	Mr(calc)	Delta	Start		End	Miss	Peptide
832.3782	831.3709	831.4239	-0.0529	179	-	186	0	K.TPGPAAYR.Q
832.4806	831.4734	831.4239	0.0495	179	-	186	0	K.TPGPAAYR.Q
1235.6752	1234.6679	1234.6961	-0.0282	30	-	40	0	K.YLIPPTTGFVK.H
1283.6610	1282.6537	1282.6492	0.0045	195	-	205	1	K.FKAPQYTMAAR.V
1299.6611	1298.6538	1298.6441	0.0097	195	-	205	1	K.FKAPQYTMAAR.V + Oxidation (M)
1317.6990	1316.6917	1316.6798	0.0119	17	-	29	0	R.GPIMALYSSPGPK.Y
1318.7017	1317.6944	1317.7041	-0.0096	213	-	225	0	K.TLKPGPGAHSPEK.V
1585.9024	1584.8952	1584.7388	0.1564	55	-	69	0	R.GAPMLLAENCSPGPR.Y + Carbamidomethyl (C); Oxidation (M)
1690.7322	1689.7249	1689.8838	-0.1589	82	-	96	1	K.DLGPAYSILGRYHTK.T

No match to: 700.3201, 713.4158, 731.2732, 781.5109, 831.5215, 833.5146, 839.1638, 845.1403, 845.2394, 855.1207, 861.1503, 877.1304, 892.4161, 893.0865, 1012.6671, 1066.1318, 1157.7036, 1158.7027, 1213.7329, 1226.6034, 1252.6824, 1257.6881, 1282.6333, 1284.7370, 1296.7999, 1301.7377, 1323.6815, 1334.6923, 1380.7924, 1386.7853, 1400.7405, 1427.7313, 1467.8666, 1612.8562, 1650.8940, 1707.0040, 1723.0042, 1724.0353, 1815.9485, 1878.1647, 2109.0717, 2465.4048

21. Q1FY16_9GAMM **Mass:** 40803 **Score:** 48 **Expect:** 49 **Queries matched:** 8

Hypothetical protein.- Psychromonas ingrahamii 37.

Observed	Mr(expt)	Mr(calc)	Delta	Start		End	Miss	Peptide
832.4806	831.4734	831.4966	-0.0233	234	-	241	0	K.VFAAVLGR.V
1235.6752	1234.6679	1234.6207	0.0472	92	-	100	1	K.LRYHFTQDR.K
1252.6824	1251.6752	1251.6346	0.0405	305	-	316	0	K.GIYVVIDSGDSK.S
1283.6610	1282.6537	1282.6227	0.0310	292	-	301	0	K.QLQQYLMSEK.I + Oxidation (M)
1284.7370	1283.7297	1283.6357	0.0940	280	-	291	1	R.SNSSKALEGYTK.Q
1299.6611	1298.6538	1298.6910	-0.0372	80	-	90	0	K.DIPSFYVFLAK.K
1427.7313	1426.7240	1426.7860	-0.0619	80	-	91	1	K.DIPSFYVFLAKK.L
1612.8562	1611.8489	1611.8178	0.0311	15	-	27	1	K.LKMYIESSIIDER.N + Oxidation (M)

No match to: 700.3201, 713.4158, 731.2732, 781.5109, 831.5215, 832.3782, 833.5146, 839.1638, 845.1403, 845.2394, 855.1207, 861.1503, 877.1304, 892.4161, 893.0865, 1012.6671, 1066.1318, 1157.7036, 1158.7027, 1213.7329, 1226.6034, 1257.6881, 1282.6333, 1296.7999, 1301.7377, 1317.6990, 1318.7017, 1323.6815, 1334.6923, 1380.7924, 1386.7853, 1400.7405, 1467.8666, 1585.9024, 1650.8940, 1690.7322, 1707.0040, 1723.0042, 1724.0353, 1815.9485, 1878.1647, 2109.0717, 2465.4048

22. Q6BMR2_DEBHA **Mass:** 29600 **Score:** 48 **Expect:** 53 **Queries matched:** 8

Similar to CA5499|CaRPS4A Candida albicans CaRPS4A ribosomal protein S4.- Debaryomyces hansenii (Yeast) (Torulaspora hansenii).

Observed	Mr(expt)	Mr(calc)	Delta	Start		End	Miss	Peptide
781.5109	780.5036	780.4606	0.0430	192	-	198	0	R.VGVITHR.E
832.4806	831.4734	831.4636	0.0097	180	-	187	0	R.LVMVTGGR.N
833.5146	832.5074	832.4290	0.0784	247	-	253	0	K.LSISEER.D
1213.7329	1212.7256	1212.6251	0.1005	135	-	145	0	R.GIPYVVTHDGR.T
1284.7370	1283.7297	1283.6332	0.0965	12	-	22	0	R.LAAPSHWMLDK.L + Oxidation (M)
1317.6990	1316.6917	1316.6935	-0.0018	156	-	168	1	R.ANDSVKVDLATGK.I
1323.6815	1322.6742	1322.7081	-0.0339	114	-	125	1	R.ITAEEASYKLAK.V
1467.8666	1466.8594	1466.7915	0.0679	63	-	75	1	K.AILMQEHVKVDGK.V

No match to: 700.3201, 713.4158, 731.2732, 831.5215, 832.3782, 839.1638, 845.1403, 845.2394, 855.1207, 861.1503, 877.1304, 892.4161, 893.0865, 1012.6671, 1066.1318, 1157.7036, 1158.7027, 1226.6034, 1235.6752, 1252.6824, 1257.6881, 1282.6333, 1283.6610, 1296.7999, 1299.6611, 1301.7377, 1318.7017, 1334.6923, 1380.7924, 1386.7853, 1400.7405, 1427.7313, 1585.9024, 1612.8562, 1650.8940, 1690.7322, 1707.0040, 1723.0042, 1724.0353, 1815.9485, 1878.1647, 2109.0717, 2465.4048

23. Q2TBH0_BOVIN **Mass:** 27616 **Score:** 48 **Expect:** 56 **Queries matched:** 9

Hypothetical protein.- Bos taurus (Bovine).

Observed	Mr(expt)	Mr(calc)	Delta	Start		End	Miss	Peptide
832.3782	831.3709	831.4239	-0.0529	179	-	186	0	K.TPGPAAYR.Q
832.4806	831.4734	831.4239	0.0495	179	-	186	0	K.TPGPAAYR.Q
1235.6752	1234.6679	1234.6961	-0.0282	30	-	40	0	K.YLIPPTTGFVK.Q
1283.6610	1282.6537	1282.6492	0.0045	195	-	205	1	K.FKAPQYTMAAR.V
1299.6611	1298.6538	1298.6441	0.0097	195	-	205	1	K.FKAPQYTMAAR.V + Oxidation (M)
1317.6990	1316.6917	1316.6798	0.0119	17	-	29	0	R.GPIMALYSSPGPK.Y
1318.7017	1317.6944	1317.7041	-0.0096	213	-	225	0	K.TLKPGPGAHSPEK.V
1585.9024	1584.8952	1584.7388	0.1564	55	-	69	0	R.GAPMLLAENCSPGPR.Y + Carbamidomethyl (C); Oxidation (M)
1690.7322	1689.7249	1689.8838	-0.1589	82	-	96	1	K.DLGPAYSILGRYHTK.T

No match to: 700.3201, 713.4158, 731.2732, 781.5109, 831.5215, 833.5146, 839.1638, 845.1403, 845.2394, 855.1207, 861.1503, 877.1304, 892.4161, 893.0865, 1012.6671, 1066.1318, 1157.7036, 1158.7027, 1213.7329, 1226.6034, 1252.6824, 1257.6881, 1282.6333, 1284.7370, 1296.7999, 1301.7377, 1323.6815, 1334.6923, 1380.7924, 1386.7853, 1400.7405, 1427.7313, 1467.8666, 1612.8562, 1650.8940, 1707.0040, 1723.0042, 1724.0353, 1815.9485, 1878.1647, 2109.0717, 2465.4048

24. Q920N1_MOUSE **Mass:** 27678 **Score:** 47 **Expect:** 59 **Queries matched:** 9

Shippo 1.- Mus musculus (Mouse).

Observed	Mr(expt)	Mr(calc)	Delta	Start		End	Miss	Peptide
832.3782	831.3709	831.4239	-0.0529	179	-	186	0	K.TPGPAAYR.Q
832.4806	831.4734	831.4239	0.0495	179	-	186	0	K.TPGPAAYR.Q
1235.6752	1234.6679	1234.6961	-0.0282	30	-	40	0	K.YLIPPTTGFVK.H
1283.6610	1282.6537	1282.6492	0.0045	195	-	205	1	K.FKAPQYTMAAR.V

1299.6611	1298.6538	1298.6441	0.0097	195 - 205	1	K.FKAPQYTMAAR.V + Oxidation (M)
1317.6990	1316.6917	1316.6798	0.0119	17 - 29	0	R.GPIMALYSSPGPK.Y
1318.7017	1317.6944	1317.7041	-0.0096	213 - 225	0	K.TLKPGPGAHSPEK.V
1585.9024	1584.8952	1584.7388	0.1564	55 - 69	0	R.GAPMLLAENCSPGPR.Y + Carbamidomethyl (C); Oxidation (M)
1690.7322	1689.7249	1689.8838	-0.1589	82 - 96	1	K.DLGPAYSILGRYHTK.T

No match to: 700.3201, 713.4158, 731.2732, 781.5109, 831.5215, 833.5146, 839.1638, 845.1403, 845.2394, 855.1207, 861.1503, 877.1304, 892.4161, 893.0865, 1012.6671, 1066.1318, 1157.7036, 1158.7027, 1213.7329, 1226.6034, 1252.6824, 1257.6881, 1282.6333, 1284.7370, 1296.7999, 1301.7377, 1323.6815, 1334.6923, 1380.7924, 1386.7853, 1400.7405, 1427.7313, 1467.8666, 1612.8562, 1650.8940, 1707.0040, 1723.0042, 1724.0353, 1815.9485, 1878.1647, 2109.0717, 2465.4048

25. E1260070 **Mass:** 5665 **Score:** 46 **Expect:** 73 **Queries matched:** 5

SEQUENCE 12 FROM PATENT WO9703197.- unidentified.

Observed	Mr(expt)	Mr(calc)	Delta	Start End	Miss	Peptide
831.5215	830.5142	830.5047	0.0094	21 - 27	1	K.VVNMLKK.N
1318.7017	1317.6944	1317.7690	-0.0746	15 - 26	1	K.TTTIAKVVNMLK.K
1334.6923	1333.6850	1333.7639	-0.0789	15 - 26	1	K.TTTIAKVVNMLK.K + Oxidation (M)
1650.8940	1649.8868	1649.8736	0.0131	27 - 41	1	K.KNNLSTIIAASDTFR.A
2109.0717	2108.0644	2108.2033	-0.1389	1 - 20	1	-.PFVIIFFGVNGVGKTTTIAK.V

No match to: 700.3201, 713.4158, 731.2732, 781.5109, 832.3782, 832.4806, 833.5146, 839.1638, 845.1403, 845.2394, 855.1207, 861.1503, 877.1304, 892.4161, 893.0865, 1012.6671, 1066.1318, 1157.7036, 1158.7027, 1213.7329, 1226.6034, 1235.6752, 1252.6824, 1257.6881, 1282.6333, 1283.6610, 1284.7370, 1296.7999, 1299.6611, 1301.7377, 1317.6990, 1323.6815, 1380.7924, 1386.7853, 1400.7405, 1427.7313, 1467.8666, 1585.9024, 1612.8562, 1690.7322, 1707.0040, 1723.0042, 1724.0353, 1815.9485, 1878.1647, 2465.4048

26. Q650G1_BACFR **Mass:** 33704 **Score:** 46 **Expect:** 81 **Queries matched:** 8

TraP.- Bacteroides fragilis.

Observed	Mr(expt)	Mr(calc)	Delta	Start End	Miss	Peptide
700.3201	699.3128	699.3704	-0.0576	171 - 175	1	R.SRYFK.G
892.4161	891.4088	891.4848	-0.0760	254 - 261	0	R.TLAALTMR.Y + Oxidation (M)
1226.6034	1225.5961	1225.6131	-0.0170	54 - 63	0	K.QLWYDFGLGK.G
1235.6752	1234.6679	1234.5975	0.0703	1 - 10	1	-.MERTEIDAVR.R + Oxidation (M)
1252.6824	1251.6752	1251.6346	0.0405	123 - 132	0	R.SLLTEYLEER.G
1284.7370	1283.7297	1283.7271	0.0026	176 - 187	1	K.GCIPPKSVSLVK.A + Carbamidomethyl (C)
1334.6923	1333.6850	1333.6132	0.0719	142 - 151	1	R.HCCRLNYGVR.G + 2 Carbamidomethyl (C)
1400.7405	1399.7332	1399.6402	0.0931	266 - 277	1	R.VTDRSSLYDGCK.D + Carbamidomethyl (C)

No match to: 713.4158, 731.2732, 781.5109, 831.5215, 832.3782, 832.4806, 833.5146, 839.1638, 845.1403, 845.2394, 855.1207, 861.1503, 877.1304, 893.0865, 1012.6671, 1066.1318, 1157.7036, 1158.7027, 1213.7329, 1257.6881, 1282.6333, 1283.6610, 1296.7999, 1299.6611, 1301.7377, 1317.6990, 1318.7017, 1323.6815, 1380.7924, 1386.7853, 1427.7313, 1467.8666, 1585.9024, 1612.8562, 1650.8940, 1690.7322, 1707.0040, 1723.0042, 1724.0353, 1815.9485, 1878.1647, 2109.0717, 2465.4048

27. Q9AJH0_VIBPR **Mass:** 12007 **Score:** 46 **Expect:** 85 **Queries matched:** 5

Hypothetical protein (Fragment).- Vibrio proteolyticus (Aeromonas proteolytica).

Observed	Mr(expt)	Mr(calc)	Delta	Start End	Miss	Peptide
1157.7036	1156.6964	1156.6815	0.0148	13 - 22	1	R.EVVLARELTK.T
1257.6881	1256.6809	1256.6699	0.0110	48 - 58	0	R.GEMVLLVHGFR.A
1334.6923	1333.6850	1333.7605	-0.0755	85 - 97	1	K.AAALAAEIYSVKK.N
1400.7405	1399.7332	1399.7248	0.0084	98 - 109	1	K.NALYKWGLEHLG.-
1467.8666	1466.8594	1466.9072	-0.0478	72 - 84	1	R.TLAILTQELPLKK.A

No match to: 700.3201, 713.4158, 731.2732, 781.5109, 831.5215, 832.3782, 832.4806, 833.5146, 839.1638, 845.1403, 845.2394, 855.1207, 861.1503, 877.1304, 892.4161, 893.0865, 1012.6671, 1066.1318, 1158.7027, 1213.7329, 1226.6034, 1235.6752, 1252.6824, 1282.6333, 1283.6610, 1284.7370, 1296.7999, 1299.6611, 1301.7377, 1317.6990, 1318.7017, 1323.6815, 1380.7924, 1386.7853, 1427.7313, 1585.9024, 1612.8562, 1650.8940, 1690.7322, 1707.0040, 1723.0042, 1724.0353, 1815.9485, 1878.1647, 2109.0717, 2465.4048

28. Q2H0H7_CHAGB **Mass:** 24347 **Score:** 46 **Expect:** 89 **Queries matched:** 7

Hypothetical protein.- Chaetomium globosum CBS 148.51.

Observed	Mr(expt)	Mr(calc)	Delta	Start End	Miss	Peptide
831.5215	830.5142	830.4497	0.0645	16 - 23	1	R.AEADGLKK.H
892.4161	891.4088	891.4484	-0.0396	175 - 183	0	R.TGAAMNALK.S + Oxidation (M)
1282.6333	1281.6260	1281.7332	-0.1072	106 - 116	1	R.TLDLSKLFAFK.K
1283.6610	1282.6537	1282.6339	0.0198	68 - 79	1	R.SLAEKAYATCAR.A
1323.6815	1322.6742	1322.6466	0.0276	162 - 174	0	K.LGEAAGSSLEYAR.T
1386.7853	1385.7780	1385.7813	-0.0033	117 - 128	1	K.KPTRELSLLCAR.E
1815.9485	1814.9412	1814.9162	0.0250	129 - 144	1	R.EDFEPPVARLLSETGR.L

No match to: 700.3201, 713.4158, 731.2732, 781.5109, 832.3782, 832.4806, 833.5146, 839.1638, 845.1403, 845.2394, 855.1207, 861.1503, 877.1304, 893.0865, 1012.6671, 1066.1318, 1157.7036, 1158.7027, 1213.7329, 1226.6034, 1235.6752, 1252.6824, 1257.6881, 1284.7370, 1296.7999, 1299.6611, 1301.7377, 1317.6990, 1318.7017, 1334.6923, 1380.7924, 1400.7405, 1427.7313, 1467.8666, 1585.9024, 1612.8562, 1650.8940, 1690.7322, 1707.0040, 1723.0042, 1724.0353, 1878.1647, 2109.0717, 2465.4048

29. Q173X5_AEDAE **Mass:** 77749 **Score:** 45 **Expect:** 1e+02 **Queries matched:** 11

Estradiol 17 beta-dehydrogenase.- Aedes aegypti (Yellowfever mosquito).

Observed	Mr(expt)	Mr(calc)	Delta	Start End	Miss	Peptide
832.3782	831.3709	831.4313	-0.0603	136 - 142	0	R.AAWPIMK.K + Oxidation (M)
832.4806	831.4734	831.4313	0.0421	136 - 142	0	R.AAWPIMK.K + Oxidation (M)
1235.6752	1234.6679	1234.6557	0.0122	607 - 618	0	K.SLQSDAVFAGIK.D

```
1252.6824  1251.6752  1251.6281   0.0471    85 -  95   0  K.IIQTAMEAFGR.V + Oxidation (M)
1296.7999  1295.7926  1295.7561   0.0365    96 - 107   0  R.VDVLINNAGILR.D
1299.6611  1298.6538  1298.7744  -0.1205   595 - 606   1  K.KIVLKPNMTAGK.S
1301.7377  1300.7304  1300.6047   0.1256   534 - 544   1  K.RYGNDDSSLFK.A
1585.9024  1584.8952  1584.8367   0.0584   515 - 529   0  K.TPILHGLCTMGISVK.A + Oxidation (M)
1612.8562  1611.8489  1611.7901   0.0588   129 - 142   1  K.GSFMTTRAAWPIMK.K + Oxidation (M)
1724.0353  1723.0280  1723.0429  -0.0149   699 - 714   1  K.ITGNIMLAQKLAPLLK.T
2465.4048  2464.3975  2464.2080   0.1895    61 -  84   1  K.VVEEIRSAGGTAVADYNSVVDGEK.I
```

No match to: 700.3201, 713.4158, 731.2732, 781.5109, 831.5215, 833.5146, 839.1638, 845.1403, 845.2394, 855.1207, 861.1503, 877.1304, 892.4161, 893.0865, 1012.6671, 1066.1318, 1157.7036, 1158.7027, 1213.7329, 1226.6034, 1257.6881, 1282.6333, 1283.6610, 1284.7370, 1317.6990, 1318.7017, 1323.6815, 1334.6923, 1380.7924, 1386.7853, 1400.7405, 1427.7313, 1467.8666, 1650.8940, 1690.7322, 1707.0040, 1723.0042, 1815.9485, 1878.1647, 2109.0717

30. BAC46077 **Mass:** 33679 **Score:** 45 **Expect:** 1e+02 **Queries matched:** 8

BA000040 NID: - Bradyrhizobium japonicum USDA 110

Observed	Mr(expt)	Mr(calc)	Delta	Start	End	Miss	Peptide
832.3782	831.3709	831.4337	-0.0628	141	148	0	K.AGLVESEK.L
832.4806	831.4734	831.4337	0.0396	141	148	0	K.AGLVESEK.L
1226.6034	1225.5961	1225.5826	0.0135	127	138	0	K.EYATSGTLAGEK.L
1235.6752	1234.6679	1234.6557	0.0122	191	201	0	R.AIYTLGEELAR.E
1318.7017	1317.6944	1317.6850	0.0094	2	13	0	M.TTMLSSDLPLPK.I + Oxidation (M)
1334.6923	1333.6850	1333.7064	-0.0214	107	117	0	R.TTVFPIECVIR.G + Carbamidomethyl (C)
1467.8666	1466.8594	1466.7616	0.0977	127	140	1	K.EYATSGTLAGEKLK.A
1724.0353	1723.0280	1722.8940	0.1340	202	216	1	R.EQGIIIADTKFEFGR.D

No match to: 700.3201, 713.4158, 731.2732, 781.5109, 831.5215, 833.5146, 839.1638, 845.1403, 845.2394, 855.1207, 861.1503, 877.1304, 892.4161, 893.0865, 1012.6671, 1066.1318, 1157.7036, 1158.7027, 1213.7329, 1252.6824, 1257.6881, 1282.6333, 1283.6610, 1284.7370, 1296.7999, 1299.6611, 1301.7377, 1317.6990, 1323.6815, 1380.7924, 1386.7853, 1400.7405, 1427.7313, 1585.9024, 1612.8562, 1650.8940, 1690.7322, 1707.0040, 1723.0042, 1815.9485, 1878.1647, 2109.0717, 2465.4048

31. Q52SQ8_SIVCZ **Mass:** 33013 **Score:** 45 **Expect:** 1e+02 **Queries matched:** 7

Gag protein (Fragment).- Simian immunodeficiency virus (isolate CPZ GAB1) (SIV-cpz) (Chimpanzee immunodeficiency virus).

Observed	Mr(expt)	Mr(calc)	Delta	Start	End	Miss	Peptide
831.5215	830.5142	830.4650	0.0492	153	159	0	R.TLNAWVK.L
832.3782	831.3709	831.4338	-0.0628	12	18	1	K.KADELEK.I
832.4806	831.4734	831.4814	-0.0080	5	12	1	R.NSVLSGKK.A
1323.6815	1322.6742	1322.6540	0.0202	278	288	0	R.MYNPTNILDVK.Q + Oxidation (M)
1400.7405	1399.7332	1399.7758	-0.0426	267	277	1	R.WIQLGLQKCVR.M + Carbamidomethyl (C)
1467.8666	1466.8594	1466.7664	0.0930	118	131	0	K.MPITSRPTAPPSGR.G
1723.0042	1721.9970	1721.8593	0.1377	275	288	1	K.CVRMYNPTNILDVK.Q + Carbamidomethyl (C)

No match to: 700.3201, 713.4158, 731.2732, 781.5109, 833.5146, 839.1638, 845.1403, 845.2394, 855.1207, 861.1503, 877.1304, 892.4161, 893.0865, 1012.6671, 1066.1318, 1157.7036, 1158.7027, 1213.7329, 1226.6034, 1235.6752, 1252.6824, 1257.6881, 1282.6333, 1283.6610, 1284.7370, 1296.7999, 1299.6611, 1301.7377, 1317.6990, 1318.7017, 1334.6923, 1380.7924, 1386.7853, 1427.7313, 1585.9024, 1612.8562, 1650.8940, 1690.7322, 1707.0040, 1724.0353, 1815.9485, 1878.1647, 2109.0717, 2465.4048

32. G75111 **Mass:** 29590 **Score:** 45 **Expect:** 1.1e+02 **Queries matched:** 7

hypothetical protein PAB1906 - Pyrococcus abyssi (strain Orsay)

Observed	Mr(expt)	Mr(calc)	Delta	Start	End	Miss	Peptide
1157.7036	1156.6964	1156.6815	0.0148	87	96	1	R.ETVELAKVLR.A
1252.6824	1251.6752	1251.6975	-0.0223	97	106	1	R.AKLEVNLFYR.T
1257.6881	1256.6809	1256.6553	0.0255	11	19	1	R.YWSLLYREK.I
1317.6990	1316.6917	1316.7122	-0.0204	20	31	1	K.IIEGMERGITAK.A
1427.7313	1426.7240	1426.7303	-0.0063	120	133	0	R.EAGAEEVLGINPTK.R
1585.9024	1584.8952	1584.8657	0.0295	51	65	1	K.TIKPAEEAMRAAVAK.L
2109.0717	2108.0644	2108.1476	-0.0832	114	133	1	K.IAEVLREAGAEEVLGINPTK.R

No match to: 700.3201, 713.4158, 731.2732, 781.5109, 831.5215, 832.3782, 832.4806, 833.5146, 839.1638, 845.1403, 845.2394, 855.1207, 861.1503, 877.1304, 892.4161, 893.0865, 1012.6671, 1066.1318, 1158.7027, 1213.7329, 1226.6034, 1235.6752, 1282.6333, 1283.6610, 1284.7370, 1296.7999, 1299.6611, 1301.7377, 1318.7017, 1323.6815, 1334.6923, 1380.7924, 1386.7853, 1400.7405, 1467.8666, 1612.8562, 1650.8940, 1690.7322, 1707.0040, 1723.0042, 1724.0353, 1815.9485, 1878.1647, 2465.4048

33. Q1V8I3_VIBAL **Mass:** 10688 **Score:** 44 **Expect:** 1.2e+02 **Queries matched:** 5

Hypothetical protein.- Vibrio alginolyticus 12G01.

Observed	Mr(expt)	Mr(calc)	Delta	Start	End	Miss	Peptide
1226.6034	1225.5961	1225.5938	0.0023	47	56	1	R.IEKHDNDEVK.T
1299.6611	1298.6538	1298.5635	0.0903	80	90	0	K.SQMFIDQMGDK.I
1427.7313	1426.7240	1426.6584	0.0656	79	90	1	K.KSQMFIDQMGDK.I
1690.7322	1689.7249	1689.7378	-0.0129	80	93	1	K.SQMFIDQMGDKIFD.- + Oxidation (M)
1815.9485	1814.9412	1814.8838	0.0574	27	42	0	K.GFLIYPESLSEGGQYR.I

No match to: 700.3201, 713.4158, 731.2732, 781.5109, 831.5215, 832.3782, 832.4806, 833.5146, 839.1638, 845.1403, 845.2394, 855.1207, 861.1503, 877.1304, 892.4161, 893.0865, 1012.6671, 1066.1318, 1157.7036, 1158.7027, 1213.7329, 1235.6752, 1252.6824, 1257.6881, 1282.6333, 1283.6610, 1284.7370, 1296.7999, 1301.7377, 1317.6990, 1318.7017, 1323.6815, 1334.6923, 1380.7924, 1386.7853, 1400.7405, 1467.8666, 1585.9024, 1612.8562, 1650.8940, 1707.0040, 1723.0042, 1724.0353, 1878.1647, 2109.0717, 2465.4048

34. Q2AF50_9FIRM **Mass:** 23685 **Score:** 44 **Expect:** 1.2e+02 **Queries matched:** 6

Thymidylate kinase.- Halothermothrix orenii H 168.

Observed	Mr(expt)	Mr(calc)	Delta	Start	End	Miss	Peptide
1284.7370	1283.7297	1283.6543	0.0754	17 -	26	1	K.STQIEMLYKR.L + Oxidation (M)
1299.6611	1298.6538	1298.6904	-0.0366	52 -	63	0	K.LLLDPDNIMGAK.A
1386.7853	1385.7780	1385.6800	0.0980	170 -	180	1	R.QAYHQLAEDRR.F
1427.7313	1426.7240	1426.7853	-0.0613	51 -	63	1	R.KLLLDPDNIMGAK.A
1724.0353	1723.0280	1722.8974	0.1306	1 -	16	1	-.MRGLFITLEGIEGSGK.S + Oxidation (M)
2465.4048	2464.3975	2464.2671	0.1304	52 -	73	1	K.LLLDPDNIMGAKAEFLLYAADR.A + Oxidation (M)

No match to: 700.3201, 713.4158, 731.2732, 781.5109, 831.5215, 832.3782, 832.4806, 833.5146, 839.1638, 845.1403, 845.2394, 855.1207, 861.1503, 877.1304, 892.4161, 893.0865, 1012.6671, 1066.1318, 1157.7036, 1158.7027, 1213.7329, 1226.6034, 1235.6752, 1252.6824, 1257.6881, 1282.6333, 1283.6610, 1296.7999, 1301.7377, 1317.6990, 1318.7017, 1323.6815, 1334.6923, 1380.7924, 1400.7405, 1467.8666, 1585.9024, 1612.8562, 1650.8940, 1690.7322, 1707.0040, 1723.0042, 1815.9485, 1878.1647, 2109.0717

35. Q7NWW0_CHRVO **Mass:** 25013 **Score:** 44 **Expect:** 1.2e+02 **Queries matched:** 8

Probable two-component response regulator, LuxR family.- Chromobacterium violaceum.

Observed	Mr(expt)	Mr(calc)	Delta	Start	End	Miss	Peptide
831.5215	830.5142	830.4610	0.0532	151 -	157	1	K.EANRLTK.K
832.3782	831.3709	831.4086	-0.0377	133 -	140	0	R.TAADIEGR.V
832.4806	831.4734	831.4086	0.0647	133 -	140	0	R.TAADIEGR.V
833.5146	832.5074	832.4767	0.0307	185 -	191	1	K.TISTQKR.S
1301.7377	1300.7304	1300.6986	0.0317	121 -	132	0	K.GDSQLALLESIR.T
1724.0353	1723.0280	1722.8796	0.1484	106 -	120	1	K.ICQGKNVGVCEIIYK.G + Carbamidomethyl (C)
1815.9485	1814.9412	1814.8760	0.0652	201 -	216	0	K.NDFGLMSYLELEGIAK.I + Oxidation (M)
2109.0717	2108.0644	2107.9442	0.1203	58 -	76	0	K.DSAPCDVLVIDFSMPDEAK.D + Carbamidomethyl (C)

No match to: 700.3201, 713.4158, 731.2732, 781.5109, 839.1638, 845.1403, 845.2394, 855.1207, 861.1503, 877.1304, 892.4161, 893.0865, 1012.6671, 1066.1318, 1157.7036, 1158.7027, 1213.7329, 1226.6034, 1235.6752, 1252.6824, 1257.6881, 1282.6333, 1283.6610, 1284.7370, 1296.7999, 1299.6611, 1317.6990, 1318.7017, 1323.6815, 1334.6923, 1380.7924, 1386.7853, 1400.7405, 1427.7313, 1467.8666, 1585.9024, 1612.8562, 1650.8940, 1690.7322, 1707.0040, 1723.0042, 1878.1647, 2465.4048

36. Q4X0H5_ASPFU **Mass:** 153863 **Score:** 44 **Expect:** 1.2e+02 **Queries matched:** 15

Dynactin, putative.- Aspergillus fumigatus (Sartorya fumigata).

Observed	Mr(expt)	Mr(calc)	Delta	Start	End	Miss	Peptide
831.5215	830.5142	830.4497	0.0645	1086 -	1092	0	R.VELVESR.M
832.3782	831.3709	831.4239	-0.0529	946 -	952	0	K.ELSAWAR.Q
832.4806	831.4734	831.4239	0.0495	946 -	952	0	K.ELSAWAR.Q
833.5146	832.5074	832.4290	0.0784	222 -	230	0	R.SSISGPASK.A
892.4161	891.4088	891.4522	-0.0434	136 -	143	1	R.SASQRTSR.V
1235.6752	1234.6679	1234.5751	0.0928	968 -	978	0	R.TEPLTMEEASK.T
1252.6824	1251.6752	1251.6432	0.0320	83 -	95	1	K.QPARGGASAPANR.G
1257.6881	1256.6809	1256.6361	0.0448	171 -	183	0	K.SPSTPAVPASSTR.S
1283.6610	1282.6537	1282.6266	0.0271	123 -	135	0	R.HSVSAASPSPTSR.S
1284.7370	1283.7297	1283.7601	-0.0304	1245 -	1255	1	R.DVFAHLLKLTK.E
1317.6990	1316.6917	1316.6513	0.0404	1297 -	1306	1	R.WAEWKDDIVR.H
1318.7017	1317.6944	1317.6412	0.0532	393 -	403	1	K.DTEAKLEEVER.L
1380.7924	1379.7851	1379.6867	0.0984	205 -	218	0	K.TSMGPPPPPSVAAR.Q + Oxidation (M)
1612.8562	1611.8489	1611.8290	0.0199	692 -	705	0	R.MNAEEALQHLSILK.L + Oxidation (M)
1815.9485	1814.9412	1814.9560	-0.0148	150 -	166	0	R.ANVLVTANMSQVQSPLK.S + Oxidation (M)

No match to: 700.3201, 713.4158, 731.2732, 781.5109, 839.1638, 845.1403, 845.2394, 855.1207, 861.1503, 877.1304, 893.0865, 1012.6671, 1066.1318, 1157.7036, 1158.7027, 1213.7329, 1226.6034, 1282.6333, 1296.7999, 1299.6611, 1301.7377, 1323.6815, 1334.6923, 1386.7853, 1400.7405, 1427.7313, 1467.8666, 1585.9024, 1650.8940, 1690.7322, 1707.0040, 1723.0042, 1724.0353, 1878.1647, 2109.0717, 2465.4048

37. AAL97589 **Mass:** 22480 **Score:** 44 **Expect:** 1.2e+02 **Queries matched:** 7

AE010023 NID: - Streptococcus pyogenes MGAS8232

Observed	Mr(expt)	Mr(calc)	Delta	Start	End	Miss	Peptide
1158.7027	1157.6954	1157.6516	0.0438	54 -	64	1	K.NLARTSSKPGK.T
1235.6752	1234.6679	1234.7033	-0.0354	43 -	53	1	K.SSFINTILGRK.N
1301.7377	1300.7304	1300.6009	0.1295	128 -	137	0	K.EDIQMYDFLK.Y
1317.6990	1316.6917	1316.5958	0.0959	128 -	137	0	K.EDIQMYDFLK.Y + Oxidation (M)
1380.7924	1379.7851	1379.7700	0.0151	138 -	149	0	K.YYDIPVIVVATK.A
1386.7853	1385.7780	1385.6826	0.0954	174 -	185	0	K.SDTFIVFSSVER.I
1467.8666	1466.8594	1466.7405	0.1188	65 -	76	0	K.TQLLNFFNIDDK.L

No match to: 700.3201, 713.4158, 731.2732, 781.5109, 831.5215, 832.3782, 832.4806, 833.5146, 839.1638, 845.1403, 845.2394, 855.1207, 861.1503, 877.1304, 892.4161, 893.0865, 1012.6671, 1066.1318, 1157.7036, 1213.7329, 1226.6034, 1252.6824, 1257.6881, 1282.6333, 1283.6610, 1284.7370, 1296.7999, 1299.6611, 1318.7017, 1323.6815, 1334.6923, 1400.7405, 1427.7313, 1585.9024, 1612.8562, 1650.8940, 1690.7322, 1707.0040, 1723.0042, 1724.0353, 1815.9485, 1878.1647, 2109.0717, 2465.4048

38. Q4R1H7_PIG **Mass:** 47693 **Score:** 44 **Expect:** 1.2e+02 **Queries matched:** 8

S-adenosylhomocysteine hydrolase.- Sus scrofa (Pig).

Observed	Mr(expt)	Mr(calc)	Delta	Start	End	Miss	Peptide
832.4806	831.4734	831.5065	-0.0332	406 -	412	1	K.LTKLTEK.Q
1158.7027	1157.6954	1157.6193	0.0761	9 -	19	0	K.VADISLAAWGR.K

1252.6824	1251.6752	1251.6459	0.0293	390	- 401	0	K.LDEAVAEAHLGK.L
1284.7370	1283.7297	1283.7390	-0.0092	379	- 389	1	K.YPVGVHFLPKK.L
1380.7924	1379.7851	1379.7408	0.0443	389	- 401	1	K.KLDEAVAEAHLGK.L
1467.8666	1466.8594	1466.7551	0.1043	37	- 49	1	R.EMYSTSKPLKGAR.I
1585.9024	1584.8952	1584.9351	-0.0399	319	- 331	1	K.VNIKPQVDRYLLK.N
1707.0040	1705.9967	1705.9362	0.0605	390	- 405	1	K.LDEAVAEAHLGKLNVK.L

No match to: 700.3201, 713.4158, 731.2732, 781.5109, 831.5215, 832.3782, 833.5146, 839.1638, 845.1403, 845.2394, 855.1207, 861.1503, 877.1304, 892.4161, 893.0865, 1012.6671, 1066.1318, 1157.7036, 1213.7329, 1226.6034, 1235.6752, 1257.6881, 1282.6333, 1283.6610, 1296.7999, 1299.6611, 1301.7377, 1317.6990, 1318.7017, 1323.6815, 1334.6923, 1386.7853, 1400.7405, 1427.7313, 1612.8562, 1650.8940, 1690.7322, 1723.0042, 1724.0353, 1815.9485, 1878.1647, 2109.0717, 2465.4048

39. Q5H5A1_XANOR **Mass:** 18629 **Score:** 44 **Expect:** 1.2e+02 **Queries matched:** 7

IS1404 transposase.- Xanthomonas oryzae pv. oryzae.

Observed	Mr(expt)	Mr(calc)	Delta	Start	End	Miss	Peptide
832.3782	831.3709	831.4272	-0.0563	47	- 54	0	K.TMVSAPAR.R
832.4806	831.4734	831.4272	0.0461	47	- 54	0	K.TMVSAPAR.R
892.4161	891.4088	891.4198	-0.0110	84	- 90	0	R.YRPGEDR.N
1252.6824	1251.6752	1251.7009	-0.0257	108	- 118	1	R.YGVGMISLKLR.Q + Oxidation (M)
1334.6923	1333.6850	1333.6846	0.0004	70	- 83	0	R.CALAAIGMSASALR.Y
2109.0717	2108.0644	2107.9817	0.0828	13	- 32	1	R.CQAVQGPGGREHATEEVAGR.A + Carbamidomethyl (C)
2465.4048	2464.3975	2464.1838	0.2137	60	- 83	1	R.EWIEGGASERCALAAIGMSASALR.Y + Oxidation (M)

No match to: 700.3201, 713.4158, 731.2732, 781.5109, 831.5215, 833.5146, 839.1638, 845.1403, 845.2394, 855.1207, 861.1503, 877.1304, 893.0865, 1012.6671, 1066.1318, 1157.7036, 1158.7027, 1213.7329, 1226.6034, 1235.6752, 1257.6881, 1282.6333, 1283.6610, 1284.7370, 1296.7999, 1299.6611, 1301.7377, 1317.6990, 1318.7017, 1323.6815, 1380.7924, 1386.7853, 1400.7405, 1427.7313, 1467.8666, 1585.9024, 1612.8562, 1650.8940, 1690.7322, 1707.0040, 1723.0042, 1724.0353, 1815.9485, 1878.1647

40. AAF47775 **Mass:** 144797 **Score:** 44 **Expect:** 1.3e+02 **Queries matched:** 15

AE003478 NID: - Drosophila melanogaster

Observed	Mr(expt)	Mr(calc)	Delta	Start	End	Miss	Peptide
700.3201	699.3128	699.3704	-0.0576	727	- 731	1	R.YSFRK.Q
832.4806	831.4734	831.4814	-0.0080	1064	- 1070	1	R.ESRLLSK.L
833.5146	832.5074	832.4290	0.0784	1179	- 1185	0	R.SSEEILR.M
892.4161	891.4088	891.4524	-0.0436	574	- 581	0	R.LGCFEVPK.E
1012.6671	1011.6598	1011.6328	0.0270	384	- 392	1	R.AIKITPLEK.N
1158.7027	1157.6954	1157.6291	0.0662	234	- 243	1	K.EIEKAVDNLK.N
1213.7329	1212.7256	1212.6574	0.0682	969	- 979	1	K.RSQVVLSGDPR.Q
1226.6034	1225.5961	1225.5860	0.0102	1	- 12	0	-.MSSEASGSITIK.E + Oxidation (M)
1252.6824	1251.6752	1251.5805	0.0947	757	- 766	0	R.ENPQLDVYMK.D + Oxidation (M)
1296.7999	1295.7926	1295.7449	0.0477	555	- 566	0	K.VDVIPGQQIVTK.R
1317.6990	1316.6917	1316.7088	-0.0171	322	- 332	1	K.LFPTRIQEGEK.C
1318.7017	1317.6944	1317.7292	-0.0348	387	- 398	1	K.ITPLEKNFGATK.L
1386.7853	1385.7780	1385.7271	0.0509	525	- 537	1	K.RMIAAEALMAPGR.N
1585.9024	1584.8952	1584.8333	0.0618	989	- 1002	1	R.IASKMGFSISFLER.L
2109.0717	2108.0644	2107.9296	0.1349	72	- 88	0	K.ELSYLSETDWMYESLDK.K

No match to: 713.4158, 731.2732, 781.5109, 831.5215, 832.3782, 839.1638, 845.1403, 845.2394, 855.1207, 861.1503, 877.1304, 893.0865, 1066.1318, 1157.7036, 1235.6752, 1257.6881, 1282.6333, 1283.6610, 1284.7370, 1299.6611, 1301.7377, 1323.6815, 1334.6923, 1380.7924, 1400.7405, 1427.7313, 1467.8666, 1612.8562, 1650.8940, 1690.7322, 1707.0040, 1723.0042, 1724.0353, 1815.9485, 1878.1647, 2465.4048

41. Q3IJD7_PSEHT **Mass:** 19651 **Score:** 44 **Expect:** 1.4e+02 **Queries matched:** 6

Hypothetical protein.- Pseudoalteromonas haloplanktis (strain TAC 125).

Observed	Mr(expt)	Mr(calc)	Delta	Start	End	Miss	Peptide
713.4158	712.4085	712.4344	-0.0258	10	- 15	1	K.KPANRK.K
832.4806	831.4734	831.4966	-0.0233	143	- 149	1	K.VRLGPYK.T
1235.6752	1234.6679	1234.6557	0.0122	110	- 120	1	R.TYPQAESLKAK.I
1317.6990	1316.6917	1316.5641	0.1276	99	- 109	0	K.GPYVMQCGSFR.T + Carbamidomethyl (C); Oxidation (M)
1318.7017	1317.6944	1317.6903	0.0041	164	- 175	1	R.VKIVGCGIWGWT.-
1723.0042	1721.9970	1721.8947	0.1022	85	- 98	1	R.NHEIQVEVKELEQK.G

No match to: 700.3201, 731.2732, 781.5109, 831.5215, 832.3782, 833.5146, 839.1638, 845.1403, 845.2394, 855.1207, 861.1503, 877.1304, 892.4161, 893.0865, 1012.6671, 1066.1318, 1157.7036, 1158.7027, 1213.7329, 1226.6034, 1252.6824, 1257.6881, 1282.6333, 1283.6610, 1284.7370, 1296.7999, 1299.6611, 1301.7377, 1323.6815, 1334.6923, 1380.7924, 1386.7853, 1400.7405, 1427.7313, 1467.8666, 1585.9024, 1612.8562, 1650.8940, 1690.7322, 1707.0040, 1724.0353, 1815.9485, 1878.1647, 2109.0717, 2465.4048

42. Q4LGD1_9BURK **Mass:** 20049 **Score:** 44 **Expect:** 1.4e+02 **Queries matched:** 7

Ribosomal protein L5:Ribosomal protein L5.- Burkholderia cenocepacia HI2424.

Observed	Mr(expt)	Mr(calc)	Delta	Start	End	Miss	Peptide
1157.7036	1156.6964	1156.7080	-0.0117	103	- 112	1	R.FVTVALPRVR.D
1257.6881	1256.6809	1256.5859	0.0949	93	- 102	0	R.GVQMFEFLDR.F + Oxidation (M)
1282.6333	1281.6260	1281.6176	0.0085	20	- 30	0	K.FGYTSPMQVPR.L
1296.7999	1295.7926	1295.7390	0.0536	168	- 178	1	K.ALLTGFRFPFK.N
1299.6611	1298.6538	1298.6652	-0.0114	48	- 59	1	K.KVMDHAVSDLGK.I
1334.6923	1333.6850	1333.6482	0.0368	81	- 92	0	R.ENQAIGCMVTLR.G

1386.7853	1385.7780	1385.8064	-0.0284	60 -	72	1	K.IAGQKPVVTMSKK.A

No match to: 700.3201, 713.4158, 731.2732, 781.5109, 831.5215, 832.3782, 832.4806, 833.5146, 839.1638, 845.1403, 845.2394, 855.1207, 861.1503, 877.1304, 892.4161, 893.0865, 1012.6671, 1066.1318, 1158.7027, 1213.7329, 1226.6034, 1235.6752, 1252.6824, 1283.6610, 1284.7370, 1301.7377, 1317.6990, 1318.7017, 1323.6815, 1380.7924, 1400.7405, 1427.7313, 1467.8666, 1585.9024, 1612.8562, 1650.8940, 1690.7322, 1707.0040, 1723.0042, 1724.0353, 1815.9485, 1878.1647, 2109.0717, 2465.4048

43. Q1INB3_ACIBL **Mass:** 14018 **Score:** 44 **Expect:** 1.4e+02 **Queries matched:** 5

PilT protein-like.- Acidobacteria bacterium (strain Ellin345).

Observed	Mr(expt)	Mr(calc)	Delta	Start	End	Miss	Peptide
1283.6610	1282.6537	1282.7245	-0.0708	19 -	30	0	R.LSAPATLVIDQR.G
1380.7924	1379.7851	1379.7673	0.0178	118 -	129	1	R.LIRASNAVHTIW.-
1427.7313	1426.7240	1426.7343	-0.0103	55 -	67	0	R.LSYAGTIDAFLEK.L
1724.0353	1723.0280	1722.9239	0.1041	1 -	16	0	-.MIVLDTHVALWAAGAR.E
1878.1647	1877.1575	1877.0271	0.1304	2 -	18	1	M.IVLDTHVALWAAGARER.L

No match to: 700.3201, 713.4158, 731.2732, 781.5109, 831.5215, 832.3782, 832.4806, 833.5146, 839.1638, 845.1403, 845.2394, 855.1207, 861.1503, 877.1304, 892.4161, 893.0865, 1012.6671, 1066.1318, 1157.7036, 1158.7027, 1213.7329, 1226.6034, 1235.6752, 1252.6824, 1257.6881, 1282.6333, 1284.7370, 1296.7999, 1299.6611, 1301.7377, 1317.6990, 1318.7017, 1323.6815, 1334.6923, 1386.7853, 1400.7405, 1467.8666, 1585.9024, 1612.8562, 1650.8940, 1690.7322, 1707.0040, 1723.0042, 1815.9485, 2109.0717, 2465.4048

44. Q2WJV1_CLOBE **Mass:** 38673 **Score:** 44 **Expect:** 1.4e+02 **Queries matched:** 8

Glycosyltransferase.- Clostridium beijerincki NCIMB 8052.

Observed	Mr(expt)	Mr(calc)	Delta	Start	End	Miss	Peptide
1226.6034	1225.5961	1225.5947	0.0014	172 -	182	0	R.NFVTGCAMIVR.S + Oxidation (M)
1282.6333	1281.6260	1281.6638	-0.0378	114 -	124	1	K.DKISIIMDGFK.E + Oxidation (M)
1283.6610	1282.6537	1282.6162	0.0375	172 -	182	0	R.NFVTGCAMIVR.S + Carbamidomethyl (C); Oxidation (M)
1400.7405	1399.7332	1399.6918	0.0414	69 -	79	0	K.CITNFNYTVIR.G + Carbamidomethyl (C)
1585.9024	1584.8952	1584.8082	0.0870	69 -	81	1	K.CITNFNYTVIRGK.T + Carbamidomethyl (C)
1724.0353	1723.0280	1722.8909	0.1371	172 -	187	1	R.NFVTGCAMIVRSNIAK.K
1815.9485	1814.9412	1814.9275	0.0138	224 -	239	1	R.QHNSNQTGILKGIYDK.N
2109.0717	2108.0644	2108.1631	-0.0987	305 -	322	0	K.ASIVLEIFLPIMPEFVFK.Y + Oxidation (M)

No match to: 700.3201, 713.4158, 731.2732, 781.5109, 831.5215, 832.3782, 832.4806, 833.5146, 839.1638, 845.1403, 845.2394, 855.1207, 861.1503, 877.1304, 892.4161, 893.0865, 1012.6671, 1066.1318, 1157.7036, 1158.7027, 1213.7329, 1235.6752, 1252.6824, 1257.6881, 1284.7370, 1296.7999, 1299.6611, 1301.7377, 1317.6990, 1318.7017, 1323.6815, 1334.6923, 1380.7924, 1386.7853, 1427.7313, 1467.8666, 1612.8562, 1650.8940, 1690.7322, 1707.0040, 1723.0042, 1878.1647, 2465.4048

45. AAM79212 **Mass:** 22460 **Score:** 44 **Expect:** 1.4e+02 **Queries matched:** 7

AE014074 NID: - Streptococcus pyogenes MGAS315

Observed	Mr(expt)	Mr(calc)	Delta	Start	End	Miss	Peptide
1158.7027	1157.6954	1157.6516	0.0438	54 -	64	1	K.NLARTSSKPGK.T
1235.6752	1234.6679	1234.7033	-0.0354	43 -	53	1	K.SSFINTILGRK.N
1301.7377	1300.7304	1300.6009	0.1295	128 -	137	0	K.EDIQMYDFLK.Y
1317.6990	1316.6917	1316.5958	0.0959	128 -	137	0	K.EDIQMYDFLK.Y + Oxidation (M)
1380.7924	1379.7851	1379.7700	0.0151	138 -	149	0	K.YYDIPVIVVATK.A
1386.7853	1385.7780	1385.6826	0.0954	174 -	185	0	K.SDTFIVFSSVER.I
1467.8666	1466.8594	1466.7405	0.1188	65 -	76	0	K.TQLLNFFNIDDK.L

No match to: 700.3201, 713.4158, 731.2732, 781.5109, 831.5215, 832.3782, 832.4806, 833.5146, 839.1638, 845.1403, 845.2394, 855.1207, 861.1503, 877.1304, 892.4161, 893.0865, 1012.6671, 1066.1318, 1157.7036, 1213.7329, 1226.6034, 1252.6824, 1257.6881, 1282.6333, 1283.6610, 1284.7370, 1296.7999, 1299.6611, 1318.7017, 1323.6815, 1334.6923, 1400.7405, 1427.7313, 1585.9024, 1612.8562, 1650.8940, 1690.7322, 1707.0040, 1723.0042, 1724.0353, 1815.9485, 1878.1647, 2109.0717, 2465.4048

46. B70318 **Mass:** 51423 **Score:** 43 **Expect:** 1.5e+02 **Queries matched:** 9

chaperone HslU - Aquifex aeolicus

Observed	Mr(expt)	Mr(calc)	Delta	Start	End	Miss	Peptide
713.4158	712.4085	712.4595	-0.0510	35 -	41	0	K.AVAIALR.N
832.3782	831.3709	831.4086	-0.0377	172 -	178	1	R.KGELDDR.I
832.4806	831.4734	831.4086	0.0647	172 -	178	1	R.KGELDDR.I
1213.7329	1212.7256	1212.7594	-0.0338	79 -	89	1	R.LANLIKAPFVK.V
1226.6034	1225.5961	1225.6918	-0.0956	350 -	360	0	R.ILVEPENALTK.Q
1467.8666	1466.8594	1466.7589	0.1004	272 -	286	1	K.TPGAGPGVSREGVQR.D
1585.9024	1584.8952	1584.8147	0.0805	90 -	103	1	K.VEATKYTEIGYVGR.D
1724.0353	1723.0280	1722.8610	0.1670	240 -	253	1	K.LIDMEEVAREAIYR.A + Oxidation (M)
2465.4048	2464.3975	2464.2882	0.1093	186 -	208	1	K.EKTVPMVGIAGPPGLEELENQIK.E + Oxidation (M)

No match to: 700.3201, 731.2732, 781.5109, 831.5215, 833.5146, 839.1638, 845.1403, 845.2394, 855.1207, 861.1503, 877.1304, 892.4161, 893.0865, 1012.6671, 1066.1318, 1157.7036, 1158.7027, 1235.6752, 1252.6824, 1257.6881, 1282.6333, 1283.6610, 1284.7370, 1296.7999, 1299.6611, 1301.7377, 1317.6990, 1318.7017, 1323.6815, 1334.6923, 1380.7924, 1386.7853, 1400.7405, 1427.7313, 1612.8562, 1650.8940, 1690.7322, 1707.0040, 1723.0042, 1815.9485, 1878.1647, 2109.0717

47. P79372_PIG **Mass:** 11271 **Score:** 43 **Expect:** 1.5e+02 **Queries matched:** 5

Na+/Ca2+ exchanger isoform NACA3 protein (Fragment).- Sus scrofa (Pig).

Observed	Mr(expt)	Mr(calc)	Delta	Start	End	Miss	Peptide
1252.6824	1251.6752	1251.5805	0.0947	1 -	10	0	-.MEFQNDEIVK.I
1380.7924	1379.7851	1379.6642	0.1209	25 -	36	0	K.ECSFSLVLEEPK.W
1585.9024	1584.8952	1584.7922	0.1029	82 -	94	1	K.LEVIIEESYEFKS.-

1690.7322	1689.7249	1689.7555	-0.0307	41 -	55	1	R.GMKGGFTLTEEYDDK.Q
1724.0353	1723.0280	1722.9199	0.1081	67 -	81	1	R.RIAEMGRPILGEHTK.L + Oxidation (M)

No match to: 700.3201, 713.4158, 731.2732, 781.5109, 831.5215, 832.3782, 832.4806, 833.5146, 839.1638, 845.1403, 845.2394, 855.1207, 861.1503, 877.1304, 892.4161, 893.0865, 1012.6671, 1066.1318, 1157.7036, 1158.7027, 1213.7329, 1226.6034, 1235.6752, 1257.6881, 1282.6333, 1283.6610, 1284.7370, 1296.7999, 1299.6611, 1301.7377, 1317.6990, 1318.7017, 1323.6815, 1334.6923, 1386.7853, 1400.7405, 1427.7313, 1467.8666, 1612.8562, 1650.8940, 1707.0040, 1723.0042, 1815.9485, 1878.1647, 2109.0717, 2465.4048

48. E69296 **Mass:** 20123 **Score:** 43 **Expect:** 1.6e+02 **Queries matched:** 7

transcription initiation factor IID homolog - Archaeoglobus fulgidus

Observed	Mr(expt)	Mr(calc)	Delta	Start	End	Miss	Peptide
700.3201	699.3128	699.2898	0.0230	1 -	5	0	-.MQDYK.I + Oxidation (M)
832.3782	831.3709	831.4198	-0.0489	69 -	75	1	K.SVEDARR.A
832.4806	831.4734	831.4198	0.0535	69 -	75	1	K.SVEDARR.A
1158.7027	1157.6954	1157.6920	0.0034	39 -	48	1	K.QFPGLVLRTK.E
1427.7313	1426.7240	1426.7555	-0.0314	86 -	98	0	K.EIGISVIDEPEVK.V
1707.0040	1705.9967	1705.9799	0.0167	145 -	161	1	R.VVVLIFGSGKMVVTGGK.S + Oxidation (M)
1815.9485	1814.9412	1814.9699	-0.0287	83 -	98	1	K.MLKEIGISVIDEPEVK.V + Oxidation (M)

No match to: 713.4158, 731.2732, 781.5109, 831.5215, 833.5146, 839.1638, 845.1403, 845.2394, 855.1207, 861.1503, 877.1304, 892.4161, 893.0865, 1012.6671, 1066.1318, 1157.7036, 1213.7329, 1226.6034, 1235.6752, 1252.6824, 1257.6881, 1282.6333, 1283.6610, 1284.7370, 1296.7999, 1299.6611, 1301.7377, 1317.6990, 1318.7017, 1323.6815, 1334.6923, 1380.7924, 1386.7853, 1400.7405, 1467.8666, 1585.9024, 1612.8562, 1650.8940, 1690.7322, 1723.0042, 1724.0353, 1878.1647, 2109.0717, 2465.4048

49. Q8T530_PLAFA **Mass:** 14687 **Score:** 43 **Expect:** 1.6e+02 **Queries matched:** 5

Erythrocyte membrane protein 1 (Fragment).- Plasmodium falciparum.

Observed	Mr(expt)	Mr(calc)	Delta	Start	End	Miss	Peptide
831.5215	830.5142	830.4498	0.0644	25 -	31	0	K.QLQDSLK.N
1282.6333	1281.6260	1281.6717	-0.0457	8 -	18	1	R.GKDLFIGYNQK.D
1427.7313	1426.7240	1426.6510	0.0730	82 -	95	1	K.AITCGAGEGDRYSK.Y
1585.9024	1584.8952	1584.8372	0.0580	32 -	45	1	K.NIFAKIHSEVTNGR.T
1650.8940	1649.8868	1649.7831	0.1037	77 -	92	1	R.STVWKAITCGAGEGDR.Y

No match to: 700.3201, 713.4158, 731.2732, 781.5109, 832.3782, 832.4806, 833.5146, 839.1638, 845.1403, 845.2394, 855.1207, 861.1503, 877.1304, 892.4161, 893.0865, 1012.6671, 1066.1318, 1157.7036, 1158.7027, 1213.7329, 1226.6034, 1235.6752, 1252.6824, 1257.6881, 1283.6610, 1284.7370, 1296.7999, 1299.6611, 1301.7377, 1317.6990, 1318.7017, 1323.6815, 1334.6923, 1380.7924, 1386.7853, 1400.7405, 1467.8666, 1612.8562, 1690.7322, 1707.0040, 1723.0042, 1724.0353, 1815.9485, 1878.1647, 2109.0717, 2465.4048

50. Q1JM76_STRPC **Mass:** 22493 **Score:** 43 **Expect:** 1.6e+02 **Queries matched:** 7

GTP-binding protein YihA.- Streptococcus pyogenes serotype M3 (strain MGAS9429).

Observed	Mr(expt)	Mr(calc)	Delta	Start	End	Miss	Peptide
1158.7027	1157.6954	1157.6516	0.0438	54 -	64	1	K.NLARTSSKPGK.T
1235.6752	1234.6679	1234.7033	-0.0354	43 -	53	1	K.SSFINTILGRK.N
1301.7377	1300.7304	1300.6009	0.1295	128 -	137	0	K.EDIQMYDFLK.Y
1317.6990	1316.6917	1316.5958	0.0959	128 -	137	0	K.EDIQMYDFLK.Y + Oxidation (M)
1380.7924	1379.7851	1379.7700	0.0151	138 -	149	0	K.YYDIPVIVVATK.A
1386.7853	1385.7780	1385.6826	0.0954	174 -	185	0	K.SDTFIVFSSVER.I
1467.8666	1466.8594	1466.7405	0.1188	65 -	76	0	K.TQLLNFFNIDDK.L

No match to: 700.3201, 713.4158, 731.2732, 781.5109, 831.5215, 832.3782, 832.4806, 833.5146, 839.1638, 845.1403, 845.2394, 855.1207, 861.1503, 877.1304, 892.4161, 893.0865, 1012.6671, 1066.1318, 1157.7036, 1213.7329, 1226.6034, 1252.6824, 1257.6881, 1282.6333, 1283.6610, 1284.7370, 1296.7999, 1299.6611, 1318.7017, 1323.6815, 1334.6923, 1400.7405, 1427.7313, 1585.9024, 1612.8562, 1650.8940, 1690.7322, 1707.0040, 1723.0042, 1724.0353, 1815.9485, 1878.1647, 2109.0717, 2465.4048

Search Parameters

Type of search : Peptide Mass Fingerprint
Enzyme : Trypsin
Variable modifications : Carbamidomethyl (C),Oxidation (M)
Mass values : Monoisotopic
Protein Mass : Unrestricted
Peptide Mass Tolerance : ± 100 ppm
Peptide Charge State : 1+
Max Missed Cleavages : 1
Number of queries : 51

Mascot: http://www.matrixscience.com/
