# Supplementary material for: A proteomic strategy to identify novel serum biomarkers for liver cirrhosis and hepatocellular cancer in individuals with fatty liver disease
Source: BMC Cancer. 2009 Aug 5;9:271. doi: 10.1186/1471-2407-9-271 (PMC2729079; doi:10.1186/1471-2407-9-271)
Supplement: Additional File 8 — Spot 4 is ApoA4. The protein summary report for spot 4, generated using Mascot Peptide Mass Fingerprint search program (Matrix Science Ltd), identifies it as ApoA4. [file 1471-2407-9-271-S8.pdf]

Mascot Search Results

```
User          : Joe Gray
Email         : joe.gray@ncl.ac.uk
Search title  : JG_3_0001.dat - SpecView
Database      : MSDB 20060831 (3239079 sequences; 1079594700 residues)
Timestamp     : 12 Jan 2007 at 12:24:13 GMT
Top Score     : 338 for AAA51748, HUMAPOAIV NID:  - Homo sapiens
```

Probability Based Mowse Score

Protein score is -10*Log(P), where P is the probability that the observed match is a random event.
Protein scores greater than 78 are significant (p<0.05).

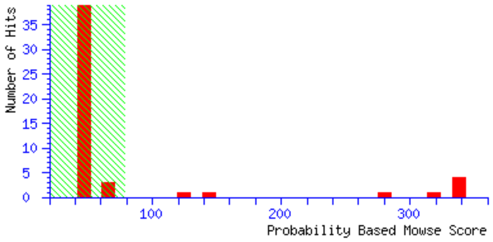

Protein Summary Report

Format As | Protein Summary | Help

Significance threshold p< 0.05 | Max. number of hits 50

Re-Search All | Search Unmatched

Index

	Accession	Mass	Score	Description
1.	AAA51748	43358	338	HUMAPOAIV NID: - Homo sapiens
2.	CAB58685	43507	335	SEQUENCE 1 FROM PATENT WO9315198.- unidentified.
3.	AAQ91809	45371	332	AY422950 NID: - Homo sapiens
4.	AAA96731	45353	332	HUMAPOA4A NID: - Homo sapiens
5.	LPHUA4	45307	315	apolipoprotein A-IV precursor [validated] - human
6.	Q13784_HUMAN	28141	280	APOA4 protein (Fragment).- Homo sapiens (Human).
7.	A47141	46510	139	apolipoprotein A-IV I isoform - baboon (fragment)
8.	S29565	49846	119	apolipoprotein A-IV - crab-eating macaque
9.	C87410	70844	64	iolC protein [imported] - Caulobacter crescentus
10.	Q3SQM9_NITWN	41297	62	Acyl-CoA dehydrogenase (EC 1.3.99.2).- Nitrobacter winogradskyi (strain Nb-255 / ATCC 25391).
11.	I54248	10612	62	apolipoprotein A-IV - chimpanzee (fragment)
12.	Q6WAZ7_TRYCR	82415	56	C71 surface protein.- Trypanosoma cruzi.
13.	Q3XUS9_9PROT	61027	56	Coenzyme B12-binding:Radical SAM.- Magnetococcus sp. MC-1.
14.	Q4USB5_XANC8	13788	56	HrpB2 protein.- Xanthomonas campestris pv. campestris (strain 8004).
15.	Q4D9P5_TRYCR	126270	54	DNA-directed RNA polymerase III subunit, putative (EC 2.7.7.6).- Trypanosoma cruzi.
16.	C83690	22489	53	hypothetical protein BH0323 [imported] - Bacillus halodurans (strain C-125)
17.	Q56EH5_9CAUD	36302	52	Hypothetical protein PHG31ORF188c.- Aeromonas phage 31.
18.	Q4MZD6_THEPA	62104	52	Hypothetical protein.- Theileria parva.
19.	Q4JCL5_SULAC	24648	52	Conserved protein.- Sulfolobus acidocaldarius.
20.	Q8SPT5_MACMU	16793	51	Growth differentiation factor 9 (Fragment).- Macaca mulatta (Rhesus macaque).
21.	A44277	64504	51	nonstructural protein NS1 - bluetongue virus (serotype 2)
22.	T50803	83475	51	hypothetical protein T30N20_210 - Arabidopsis thaliana
23.	Q94BQ3_ARATH	83778	51	Hypothetical protein At5g10940.- Arabidopsis thaliana (Mouse-ear cress).
24.	Q6Q539_YEAST	24211	50	YDR226W.- Saccharomyces cerevisiae (Baker's yeast).
25.	Q2BL66_9GAMM	52033	50	Argininosuccinate lyase.- Oceanospirillum sp. MED92.

**26.**	Q4IVR0_AZOVI	36505	50	Regulatory protein, ArsR.- Azotobacter vinelandii AvOP.
**27.**	Q1RSK3_MEDTR	105088	50	Zinc finger, RING-type; Transcription factor jumonji, jmjC.- Medicago truncatula (Barrel medic).
**28.**	Q1ZTR3_9VIBR	31022	50	Putative putative phospholipid biosynthesis acyltransferase.- Vibrio angustum S14.
**29.**	Q390G0_BURS3	100380	50	ATP-dependent transcriptional regulator, MalT-like, LuxR family.- Burkholderia sp. (strain 383) (Burkholderia cepacia (strain ATCC 17760 / NCIB 9086 / R18194))
**30.**	Q8TA35_HETGL	25472	50	Dorsal gland cell protein Hgg-26 (Fragment).- Heterodera glycines (Soybean cyst nematode worm).
**31.**	Q7XUX8_ORYSA	41458	50	OSJNBa0027G07.11 protein.- Oryza sativa (japonica cultivar-group).
**32.**	Q6IQ22_HUMAN	11751	50	RAB12, member RAS oncogene family.- Homo sapiens (Human).
**33.**	AH3404	20696	49	hypothetical protein BMEI1222 [imported] - Brucella melitensis (strain 16M)
**34.**	AAN43115	54614	49	AE005674 NID: - Shigella flexneri 2a str. 301
**35.**	Q2RNF9_RHORT	80918	49	Malate dehydrogenase (EC 1.1.1.40) (EC 2.3.1.8).- Rhodospirillum rubrum (strain ATCC 11170 / NCIB 8255).
**36.**	T08180	65798	49	PF20 protein, microtubule-associated - Chlamydomonas reinhardtii
**37.**	Q7V0J9_PROMP	38698	48	Possible dTDP-glucose 4,6-dehydratase (EC 4.2.1.46).- Prochlorococcus marinus subsp. pastoris (strain CCMP 1378 / MED4).
**38.**	Q5ZI47_CHICK	101099	48	Hypothetical protein.- Gallus gallus (Chicken).
**39.**	Q4DLZ9_TRYCR	19461	48	Protein tyrosine phosphatase-like protein, putative.- Trypanosoma cruzi.
**40.**	Q39YY1_GEOMG	16521	48	Putative PAS/PAC sensor protein.- Geobacter metallireducens (strain GS-15 / ATCC 53774 / DSM 7210).
**41.**	Q9TUL8_HORSE	42919	48	Matrix metalloproteinase-2 (Fragment).- Equus caballus (Horse).
**42.**	Q3M485_ANAVT	42516	48	Hydrogenase formation HypD protein.- Anabaena variabilis (strain ATCC 29413 / PCC 7937).
**43.**	Q5NVL1_PONPY	79482	48	Hypothetical protein DKFZp459N0220 (Fragment).- Pongo pygmaeus (Orangutan).
**44.**	Q4AI58_9CHLB	37062	48	Cell shape determining protein MreB/Mrl.- Chlorobium phaeobacteroides BS1.
**45.**	Q2HUK6_MEDTR	39508	47	Mitochondrial substrate carrier.- Medicago truncatula (Barrel medic).
**46.**	Q5ZF44_MEDTR	39616	47	Mitochondrial phosphate translocator.- Medicago truncatula (Barrel medic).
**47.**	Q5R9R8_PONPY	72095	47	Hypothetical protein DKFZp469P0412.- Pongo pygmaeus (Orangutan).
**48.**	Q4CFZ5_CLOTM	11661	47	Hypothetical protein.- Clostridium thermocellum ATCC 27405.
**49.**	Q2DM63_9DELT	19010	47	Hypothetical protein precursor.- Geobacter uraniumreducens Rf4.
**50.**	Q7P987_RICSI	73246	47	Propionyl-CoA carboxylase alpha chain.- Rickettsia sibirica 246.

Results List

1. AAA51748 **Mass:** 43358 **Score:** 338 **Expect:** 5.1e-28 **Queries matched:** 31

HUMAPOAIV NID: - Homo sapiens

Observed	Mr(expt)	Mr(calc)	Delta	Start	End	Miss	Peptide
975.4483	974.4410	974.5032	-0.0623	236 -	244	0	R.ISASAEELR.Q
983.4562	982.4489	982.5447	-0.0958	247 -	255	0	R.LAPLAEDVR.G
1076.4495	1075.4422	1075.5338	-0.0916	331 -	339	0	K.VNSFFSTFK.E
1083.4282	1082.4209	1082.5284	-0.1075	181 -	189	0	R.LTPYADEFK.V
1104.4679	1103.4606	1103.5611	-0.1005	115 -	123	0	R.LEPYADQLR.T
1132.5159	1131.5087	1131.6149	-0.1062	135 -	143	1	R.RQLTPYAQR.M
1196.4419	1195.4346	1195.5509	-0.1163	287 -	296	0	R.VEPYGENFNK.A
1215.5637	1214.5564	1214.6441	-0.0877	297 -	306	0	K.ALVQQMEQLR.T
1231.5737	1230.5665	1230.6390	-0.0726	297 -	306	0	K.ALVQQMEQLR.T + Oxidation (M)
1235.5752	1234.5679	1234.6669	-0.0990	93 -	103	0	R.LLPHANEVSQK.I
1258.5780	1257.5707	1257.6677	-0.0970	192 -	201	1	K.IDQTVEELRR.S
1287.5711	1286.5638	1286.6579	-0.0940	124 -	134	0	R.TQVNTQAEQLR.R
1300.6026	1299.5954	1299.6782	-0.0829	170 -	180	1	K.IDQNVEELKGR.L
1310.5953	1309.5880	1309.6918	-0.1038	181 -	191	1	R.LTPYADEFKVK.I
1311.6184	1310.6111	1310.6982	-0.0871	60 -	70	0	K.LVPFATELHER.L
1319.5820	1318.5747	1318.6557	-0.0810	329 -	339	1	R.DKVNSFFSTFK.E
1350.5619	1349.5546	1349.6463	-0.0917	202 -	213	0	R.SLAPYAQDTQEK.L
1352.5712	1351.5639	1351.6520	-0.0881	286 -	296	1	R.RVEPYGENFNK.A
1407.6373	1406.6300	1406.7041	-0.0741	46 -	58	0	K.LGEVNTYAGDLQK.K
1439.7198	1438.7125	1438.7932	-0.0807	59 -	70	1	K.KLVPFATELHER.L
1443.6886	1442.6813	1442.7590	-0.0776	124 -	135	1	R.TQVNTQAEQLRR.Q
1558.7275	1557.7202	1557.7973	-0.0771	214 -	226	0	K.LNHQLEGLTFQMK.K
1574.7196	1573.7123	1573.7922	-0.0799	214 -	226	0	K.LNHQLEGLTFQMK.K + Oxidation (M)
1634.7576	1633.7503	1633.8311	-0.0808	32 -	45	0	K.SELTQQLNALFQDK.L
1805.8453	1804.8380	1804.9107	-0.0727	309 -	325	0	K.LGPHAGDVEGHLSFLEK.D
1927.9091	1926.9019	1926.9435	-0.0416	268 -	284	0	K.SLAELGGHLDQQVEEFR.R
1993.9450	1992.9377	1992.9864	-0.0487	150 -	167	0	R.ENADSLQASLRPHADELK.A
2084.0093	2083.0020	2083.0446	-0.0426	268 -	285	1	K.SLAELGGHLDQQVEEFRR.R
2193.1061	2192.0988	2192.1185	-0.0197	150 -	169	1	R.ENADSLQASLRPHADELKAK.I
2362.2237	2361.2164	2361.2400	-0.0236	147 -	167	1	R.VLRENADSLQASLRPHADELK.A
2890.4720	2889.4647	2889.4330	0.0317	202 -	226	1	R.SLAPYAQDTQEKLNHQLEGLTFQMK.K

No match to: 854.9449, 860.9786, 876.9413, 959.3966, 976.4208, 980.4021, 1065.9443, 1218.5811, 1297.5441, 1336.3908, 1427.4641, 1485.7681, 1499.7430, 1508.6825, 1585.8063, 1656.7520, 1707.7763, 1834.8681, 1976.9265, 2045.0624, 2067.6716, 2069.7862, 2211.0997

2. CAB58685 **Mass:** 43507 **Score:** 335 **Expect:** 1e-27 **Queries matched:** 31

SEQUENCE 1 FROM PATENT WO9315198.- unidentified.

Observed	Mr(expt)	Mr(calc)	Delta	Start	End	Miss	Peptide

975.4483	974.4410	974.5032	-0.0623	237 -	245	0	R.ISASAEELR.Q
983.4562	982.4489	982.5447	-0.0958	248 -	256	0	R.LAPLAEDVR.G
1076.4495	1075.4422	1075.5338	-0.0916	332 -	340	0	K.VNSFFSTFK.E
1083.4282	1082.4209	1082.5284	-0.1075	182 -	190	0	R.LTPYADEFK.V
1104.4679	1103.4606	1103.5611	-0.1005	116 -	124	0	R.LEPYADQLR.T
1132.5159	1131.5087	1131.6149	-0.1062	136 -	144	1	R.RQLTPYAQR.M
1196.4419	1195.4346	1195.5509	-0.1163	288 -	297	0	R.VEPYGENFNK.A
1215.5637	1214.5564	1214.6441	-0.0877	298 -	307	0	K.ALVQQMEQLR.Q
1231.5737	1230.5665	1230.6390	-0.0726	298 -	307	0	K.ALVQQMEQLR.Q + Oxidation (M)
1235.5752	1234.5679	1234.6669	-0.0990	94 -	104	0	R.LLPHANEVSQK.I
1258.5780	1257.5707	1257.6677	-0.0970	193 -	202	1	K.IDQTVEELRR.S
1287.5711	1286.5638	1286.6579	-0.0940	125 -	135	0	R.TQVNTQAEQLR.R
1300.6026	1299.5954	1299.6782	-0.0829	171 -	181	1	K.IDQNVEELKGR.L
1310.5953	1309.5880	1309.6918	-0.1038	182 -	192	1	R.LTPYADEFKVK.I
1311.6184	1310.6111	1310.6982	-0.0871	61 -	71	0	K.LVPFATELHER.L
1319.5820	1318.5747	1318.6557	-0.0810	330 -	340	1	R.DKVNSFFSTFK.E
1350.5619	1349.5546	1349.6463	-0.0917	203 -	214	0	R.SLAPYAQDTQEK.L
1352.5712	1351.5639	1351.6520	-0.0881	287 -	297	1	R.RVEPYGENFNK.A
1407.6373	1406.6300	1406.7041	-0.0741	47 -	59	0	K.LGEVNTYAGDLQK.K
1439.7198	1438.7125	1438.7932	-0.0807	60 -	71	1	K.KLVPFATELHER.L
1443.6886	1442.6813	1442.7590	-0.0776	125 -	136	1	R.TQVNTQAEQLRR.Q
1558.7275	1557.7202	1557.7973	-0.0771	215 -	227	0	K.LNHQLEGLTFQMK.K
1574.7196	1573.7123	1573.7922	-0.0799	215 -	227	0	K.LNHQLEGLTFQMK.K + Oxidation (M)
1634.7576	1633.7503	1633.8311	-0.0808	33 -	46	0	K.SELTQQLNALFQDK.L
1805.8453	1804.8380	1804.9107	-0.0727	310 -	326	0	K.LGPHAGDVEGHLSFLEK.D
1927.9091	1926.9019	1926.9435	-0.0416	269 -	285	0	K.SLAELGGHLDQQVEEFR.R
1993.9450	1992.9377	1992.9864	-0.0487	151 -	168	0	R.ENADSLQASLRPHADELK.A
2084.0093	2083.0020	2083.0446	-0.0426	269 -	286	1	K.SLAELGGHLDQQVEEFRR.R
2193.1061	2192.0988	2192.1185	-0.0197	151 -	170	1	R.ENADSLQASLRPHADELKAK.I
2362.2237	2361.2164	2361.2400	-0.0236	148 -	168	1	R.VLRENADSLQASLRPHADELK.A
2890.4720	2889.4647	2889.4330	0.0317	203 -	227	1	R.SLAPYAQDTQEKLNHQLEGLTFQMK.K

No match to: 854.9449, 860.9786, 876.9413, 959.3966, 976.4208, 980.4021, 1065.9443, 1218.5811, 1297.5441, 1336.3908, 1427.4641, 1485.7681, 1499.7430, 1508.6825, 1585.8063, 1656.7520, 1707.7763, 1834.8681, 1976.9265, 2045.0624, 2067.6716, 2069.7862, 2211.0997

3. AAQ91809 **Mass:** 45371 **Score:** **332** **Expect:** 2e-27 **Queries matched:** 31

AY422950 NID: - Homo sapiens

Observed	Mr(expt)	Mr(calc)	Delta	Start	End	Miss	Peptide
975.4483	974.4410	974.5032	-0.0623	256 -	264	0	R.ISASAEELR.Q
983.4562	982.4489	982.5447	-0.0958	267 -	275	0	R.LAPLAEDVR.G
1076.4495	1075.4422	1075.5338	-0.0916	351 -	359	0	K.VNSFFSTFK.E
1083.4282	1082.4209	1082.5284	-0.1075	201 -	209	0	R.LTPYADEFK.V
1104.4679	1103.4606	1103.5611	-0.1005	135 -	143	0	R.LEPYADQLR.T
1132.5159	1131.5087	1131.6149	-0.1062	155 -	163	1	R.RQLTPYAQR.M
1196.4419	1195.4346	1195.5509	-0.1163	307 -	316	0	R.VEPYGENFNK.A
1215.5637	1214.5564	1214.6441	-0.0877	317 -	326	0	K.ALVQQMEQLR.Q
1231.5737	1230.5665	1230.6390	-0.0726	317 -	326	0	K.ALVQQMEQLR.Q + Oxidation (M)
1235.5752	1234.5679	1234.6669	-0.0990	113 -	123	0	R.LLPHANEVSQK.I
1258.5780	1257.5707	1257.6677	-0.0970	212 -	221	1	K.IDQTVEELRR.S
1287.5711	1286.5638	1286.6579	-0.0940	144 -	154	0	R.TQVNTQAEQLR.R
1300.6026	1299.5954	1299.6782	-0.0829	190 -	200	1	K.IDQNVEELKGR.L
1310.5953	1309.5880	1309.6918	-0.1038	201 -	211	1	R.LTPYADEFKVK.I
1311.6184	1310.6111	1310.6982	-0.0871	80 -	90	0	K.LVPFATELHER.L
1319.5820	1318.5747	1318.6557	-0.0810	349 -	359	1	R.DKVNSFFSTFK.E
1350.5619	1349.5546	1349.6463	-0.0917	222 -	233	0	R.SLAPYAQDTQEK.L
1352.5712	1351.5639	1351.6520	-0.0881	306 -	316	1	R.RVEPYGENFNK.A
1407.6373	1406.6300	1406.7041	-0.0741	66 -	78	0	K.LGEVNTYAGDLQK.K
1439.7198	1438.7125	1438.7932	-0.0807	79 -	90	1	K.KLVPFATELHER.L
1443.6886	1442.6813	1442.7590	-0.0776	144 -	155	1	R.TQVNTQAEQLRR.Q
1558.7275	1557.7202	1557.7973	-0.0771	234 -	246	0	K.LNHQLEGLTFQMK.K
1574.7196	1573.7123	1573.7922	-0.0799	234 -	246	0	K.LNHQLEGLTFQMK.K + Oxidation (M)
1634.7576	1633.7503	1633.8311	-0.0808	52 -	65	0	K.SELTQQLNALFQDK.L
1805.8453	1804.8380	1804.9107	-0.0727	329 -	345	0	K.LGPHAGDVEGHLSFLEK.D
1927.9091	1926.9019	1926.9435	-0.0416	288 -	304	0	K.SLAELGGHLDQQVEEFR.R
1993.9450	1992.9377	1992.9864	-0.0487	170 -	187	0	R.ENADSLQASLRPHADELK.A
2084.0093	2083.0020	2083.0446	-0.0426	288 -	305	1	K.SLAELGGHLDQQVEEFRR.R
2193.1061	2192.0988	2192.1185	-0.0197	170 -	189	1	R.ENADSLQASLRPHADELKAK.I
2362.2237	2361.2164	2361.2400	-0.0236	167 -	187	1	R.VLRENADSLQASLRPHADELK.A
2890.4720	2889.4647	2889.4330	0.0317	222 -	246	1	R.SLAPYAQDTQEKLNHQLEGLTFQMK.K

No match to: 854.9449, 860.9786, 876.9413, 959.3966, 976.4208, 980.4021, 1065.9443, 1218.5811, 1297.5441, 1336.3908, 1427.4641, 1485.7681, 1499.7430, 1508.6825, 1585.8063, 1656.7520, 1707.7763, 1834.8681, 1976.9265, 2045.0624, 2067.6716, 2069.7862, 2211.0997

4. AAA96731 **Mass:** 45353 **Score:** 332 **Expect:** 2e-27 **Queries matched:** 31

HUMAPOA4A NID: - Homo sapiens

Observed	Mr(expt)	Mr(calc)	Delta	Start		End	Miss	Peptide
975.4483	974.4410	974.5032	-0.0623	256	-	264	0	R.ISASAEELR.Q
983.4562	982.4489	982.5447	-0.0958	267	-	275	0	R.LAPLAEDVR.G
1076.4495	1075.4422	1075.5338	-0.0916	351	-	359	0	K.VNSFFSTFK.E
1083.4282	1082.4209	1082.5284	-0.1075	201	-	209	0	R.LTPYADEFK.V
1104.4679	1103.4606	1103.5611	-0.1005	135	-	143	0	R.LEPYADQLR.T
1132.5159	1131.5087	1131.6149	-0.1062	155	-	163	1	R.RQLTPYAQR.M
1196.4419	1195.4346	1195.5509	-0.1163	307	-	316	0	R.VEPYGENFNK.A
1215.5637	1214.5564	1214.6441	-0.0877	317	-	326	0	K.ALVQQMEQLR.T
1231.5737	1230.5665	1230.6390	-0.0726	317	-	326	0	K.ALVQQMEQLR.T + Oxidation (M)
1235.5752	1234.5679	1234.6669	-0.0990	113	-	123	0	R.LLPHANEVSQK.I
1258.5780	1257.5707	1257.6677	-0.0970	212	-	221	1	K.IDQTVEELRR.S
1287.5711	1286.5638	1286.6579	-0.0940	144	-	154	0	R.TQVNTQAEQLR.R
1300.6026	1299.5954	1299.6782	-0.0829	190	-	200	1	K.IDQNVEELKGR.L
1310.5953	1309.5880	1309.6918	-0.1038	201	-	211	1	R.LTPYADEFKVK.I
1311.6184	1310.6111	1310.6982	-0.0871	80	-	90	0	K.LVPFATELHER.L
1319.5820	1318.5747	1318.6557	-0.0810	349	-	359	1	R.DKVNSFFSTFK.E
1350.5619	1349.5546	1349.6463	-0.0917	222	-	233	0	R.SLAPYAQDTQEK.L
1352.5712	1351.5639	1351.6520	-0.0881	306	-	316	1	R.RVEPYGENFNK.A
1407.6373	1406.6300	1406.7041	-0.0741	66	-	78	0	K.LGEVNTYAGDLQK.K
1439.7198	1438.7125	1438.7932	-0.0807	79	-	90	1	K.KLVPFATELHER.L
1443.6886	1442.6813	1442.7590	-0.0776	144	-	155	1	R.TQVNTQAEQLRR.Q
1558.7275	1557.7202	1557.7973	-0.0771	234	-	246	0	K.LNHQLEGLTFQMK.K
1574.7196	1573.7123	1573.7922	-0.0799	234	-	246	0	K.LNHQLEGLTFQMK.K + Oxidation (M)
1634.7576	1633.7503	1633.8311	-0.0808	52	-	65	0	K.SELTQQLNALFQDK.L
1805.8453	1804.8380	1804.9107	-0.0727	329	-	345	0	K.LGPHAGDVEGHLSFLEK.D
1927.9091	1926.9019	1926.9435	-0.0416	288	-	304	0	K.SLAELGGHLDQQVEEFR.R
1993.9450	1992.9377	1992.9864	-0.0487	170	-	187	0	R.ENADSLQASLRPHADELK.A
2084.0093	2083.0020	2083.0446	-0.0426	288	-	305	1	K.SLAELGGHLDQQVEEFRR.R
2193.1061	2192.0988	2192.1185	-0.0197	170	-	189	1	R.ENADSLQASLRPHADELKAK.I
2362.2237	2361.2164	2361.2400	-0.0236	167	-	187	1	R.VLRENADSLQASLRPHADELK.A
2890.4720	2889.4647	2889.4330	0.0317	222	-	246	1	R.SLAPYAQDTQEKLNHQLEGLTFQMK.K

No match to: 854.9449, 860.9786, 876.9413, 959.3966, 976.4208, 980.4021, 1065.9443, 1218.5811, 1297.5441, 1336.3908, 1427.4641, 1485.7681, 1499.7430, 1508.6825, 1585.8063, 1656.7520, 1707.7763, 1834.8681, 1976.9265, 2045.0624, 2067.6716, 2069.7862, 2211.0997

5. LPHUA4 **Mass:** 45307 **Score:** 315 **Expect:** 1e-25 **Queries matched:** 30

apolipoprotein A-IV precursor [validated] - human

Observed	Mr(expt)	Mr(calc)	Delta	Start		End	Miss	Peptide
975.4483	974.4410	974.5032	-0.0623	256	-	264	0	R.ISASAEELR.Q
983.4562	982.4489	982.5447	-0.0958	267	-	275	0	R.LAPLAEDVR.G
1076.4495	1075.4422	1075.5338	-0.0916	351	-	359	0	K.VNSFFSTFK.E
1083.4282	1082.4209	1082.5284	-0.1075	201	-	209	0	R.LTPYADEFK.V
1104.4679	1103.4606	1103.5611	-0.1005	135	-	143	0	R.LEPYADQLR.T
1196.4419	1195.4346	1195.5509	-0.1163	307	-	316	0	R.VEPYGENFNK.A
1215.5637	1214.5564	1214.6441	-0.0877	317	-	326	0	K.ALVQQMEQLR.Q
1231.5737	1230.5665	1230.6390	-0.0726	317	-	326	0	K.ALVQQMEQLR.Q + Oxidation (M)
1235.5752	1234.5679	1234.6669	-0.0990	113	-	123	0	R.LLPHANEVSQK.I
1258.5780	1257.5707	1257.6676	-0.0970	276	-	287	1	R.GNLKGNTEGLQK.S
1287.5711	1286.5638	1286.6579	-0.0940	144	-	154	0	R.TQVNTQAEQLR.R
1300.6026	1299.5954	1299.6782	-0.0829	190	-	200	1	K.IDQNVEELKGR.L
1310.5953	1309.5880	1309.6918	-0.1038	201	-	211	1	R.LTPYADEFKVK.I
1311.6184	1310.6111	1310.6982	-0.0871	80	-	90	0	K.LVPFATELHER.L
1319.5820	1318.5747	1318.6557	-0.0810	349	-	359	1	R.DKVNSFFSTFK.E
1350.5619	1349.5546	1349.6463	-0.0917	222	-	233	0	R.SLAPYAQDTQEK.L
1352.5712	1351.5639	1351.6520	-0.0881	306	-	316	1	R.RVEPYGENFNK.A
1407.6373	1406.6300	1406.7041	-0.0741	66	-	78	0	K.LGEVNTYAGDLQK.K
1439.7198	1438.7125	1438.7932	-0.0807	79	-	90	1	K.KLVPFATELHER.L
1443.6886	1442.6813	1442.7590	-0.0776	144	-	155	1	R.TQVNTQAEQLRR.Q
1558.7275	1557.7202	1557.7973	-0.0771	234	-	246	0	K.LNHQLEGLTFQMK.K
1574.7196	1573.7123	1573.7922	-0.0799	234	-	246	0	K.LNHQLEGLTFQMK.K + Oxidation (M)
1634.7576	1633.7503	1633.8311	-0.0808	52	-	65	0	K.SELTQQLNALFQDK.L
1805.8453	1804.8380	1804.9107	-0.0727	329	-	345	0	K.LGPHAGDVEGHLSFLEK.D
1927.9091	1926.9019	1926.9435	-0.0416	288	-	304	0	K.SLAELGGHLDQQVEEFR.R
1993.9450	1992.9377	1992.9864	-0.0487	170	-	187	0	R.ENADSLQASLRPHADELK.A
2084.0093	2083.0020	2083.0446	-0.0426	288	-	305	1	K.SLAELGGHLDQQVEEFRR.R
2193.1061	2192.0988	2192.1185	-0.0197	170	-	189	1	R.ENADSLQASLRPHADELKAK.I
2362.2237	2361.2164	2361.2400	-0.0236	167	-	187	1	R.VLRENADSLQASLRPHADELK.A
2890.4720	2889.4647	2889.4330	0.0317	222	-	246	1	R.SLAPYAQDTQEKLNHQLEGLTFQMK.K

No match to: 854.9449, 860.9786, 876.9413, 959.3966, 976.4208, 980.4021, 1065.9443, 1132.5159, 1218.5811, 1297.5441, 1336.3908, 1427.4641, 1485.7681, 1499.7430, 1508.6825, 1585.8063, 1656.7520, 1707.7763, 1834.8681, 1976.9265, 2045.0624, 2067.6716, 2069.7862, 2211.0997

6. Q13784_HUMAN **Mass:** 28141 **Score:** 280 **Expect:** 3.2e-22 **Queries matched:** 25

APOA4 protein (Fragment).- Homo sapiens (Human).

Observed	Mr(expt)	Mr(calc)	Delta	Start	End	Miss	Peptide
975.4483	974.4410	974.5032	-0.0623	122 -	130	0	R.ISASAEELR.Q
983.4562	982.4489	982.5447	-0.0958	133 -	141	0	R.LAPLAEDVR.G
1076.4495	1075.4422	1075.5338	-0.0916	217 -	225	0	K.VNSFFSTFK.E
1083.4282	1082.4209	1082.5284	-0.1075	67 -	75	0	R.LTPYADEFK.V
1104.4679	1103.4606	1103.5611	-0.1005	1 -	9	0	-.LEPYADQLR.T
1196.4419	1195.4346	1195.5509	-0.1163	173 -	182	0	R.VEPYGENFNK.A
1215.5637	1214.5564	1214.6441	-0.0877	183 -	192	0	K.ALVQQMEQLR.Q
1231.5737	1230.5665	1230.6390	-0.0726	183 -	192	0	K.ALVQQMEQLR.Q + Oxidation (M)
1258.5780	1257.5707	1257.6676	-0.0970	142 -	153	1	R.GNLKGNTEGLQK.S
1287.5711	1286.5638	1286.6579	-0.0940	10 -	20	0	R.TQVNTQAEQLR.R
1300.6026	1299.5954	1299.6782	-0.0829	56 -	66	1	K.IDQNVEELKGR.L
1310.5953	1309.5880	1309.6918	-0.1038	67 -	77	1	R.LTPYADEFKVK.I
1319.5820	1318.5747	1318.6557	-0.0810	215 -	225	1	R.DKVNSFFSTFK.E
1350.5619	1349.5546	1349.6463	-0.0917	88 -	99	0	R.SLAPYAQDTQEK.L
1352.5712	1351.5639	1351.6520	-0.0881	172 -	182	1	R.RVEPYGENFNK.A
1443.6886	1442.6813	1442.7590	-0.0776	10 -	21	1	R.TQVNTQAEQLRR.Q
1558.7275	1557.7202	1557.7973	-0.0771	100 -	112	0	K.LNHQLEGLTFQMK.K
1574.7196	1573.7123	1573.7922	-0.0799	100 -	112	0	K.LNHQLEGLTFQMK.K + Oxidation (M)
1805.8453	1804.8380	1804.9107	-0.0727	195 -	211	0	K.LGPHAGDVEGHLSFLEK.D
1927.9091	1926.9019	1926.9435	-0.0416	154 -	170	0	K.SLAELGGHLDQQVEEFR.R
1993.9450	1992.9377	1992.9864	-0.0487	36 -	53	0	R.ENADSLQASLRPHADELK.A
2084.0093	2083.0020	2083.0446	-0.0426	154 -	171	1	K.SLAELGGHLDQQVEEFRR.R
2193.1061	2192.0988	2192.1185	-0.0197	36 -	55	1	R.ENADSLQASLRPHADELKAK.I
2362.2237	2361.2164	2361.2400	-0.0236	33 -	53	1	R.VLRENADSLQASLRPHADELK.A
2890.4720	2889.4647	2889.4330	0.0317	88 -	112	1	R.SLAPYAQDTQEKLNHQLEGLTFQMK.K

No match to: 854.9449, 860.9786, 876.9413, 959.3966, 976.4208, 980.4021, 1065.9443, 1132.5159, 1218.5811, 1235.5752, 1297.5441, 1311.6184, 1336.3908, 1407.6373, 1427.4641, 1439.7198, 1485.7681, 1499.7430, 1508.6825, 1585.8063, 1634.7576, 1656.7520, 1707.7763, 1834.8681, 1976.9265, 2045.0624, 2067.6716, 2069.7862, 2211.0997

7. A47141 **Mass:** 46510 **Score:** 139 **Expect:** 4.1e-08 **Queries matched:** 18

apolipoprotein A-IV I isoform - baboon (fragment)

Observed	Mr(expt)	Mr(calc)	Delta	Start	End	Miss	Peptide
975.4483	974.4410	974.5032	-0.0623	240 -	248	0	R.ISASAEELR.Q
1076.4495	1075.4422	1075.5338	-0.0916	335 -	343	0	K.VNSFFSTFK.E
1083.4282	1082.4209	1082.5284	-0.1075	185 -	193	0	R.LTPYADEFK.V
1132.5159	1131.5087	1131.6149	-0.1062	139 -	147	1	R.RQLTPYAQR.M
1196.4419	1195.4346	1195.5509	-0.1163	291 -	300	0	R.VEPYGENFNK.A
1215.5637	1214.5564	1214.6441	-0.0877	301 -	310	0	K.ALVQQMEQLR.Q
1231.5737	1230.5665	1230.6390	-0.0726	301 -	310	0	K.ALVQQMEQLR.Q + Oxidation (M)
1235.5752	1234.5679	1234.6669	-0.0990	97 -	107	0	R.LLPHANEVSQK.I
1258.5780	1257.5707	1257.6677	-0.0970	196 -	205	1	K.IDQTVEELRR.S
1300.6026	1299.5954	1299.6782	-0.0829	174 -	184	1	K.IDQNVEELKGR.L
1310.5953	1309.5880	1309.6918	-0.1038	185 -	195	1	R.LTPYADEFKVK.I
1311.6184	1310.6111	1310.6982	-0.0871	64 -	74	0	K.LVPFATELHER.L
1319.5820	1318.5747	1318.6557	-0.0810	333 -	343	1	R.DKVNSFFSTFK.E
1407.6373	1406.6300	1406.7041	-0.0741	50 -	62	0	K.LGEVNTYAGDLQK.K
1439.7198	1438.7125	1438.7932	-0.0807	63 -	74	1	K.KLVPFATELHER.L
1634.7576	1633.7503	1633.8311	-0.0808	36 -	49	0	K.SELTQQLNALFQDK.L
1656.7520	1655.7447	1655.8817	-0.1370	218 -	231	1	K.LNHQLEGLAFQMKK.N
1805.8453	1804.8380	1804.9107	-0.0727	313 -	329	0	K.LGPHAGDVEGHLSFLEK.D

No match to: 854.9449, 860.9786, 876.9413, 959.3966, 976.4208, 980.4021, 983.4562, 1065.9443, 1104.4679, 1218.5811, 1287.5711, 1297.5441, 1336.3908, 1350.5619, 1352.5712, 1427.4641, 1443.6886, 1485.7681, 1499.7430, 1508.6825, 1558.7275, 1574.7196, 1585.8063, 1707.7763, 1834.8681, 1927.9091, 1976.9265, 1993.9450, 2045.0624, 2067.6716, 2069.7862, 2084.0093, 2193.1061, 2211.0997, 2362.2237, 2890.4720

8. S29565 **Mass:** 49846 **Score:** 119 **Expect:** 4.1e-06 **Queries matched:** 17

apolipoprotein A-IV - crab-eating macaque

Observed	Mr(expt)	Mr(calc)	Delta	Start	End	Miss	Peptide
975.4483	974.4410	974.5032	-0.0623	256 -	264	0	R.ISASAEELR.Q
1076.4495	1075.4422	1075.5338	-0.0916	351 -	359	0	K.VNSFFSTFK.E
1083.4282	1082.4209	1082.5284	-0.1075	201 -	209	0	R.LTPYADEFK.V
1132.5159	1131.5087	1131.6149	-0.1062	155 -	163	1	R.RQLTPYAQR.M
1196.4419	1195.4346	1195.5509	-0.1163	307 -	316	0	R.VEPYGENFNK.A
1215.5637	1214.5564	1214.6441	-0.0877	317 -	326	0	K.ALVQQMEQLR.Q
1231.5737	1230.5665	1230.6390	-0.0726	317 -	326	0	K.ALVQQMEQLR.Q + Oxidation (M)
1235.5752	1234.5679	1234.6669	-0.0990	113 -	123	0	R.LLPHANEVSQK.I
1258.5780	1257.5707	1257.6677	-0.0970	212 -	221	1	K.IDQTVEELRR.S

1310.5953	1309.5880	1309.6918	-0.1038	201 - 211	1	R.LTPYADEFKVK.I
1311.6184	1310.6111	1310.6982	-0.0871	80 - 90	0	K.LVPFATELHER.L
1319.5820	1318.5747	1318.6557	-0.0810	349 - 359	1	R.DKVNSFFSTFK.E
1407.6373	1406.6300	1406.7041	-0.0741	66 - 78	0	K.LGEVNTYAGDLQK.K
1439.7198	1438.7125	1438.7932	-0.0807	79 - 90	1	K.KLVPFATELHER.L
1634.7576	1633.7503	1633.8311	-0.0808	52 - 65	0	K.SELTQQLNALFQDK.L
1656.7520	1655.7447	1655.8817	-0.1370	234 - 247	1	K.LNHQLEGLAFQMKK.N
1805.8453	1804.8380	1804.9107	-0.0727	329 - 345	0	K.LGPHAGDVEGHLSFLEK.D

No match to: 854.9449, 860.9786, 876.9413, 959.3966, 976.4208, 980.4021, 983.4562, 1065.9443, 1104.4679, 1218.5811, 1287.5711, 1297.5441, 1300.6026, 1336.3908, 1350.5619, 1352.5712, 1427.4641, 1443.6886, 1485.7681, 1499.7430, 1508.6825, 1558.7275, 1574.7196, 1585.8063, 1707.7763, 1834.8681, 1927.9091, 1976.9265, 1993.9450, 2045.0624, 2067.6716, 2069.7862, 2084.0093, 2193.1061, 2211.0997, 2362.2237, 2890.4720

9. C87410 **Mass:** 70844 **Score:** 64 **Expect:** 1.3 **Queries matched:** 12

iolC protein [imported] - Caulobacter crescentus

Observed	Mr(expt)	Mr(calc)	Delta	Start - End	Miss	Peptide
976.4208	975.4135	975.5059	-0.0924	236 - 244	0	R.ASDALLVCK.R + Carbamidomethyl (C)
1132.5159	1131.5087	1131.6070	-0.0983	236 - 245	1	R.ASDALLVCKR.G + Carbamidomethyl (C)
1215.5637	1214.5564	1214.6553	-0.0990	487 - 496	1	R.QLLRLADACR.K + Carbamidomethyl (C)
1258.5780	1257.5707	1257.6400	-0.0693	63 - 73	1	R.VGADHMGRFIR.E
1439.7198	1438.7125	1438.7721	-0.0596	103 - 113	1	R.DRVNFPLIFYR.E
1499.7430	1498.7357	1498.7674	-0.0317	57 - 70	1	K.TGLLTRVGADHMGR.F + Oxidation (M)
1585.8063	1584.7990	1584.7817	0.0173	549 - 562	0	R.EIEIAIAENDPLCR.G
1707.7763	1706.7690	1706.8012	-0.0322	343 - 356	0	R.EDAELEHIHWATTR.E
1805.8453	1804.8380	1804.8703	-0.0323	184 - 199	1	K.DAGENRFVENQQVTAK.L
1976.9265	1975.9192	1975.9864	-0.0672	341 - 356	1	R.LREDAELEHIHWATTR.E
2084.0093	2083.0020	2083.0181	-0.0161	74 - 92	1	R.EQLEREGVDVAGVLSDPDR.L
2362.2237	2361.2164	2361.2592	-0.0429	162 - 183	1	K.AAGGRVAFDIDYRPVLWGLTGK.D

No match to: 854.9449, 860.9786, 876.9413, 959.3966, 975.4483, 980.4021, 983.4562, 1065.9443, 1076.4495, 1083.4282, 1104.4679, 1196.4419, 1218.5811, 1231.5737, 1235.5752, 1287.5711, 1297.5441, 1300.6026, 1310.5953, 1311.6184, 1319.5820, 1336.3908, 1350.5619, 1352.5712, 1407.6373, 1427.4641, 1443.6886, 1485.7681, 1508.6825, 1558.7275, 1574.7196, 1634.7576, 1656.7520, 1834.8681, 1927.9091, 1993.9450, 2045.0624, 2067.6716, 2069.7862, 2193.1061, 2211.0997, 2890.4720

10. Q3SQM9_NITWN **Mass:** 41297 **Score:** 62 **Expect:** 1.8 **Queries matched:** 10

Acyl-CoA dehydrogenase (EC 1.3.99.2).- Nitrobacter winogradskyi (strain Nb-255 / ATCC 25391).

Observed	Mr(expt)	Mr(calc)	Delta	Start - End	Miss	Peptide
975.4483	974.4410	974.4781	-0.0371	57 - 66	0	R.NDVGGSALSR.L
976.4208	975.4135	975.4807	-0.0672	232 - 241	0	R.IAMAGLDGGR.L + Oxidation (M)
983.4562	982.4489	982.4906	-0.0417	259 - 266	1	K.ALGYMKER.K + Oxidation (M)
1076.4495	1075.4422	1075.4869	-0.0447	200 - 208	0	K.MGWNAQPTR.A + Oxidation (M)
1300.6026	1299.5954	1299.6968	-0.1015	44 - 56	0	R.EAAALGIGGICIR.N + Carbamidomethyl (C)
1319.5820	1318.5747	1318.6629	-0.0881	187 - 199	1	R.GTPGVSFGANERK.M
1439.7198	1438.7125	1438.6432	0.0693	307 - 319	0	R.EDTNATMLCAVAK.R + Carbamidomethyl (C); Oxidation (M)
1574.7196	1573.7123	1573.8140	-0.1017	23 - 35	1	R.KIAPFALQWDEEK.H
1834.8681	1833.8608	1833.9155	-0.0547	223 - 241	1	R.IGAEGAGFRIAMAGLDGGR.L + Oxidation (M)
1976.9265	1975.9192	1975.9686	-0.0494	200 - 216	1	K.MGWNAQPTRAVIFENAR.V + Oxidation (M)

No match to: 854.9449, 860.9786, 876.9413, 959.3966, 980.4021, 1065.9443, 1083.4282, 1104.4679, 1132.5159, 1196.4419, 1215.5637, 1218.5811, 1231.5737, 1235.5752, 1258.5780, 1287.5711, 1297.5441, 1310.5953, 1311.6184, 1336.3908, 1350.5619, 1352.5712, 1407.6373, 1427.4641, 1443.6886, 1485.7681, 1499.7430, 1508.6825, 1558.7275, 1585.8063, 1634.7576, 1656.7520, 1707.7763, 1805.8453, 1927.9091, 1993.9450, 2045.0624, 2067.6716, 2069.7862, 2084.0093, 2193.1061, 2211.0997, 2362.2237, 2890.4720

11. I54248 **Mass:** 10612 **Score:** 62 **Expect:** 2.2 **Queries matched:** 7

apolipoprotein A-IV - chimpanzee (fragment)

Observed	Mr(expt)	Mr(calc)	Delta	Start - End	Miss	Peptide
1076.4495	1075.4422	1075.5338	-0.0916	46 - 54	0	K.VNSFFSTFK.E
1196.4419	1195.4346	1195.5509	-0.1163	2 - 11	0	R.VEPYGENFNK.A
1215.5637	1214.5564	1214.6441	-0.0877	12 - 21	0	K.ALVQQMEQLR.T
1231.5737	1230.5665	1230.6390	-0.0726	12 - 21	0	K.ALVQQMEQLR.T + Oxidation (M)
1319.5820	1318.5747	1318.6557	-0.0810	44 - 54	1	R.DKVNSFFSTFK.E
1352.5712	1351.5639	1351.6520	-0.0881	1 - 11	1	-.RVEPYGENFNK.A
1805.8453	1804.8380	1804.9107	-0.0727	24 - 40	0	K.LGPHAGDVEGHLSFLEK.D

No match to: 854.9449, 860.9786, 876.9413, 959.3966, 975.4483, 976.4208, 980.4021, 983.4562, 1065.9443, 1083.4282, 1104.4679, 1132.5159, 1218.5811, 1235.5752, 1258.5780, 1287.5711, 1297.5441, 1300.6026, 1310.5953, 1311.6184, 1336.3908, 1350.5619, 1407.6373, 1427.4641, 1439.7198, 1443.6886, 1485.7681, 1499.7430, 1508.6825, 1558.7275, 1574.7196, 1585.8063, 1634.7576, 1656.7520, 1707.7763, 1834.8681, 1927.9091, 1976.9265, 1993.9450, 2045.0624, 2067.6716, 2069.7862, 2084.0093, 2193.1061, 2211.0997, 2362.2237, 2890.4720

12. Q6WAZ7_TRYCR **Mass:** 82415 **Score:** 56 **Expect:** 7.6 **Queries matched:** 12

C71 surface protein.- Trypanosoma cruzi.

Observed	Mr(expt)	Mr(calc)	Delta	Start - End	Miss	Peptide
975.4483	974.4410	974.5145	-0.0735	674 - 682	1	K.NVDSKGISR.F
1132.5159	1131.5087	1131.5996	-0.0909	87 - 96	1	K.NGRETLSSLR.V
1215.5637	1214.5564	1214.5567	-0.0003	683 - 695	0	R.FYIGGDGGSAGSK.E
1235.5752	1234.5679	1234.5975	-0.0296	450 - 462	0	K.GGGESSLGMVSVR.L
1287.5711	1286.5638	1286.6255	-0.0616	633 - 642	1	K.ELRSNWEPEK.T
1300.6026	1299.5954	1299.5080	0.0874	322 - 332	0	K.LMMMTACDDGR.R + Carbamidomethyl (C)

1508.6825	1507.6753	1507.7266	-0.0513	217 -	232	0	R.LFGAVGNVSNDGSSGK.K
1585.8063	1584.7990	1584.7532	0.0459	539 -	554	0	K.GTNGVATGYADGVTFR.G
1634.7576	1633.7503	1633.8861	-0.1358	380 -	393	1	K.DVMLVTLPVYSNRK.G
1656.7520	1655.7447	1655.7824	-0.0377	592 -	607	0	K.GDNPIPLVGATMNDDK.N
1805.8453	1804.8380	1804.8447	-0.0067	148 -	164	0	K.TQVLVECPAGNNSCVSK.K + Carbamidomethyl (C)
2362.2237	2361.2164	2361.1052	0.1112	484 -	507	1	R.VSELCPSPGTAKNPSAGSGCSTVR.I + Carbamidomethyl (C)

No match to: 854.9449, 860.9786, 876.9413, 959.3966, 976.4208, 980.4021, 983.4562, 1065.9443, 1076.4495, 1083.4282, 1104.4679, 1196.4419, 1218.5811, 1231.5737, 1258.5780, 1297.5441, 1310.5953, 1311.6184, 1319.5820, 1336.3908, 1350.5619, 1352.5712, 1407.6373, 1427.4641, 1439.7198, 1443.6886, 1485.7681, 1499.7430, 1558.7275, 1574.7196, 1707.7763, 1834.8681, 1927.9091, 1976.9265, 1993.9450, 2045.0624, 2067.6716, 2069.7862, 2084.0093, 2193.1061, 2211.0997, 2890.4720

13. Q3XUS9_9PROT **Mass:** 61027 **Score:** 56 **Expect:** 8.1 **Queries matched:** 11

Coenzyme B12-binding:Radical SAM.- Magnetococcus sp. MC-1.

Observed	Mr(expt)	Mr(calc)	Delta	Start	End	Miss	Peptide
1076.4495	1075.4422	1075.5406	-0.0984	1 -	9	0	-.MDMLLVNPK.H + Oxidation (M)
1218.5811	1217.5738	1217.6139	-0.0401	294 -	305	0	R.IGAESGSDEILK.D
1235.5752	1234.5679	1234.6094	-0.0415	236 -	245	1	R.DELFFHSKGR.V
1258.5780	1257.5707	1257.5416	0.0290	284 -	293	1	R.MEQCGCRFLR.I + Oxidation (M)
1287.5711	1286.5638	1286.6830	-0.1192	212 -	222	1	R.SAKDILDEIQR.I
1407.6373	1406.6300	1406.7153	-0.0853	178 -	190	0	K.ATYGQEVQVLSGR.G
1508.6825	1507.6753	1507.7531	-0.0779	426 -	437	1	R.DQDRFLVQNAFR.L
1574.7196	1573.7123	1573.8199	-0.1076	294 -	308	1	R.IGAESGSDEILKDIK.K
1585.8063	1584.7990	1584.8088	-0.0098	232 -	243	1	R.IFFRDELFFHSK.G
1634.7576	1633.7503	1633.8675	-0.1172	291 -	305	1	R.FLRIGAESGSDEILK.D
1927.9091	1926.9019	1926.8426	0.0593	191 -	207	1	R.GCSFKCTFCYNVVSGQK.W + Carbamidomethyl (C)

No match to: 854.9449, 860.9786, 876.9413, 959.3966, 975.4483, 976.4208, 980.4021, 983.4562, 1065.9443, 1083.4282, 1104.4679, 1132.5159, 1196.4419, 1215.5637, 1231.5737, 1297.5441, 1300.6026, 1310.5953, 1311.6184, 1319.5820, 1336.3908, 1350.5619, 1352.5712, 1427.4641, 1439.7198, 1443.6886, 1485.7681, 1499.7430, 1558.7275, 1656.7520, 1707.7763, 1805.8453, 1834.8681, 1976.9265, 1993.9450, 2045.0624, 2067.6716, 2069.7862, 2084.0093, 2193.1061, 2211.0997, 2362.2237, 2890.4720

14. Q4USB5_XANC8 **Mass:** 13788 **Score:** 56 **Expect:** 8.3 **Queries matched:** 7

HrpB2 protein.- Xanthomonas campestris pv. campestris (strain 8004).

Observed	Mr(expt)	Mr(calc)	Delta	Start	End	Miss	Peptide
975.4483	974.4410	974.4855	-0.0445	52 -	60	0	R.VGEPSMISR.M
1196.4419	1195.4346	1195.4961	-0.0615	61 -	70	0	R.MVDMQNDGVR.A + 2 Oxidation (M)
1350.5619	1349.5546	1349.6206	-0.0660	86 -	97	0	K.MGLEEIAAEEMK.L
1558.7275	1557.7202	1557.7245	-0.0043	71 -	85	0	R.AMTEHVDAFGANAPK.M
1574.7196	1573.7123	1573.7194	-0.0072	71 -	85	0	R.AMTEHVDAFGANAPK.M + Oxidation (M)
1993.9450	1992.9377	1992.9220	0.0158	35 -	51	0	R.FQALMQSSCPLPPAMQR.V + Carbamidomethyl (C); 2 Oxidation (M)
2890.4720	2889.4647	2889.3346	0.1301	71 -	97	1	R.AMTEHVDAFGANAPKMGLEEIAAEEMK.L

No match to: 854.9449, 860.9786, 876.9413, 959.3966, 976.4208, 980.4021, 983.4562, 1065.9443, 1076.4495, 1083.4282, 1104.4679, 1132.5159, 1215.5637, 1218.5811, 1231.5737, 1235.5752, 1258.5780, 1287.5711, 1297.5441, 1300.6026, 1310.5953, 1311.6184, 1319.5820, 1336.3908, 1352.5712, 1407.6373, 1427.4641, 1439.7198, 1443.6886, 1485.7681, 1499.7430, 1508.6825, 1585.8063, 1634.7576, 1656.7520, 1707.7763, 1805.8453, 1834.8681, 1927.9091, 1976.9265, 2045.0624, 2067.6716, 2069.7862, 2084.0093, 2193.1061, 2211.0997, 2362.2237

15. Q4D9P5_TRYCR **Mass:** 126270 **Score:** 54 **Expect:** 13 **Queries matched:** 15

DNA-directed RNA polymerase III subunit, putative (EC 2.7.7.6).- Trypanosoma cruzi.

Observed	Mr(expt)	Mr(calc)	Delta	Start	End	Miss	Peptide
975.4483	974.4410	974.5032	-0.0623	810 -	819	0	K.ALNSDGIASK.G
983.4562	982.4489	982.4542	-0.0053	691 -	699	0	R.NTFQSAMGK.Q
1083.4282	1082.4209	1082.4927	-0.0718	768 -	776	1	R.CSLDRGYGR.C + Carbamidomethyl (C)
1218.5811	1217.5738	1217.6040	-0.0302	886 -	896	1	R.VPEPGDKFSSR.H
1310.5953	1309.5880	1309.6700	-0.0820	407 -	417	1	K.AFNVKQLMESK.L + Oxidation (M)
1352.5712	1351.5639	1351.6442	-0.0803	730 -	740	1	R.TKAMDLTHYEK.L + Oxidation (M)
1439.7198	1438.7125	1438.7213	-0.0088	336 -	346	1	R.HKALYVCFMVR.R + Carbamidomethyl (C); Oxidation (M)
1508.6825	1507.6753	1507.7381	-0.0628	157 -	169	0	R.ECPLDPGGYFIIK.G + Carbamidomethyl (C)
1558.7275	1557.7202	1557.8548	-0.1346	1108 -	1121	0	K.LLLQELQGMGISTR.L
1574.7196	1573.7123	1573.8133	-0.1011	170 -	183	1	K.GVEKVCLVQEQQSK.N
1805.8453	1804.8380	1804.9028	-0.0648	901 -	917	0	K.GVVGLITDGVNMPFNEK.G + Oxidation (M)
1927.9091	1926.9019	1927.0012	-0.0993	369 -	385	0	R.FETTGTLMGLLFEDLLK.Q
1993.9450	1992.9377	1993.0488	-0.1111	130 -	147	1	K.IGTIPIMLKSSSCNLFGR.T + Carbamidomethyl (C)
2045.0624	2044.0551	2044.1060	-0.0509	121 -	138	1	K.RPCTETDIKIGTIPIMLK.S + Oxidation (M)
2084.0093	2083.0020	2083.1023	-0.1003	368 -	385	1	K.RFETTGTLMGLLFEDLLK.Q

No match to: 854.9449, 860.9786, 876.9413, 959.3966, 976.4208, 980.4021, 1065.9443, 1076.4495, 1104.4679, 1132.5159, 1196.4419, 1215.5637, 1231.5737, 1235.5752, 1258.5780, 1287.5711, 1297.5441, 1300.6026, 1311.6184, 1319.5820, 1336.3908, 1350.5619, 1407.6373, 1427.4641, 1443.6886, 1485.7681, 1499.7430, 1585.8063, 1634.7576, 1656.7520, 1707.7763, 1834.8681, 1976.9265, 2067.6716, 2069.7862, 2193.1061, 2211.0997, 2362.2237, 2890.4720

16. C83690 **Mass:** 22489 **Score:** 53 **Expect:** 16 **Queries matched:** 7

hypothetical protein BH0323 [imported] - Bacillus halodurans (strain C-125)

Observed	Mr(expt)	Mr(calc)	Delta	Start	End	Miss	Peptide
1235.5752	1234.5679	1234.6346	-0.0667	90 -	99	0	R.LNPFDFNQLK.T
1300.6026	1299.5954	1299.6935	-0.0981	127 -	137	1	K.DGKHVTEFIVR.E
1310.5953	1309.5880	1309.6374	-0.0494	80 -	89	1	K.EHREEIIDNR.L
1834.8681	1833.8608	1833.9049	-0.0441	46 -	59	1	K.LKFHDHLDENYIYK.C

```
1927.9091  1926.9019  1926.9184  -0.0165   102 - 117   0  K.EDHILTHINIDEHEGR.R
2084.0093  2083.0020  2083.0195  -0.0175   102 - 118   1  K.EDHILTHINIDEHEGRR.V
2211.0997  2210.0924  2210.1081  -0.0157    60 -  78   1  K.CFVIDDPVILNKWYSAAEK.K
```

No match to: 854.9449, 860.9786, 876.9413, 959.3966, 975.4483, 976.4208, 980.4021, 983.4562, 1065.9443, 1076.4495, 1083.4282, 1104.4679, 1132.5159, 1196.4419, 1215.5637, 1218.5811, 1231.5737, 1258.5780, 1287.5711, 1297.5441, 1311.6184, 1319.5820, 1336.3908, 1350.5619, 1352.5712, 1407.6373, 1427.4641, 1439.7198, 1443.6886, 1485.7681, 1499.7430, 1508.6825, 1558.7275, 1574.7196, 1585.8063, 1634.7576, 1656.7520, 1707.7763, 1805.8453, 1976.9265, 1993.9450, 2045.0624, 2067.6716, 2069.7862, 2193.1061, 2362.2237, 2890.4720

17. Q56EH5_9CAUD **Mass:** 36302 **Score:** 52 **Expect:** 20 **Queries matched:** 8

Hypothetical protein PHG31ORF188c.- Aeromonas phage 31.

Observed	Mr(expt)	Mr(calc)	Delta	Start	End	Miss	Peptide
1287.5711	1286.5638	1286.6111	-0.0473	247 -	257	0	K.INMHIDCSGIK.H + Carbamidomethyl (C)
1311.6184	1310.6111	1310.6176	-0.0065	10 -	20	0	K.VEISDCVYQQK.K
1407.6373	1406.6300	1406.7306	-0.1006	236 -	246	1	R.YNLDFPNRQIK.I
1439.7198	1438.7125	1438.7126	-0.0001	10 -	21	1	K.VEISDCVYQQKK.S
1656.7520	1655.7447	1655.8487	-0.1040	244 -	257	1	R.QIKINMHIDCSGIK.H + Carbamidomethyl (C)
1707.7763	1706.7690	1706.8185	-0.0495	264 -	277	0	K.ITGYPEECEIILDR.N + Carbamidomethyl (C)
2193.1061	2192.0988	2191.9336	0.1652	161 -	177	1	K.QCVEVMDRYMDDSIEFR.V + Carbamidomethyl (C)
2362.2237	2361.2164	2361.0728	0.1435	147 -	168	1	R.SGTFDSGSAGIFAKQCVEVMDR.Y + Carbamidomethyl (C)

No match to: 854.9449, 860.9786, 876.9413, 959.3966, 975.4483, 976.4208, 980.4021, 983.4562, 1065.9443, 1076.4495, 1083.4282, 1104.4679, 1132.5159, 1196.4419, 1215.5637, 1218.5811, 1231.5737, 1235.5752, 1258.5780, 1297.5441, 1300.6026, 1310.5953, 1319.5820, 1336.3908, 1350.5619, 1352.5712, 1427.4641, 1443.6886, 1485.7681, 1499.7430, 1508.6825, 1558.7275, 1574.7196, 1585.8063, 1634.7576, 1805.8453, 1834.8681, 1927.9091, 1976.9265, 1993.9450, 2045.0624, 2067.6716, 2069.7862, 2084.0093, 2211.0997, 2890.4720

18. Q4MZD6_THEPA **Mass:** 62104 **Score:** 52 **Expect:** 20 **Queries matched:** 13

Hypothetical protein.- Theileria parva.

Observed	Mr(expt)	Mr(calc)	Delta	Start	End	Miss	Peptide
1132.5159	1131.5087	1131.6175	-0.1089	415 -	424	0	K.ALELDPTFVK.A
1231.5737	1230.5665	1230.5591	0.0074	311 -	320	0	R.YDDAILCYQK.S
1258.5780	1257.5707	1257.5845	-0.0139	301 -	310	1	R.LAACYTKMER.Y + Carbamidomethyl (C); Oxidation (M)
1287.5711	1286.5638	1286.4875	0.0763	178 -	188	0	R.DGLMACMGMNR.N + Carbamidomethyl (C); 2 Oxidation (M)
1350.5619	1349.5546	1349.6649	-0.1103	76 -	87	1	K.GLCEYKLGNPEK.A
1407.6373	1406.6300	1406.6863	-0.0563	76 -	87	1	K.GLCEYKLGNPEK.A + Carbamidomethyl (C)
1485.7681	1484.7608	1484.6783	0.0825	139 -	150	1	R.KYQEQDPEYASK.L
1558.7275	1557.7202	1557.7054	0.0148	508 -	520	0	K.ISENPMTMAEYLK.D + 2 Oxidation (M)
1656.7520	1655.7447	1655.8049	-0.0602	449 -	463	1	K.GLKVDPNNNECLQGR.N
1805.8453	1804.8380	1804.8375	0.0005	261 -	275	1	K.AAVYLEMGDYEKCIK.T + Carbamidomethyl (C); Oxidation (M)
1993.9450	1992.9377	1993.0012	-0.0635	396 -	414	1	R.AAALLKLCEYPSALADCNK.A
2045.0624	2044.0551	2043.9214	0.1337	452 -	469	1	K.VDPNNNECLQGRNNCLNK.I
2069.7862	2068.7789	2068.8869	-0.1080	42 -	60	0	R.SGAYASMYMYNEALADANK.C

No match to: 854.9449, 860.9786, 876.9413, 959.3966, 975.4483, 976.4208, 980.4021, 983.4562, 1065.9443, 1076.4495, 1083.4282, 1104.4679, 1196.4419, 1215.5637, 1218.5811, 1235.5752, 1297.5441, 1300.6026, 1310.5953, 1311.6184, 1319.5820, 1336.3908, 1352.5712, 1427.4641, 1439.7198, 1443.6886, 1499.7430, 1508.6825, 1574.7196, 1585.8063, 1634.7576, 1707.7763, 1834.8681, 1927.9091, 1976.9265, 2067.6716, 2084.0093, 2193.1061, 2211.0997, 2362.2237, 2890.4720

19. Q4JCL5_SULAC **Mass:** 24648 **Score:** 52 **Expect:** 22 **Queries matched:** 8

Conserved protein.- Sulfolobus acidocaldarius.

Observed	Mr(expt)	Mr(calc)	Delta	Start	End	Miss	Peptide
983.4562	982.4489	982.4793	-0.0304	210 -	219	0	R.VSIACSGFLS.-
1132.5159	1131.5087	1131.5706	-0.0619	49 -	58	1	K.AMKNLENANK.R
1215.5637	1214.5564	1214.6295	-0.0731	81 -	91	0	R.GDLFIHTIDGK.I
1310.5953	1309.5880	1309.6877	-0.0997	144 -	155	0	K.YSSLIVLSESGR.K
1311.6184	1310.6111	1310.6765	-0.0653	199 -	209	0	K.NVLETHQVICR.V
1319.5820	1318.5747	1318.6564	-0.0817	98 -	107	1	R.QMRPPKNYNR.F + Oxidation (M)
1499.7430	1498.7357	1498.8442	-0.1085	92 -	103	1	K.IIFVDRQMRPPK.N
1927.9091	1926.9019	1927.0852	-0.1833	108 -	124	1	R.FVGLMEQLLLEGKIPPK.G + Oxidation (M)

No match to: 854.9449, 860.9786, 876.9413, 959.3966, 975.4483, 976.4208, 980.4021, 1065.9443, 1076.4495, 1083.4282, 1104.4679, 1196.4419, 1218.5811, 1231.5737, 1235.5752, 1258.5780, 1287.5711, 1297.5441, 1300.6026, 1336.3908, 1350.5619, 1352.5712, 1407.6373, 1427.4641, 1439.7198, 1443.6886, 1485.7681, 1508.6825, 1558.7275, 1574.7196, 1585.8063, 1634.7576, 1656.7520, 1707.7763, 1805.8453, 1834.8681, 1976.9265, 1993.9450, 2045.0624, 2067.6716, 2069.7862, 2084.0093, 2193.1061, 2211.0997, 2362.2237, 2890.4720

20. Q8SPT5_MACMU **Mass:** 16793 **Score:** 51 **Expect:** 24 **Queries matched:** 7

Growth differentiation factor 9 (Fragment).- Macaca mulatta (Rhesus macaque).

Observed	Mr(expt)	Mr(calc)	Delta	Start	End	Miss	Peptide
975.4483	974.4410	974.5297	-0.0888	34 -	41	1	K.FSSKTLHR.A
980.4021	979.3948	979.4653	-0.0705	23 -	30	0	K.CVCNLMIK.E + Carbamidomethyl (C)
1350.5619	1349.5546	1349.6505	-0.0959	23 -	33	1	K.CVCNLMIKEPK.F + Carbamidomethyl (C); Oxidation (M)
1407.6373	1406.6300	1406.6720	-0.0420	23 -	33	1	K.CVCNLMIKEPK.F + 2 Carbamidomethyl (C); Oxidation (M)
1443.6886	1442.6813	1442.6356	0.0457	79 -	90	0	R.SIHMSINFTCMK.D + 2 Oxidation (M)
1656.7520	1655.7447	1655.7582	-0.0135	78 -	90	1	K.RSIHMSINFTCMK.D + Carbamidomethyl (C); 2 Oxidation (M)
2890.4720	2889.4647	2889.4801	-0.0154	5 -	30	1	K.SVLLYTINNSVSFSSAVKCVCNLMIK.E + Carbamidomethyl (C)

No match to: 854.9449, 860.9786, 876.9413, 959.3966, 976.4208, 983.4562, 1065.9443, 1076.4495, 1083.4282, 1104.4679, 1132.5159, 1196.4419, 1215.5637, 1218.5811, 1231.5737, 1235.5752, 1258.5780, 1287.5711, 1297.5441, 1300.6026, 1310.5953, 1311.6184, 1319.5820, 1336.3908, 1352.5712, 1427.4641, 1439.7198, 1485.7681, 1499.7430, 1508.6825, 1558.7275, 1574.7196, 1585.8063, 1634.7576, 1707.7763, 1805.8453, 1834.8681, 1927.9091, 1976.9265, 1993.9450, 2045.0624, 2067.6716, 2069.7862, 2084.0093, 2193.1061, 2211.0997, 2362.2237

21. A44277 **Mass:** 64504 **Score:** 51 **Expect:** 25 **Queries matched:** 13

nonstructural protein NS1 - bluetongue virus (serotype 2)

Observed	Mr(expt)	Mr(calc)	Delta	Start	End	Miss	Peptide
975.4483	974.4410	974.4781	-0.0371	124 -	132	0	K.QSALANSER.V
983.4562	982.4489	982.4099	0.0390	115 -	123	0	K.SGMMDEAVK.Q + Oxidation (M)
1218.5811	1217.5738	1217.5573	0.0165	503 -	512	0	R.AYATMFEMVR.C
1258.5780	1257.5707	1257.5304	0.0403	36 -	45	0	R.NCLFNGMCVK.Q + 2 Carbamidomethyl (C); Oxidation (M)
1287.5711	1286.5638	1286.6653	-0.1014	347 -	357	0	K.QIPTCSVIDVR.A + Carbamidomethyl (C)
1300.6026	1299.5954	1299.5886	0.0068	35 -	45	1	K.RNCLFNGMCVK.Q + Oxidation (M)
1350.5619	1349.5546	1349.6584	-0.1038	334 -	343	1	R.KHTCQLCYLK.H + 2 Carbamidomethyl (C)
1352.5712	1351.5639	1351.6554	-0.0915	104 -	114	1	R.CGAQLLEDYRK.S + Carbamidomethyl (C)
1574.7196	1573.7123	1573.7493	-0.0371	335 -	346	1	K.HTCQLCYLKHSK.Q + 2 Carbamidomethyl (C)
1585.8063	1584.7990	1584.7467	0.0524	201 -	213	1	R.QINTCPYTGYRGR.V + Carbamidomethyl (C)
1834.8681	1833.8608	1833.8575	0.0032	267 -	281	0	R.VPTGEFPLHQMMLMR.R + 3 Oxidation (M)
1927.9091	1926.9019	1926.9951	-0.0932	251 -	266	1	R.VGYAEEIRYVQQLFGR.V
1993.9450	1992.9377	1993.1222	-0.1845	214 -	229	0	R.VFQVMFLPIQLINFLR.M + Oxidation (M)

No match to: 854.9449, 860.9786, 876.9413, 959.3966, 976.4208, 980.4021, 1065.9443, 1076.4495, 1083.4282, 1104.4679, 1132.5159, 1196.4419, 1215.5637, 1231.5737, 1235.5752, 1297.5441, 1310.5953, 1311.6184, 1319.5820, 1336.3908, 1407.6373, 1427.4641, 1439.7198, 1443.6886, 1485.7681, 1499.7430, 1508.6825, 1558.7275, 1634.7576, 1656.7520, 1707.7763, 1805.8453, 1976.9265, 2045.0624, 2067.6716, 2069.7862, 2084.0093, 2193.1061, 2211.0997, 2362.2237, 2890.4720

22. T50803 **Mass:** 83475 **Score:** 51 **Expect:** 27 **Queries matched:** 11

hypothetical protein T30N20_210 - Arabidopsis thaliana

Observed	Mr(expt)	Mr(calc)	Delta	Start	End	Miss	Peptide
975.4483	974.4410	974.4062	0.0348	402 -	408	0	R.HECLCTR.A + 2 Carbamidomethyl (C)
1104.4679	1103.4606	1103.5546	-0.0940	30 -	38	0	R.MQFHSSLVR.R
1297.5441	1296.5368	1296.5694	-0.0326	19 -	29	0	R.SQDPSHEVDQR.M
1350.5619	1349.5546	1349.6880	-0.1334	642 -	651	1	R.WFIWEKQTGR.L
1585.8063	1584.7990	1584.8042	-0.0051	723 -	735	1	R.NRENPLSVELMQR.F
1634.7576	1633.7503	1633.8246	-0.0743	725 -	737	1	R.ENPLSVELMQRFR.M + Oxidation (M)
1927.9091	1926.9019	1926.9622	-0.0603	84 -	100	0	K.LLHSIDTGHTANIFCTK.F + Carbamidomethyl (C)
2193.1061	2192.0988	2192.0796	0.0192	610 -	629	1	R.YVGHCNVGTDIKQASFLGQR.G
2211.0997	2210.0924	2210.0967	-0.0043	153 -	173	0	K.LAVEPGNPNVVWSASEDGTLR.Q
2362.2237	2361.2164	2360.9942	0.2222	736 -	754	1	R.FRMQEFAEGNFHPFECTQS.- + Carbamidomethyl (C)
2890.4720	2889.4647	2889.4555	0.0092	220 -	245	1	K.SCDISATRPHLLLVGGSDAFARLYDR.R + Carbamidomethyl (C)

No match to: 854.9449, 860.9786, 876.9413, 959.3966, 976.4208, 980.4021, 983.4562, 1065.9443, 1076.4495, 1083.4282, 1132.5159, 1196.4419, 1215.5637, 1218.5811, 1231.5737, 1235.5752, 1258.5780, 1287.5711, 1300.6026, 1310.5953, 1311.6184, 1319.5820, 1336.3908, 1352.5712, 1407.6373, 1427.4641, 1439.7198, 1443.6886, 1485.7681, 1499.7430, 1508.6825, 1558.7275, 1574.7196, 1656.7520, 1707.7763, 1805.8453, 1834.8681, 1976.9265, 1993.9450, 2045.0624, 2067.6716, 2069.7862, 2084.0093

23. Q94BQ3_ARATH **Mass:** 83778 **Score:** 51 **Expect:** 28 **Queries matched:** 11

Hypothetical protein At5g10940.- Arabidopsis thaliana (Mouse-ear cress).

Observed	Mr(expt)	Mr(calc)	Delta	Start	End	Miss	Peptide
975.4483	974.4410	974.4062	0.0348	405 -	411	0	R.HECLCTR.A + 2 Carbamidomethyl (C)
1104.4679	1103.4606	1103.5546	-0.0940	30 -	38	0	R.MQFHSSLVR.R
1297.5441	1296.5368	1296.5694	-0.0326	19 -	29	0	R.SQDPSHEVDQR.M
1350.5619	1349.5546	1349.6880	-0.1334	645 -	654	1	R.WFIWEKQTGR.L
1585.8063	1584.7990	1584.8042	-0.0051	726 -	738	1	R.NRENPLSVELMQR.F
1634.7576	1633.7503	1633.8246	-0.0743	728 -	740	1	R.ENPLSVELMQRFR.M + Oxidation (M)
1927.9091	1926.9019	1926.9622	-0.0603	84 -	100	0	K.LLHSIDTGHTANIFCTK.F + Carbamidomethyl (C)
2193.1061	2192.0988	2192.0796	0.0192	613 -	632	1	R.YVGHCNVGTDIKQASFLGQR.G
2211.0997	2210.0924	2210.0967	-0.0043	153 -	173	0	K.LAVEPGNPNVVWSASEDGTLR.Q
2362.2237	2361.2164	2360.9942	0.2222	739 -	757	1	R.FRMQEFAEGNFHPFECTQS.- + Carbamidomethyl (C)
2890.4720	2889.4647	2889.4555	0.0092	220 -	245	1	K.SCDISATRPHLLLVGGSDAFARLYDR.R + Carbamidomethyl (C)

No match to: 854.9449, 860.9786, 876.9413, 959.3966, 976.4208, 980.4021, 983.4562, 1065.9443, 1076.4495, 1083.4282, 1132.5159, 1196.4419, 1215.5637, 1218.5811, 1231.5737, 1235.5752, 1258.5780, 1287.5711, 1300.6026, 1310.5953, 1311.6184, 1319.5820, 1336.3908, 1352.5712, 1407.6373, 1427.4641, 1439.7198, 1443.6886, 1485.7681, 1499.7430, 1508.6825, 1558.7275, 1574.7196, 1656.7520, 1707.7763, 1805.8453, 1834.8681, 1976.9265, 1993.9450, 2045.0624, 2067.6716, 2069.7862, 2084.0093

24. Q6Q539_YEAST **Mass:** 24211 **Score:** 50 **Expect:** 30 **Queries matched:** 7

YDR226W.- Saccharomyces cerevisiae (Baker's yeast).

Observed	Mr(expt)	Mr(calc)	Delta	Start	End	Miss	Peptide
1076.4495	1075.4422	1075.5145	-0.0723	168 -	177	1	R.SDDNADALKK.R
1104.4679	1103.4606	1103.4917	-0.0311	76 -	85	0	K.DELTNNPACK.N
1218.5811	1217.5738	1217.6114	-0.0376	147 -	156	1	K.IFNPPKEDMK.D
1439.7198	1438.7125	1438.7139	-0.0014	30 -	42	0	R.FHAAHLATGDMLR.S
1443.6886	1442.6813	1442.8092	-0.1279	43 -	56	1	R.SQIAKGTQLGLEAK.K
1993.9450	1992.9377	1993.0309	-0.0931	179 -	195	1	R.LAAYHAQTEPIVDFYKK.T
2193.1061	2192.0988	2192.0571	0.0417	76 -	95	1	K.DELTNNPACKNGFILDGFPK.T

No match to: 854.9449, 860.9786, 876.9413, 959.3966, 975.4483, 976.4208, 980.4021, 983.4562, 1065.9443, 1083.4282, 1132.5159, 1196.4419, 1215.5637, 1231.5737, 1235.5752, 1258.5780, 1287.5711, 1297.5441, 1300.6026, 1310.5953, 1311.6184, 1319.5820, 1336.3908, 1350.5619, 1352.5712, 1407.6373, 1427.4641, 1485.7681, 1499.7430, 1508.6825, 1558.7275, 1574.7196, 1585.8063, 1634.7576, 1656.7520, 1707.7763, 1805.8453, 1834.8681, 1927.9091, 1976.9265, 2045.0624, 2067.6716, 2069.7862, 2084.0093, 2211.0997, 2362.2237, 2890.4720

25. Q2BL66_9GAMM **Mass:** 52033 **Score:** 50 **Expect:** 31 **Queries matched:** 9

Argininosuccinate lyase.- Oceanospirillum sp. MED92.

Observed	Mr(expt)	Mr(calc)	Delta	Start	End	Miss	Peptide
1218.5811	1217.5738	1217.5822	-0.0084	357 -	366	1	K.EENMREAALR.G
1231.5737	1230.5665	1230.5087	0.0577	183 -	191	1	R.DFSRFEDCR.E + Carbamidomethyl (C)
1407.6373	1406.6300	1406.6361	-0.0060	1 -	12	1	-.MSDKTNQQWGGR.F
1656.7520	1655.7447	1655.8518	-0.1071	367 -	381	1	R.GFSTATDLADYLVRK.G
1707.7763	1706.7690	1706.8483	-0.0793	341 -	356	1	K.GCLRAFADMVPALEAK.E + Oxidation (M)
1805.8453	1804.8380	1804.9691	-0.1311	300 -	315	1	K.SGRVFGHLMSLLTLMK.S + Oxidation (M)
1976.9265	1975.9192	1975.9374	-0.0182	324 -	340	1	K.DNQEDKEPLFDAIDTVK.G
2045.0624	2044.0551	2044.0775	-0.0224	194 -	213	0	R.ANIMPLGAAALAGTTYPIQR.D + Oxidation (M)
2211.0997	2210.0924	2210.0863	0.0060	237 -	255	0	R.DFAIEFCSASSILLMHLTR.M + Carbamidomethyl (C)

No match to: 854.9449, 860.9786, 876.9413, 959.3966, 975.4483, 976.4208, 980.4021, 983.4562, 1065.9443, 1076.4495, 1083.4282, 1104.4679, 1132.5159, 1196.4419, 1215.5637, 1235.5752, 1258.5780, 1287.5711, 1297.5441, 1300.6026, 1310.5953, 1311.6184, 1319.5820, 1336.3908, 1350.5619, 1352.5712, 1427.4641, 1439.7198, 1443.6886, 1485.7681, 1499.7430, 1508.6825, 1558.7275, 1574.7196, 1585.8063, 1634.7576, 1834.8681, 1927.9091, 1993.9450, 2067.6716, 2069.7862, 2084.0093, 2193.1061, 2362.2237, 2890.4720

26. Q4IVR0_AZOVI **Mass:** 36505 **Score:** 50 **Expect:** 31 **Queries matched:** 8

Regulatory protein, ArsR.- Azotobacter vinelandii AvOP.

Observed	Mr(expt)	Mr(calc)	Delta	Start	End	Miss	Peptide
975.4483	974.4410	974.5297	-0.0887	313 -	320	0	R.NGFQIQIR.H
1076.4495	1075.4422	1075.5219	-0.0797	211 -	219	0	R.ECLENVELK.L
1132.5159	1131.5087	1131.5019	0.0068	129 -	137	1	R.DFFSRMADK.F + Oxidation (M)
1215.5637	1214.5564	1214.6771	-0.1207	84 -	96	0	R.ALPAAAGFAGTLR.A
1218.5811	1217.5738	1217.6438	-0.0700	260 -	270	0	R.GGSLLLTELCR.H + Carbamidomethyl (C)
1310.5953	1309.5880	1309.5166	0.0714	11 -	22	0	R.DDDCDELAALCK.A
1976.9265	1975.9192	1975.8615	0.0577	11 -	29	1	R.DDDCDELAALCKAGGDPLR.L
2193.1061	2192.0988	2192.1048	-0.0060	134 -	152	1	R.MADKFQAQQDLIAGLPQYR.D

No match to: 854.9449, 860.9786, 876.9413, 959.3966, 976.4208, 980.4021, 983.4562, 1065.9443, 1083.4282, 1104.4679, 1196.4419, 1231.5737, 1235.5752, 1258.5780, 1287.5711, 1297.5441, 1300.6026, 1311.6184, 1319.5820, 1336.3908, 1350.5619, 1352.5712, 1407.6373, 1427.4641, 1439.7198, 1443.6886, 1485.7681, 1499.7430, 1508.6825, 1558.7275, 1574.7196, 1585.8063, 1634.7576, 1656.7520, 1707.7763, 1805.8453, 1834.8681, 1927.9091, 1993.9450, 2045.0624, 2067.6716, 2069.7862, 2084.0093, 2211.0997, 2362.2237, 2890.4720

27. Q1RSK3_MEDTR **Mass:** 105088 **Score:** 50 **Expect:** 32 **Queries matched:** 14

Zinc finger, RING-type; Transcription factor jumonji, jmjC.- Medicago truncatula (Barrel medic).

Observed	Mr(expt)	Mr(calc)	Delta	Start	End	Miss	Peptide
980.4021	979.3948	979.4467	-0.0519	664 -	671	0	R.TNVEMMQK.T
983.4562	982.4489	982.3922	0.0567	34 -	42	0	R.CTAMSMPDK.T
1196.4419	1195.4346	1195.4961	-0.0615	422 -	431	0	K.NVEEMVSGCR.T + Carbamidomethyl (C); Oxidation (M)
1215.5637	1214.5564	1214.4986	0.0578	680 -	690	0	K.ESHGDPDICSR.A
1231.5737	1230.5665	1230.6092	-0.0427	702 -	712	0	K.INGLDLESDQK.D
1310.5953	1309.5880	1309.5860	0.0020	292 -	301	0	R.EQCFEVELEK.K + Carbamidomethyl (C)
1319.5820	1318.5747	1318.6703	-0.0956	43 -	53	1	K.TVCEKHYIQAK.K
1350.5619	1349.5546	1349.6802	-0.1256	764 -	773	1	K.VTEYLKMHWK.E + Oxidation (M)
1352.5712	1351.5639	1351.6013	-0.0374	207 -	216	1	K.DVTWCLKCDR.R + 2 Carbamidomethyl (C)
1485.7681	1484.7608	1484.7511	0.0097	620 -	632	0	K.IYISYGISDELGR.G
1508.6825	1507.6753	1507.6516	0.0236	239 -	250	1	K.ICPACRGICNCK.I + 4 Carbamidomethyl (C)
1834.8681	1833.8608	1833.8493	0.0115	354 -	369	0	R.EATLHQSEEPQTEHAK.T
2211.0997	2210.0924	2210.0235	0.0689	528 -	546	0	K.AIDCLDGSEIDIELNQFMK.G + Carbamidomethyl (C)
2362.2237	2361.2164	2361.1787	0.0377	645 -	663	1	R.DMVYLLVHSSEVQLKDWQR.T + Oxidation (M)

No match to: 854.9449, 860.9786, 876.9413, 959.3966, 975.4483, 976.4208, 1065.9443, 1076.4495, 1083.4282, 1104.4679, 1132.5159, 1218.5811, 1235.5752, 1258.5780, 1287.5711, 1297.5441, 1300.6026, 1311.6184, 1336.3908, 1407.6373, 1427.4641, 1439.7198, 1443.6886, 1499.7430, 1558.7275, 1574.7196, 1585.8063, 1634.7576, 1656.7520, 1707.7763, 1805.8453, 1927.9091, 1976.9265, 1993.9450, 2045.0624, 2067.6716, 2069.7862, 2084.0093, 2193.1061, 2890.4720

28. Q1ZTR3_9VIBR **Mass:** 31022 **Score:** 50 **Expect:** 33 **Queries matched:** 8

Putative putative phospholipid biosynthesis acyltransferase.- Vibrio angustum S14.

Observed	Mr(expt)	Mr(calc)	Delta	Start	End	Miss	Peptide
1132.5159	1131.5087	1131.5416	-0.0330	113 -	121	0	R.LPQCDCLVK.A + 2 Carbamidomethyl (C)
1350.5619	1349.5546	1349.5769	-0.0223	144 -	155	0	R.DPESLLDSCSER.L
1407.6373	1406.6300	1406.5984	0.0317	144 -	155	0	R.DPESLLDSCSER.L + Carbamidomethyl (C)
1558.7275	1557.7202	1557.8514	-0.1312	156 -	170	0	R.LGSGNVLLVFPEGTR.T
2084.0093	2083.0020	2083.0069	-0.0049	228 -	247	1	K.DKIEVGSFIESSDSATTAAR.K
2193.1061	2192.0988	2192.0167	0.0821	136 -	155	1	K.AAGYIPNRDPESLLDSCSER.L
2211.0997	2210.0924	2210.1881	-0.0957	93 -	112	0	R.NCLIVANHPSLIDYVLIASR.L
2890.4720	2889.4647	2889.4177	0.0470	144 -	170	1	R.DPESLLDSCSERLGSGNVLLVFPEGTR.T

No match to: 854.9449, 860.9786, 876.9413, 959.3966, 975.4483, 976.4208, 980.4021, 983.4562, 1065.9443, 1076.4495, 1083.4282, 1104.4679, 1196.4419, 1215.5637, 1218.5811, 1231.5737, 1235.5752, 1258.5780, 1287.5711, 1297.5441, 1300.6026, 1310.5953, 1311.6184, 1319.5820, 1336.3908, 1352.5712, 1427.4641, 1439.7198, 1443.6886, 1485.7681, 1499.7430, 1508.6825, 1574.7196, 1585.8063, 1634.7576, 1656.7520, 1707.7763, 1805.8453, 1834.8681, 1927.9091, 1976.9265, 1993.9450, 2045.0624, 2067.6716, 2069.7862, 2362.2237

29. Q390G0_BURS3 **Mass:** 100380 **Score:** 50 **Expect:** 33 **Queries matched:** 13

ATP-dependent transcriptional regulator, MalT-like, LuxR family.- Burkholderia sp. (strain 383) (Burkholderia cepacia (strain ATCC 17760 / NCIB 9086 / R18194))

Observed	Mr(expt)	Mr(calc)	Delta	Start	End	Miss	Peptide

975.4483	974.4410	974.5185	-0.0775	412 - 419	0	R.TVLDWVSR.L
976.4208	975.4135	975.4985	-0.0850	907 - 916	0	R.SGLALDASSR.T
980.4021	979.3948	979.4579	-0.0631	673 - 681	0	R.VACMATGVR.L + Carbamidomethyl (C); Oxidation (M)
1132.5159	1131.5087	1131.5771	-0.0684	488 - 497	0	R.SIESLELGER.V
1287.5711	1286.5638	1286.6805	-0.1167	289 - 299	0	R.HFLLCTAVLDR.M
1311.6184	1310.6111	1310.6805	-0.0693	635 - 645	0	R.FMQTAAVLQFR.S
1634.7576	1633.7503	1633.8320	-0.0817	620 - 634	0	R.FDIAMQACSLGPLLR.F
1656.7520	1655.7447	1655.8599	-0.1152	673 - 688	1	R.VACMATGVRLHVSAGK.L + Carbamidomethyl (C)
1707.7763	1706.7690	1706.8484	-0.0794	620 - 634	0	R.FDIAMQACSLGPLLR.F + Carbamidomethyl (C); Oxidation (M)
1927.9091	1926.9019	1926.9808	-0.0789	433 - 449	0	R.IAHVWALALSMQMVDAR.R + Oxidation (M)
2045.0624	2044.0551	2044.0800	-0.0249	479 - 497	1	R.SLIAGLSDRSIESLELGER.V
2084.0093	2083.0020	2083.0819	-0.0799	433 - 450	1	R.IAHVWALALSMQMVDARR.A + Oxidation (M)
2362.2237	2361.2164	2361.1230	0.0934	1 - 23	1	-.MTSSDSRQAAGPADADGIEVLVR.T + Oxidation (M)

No match to: 854.9449, 860.9786, 876.9413, 959.3966, 983.4562, 1065.9443, 1076.4495, 1083.4282, 1104.4679, 1196.4419, 1215.5637, 1218.5811, 1231.5737, 1235.5752, 1258.5780, 1297.5441, 1300.6026, 1310.5953, 1319.5820, 1336.3908, 1350.5619, 1352.5712, 1407.6373, 1427.4641, 1439.7198, 1443.6886, 1485.7681, 1499.7430, 1508.6825, 1558.7275, 1574.7196, 1585.8063, 1805.8453, 1834.8681, 1976.9265, 1993.9450, 2067.6716, 2069.7862, 2193.1061, 2211.0997, 2890.4720

30. Q8TA35_HETGL **Mass:** 25472 **Score:** 50 **Expect:** 34 **Queries matched:** 7

Dorsal gland cell protein Hgg-26 (Fragment).- Heterodera glycines (Soybean cyst nematode worm).

Observed	Mr(expt)	Mr(calc)	Delta	Start - End	Miss	Peptide
1083.4282	1082.4209	1082.5145	-0.0936	157 - 165	0	R.AADFYNINR.L
1132.5159	1131.5087	1131.5270	-0.0183	82 - 89	1	K.LCEEYKYK.F + Carbamidomethyl (C)
1196.4419	1195.4346	1195.4993	-0.0647	217 - 227	0	R.STSWDESVQGT.-
1352.5712	1351.5639	1351.6004	-0.0365	216 - 227	1	R.RSTSWDESVQGT.-
1927.9091	1926.9019	1926.9257	-0.0239	150 - 165	1	K.TDLEMVRAADFYNINR.L
2084.0093	2083.0020	2082.9528	0.0492	196 - 215	1	R.DSASKSPYPPGTSEMGTSLR.R + Oxidation (M)
2890.4720	2889.4647	2889.4184	0.0463	157 - 180	1	R.AADFYNINRLINDDYIDNVYEIVK.V

No match to: 854.9449, 860.9786, 876.9413, 959.3966, 975.4483, 976.4208, 980.4021, 983.4562, 1065.9443, 1076.4495, 1104.4679, 1215.5637, 1218.5811, 1231.5737, 1235.5752, 1258.5780, 1287.5711, 1297.5441, 1300.6026, 1310.5953, 1311.6184, 1319.5820, 1336.3908, 1350.5619, 1407.6373, 1427.4641, 1439.7198, 1443.6886, 1485.7681, 1499.7430, 1508.6825, 1558.7275, 1574.7196, 1585.8063, 1634.7576, 1656.7520, 1707.7763, 1805.8453, 1834.8681, 1976.9265, 1993.9450, 2045.0624, 2067.6716, 2069.7862, 2193.1061, 2211.0997, 2362.2237

31. Q7XUX8_ORYSA **Mass:** 41458 **Score:** 50 **Expect:** 36 **Queries matched:** 9

OSJNBa0027G07.11 protein.- Oryza sativa (japonica cultivar-group).

Observed	Mr(expt)	Mr(calc)	Delta	Start - End	Miss	Peptide
1218.5811	1217.5738	1217.5564	0.0174	206 - 215	0	K.EDDAWVQDLK.L
1350.5619	1349.5546	1349.5427	0.0119	313 - 324	0	R.GWQNNGCCPLCR.R
1407.6373	1406.6300	1406.5642	0.0659	313 - 324	0	R.GWQNNGCCPLCR.R + Carbamidomethyl (C)
1443.6886	1442.6813	1442.7187	-0.0374	326 - 338	0	R.ETETALHLVATCR.Y
1585.8063	1584.7990	1584.8347	-0.0357	291 - 302	1	K.CKFHAWLIIQNR.V + Carbamidomethyl (C)
1656.7520	1655.7447	1655.8413	-0.0966	325 - 338	1	R.RETETALHLVATCR.Y + Carbamidomethyl (C)
1993.9450	1992.9377	1992.9608	-0.0231	62 - 79	1	K.NMAAAGRTMLESLEQLDK.Q + Oxidation (M)
2211.0997	2210.0924	2210.0823	0.0101	104 - 123	1	K.TCMPTSQGGLGVLNLDKFTR.A + Carbamidomethyl (C); Oxidation (M)
2362.2237	2361.2164	2361.2032	0.0132	99 - 120	1	K.VNLTKTCMPTSQGGLGVLNLDK.F + Carbamidomethyl (C); Oxidation (M)

No match to: 854.9449, 860.9786, 876.9413, 959.3966, 975.4483, 976.4208, 980.4021, 983.4562, 1065.9443, 1076.4495, 1083.4282, 1104.4679, 1132.5159, 1196.4419, 1215.5637, 1231.5737, 1235.5752, 1258.5780, 1287.5711, 1297.5441, 1300.6026, 1310.5953, 1311.6184, 1319.5820, 1336.3908, 1352.5712, 1427.4641, 1439.7198, 1485.7681, 1499.7430, 1508.6825, 1558.7275, 1574.7196, 1634.7576, 1707.7763, 1805.8453, 1834.8681, 1927.9091, 1976.9265, 2045.0624, 2067.6716, 2069.7862, 2084.0093, 2193.1061, 2890.4720

32. Q6IQ22_HUMAN **Mass:** 11751 **Score:** 50 **Expect:** 36 **Queries matched:** 6

RAB12, member RAS oncogene family.- Homo sapiens (Human).

Observed	Mr(expt)	Mr(calc)	Delta	Start - End	Miss	Peptide
983.4562	982.4489	982.5308	-0.0819	2 - 10	1	M.DPGAALQRR.A
1218.5811	1217.5738	1217.6727	-0.0990	73 - 85	1	K.STVGKGATATLGR.A
1300.6026	1299.5954	1299.6142	-0.0188	86 - 97	0	R.ASWRPGSCPSPR.G
1336.3908	1335.3835	1335.5111	-0.1276	62 - 72	0	R.FTDDTFCEACK.S + Carbamidomethyl (C)
1508.6825	1507.6753	1507.7895	-0.1142	101 - 114	1	R.VSSSLGVFRGWASR.-
2069.7862	2068.7789	2068.8540	-0.0751	56 - 72	1	K.TSLMERFTDDTFCEACK.S + Carbamidomethyl (C); Oxidation (M)

No match to: 854.9449, 860.9786, 876.9413, 959.3966, 975.4483, 976.4208, 980.4021, 1065.9443, 1076.4495, 1083.4282, 1104.4679, 1132.5159, 1196.4419, 1215.5637, 1231.5737, 1235.5752, 1258.5780, 1287.5711, 1297.5441, 1310.5953, 1311.6184, 1319.5820, 1350.5619, 1352.5712, 1407.6373, 1427.4641, 1439.7198, 1443.6886, 1485.7681, 1499.7430, 1558.7275, 1574.7196, 1585.8063, 1634.7576, 1656.7520, 1707.7763, 1805.8453, 1834.8681, 1927.9091, 1976.9265, 1993.9450, 2045.0624, 2067.6716, 2084.0093, 2193.1061, 2211.0997, 2362.2237, 2890.4720

33. AH3404 **Mass:** 20696 **Score:** 49 **Expect:** 37 **Queries matched:** 8

hypothetical protein BMEI1222 [imported] - Brucella melitensis (strain 16M)

Observed	Mr(expt)	Mr(calc)	Delta	Start - End	Miss	Peptide
1083.4282	1082.4209	1082.4485	-0.0276	6 - 14	0	R.GSCQMTDLR.F + Carbamidomethyl (C); Oxidation (M)
1215.5637	1214.5564	1214.6441	-0.0877	176 - 186	1	K.SLMQPRIGQSV.-
1231.5737	1230.5665	1230.6390	-0.0726	176 - 186	1	K.SLMQPRIGQSV.- + Oxidation (M)
1235.5752	1234.5679	1234.6202	-0.0523	130 - 139	0	R.TEMVIWAMVR.Q
1258.5780	1257.5707	1257.6564	-0.0858	50 - 61	0	R.AELAADISLEAR.G
1310.5953	1309.5880	1309.5867	0.0013	4 - 14	1	K.SRGSCQMTDLR.F + Carbamidomethyl (C)
1407.6373	1406.6300	1406.7162	-0.0862	129 - 139	1	R.RTEMVIWAMVR.Q + Oxidation (M)
1558.7275	1557.7202	1557.8402	-0.1200	97 - 111	0	R.ADVVSFQVPSPATLK.F

No match to: 854.9449, 860.9786, 876.9413, 959.3966, 975.4483, 976.4208, 980.4021, 983.4562, 1065.9443, 1076.4495, 1104.4679, 1132.5159, 1196.4419, 1218.5811, 1287.5711, 1297.5441, 1300.6026, 1311.6184, 1319.5820, 1336.3908, 1350.5619, 1352.5712, 1427.4641, 1439.7198, 1443.6886, 1485.7681, 1499.7430, 1508.6825, 1574.7196, 1585.8063, 1634.7576, 1656.7520, 1707.7763, 1805.8453, 1834.8681, 1927.9091, 1976.9265, 1993.9450, 2045.0624, 2067.6716, 2069.7862, 2084.0093, 2193.1061, 2211.0997, 2362.2237, 2890.4720

34. AAN43115 **Mass:** 54614 **Score:** 49 **Expect:** 39 **Queries matched:** 9

AE005674 NID: - Shigella flexneri 2a str. 301

Observed	Mr(expt)	Mr(calc)	Delta	Start	End	Miss	Peptide
1310.5953	1309.5880	1309.6965	-0.1085	1	10	1	-.MTLSFITRWR.D
1407.6373	1406.6300	1406.7405	-0.1105	147	159	0	R.ALSIVTSDSPVYR.E
1499.7430	1498.7357	1498.7892	-0.0535	425	437	1	R.NWLAQRAIEAAEK.G
1558.7275	1557.7202	1557.7265	-0.0063	178	188	1	R.FGHFEHFYYRR.E
1656.7520	1655.7447	1655.8301	-0.0854	366	380	0	R.MLSLTEQHSAASPLR.E + Oxidation (M)
1707.7763	1706.7690	1706.8344	-0.0654	438	451	1	K.GDMMELHRLHEALR.N
1993.9450	1992.9377	1993.0237	-0.0859	160	177	1	R.ETVEPGAMLMRVAPSHLR.F
2045.0624	2044.0551	2044.0524	0.0027	363	380	1	R.TFRMLSLTEQHSAASPLR.E
2211.0997	2210.0924	2210.1405	-0.0481	88	107	0	R.GILLGEQLLADGTTMDWHLK.G

No match to: 854.9449, 860.9786, 876.9413, 959.3966, 975.4483, 976.4208, 980.4021, 983.4562, 1065.9443, 1076.4495, 1083.4282, 1104.4679, 1132.5159, 1196.4419, 1215.5637, 1218.5811, 1231.5737, 1235.5752, 1258.5780, 1287.5711, 1297.5441, 1300.6026, 1311.6184, 1319.5820, 1336.3908, 1350.5619, 1352.5712, 1427.4641, 1439.7198, 1443.6886, 1485.7681, 1508.6825, 1574.7196, 1585.8063, 1634.7576, 1805.8453, 1834.8681, 1927.9091, 1976.9265, 2067.6716, 2069.7862, 2084.0093, 2193.1061, 2362.2237, 2890.4720

35. Q2RNF9_RHORT **Mass:** 80918 **Score:** 49 **Expect:** 39 **Queries matched:** 11

Malate dehydrogenase (EC 1.1.1.40) (EC 2.3.1.8).- Rhodospirillum rubrum (strain ATCC 11170 / NCIB 8255).

Observed	Mr(expt)	Mr(calc)	Delta	Start	End	Miss	Peptide
1231.5737	1230.5665	1230.6720	-0.1056	466	477	0	R.NAGYGTPILIGR.A
1287.5711	1286.5638	1286.6077	-0.0439	559	569	0	R.HFHSCLSDITK.V
1350.5619	1349.5546	1349.5993	-0.0448	1	11	1	-.MADETNRNLDR.E + Oxidation (M)
1499.7430	1498.7357	1498.8177	-0.0820	390	404	0	R.LIAAISPAVAEAAMR.S + Oxidation (M)
1508.6825	1507.6753	1507.7340	-0.0587	447	459	1	R.VAFAEGEEEKMIR.A
1585.8063	1584.7990	1584.8042	-0.0051	217	231	1	R.SANVTLCDSKGVVHR.G
1634.7576	1633.7503	1633.7695	-0.0193	359	373	1	R.EDVPDEVAAAYSGRR.L
1656.7520	1655.7447	1655.7429	0.0018	212	226	1	K.AMGMRSANVTLCDSK.G + Carbamidomethyl (C); Oxidation (M)
1707.7763	1706.7690	1706.8780	-0.1090	376	389	1	R.YGRDYIIPVPFDPR.L
1805.8453	1804.8380	1804.8995	-0.0615	503	517	0	K.LSTQTKPYTDYLYGR.L
2193.1061	2192.0988	2192.0638	0.0350	481	502	0	K.IAATMAAMGLGGLEGMEIQNAK.L + Oxidation (M)

No match to: 854.9449, 860.9786, 876.9413, 959.3966, 975.4483, 976.4208, 980.4021, 983.4562, 1065.9443, 1076.4495, 1083.4282, 1104.4679, 1132.5159, 1196.4419, 1215.5637, 1218.5811, 1235.5752, 1258.5780, 1297.5441, 1300.6026, 1310.5953, 1311.6184, 1319.5820, 1336.3908, 1352.5712, 1407.6373, 1427.4641, 1439.7198, 1443.6886, 1485.7681, 1558.7275, 1574.7196, 1834.8681, 1927.9091, 1976.9265, 1993.9450, 2045.0624, 2067.6716, 2069.7862, 2084.0093, 2211.0997, 2362.2237, 2890.4720

36. T08180 **Mass:** 65798 **Score:** 49 **Expect:** 42 **Queries matched:** 10

PF20 protein, microtubule-associated - Chlamydomonas reinhardtii

Observed	Mr(expt)	Mr(calc)	Delta	Start	End	Miss	Peptide
1083.4282	1082.4209	1082.4404	-0.0195	11	19	0	R.LEDSDDDFK.Y
1215.5637	1214.5564	1214.6230	-0.0666	436	445	1	R.LWDLPAGKCR.M + Carbamidomethyl (C)
1231.5737	1230.5665	1230.5564	0.0101	537	546	1	K.HPANKSCFDR.S + Carbamidomethyl (C)
1235.5752	1234.5679	1234.6227	-0.0548	525	536	0	R.MTAEVATINTGK.H
1311.6184	1310.6111	1310.6843	-0.0732	289	300	1	R.SGWASLNAPPRR.N
1319.5820	1318.5747	1318.6187	-0.0439	547	559	0	R.SGQVLAVACDDGK.V + Carbamidomethyl (C)
1707.7763	1706.7690	1706.6956	0.0734	91	103	0	R.TCECFEAEWYELK.A + Carbamidomethyl (C)
1927.9091	1926.9019	1926.8563	0.0455	542	559	1	K.SCFDRSGQVLAVACDDGK.V + Carbamidomethyl (C)
2211.0997	2210.0924	2209.9482	0.1442	86	103	1	K.MGLSRTCECFEAEWYELK.A + Oxidation (M)
2362.2237	2361.2164	2361.0162	0.2001	349	368	1	K.TWKMWHMPGGDLIMCGEGHK.D + 3 Oxidation (M)

No match to: 854.9449, 860.9786, 876.9413, 959.3966, 975.4483, 976.4208, 980.4021, 983.4562, 1065.9443, 1076.4495, 1104.4679, 1132.5159, 1196.4419, 1218.5811, 1258.5780, 1287.5711, 1297.5441, 1300.6026, 1310.5953, 1336.3908, 1350.5619, 1352.5712, 1407.6373, 1427.4641, 1439.7198, 1443.6886, 1485.7681, 1499.7430, 1508.6825, 1558.7275, 1574.7196, 1585.8063, 1634.7576, 1656.7520, 1805.8453, 1834.8681, 1976.9265, 1993.9450, 2045.0624, 2067.6716, 2069.7862, 2084.0093, 2193.1061, 2890.4720

37. Q7V0J9_PROMP **Mass:** 38698 **Score:** 48 **Expect:** 46 **Queries matched:** 8

Possible dTDP-glucose 4,6-dehydratase (EC 4.2.1.46).- Prochlorococcus marinus subsp. pastoris (strain CCMP 1378 / MED4).

Observed	Mr(expt)	Mr(calc)	Delta	Start	End	Miss	Peptide
976.4208	975.4135	975.4338	-0.0203	322	328	0	K.SWTYEYK.D
1076.4495	1075.4422	1075.5332	-0.0910	256	264	0	K.ICNLTNVDK.S + Carbamidomethyl (C)
1235.5752	1234.5679	1234.6305	-0.0626	203	213	1	R.NFILDGKGESR.R
1319.5820	1318.5747	1318.6663	-0.0916	256	266	1	K.ICNLTNVDKSR.I + Carbamidomethyl (C)
1499.7430	1498.7357	1498.7721	-0.0364	35	45	1	R.VKDHYQWPWIK.K
1834.8681	1833.8608	1833.9236	-0.0628	163	176	1	R.CLYKQYNFPYLIGR.F + Carbamidomethyl (C)
1927.9091	1926.9019	1927.0237	-0.1218	109	124	1	R.LMNALIDSSFLQKYIR.I + Oxidation (M)
2084.0093	2083.0020	2083.0737	-0.0718	122	139	1	K.YIRIGTPEVFGSNENFLK.E

No match to: 854.9449, 860.9786, 876.9413, 959.3966, 975.4483, 980.4021, 983.4562, 1065.9443, 1083.4282, 1104.4679, 1132.5159, 1196.4419, 1215.5637, 1218.5811, 1231.5737, 1258.5780, 1287.5711, 1297.5441, 1300.6026, 1310.5953, 1311.6184, 1336.3908, 1350.5619, 1352.5712, 1407.6373, 1427.4641, 1439.7198, 1443.6886, 1485.7681, 1508.6825, 1558.7275, 1574.7196, 1585.8063, 1634.7576, 1656.7520, 1707.7763, 1805.8453, 1976.9265, 1993.9450, 2045.0624, 2067.6716, 2069.7862, 2193.1061, 2211.0997, 2362.2237, 2890.4720

38. Q5ZI47_CHICK **Mass:** 101099 **Score:** 48 **Expect:** 49 **Queries matched:** 13

Hypothetical protein.- Gallus gallus (Chicken).

Observed	Mr(expt)	Mr(calc)	Delta	Start		End	Miss	Peptide
976.4208	975.4135	975.4695	-0.0560	605	-	612	0	R.MTETPELR.S
1104.4679	1103.4606	1103.4805	-0.0199	558	-	566	0	R.LECEEPEAK.G + Carbamidomethyl (C)
1196.4419	1195.4346	1195.5399	-0.1053	926	-	935	1	R.CAIEADMKMK.K + Carbamidomethyl (C)
1231.5737	1230.5665	1230.6091	-0.0427	151	-	160	1	K.LEEEEAEVKR.K
1311.6184	1310.6111	1310.5772	0.0340	442	-	456	0	K.ECLGSGAGSTAGSSK.D
1350.5619	1349.5546	1349.6570	-0.1025	138	-	148	0	R.LEELCNEIMIK.K + Oxidation (M)
1407.6373	1406.6300	1406.6785	-0.0485	138	-	148	0	R.LEELCNEIMIK.K + Carbamidomethyl (C); Oxidation (M)
1439.7198	1438.7125	1438.6721	0.0403	441	-	456	1	K.KECLGSGAGSTAGSSK.D
1443.6886	1442.6813	1442.7616	-0.0803	86	-	98	0	K.GEVVETVEDVIVR.K
1485.7681	1484.7608	1484.6678	0.0930	125	-	137	0	K.DAELIQAGHMDNR.L + Oxidation (M)
1508.6825	1507.6753	1507.8194	-0.1441	788	-	800	1	K.AIMLVWRAAANHR.Y
1993.9450	1992.9377	1992.9099	0.0279	63	-	79	0	K.HCASQYSELLETTETPK.R + Carbamidomethyl (C)
2211.0997	2210.0924	2210.0572	0.0352	25	-	44	1	K.LCLASSVMRSGDQNWVSVSR.A + Oxidation (M)

No match to: 854.9449, 860.9786, 876.9413, 959.3966, 975.4483, 980.4021, 983.4562, 1065.9443, 1076.4495, 1083.4282, 1132.5159, 1215.5637, 1218.5811, 1235.5752, 1258.5780, 1287.5711, 1297.5441, 1300.6026, 1310.5953, 1319.5820, 1336.3908, 1352.5712, 1427.4641, 1499.7430, 1558.7275, 1574.7196, 1585.8063, 1634.7576, 1656.7520, 1707.7763, 1805.8453, 1834.8681, 1927.9091, 1976.9265, 2045.0624, 2067.6716, 2069.7862, 2084.0093, 2193.1061, 2362.2237, 2890.4720

39. Q4DLZ9_TRYCR **Mass:** 19461 **Score:** 48 **Expect:** 51 **Queries matched:** 7

Protein tyrosine phosphatase-like protein, putative.- Trypanosoma cruzi.

Observed	Mr(expt)	Mr(calc)	Delta	Start		End	Miss	Peptide
1350.5619	1349.5546	1349.6431	-0.0885	1	-	12	1	-.MEANGTLVECKR.G
1407.6373	1406.6300	1406.6646	-0.0346	1	-	12	1	-.MEANGTLVECKR.G + Carbamidomethyl (C)
1443.6886	1442.6813	1442.6572	0.0242	160	-	172	0	R.HQESNEGSLICAR.C
1485.7681	1484.7608	1484.6605	0.1003	51	-	63	0	R.ACGPTYNAEVFEK.Q + Carbamidomethyl (C)
1558.7275	1557.7202	1557.8085	-0.0883	143	-	155	0	K.GAINQVQLNWLMR.Y + Oxidation (M)
2045.0624	2044.0551	2043.9908	0.0643	156	-	172	1	R.YKPRHQESNEGSLICAR.C + Carbamidomethyl (C)
2362.2237	2361.2164	2361.2766	-0.0602	22	-	42	1	R.FLIIDAPSPSSVPAYMKLLQR.H + Oxidation (M)

No match to: 854.9449, 860.9786, 876.9413, 959.3966, 975.4483, 976.4208, 980.4021, 983.4562, 1065.9443, 1076.4495, 1083.4282, 1104.4679, 1132.5159, 1196.4419, 1215.5637, 1218.5811, 1231.5737, 1235.5752, 1258.5780, 1287.5711, 1297.5441, 1300.6026, 1310.5953, 1311.6184, 1319.5820, 1336.3908, 1352.5712, 1427.4641, 1439.7198, 1499.7430, 1508.6825, 1574.7196, 1585.8063, 1634.7576, 1656.7520, 1707.7763, 1805.8453, 1834.8681, 1927.9091, 1976.9265, 1993.9450, 2067.6716, 2069.7862, 2084.0093, 2193.1061, 2211.0997, 2890.4720

40. Q39YY1_GEOMG **Mass:** 16521 **Score:** 48 **Expect:** 53 **Queries matched:** 7

Putative PAS/PAC sensor protein.- Geobacter metallireducens (strain GS-15 / ATCC 53774 / DSM 7210).

Observed	Mr(expt)	Mr(calc)	Delta	Start		End	Miss	Peptide
1215.5637	1214.5564	1214.6441	-0.0877	84	-	96	0	K.LLSAPALCSAGGR.I
1319.5820	1318.5747	1318.6551	-0.0803	1	-	11	1	-.MPDTTNDKLLR.L + Oxidation (M)
1350.5619	1349.5546	1349.6067	-0.0521	108	-	121	0	R.DCEGAMAGSGAIIR.D
1352.5712	1351.5639	1351.6078	-0.0439	72	-	83	0	R.VMETGETHYGTK.L
1407.6373	1406.6300	1406.6282	0.0018	108	-	121	0	R.DCEGAMAGSGAIIR.D + Carbamidomethyl (C)
1508.6825	1507.6753	1507.7089	-0.0336	71	-	83	1	R.RVMETGETHYGTK.L
1585.8063	1584.7990	1584.8511	-0.0521	12	-	26	0	R.LIAEGVPDAVIFADR.E

No match to: 854.9449, 860.9786, 876.9413, 959.3966, 975.4483, 976.4208, 980.4021, 983.4562, 1065.9443, 1076.4495, 1083.4282, 1104.4679, 1132.5159, 1196.4419, 1218.5811, 1231.5737, 1235.5752, 1258.5780, 1287.5711, 1297.5441, 1300.6026, 1310.5953, 1311.6184, 1336.3908, 1427.4641, 1439.7198, 1443.6886, 1485.7681, 1499.7430, 1558.7275, 1574.7196, 1634.7576, 1656.7520, 1707.7763, 1805.8453, 1834.8681, 1927.9091, 1976.9265, 1993.9450, 2045.0624, 2067.6716, 2069.7862, 2084.0093, 2193.1061, 2211.0997, 2362.2237, 2890.4720

41. Q9TUL8_HORSE **Mass:** 42919 **Score:** 48 **Expect:** 54 **Queries matched:** 8

Matrix metalloproteinase-2 (Fragment).- Equus caballus (Horse).

Observed	Mr(expt)	Mr(calc)	Delta	Start		End	Miss	Peptide
1132.5159	1131.5087	1131.4502	0.0584	135	-	144	0	K.EYTSCTDTGR.S
1310.5953	1309.5880	1309.6931	-0.1051	1	-	10	1	-.NYNFFPRKPK.W
1407.6373	1406.6300	1406.5891	0.0409	68	-	79	0	R.WEHGDGYPFDGK.D
1634.7576	1633.7503	1633.6137	0.1366	265	-	277	0	K.MWCATTANYDDDR.K + Carbamidomethyl (C); Oxidation (M)
1993.9450	1992.9377	1992.9475	-0.0098	51	-	67	1	R.FSRIHDGEADIMINFGR.W + Oxidation (M)
2069.7862	2068.7789	2068.9352	-0.1563	117	-	134	1	K.YGNADGEYCKFPFLFNGK.E
2084.0093	2083.0020	2082.8993	0.1027	145	-	162	1	R.SDGFLWCSTTYNFDKDGK.Y
2890.4720	2889.4647	2889.2824	0.1823	163	-	188	1	K.YGFCPHEALFTMGGNADGQPCKFPFR.F

No match to: 854.9449, 860.9786, 876.9413, 959.3966, 975.4483, 976.4208, 980.4021, 983.4562, 1065.9443, 1076.4495, 1083.4282, 1104.4679, 1196.4419, 1215.5637, 1218.5811, 1231.5737, 1235.5752, 1258.5780, 1287.5711, 1297.5441, 1300.6026, 1311.6184, 1319.5820, 1336.3908, 1350.5619, 1352.5712, 1427.4641, 1439.7198, 1443.6886, 1485.7681, 1499.7430, 1508.6825, 1558.7275, 1574.7196, 1585.8063, 1656.7520, 1707.7763, 1805.8453, 1834.8681, 1927.9091, 1976.9265, 2045.0624, 2067.6716, 2193.1061, 2211.0997, 2362.2237

42. Q3M485_ANAVT **Mass:** 42516 **Score:** 48 **Expect:** 55 **Queries matched:** 9

Hydrogenase formation HypD protein.- Anabaena variabilis (strain ATCC 29413 / PCC 7937).

Observed	Mr(expt)	Mr(calc)	Delta	Start		End	Miss	Peptide
1258.5780	1257.5707	1257.6928	-0.1222	372	-	383	1	K.QAAEKPKVTISS.-
1311.6184	1310.6111	1310.6142	-0.0031	3	-	12	1	K.YVDEFREPEK.A
1508.6825	1507.6753	1507.7704	-0.0952	117	-	129	0	R.MVYSPLDSLQIAR.N + Oxidation (M)
1558.7275	1557.7202	1557.7431	-0.0229	33	-	46	0	K.IMEVCGGHTHSIFK.Y
1574.7196	1573.7123	1573.7381	-0.0258	33	-	46	0	K.IMEVCGGHTHSIFK.Y + Oxidation (M)
1927.9091	1926.9019	1927.0097	-0.1079	257	-	273	1	K.QGNQIALEAMHKVFAVR.E + Oxidation (M)

1993.9450	1992.9377	1993.0025	-0.0648	30 - 46	1	K.HIKIMEVCGGHTHSIFK.Y + Carbamidomethyl (C)
2084.0093	2083.0020	2083.0520	-0.0500	117 - 134	1	R.MVYSPLDSLQIARNHPDK.E
2193.1061	2192.0988	2192.1259	-0.0271	110 - 129	1	K.ATGADIRMVYSPLDSLQIAR.N + Oxidation (M)

No match to: 854.9449, 860.9786, 876.9413, 959.3966, 975.4483, 976.4208, 980.4021, 983.4562, 1065.9443, 1076.4495, 1083.4282, 1104.4679, 1132.5159, 1196.4419, 1215.5637, 1218.5811, 1231.5737, 1235.5752, 1287.5711, 1297.5441, 1300.6026, 1310.5953, 1319.5820, 1336.3908, 1350.5619, 1352.5712, 1407.6373, 1427.4641, 1439.7198, 1443.6886, 1485.7681, 1499.7430, 1585.8063, 1634.7576, 1656.7520, 1707.7763, 1805.8453, 1834.8681, 1976.9265, 2045.0624, 2067.6716, 2069.7862, 2211.0997, 2362.2237, 2890.4720

43. Q5NVL1_PONPY **Mass:** 79482 **Score:** 48 **Expect:** 58 **Queries matched:** 11

Hypothetical protein DKFZp459N0220 (Fragment).- Pongo pygmaeus (Orangutan).

Observed	Mr(expt)	Mr(calc)	Delta	Start - End	Miss	Peptide
975.4483	974.4410	974.4855	-0.0445	58 - 65	0	K.LAEMLDQR.Q
1104.4679	1103.4606	1103.5094	-0.0489	552 - 561	0	K.ASQEDANEIK.S
1132.5159	1131.5087	1131.5957	-0.0871	659 - 668	1	R.DKVQLMAAEK.K
1287.5711	1286.5638	1286.5812	-0.0174	9 - 21	0	R.AAGEPGTSMPPEK.K + Oxidation (M)
1311.6184	1310.6111	1310.6726	-0.0615	642 - 651	1	K.EMKLLLDMYR.S
1319.5820	1318.5747	1318.6364	-0.0617	552 - 563	1	K.ASQEDANEIKSK.R
1443.6886	1442.6813	1442.6823	-0.0010	8 - 21	1	K.RAAGEPGTSMPPEK.K + Oxidation (M)
1634.7576	1633.7503	1633.7795	-0.0292	515 - 530	0	R.SGSALLQSQSSTEDPK.D
1656.7520	1655.7447	1655.8492	-0.1045	478 - 491	0	R.HLTSSLQNHNHQLK.G
1927.9091	1926.9019	1927.0162	-0.1144	306 - 324	1	K.VYGAGSSLYGGTITINARK.F
2211.0997	2210.0924	2210.1668	-0.0744	478 - 496	1	R.HLTSSLQNHNHQLKGEVLR.Y

No match to: 854.9449, 860.9786, 876.9413, 959.3966, 976.4208, 980.4021, 983.4562, 1065.9443, 1076.4495, 1083.4282, 1196.4419, 1215.5637, 1218.5811, 1231.5737, 1235.5752, 1258.5780, 1297.5441, 1300.6026, 1310.5953, 1336.3908, 1350.5619, 1352.5712, 1407.6373, 1427.4641, 1439.7198, 1485.7681, 1499.7430, 1508.6825, 1558.7275, 1574.7196, 1585.8063, 1707.7763, 1805.8453, 1834.8681, 1976.9265, 1993.9450, 2045.0624, 2067.6716, 2069.7862, 2084.0093, 2193.1061, 2362.2237, 2890.4720

44. Q4AI58_9CHLB **Mass:** 37062 **Score:** 48 **Expect:** 58 **Queries matched:** 8

Cell shape determining protein MreB/Mrl.- Chlorobium phaeobacteroides BS1.

Observed	Mr(expt)	Mr(calc)	Delta	Start - End	Miss	Peptide
1235.5752	1234.5679	1234.6669	-0.0990	309 - 319	0	K.LSVHVAEDPLR.A
1300.6026	1299.5954	1299.6975	-0.1021	331 - 340	1	K.NFDKFTFLIR.-
1311.6184	1310.6111	1310.6652	-0.0541	285 - 297	0	R.TGIYLAGGGSMLR.G + Oxidation (M)
1350.5619	1349.5546	1349.6860	-0.1315	251 - 262	1	K.EIASCLDKSISK.I + Carbamidomethyl (C)
1439.7198	1438.7125	1438.8031	-0.0906	28 - 40	0	K.VIVDEPSIVALER.N
1585.8063	1584.7990	1584.9086	-0.1096	259 - 273	1	K.SISKIEAAVLNALEK.T
1707.7763	1706.7690	1706.8913	-0.1222	236 - 250	1	R.DMLTGIPKEVTVNYK.E
1976.9265	1975.9192	1976.0466	-0.1274	10 - 27	0	K.EIAIDLGTANTIIIYNDK.V

No match to: 854.9449, 860.9786, 876.9413, 959.3966, 975.4483, 976.4208, 980.4021, 983.4562, 1065.9443, 1076.4495, 1083.4282, 1104.4679, 1132.5159, 1196.4419, 1215.5637, 1218.5811, 1231.5737, 1258.5780, 1287.5711, 1297.5441, 1310.5953, 1319.5820, 1336.3908, 1352.5712, 1407.6373, 1427.4641, 1443.6886, 1485.7681, 1499.7430, 1508.6825, 1558.7275, 1574.7196, 1634.7576, 1656.7520, 1805.8453, 1834.8681, 1927.9091, 1993.9450, 2045.0624, 2067.6716, 2069.7862, 2084.0093, 2193.1061, 2211.0997, 2362.2237, 2890.4720

45. Q2HUK6_MEDTR **Mass:** 39508 **Score:** 47 **Expect:** 65 **Queries matched:** 8

Mitochondrial substrate carrier.- Medicago truncatula (Barrel medic).

Observed	Mr(expt)	Mr(calc)	Delta	Start - End	Miss	Peptide
1076.4495	1075.4422	1075.4790	-0.0368	104 - 112	0	K.CNMQIDPAK.Y + Carbamidomethyl (C)
1258.5780	1257.5707	1257.6942	-0.1235	201 - 211	1	K.VRVQTQPGFAR.G
1310.5953	1309.5880	1309.6158	-0.0278	104 - 114	1	K.CNMQIDPAKYK.S
1311.6184	1310.6111	1310.7234	-0.1122	220 - 231	1	K.FVKSEGALGLYK.G
1350.5619	1349.5546	1349.6761	-0.1215	52 - 64	1	R.GTGSGRFMIPSPK.E + Oxidation (M)
1574.7196	1573.7123	1573.8028	-0.0905	248 - 260	0	K.FASFETIVEQIYK.H
1707.7763	1706.7690	1706.8450	-0.0760	135 - 150	0	R.GWVPTLLGYSAQGACK.F + Carbamidomethyl (C)
2084.0093	2083.0020	2083.0812	-0.0792	333 - 351	0	R.IVMIGTLTGAQWGIYDAFK.V

No match to: 854.9449, 860.9786, 876.9413, 959.3966, 975.4483, 976.4208, 980.4021, 983.4562, 1065.9443, 1083.4282, 1104.4679, 1132.5159, 1196.4419, 1215.5637, 1218.5811, 1231.5737, 1235.5752, 1287.5711, 1297.5441, 1300.6026, 1319.5820, 1336.3908, 1352.5712, 1407.6373, 1427.4641, 1439.7198, 1443.6886, 1485.7681, 1499.7430, 1508.6825, 1558.7275, 1585.8063, 1634.7576, 1656.7520, 1805.8453, 1834.8681, 1927.9091, 1976.9265, 1993.9450, 2045.0624, 2067.6716, 2069.7862, 2193.1061, 2211.0997, 2362.2237, 2890.4720

46. Q5ZF44_MEDTR **Mass:** 39616 **Score:** 47 **Expect:** 66 **Queries matched:** 8

Mitochondrial phosphate translocator.- Medicago truncatula (Barrel medic).

Observed	Mr(expt)	Mr(calc)	Delta	Start - End	Miss	Peptide
1076.4495	1075.4422	1075.4790	-0.0368	104 - 112	0	K.CNMQIDPAK.Y + Carbamidomethyl (C)
1258.5780	1257.5707	1257.6942	-0.1235	201 - 211	1	K.VRVQTQPGFAR.G
1310.5953	1309.5880	1309.6158	-0.0278	104 - 114	1	K.CNMQIDPAKYK.S
1311.6184	1310.6111	1310.7234	-0.1122	220 - 231	1	K.FVKSEGALGLYK.G
1350.5619	1349.5546	1349.6761	-0.1215	52 - 64	1	R.GTGSGRFMIPSPK.E + Oxidation (M)
1574.7196	1573.7123	1573.8028	-0.0905	248 - 260	0	K.FASFETIVEQIYK.H
1707.7763	1706.7690	1706.8450	-0.0760	135 - 150	0	R.GWVPTLLGYSAQGACK.F + Carbamidomethyl (C)
2084.0093	2083.0020	2083.0812	-0.0792	333 - 351	0	R.IVMIGTLTGAQWGIYDAFK.V

No match to: 854.9449, 860.9786, 876.9413, 959.3966, 975.4483, 976.4208, 980.4021, 983.4562, 1065.9443, 1083.4282, 1104.4679, 1132.5159, 1196.4419, 1215.5637, 1218.5811, 1231.5737, 1235.5752, 1287.5711, 1297.5441, 1300.6026, 1319.5820, 1336.3908, 1352.5712, 1407.6373, 1427.4641, 1439.7198, 1443.6886, 1485.7681, 1499.7430, 1508.6825, 1558.7275, 1585.8063, 1634.7576, 1656.7520, 1805.8453, 1834.8681, 1927.9091, 1976.9265, 1993.9450, 2045.0624, 2067.6716, 2069.7862, 2193.1061, 2211.0997, 2362.2237, 2890.4720

47. Q5R9R8_PONPY **Mass:** 72095 **Score:** 47 **Expect:** 66 **Queries matched:** 12

Hypothetical protein DKFZp469P0412.- Pongo pygmaeus (Orangutan).

Observed	Mr(expt)	Mr(calc)	Delta	Start		End	Miss	Peptide
976.4208	975.4135	975.4985	-0.0850	56	-	63	1	K.ENRSLETK.Y
1297.5441	1296.5368	1296.6383	-0.1015	38	-	48	0	R.YMATVVENLNK.A + Oxidation (M)
1300.6026	1299.5954	1299.5942	0.0011	351	-	361	0	R.EPEQLGEDVER.R
1311.6184	1310.6111	1310.5383	0.0728	465	-	474	0	K.VYHMDMEAQR.K + 2 Oxidation (M)
1407.6373	1406.6300	1406.6434	-0.0134	465	-	475	1	K.VYHMDMEAQRK.N
1439.7198	1438.7125	1438.6333	0.0792	465	-	475	1	K.VYHMDMEAQRK.N + 2 Oxidation (M)
1585.8063	1584.7990	1584.7462	0.0528	580	-	592	1	R.RECLSSMVTMFPK.V + Carbamidomethyl (C)
1656.7520	1655.7447	1655.8413	-0.0966	66	-	79	1	R.HLQMQATKELSSQR.L
1834.8681	1833.8608	1833.9519	-0.0911	395	-	411	0	R.RPHLPMSPSTQQSALGK.Q
1976.9265	1975.9192	1975.9210	-0.0018	294	-	307	1	R.RQLWLEEEEMWQQR.Q + Oxidation (M)
2084.0093	2083.0020	2082.9453	0.0566	344	-	361	1	K.EQESPGREPEQLGEDVER.R
2193.1061	2192.0988	2192.0605	0.0383	529	-	546	1	R.QEVINHVQIMKEMEASYK.A + Oxidation (M)

No match to: 854.9449, 860.9786, 876.9413, 959.3966, 975.4483, 980.4021, 983.4562, 1065.9443, 1076.4495, 1083.4282, 1104.4679, 1132.5159, 1196.4419, 1215.5637, 1218.5811, 1231.5737, 1235.5752, 1258.5780, 1287.5711, 1310.5953, 1319.5820, 1336.3908, 1350.5619, 1352.5712, 1427.4641, 1443.6886, 1485.7681, 1499.7430, 1508.6825, 1558.7275, 1574.7196, 1634.7576, 1707.7763, 1805.8453, 1927.9091, 1993.9450, 2045.0624, 2067.6716, 2069.7862, 2211.0997, 2362.2237, 2890.4720

48. Q4CFZ5_CLOTM **Mass:** 11661 **Score:** 47 **Expect:** 68 **Queries matched:** 6

Hypothetical protein.- Clostridium thermocellum ATCC 27405.

Observed	Mr(expt)	Mr(calc)	Delta	Start		End	Miss	Peptide
1352.5712	1351.5639	1351.6805	-0.1166	41	-	51	0	K.VYNQDVVQLMK.R + Oxidation (M)
1508.6825	1507.6753	1507.7816	-0.1064	41	-	52	1	K.VYNQDVVQLMKR.L + Oxidation (M)
1558.7275	1557.7202	1557.8038	-0.0836	16	-	29	1	K.YAKSVLGSELYSNK.I
1707.7763	1706.7690	1706.8661	-0.0971	38	-	51	1	K.ENKVYNQDVVQLMK.R
1834.8681	1833.8608	1833.9988	-0.1380	19	-	34	1	K.SVLGSELYSNKIFIHK.I
1976.9265	1975.9192	1975.9239	-0.0047	68	-	84	0	R.CEIPPVDIVITMGCNMK.Y + 2 Carbamidomethyl (C)

No match to: 854.9449, 860.9786, 876.9413, 959.3966, 975.4483, 976.4208, 980.4021, 983.4562, 1065.9443, 1076.4495, 1083.4282, 1104.4679, 1132.5159, 1196.4419, 1215.5637, 1218.5811, 1231.5737, 1235.5752, 1258.5780, 1287.5711, 1297.5441, 1300.6026, 1310.5953, 1311.6184, 1319.5820, 1336.3908, 1350.5619, 1407.6373, 1427.4641, 1439.7198, 1443.6886, 1485.7681, 1499.7430, 1574.7196, 1585.8063, 1634.7576, 1656.7520, 1805.8453, 1927.9091, 1993.9450, 2045.0624, 2067.6716, 2069.7862, 2084.0093, 2193.1061, 2211.0997, 2362.2237, 2890.4720

49. Q2DM63_9DELT **Mass:** 19010 **Score:** 47 **Expect:** 69 **Queries matched:** 7

Hypothetical protein precursor.- Geobacter uraniumreducens Rf4.

Observed	Mr(expt)	Mr(calc)	Delta	Start		End	Miss	Peptide
975.4483	974.4410	974.4967	-0.0557	159	-	166	1	K.EMANKNIR.-
1132.5159	1131.5087	1131.5780	-0.0693	142	-	151	1	K.MQGCLPEVKK.L
1231.5737	1230.5665	1230.6464	-0.0799	140	-	150	1	R.VKMQGCLPEVK.K
1352.5712	1351.5639	1351.6111	-0.0472	152	-	163	1	K.LAGDMEKEMANK.N + Oxidation (M)
1485.7681	1484.7608	1484.7398	0.0210	93	-	105	0	K.DEVVAVYADLYTK.D
1499.7430	1498.7357	1498.8467	-0.1110	127	-	139	1	K.ASELNERLISVLR.V
2890.4720	2889.4647	2889.5098	-0.0451	2	-	27	1	M.KAAISGLLFLSVYLPSFCFASDLSQR.A + Carbamidomethyl (C)

No match to: 854.9449, 860.9786, 876.9413, 959.3966, 976.4208, 980.4021, 983.4562, 1065.9443, 1076.4495, 1083.4282, 1104.4679, 1196.4419, 1215.5637, 1218.5811, 1235.5752, 1258.5780, 1287.5711, 1297.5441, 1300.6026, 1310.5953, 1311.6184, 1319.5820, 1336.3908, 1350.5619, 1407.6373, 1427.4641, 1439.7198, 1443.6886, 1508.6825, 1558.7275, 1574.7196, 1585.8063, 1634.7576, 1656.7520, 1707.7763, 1805.8453, 1834.8681, 1927.9091, 1976.9265, 1993.9450, 2045.0624, 2067.6716, 2069.7862, 2084.0093, 2193.1061, 2211.0997, 2362.2237

50. Q7P987_RICSI **Mass:** 73246 **Score:** 47 **Expect:** 71 **Queries matched:** 10

Propionyl-CoA carboxylase alpha chain.- Rickettsia sibirica 246.

Observed	Mr(expt)	Mr(calc)	Delta	Start		End	Miss	Peptide
975.4483	974.4410	974.5073	-0.0663	500	-	507	0	R.WVVTIDDK.L
976.4208	975.4135	975.4668	-0.0533	163	-	173	1	K.AAAGGGGRGMR.V + Oxidation (M)
1104.4679	1103.4606	1103.5645	-0.1039	632	-	640	0	K.MENLILAER.D + Oxidation (M)
1235.5752	1234.5679	1234.6478	-0.0799	580	-	590	0	R.ISELEALMVSK.V + Oxidation (M)
1350.5619	1349.5546	1349.6074	-0.0528	287	-	296	0	K.NFYFLEMNTR.L + Oxidation (M)
1443.6886	1442.6813	1442.7477	-0.0664	482	-	495	0	R.ASLISGNINNQANK.I
1558.7275	1557.7202	1557.8514	-0.1312	359	-	372	1	R.IIAYSEPAKSPNIR.I
1707.7763	1706.7690	1706.7933	-0.0243	174	-	189	0	R.VVNNPAEMANAFESAK.L + Oxidation (M)
1976.9265	1975.9192	1975.9958	-0.0766	614	-	631	0	K.EGQEVTVGQEIMILTAMK.M
2890.4720	2889.4647	2889.5157	-0.0510	126	-	153	1	K.IAIEAGVSTVPGYMGTINDVKQAIDIAK.E + Oxidation (M)

No match to: 854.9449, 860.9786, 876.9413, 959.3966, 980.4021, 983.4562, 1065.9443, 1076.4495, 1083.4282, 1132.5159, 1196.4419, 1215.5637, 1218.5811, 1231.5737, 1258.5780, 1287.5711, 1297.5441, 1300.6026, 1310.5953, 1311.6184, 1319.5820, 1336.3908, 1352.5712, 1407.6373, 1427.4641, 1439.7198, 1485.7681, 1499.7430, 1508.6825, 1574.7196, 1585.8063, 1634.7576, 1656.7520, 1805.8453, 1834.8681, 1927.9091, 1993.9450, 2045.0624, 2067.6716, 2069.7862, 2084.0093, 2193.1061, 2211.0997, 2362.2237

Search Parameters

Type of search : Peptide Mass Fingerprint
Enzyme : Trypsin
Variable modifications : Carbamidomethyl (C),Oxidation (M)
Mass values : Monoisotopic
Protein Mass : Unrestricted
Peptide Mass Tolerance : ± 100 ppm
Peptide Charge State : 1+

```
Max Missed Cleavages   : 1
Number of queries      : 54
```

Mascot: http://www.matrixscience.com/
