# Supplementary material for: A proteomic strategy to identify novel serum biomarkers for liver cirrhosis and hepatocellular cancer in individuals with fatty liver disease
Source: BMC Cancer. 2009 Aug 5;9:271. doi: 10.1186/1471-2407-9-271 (PMC2729079; doi:10.1186/1471-2407-9-271)
Supplement: Additional File 10 — Spot 5 is CD5L. The protein summary report for spot 5, generated using Mascot Peptide Mass Fingerprint search program (Matrix Science Ltd), identifies it as CD5L. [file 1471-2407-9-271-S10.pdf]

Mascot Search Results

User : Joe Gray
Email : joe.gray@ncl.ac.uk
Search title : JG_2_0001.dat - SpecView
Database : MSDB 20060831 (3239079 sequences; 1079594700 residues)
Timestamp : 12 Jan 2007 at 12:20:49 GMT
Top Score : 218 for AAD01446, AF011429 NID: - Homo sapiens

Probability Based Mowse Score

Protein score is -10*Log(P), where P is the probability that the observed match is a random event.
Protein scores greater than 78 are significant (p<0.05).

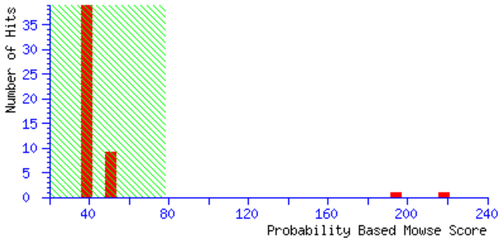

Protein Summary Report

Format As	Protein Summary	Help
	Significance threshold p< 0.05	Max. number of hits 50

Re-Search All | Search Unmatched

Index

	Accession	Mass	Score	Description
1.	AAD01446	38063	218	AF011429 NID: - Homo sapiens
2.	AAQ88858	38105	189	AY358494 NID: - Homo sapiens
3.	S39393	13733	53	glutathione transferase (EC 2.5.1.18) 3 - rat (fragments)
4.	MTBC2_METMA	22660	51	Dimethylamine corrinoid protein 2.- Methanosarcina mazei (Methanosarcina frisia).
5.	Q492F4_BLOPB	18561	51	Bacterioferritin comigratory protein.- Blochmannia pennsylvanicus (strain BPEN).
6.	MTBC1_METMA	22965	50	Dimethylamine corrinoid protein 1.- Methanosarcina mazei (Methanosarcina frisia).
7.	Q76CL0_9CNID	74138	48	FcEP-1 (Fragment).- Favites chinensis.
8.	Q5X4J4_LEGPA	64177	47	Hypothetical protein.- Legionella pneumophila (strain Paris).
9.	AAM31748	23736	47	AE008384 NID: - Methanosarcina mazei Go1
10.	Q7NU53_CHRVO	12921	46	Probable ferredoxin.- Chromobacterium violaceum.
11.	Q4P4T4_USTMA	15821	46	Hypothetical protein.- Ustilago maydis (Smut fungus).
12.	Q766Y7_PIG	12960	44	Calcitonin receptor-stimulating peptide-2.- Sus scrofa (Pig).
13.	Q2KKS8_9PERO	13440	44	CG11943-PB-like protein (Fragment).- Siniperca chuatsi (Chinese perch).
14.	AAM13420	103703	44	AF443596 NID: - Zea mays
15.	Q31EJ1_THICR	23625	44	Methionine biosynthesis MetW.- Thiomicrospira crunogena (strain XCL-2).
16.	C72150	15663	43	B3R protein - variola minor virus (strain Garcia-1966)
17.	T28439	15693	43	hypothetical protein D4R - variola major virus
18.	S30979	10815	43	gene 34 protein - Mycobacterium phage L5
19.	Q3F6Z7_9BURK	46446	43	Extracellular ligand-binding receptor.- Burkholderia ambifaria AMMD.
20.	Q2XLE6_PSEPU	48195	42	Toluene transport protein, putative precursor.- Pseudomonas putida F1.
21.	B36837	15761	42	D1L protein - variola virus (strain India-1967)
22.	AAU28929	78546	42	AE017354 NID: - Legionella pneumophila subsp. pneumophila str. Philadelphia 1
23.	Q6DIG7_XENTR	13411	42	Cyclin-dependent kinase inhibitor 2B (P15, inhibits CDK4).- Xenopus tropicalis (Western clawed frog) (Silurana tropicalis).
24.	Q1Z385_PHOPR	33824	42	Putative transcriptional activator protein NhaR.- Photobacterium profundum 3TCK.
25.	C96537	39973	42	hypothetical protein F2J10.5 [imported] - Arabidopsis thaliana

26.	Q5JMR0_ORYSA	22633	42	Sperm protein-like.- Oryza sativa (japonica cultivar-group).
27.	Q4DF81_TRYCR	73363	42	Hypothetical protein.- Trypanosoma cruzi.
28.	Q1VVY8_9FLAO	7992	42	Hypothetical protein.- Psychroflexus torquis ATCC 700755.
29.	Q8RET8_FUSNN	51567	41	Adenosylmethionine-8-amino-7-oxononanoate aminotransferase (EC 2.6.1.62).- Fusobacterium nucleatum subsp. nucleatum.
30.	Q24161_DROME	47840	41	CKII alpha subunit interactor 1.- Drosophila melanogaster (Fruit fly).
31.	CAH17038	75747	41	CR628337 NID: - Legionella pneumophila str. Lens
32.	Q9VUU3_DROME	47883	40	CG6215-PA (RE17810p) (RE16596p).- Drosophila melanogaster (Fruit fly).
33.	HLDD_PSEAE	37165	40	ADP-L-glycero-D-manno-heptose-6-epimerase (EC 5.1.3.20) (ADP-L- glycero-beta-D-manno-heptose-6-epimerase) (ADP-glyceromanno-heptose 6- epimerase) (ADP-hep 6-ep
34.	C83228	37394	40	ADP-L-glycero-D-mannoheptose 6-epimerase PA3337 [imported] - Pseudomonas aeruginosa (strain PAO1)
35.	Q8PZQ4_METMA	31906	40	Methylenetetrahydrofolate reductase (EC 1.5.1.20).- Methanosarcina mazei (Methanosarcina frisia).
36.	Q268R7_MYCVN	26517	40	Regulatory protein, TetR.- Mycobacterium vanbaalenii PYR-1.
37.	Q39SP8_GEOMG	5632	40	Hypothetical protein.- Geobacter metallireducens (strain GS-15 / ATCC 53774 / DSM 7210).
38.	PN0600	8559	40	DNA-directed RNA polymerase (EC 2.7.7.6) III - Pneumocystis carinii (fragment)
39.	Q8NRN6_CORGL	10266	39	Hypothetical protein Cgl1012 (Hypothetical protein).- Corynebacterium glutamicum (Brevibacterium flavum).
40.	Q3MHI8_BOVIN	39549	39	Hypothetical protein MGC128771.- Bos taurus (Bovine).
41.	BAB22477	30402	39	AK002954 NID: - Mus musculus
42.	Q3ISL2_NATPD	6952	39	Hypothetical protein.- Natronomonas pharaonis (strain DSM 2160 / ATCC 35678).
43.	BAD13409	14179	39	AB121202 NID: - Macrothele gigas
44.	Q963V7_9DIPT	17429	39	Reverse transcriptase (Fragment).- Chironomus agilis.
45.	Q4XGI8_PLACH	3067	39	Hypothetical protein (Fragment).- Plasmodium chabaudi.
46.	Q9JI50_MOUSE	27603	39	Dendritic cell-associated C-type lectin-1.- Mus musculus (Mouse).
47.	Q8GXA4_ARATH	53868	39	Hypothetical protein At4g26450/M3E9_120 (At4g26450).- Arabidopsis thaliana (Mouse-ear cress).
48.	Q9D0J3_MOUSE	12279	39	10 days embryo whole body cDNA, RIKEN full-length enriched library, clone:2610010G17 product:GRAF homolog.- Mus musculus (Mouse).
49.	Q4DDU3_TRYCR	29352	38	Mucin-associated surface protein (MASP), putative.- Trypanosoma cruzi.
50.	T43206	43182	38	probable aminopeptidase (EC 3.4.11.-) - fission yeast (Schizosaccharomyces pombe)

Results List

1. AAD01446 **Mass:** 38063 **Score:** 218 **Expect:** 5.1e-16 **Queries matched:** 26

AF011429 NID: - Homo sapiens

Observed	Mr(expt)	Mr(calc)	Delta	Start	End	Miss	Peptide
751.3859	750.3787	750.4500	-0.0714	26 -	32	0	R.LVGGLHR.C
793.3455	792.3382	792.4130	-0.0748	290 -	296	0	K.SLSPSFR.D
865.3301	864.3228	864.3912	-0.0683	300 -	307	0	K.CYGPGVGR.I + Carbamidomethyl (C)
915.4276	914.4203	914.4974	-0.0771	308 -	314	0	R.IWLDNVR.C
993.4277	992.4205	992.4861	-0.0657	299 -	307	1	R.KCYGPGVGR.I + Carbamidomethyl (C)
1005.3922	1004.3850	1004.4610	-0.0760	191 -	198	1	R.CNKHAYGR.K + Carbamidomethyl (C)
1064.4451	1063.4378	1063.5410	-0.1032	290 -	298	1	K.SLSPSFRDR.K
1167.4829	1166.4756	1166.5614	-0.0858	140 -	150	1	R.LADGPGHCKGR.V + Carbamidomethyl (C)
1545.5785	1544.5712	1544.6929	-0.1217	212 -	225	0	R.EATLQDCPSGPWGK.N + Carbamidomethyl (C)
1549.6700	1548.6627	1548.7541	-0.0913	199 -	211	0	R.KPIWLSQMSCSGR.E + Carbamidomethyl (C)
1565.6430	1564.6358	1564.7490	-0.1132	199 -	211	0	R.KPIWLSQMSCSGR.E + Carbamidomethyl (C); Oxidation (M)
1747.6291	1746.6218	1746.7050	-0.0831	315 -	328	0	R.CSGEEQSLEQCQHR.F + 2 Carbamidomethyl (C)
1761.7506	1760.7434	1760.8780	-0.1346	300 -	314	1	K.CYGPGVGRIWLDNVR.C + Carbamidomethyl (C)
1866.8770	1865.8697	1865.9781	-0.1084	246 -	262	1	R.LVGGDNLCSGRLEVLHK.G + Carbamidomethyl (C)
2063.8573	2062.8500	2062.9432	-0.0931	155 -	170	0	K.HQNQWYTVCQTGWSLR.A + Carbamidomethyl (C)
2133.9402	2132.9329	2133.0360	-0.1030	194 -	211	1	K.HAYGRKPIWLSQMSCSGR.E + Carbamidomethyl (C)
2149.9197	2148.9125	2149.0309	-0.1184	194 -	211	1	K.HAYGRKPIWLSQMSCSGR.E + Carbamidomethyl (C); Oxidation (M)
2264.8464	2263.8392	2263.9415	-0.1023	329 -	347	0	R.FWGFHDCTHQEDVAVICSG.- + 2 Carbamidomethyl (C)
2334.0437	2333.0364	2333.1123	-0.0759	155 -	173	1	K.HQNQWYTVCQTGWSLRAAK.V + Carbamidomethyl (C)
2450.0481	2449.0408	2449.1154	-0.0746	43 -	63	1	K.GQWGTVCDDGWDIKDVAVLCR.E + 2 Carbamidomethyl (C)
2466.9528	2465.9455	2466.0579	-0.1124	263 -	283	1	K.GVWGSVCDDNWGEKEDQVVCK.Q + 2 Carbamidomethyl (C)
2519.1418	2518.1345	2518.2175	-0.0830	151 -	170	1	R.VEVKHQNQWYTVCQTGWSLR.A + Carbamidomethyl (C)
2551.9627	2550.9554	2551.0016	-0.0462	226 -	245	0	K.NTCNHDEDTWVECEDPFDLR.L + 2 Carbamidomethyl (C)
2589.1406	2588.1334	2588.2427	-0.1094	64 -	88	1	R.ELGCGAASGTPSGILYEPPAEKEQK.V + Carbamidomethyl (C)
2644.0843	2643.0770	2643.1918	-0.1147	308 -	328	1	R.IWLDNVRCSGEEQSLEQCQHR.F + 2 Carbamidomethyl (C)
3993.6333	3992.6260	3992.6359	-0.0099	315 -	347	1	R.CSGEEQSLEQCQHRFWGFHDCTHQEDVAVICSG.- + 4 Carbamidomethyl (C)

No match to: 832.2988, 848.3093, 861.0016, 876.9628, 892.9507, 988.3750, 1216.4928, 1264.5354, 1501.7024, 1622.7394, 1729.6508, 1730.5932, 1744.7499, 1832.7851, 1848.8553, 1889.8854, 2019.7869, 2046.8381, 2062.8302, 2079.8709, 2095.8419, 2117.7673, 2210.9927, 2535.1542, 2611.1242, 2717.0583, 2732.2341, 2789.3006, 3110.3047, 3163.4230, 3647.6063, 3664.6062, 3937.5706, 3976.6005

2. AAQ88858 **Mass:** 38105 **Score:** 189 **Expect:** 4.1e-13 **Queries matched:** 24

AY358494 NID: - Homo sapiens

Observed	Mr(expt)	Mr(calc)	Delta	Start	End	Miss	Peptide
751.3859	750.3787	750.4500	-0.0714	26 -	32	0	R.LVGGLHR.C
793.3455	792.3382	792.4130	-0.0748	290 -	296	0	K.SLSPSFR.D
865.3301	864.3228	864.3912	-0.0683	300 -	307	0	K.CYGPGVGR.I + Carbamidomethyl (C)
915.4276	914.4203	914.4974	-0.0771	308 -	314	0	R.IWLDNVR.C

993.4277	992.4205	992.4861	-0.0657	299	- 307	1	R.KCYGPGVGR.I + Carbamidomethyl (C)
1005.3922	1004.3850	1004.4610	-0.0760	191	- 198	1	R.CNKHAYGR.K + Carbamidomethyl (C)
1064.4451	1063.4378	1063.5410	-0.1032	290	- 298	1	K.SLSPSFRDR.K
1167.4829	1166.4756	1166.5614	-0.0858	140	- 150	1	R.LADGPGHCKGR.V + Carbamidomethyl (C)
1545.5785	1544.5712	1544.6929	-0.1217	212	- 225	0	R.EATLQDCPSGPWGK.N + Carbamidomethyl (C)
1549.6700	1548.6627	1548.7541	-0.0913	199	- 211	0	R.KPIWLSQMSCSGR.E + Carbamidomethyl (C)
1565.6430	1564.6358	1564.7490	-0.1132	199	- 211	0	R.KPIWLSQMSCSGR.E + Carbamidomethyl (C); Oxidation (M)
1747.6291	1746.6218	1746.7050	-0.0831	315	- 328	0	R.CSGEEQSLEQCQHR.F + 2 Carbamidomethyl (C)
1761.7506	1760.7434	1760.8780	-0.1346	300	- 314	1	K.CYGPGVGRIWLDNVR.C + Carbamidomethyl (C)
1866.8770	1865.8697	1865.9781	-0.1084	246	- 262	1	R.LVGGDNLCSGRLEVLHK.G + Carbamidomethyl (C)
2063.8573	2062.8500	2062.9432	-0.0931	155	- 170	0	K.HQNQWYTVCQTGWSLR.A + Carbamidomethyl (C)
2133.9402	2132.9329	2133.0360	-0.1030	194	- 211	1	K.HAYGRKPIWLSQMSCSGR.E + Carbamidomethyl (C)
2149.9197	2148.9125	2149.0309	-0.1184	194	- 211	1	K.HAYGRKPIWLSQMSCSGR.E + Carbamidomethyl (C); Oxidation (M)
2334.0437	2333.0364	2333.1123	-0.0759	155	- 173	1	K.HQNQWYTVCQTGWSLRAAK.V + Carbamidomethyl (C)
2450.0481	2449.0408	2449.1154	-0.0746	43	- 63	1	K.GQWGTVCDDGWDIKDVAVLCR.E + 2 Carbamidomethyl (C)
2466.9528	2465.9455	2466.0579	-0.1124	263	- 283	1	K.GVWGSVCDDNWGEKEDQVVCK.Q + 2 Carbamidomethyl (C)
2519.1418	2518.1345	2518.2175	-0.0830	151	- 170	1	R.VEVKHQNQWYTVCQTGWSLR.A + Carbamidomethyl (C)
2551.9627	2550.9554	2551.0016	-0.0462	226	- 245	0	K.NTCNHDEDTWVECEDPFDLR.L + 2 Carbamidomethyl (C)
2589.1406	2588.1334	2588.2427	-0.1094	64	- 88	1	R.ELGCGAASGTPSGILYEPPAEKEQK.V + Carbamidomethyl (C)
2644.0843	2643.0770	2643.1918	-0.1147	308	- 328	1	R.IWLDNVRCSGEEQSLEQCQHR.F + 2 Carbamidomethyl (C)

No match to: 832.2988, 848.3093, 861.0016, 876.9628, 892.9507, 988.3750, 1216.4928, 1264.5354, 1501.7024, 1622.7394, 1729.6508, 1730.5932, 1744.7499, 1832.7851, 1848.8553, 1889.8854, 2019.7869, 2046.8381, 2062.8302, 2079.8709, 2095.8419, 2117.7673, 2210.9927, 2264.8464, 2535.1542, 2611.1242, 2717.0583, 2732.2341, 2789.3006, 3110.3047, 3163.4230, 3647.6063, 3664.6062, 3937.5706, 3976.6005, 3993.6333

3. S39393 **Mass:** 13733 **Score:** 53 **Expect:** 16 **Queries matched:** 7

glutathione transferase (EC 2.5.1.18) 3 - rat (fragments)

Observed	Mr(expt)	Mr(calc)	Delta	Start	End	Miss	Peptide
1264.5354	1263.5281	1263.6434	-0.1153	1	- 10	0	-.PMILGYWNVR.Y + Oxidation (M)
1622.7394	1621.7321	1621.8286	-0.0965	102	- 115	1	K.CLDAFPNLKDFLAR.-
1744.7499	1743.7427	1743.8034	-0.0607	57	- 70	0	R.MQLIMLCYNPDFEK.Q
2519.1418	2518.1345	2518.1409	-0.0064	1	- 21	1	-.PMILGYWNVRYAMGDAPDYDR.S + Oxidation (M)
2535.1542	2534.1469	2534.1358	0.0111	1	- 21	1	-.PMILGYWNVRYAMGDAPDYDR.S + 2 Oxidation (M)
2589.1406	2588.1334	2588.2838	-0.1505	81	- 101	0	K.VTYVDFLAYDILDQYHIFEPK.C
3647.6063	3646.5990	3646.8057	-0.2067	81	- 110	1	K.VTYVDFLAYDILDQYHIFEPKCLDAFPNLK.D + Carbamidomethyl (C)

No match to: 751.3859, 793.3455, 832.2988, 848.3093, 861.0016, 865.3301, 876.9628, 892.9507, 915.4276, 988.3750, 993.4277, 1005.3922, 1064.4451, 1167.4829, 1216.4928, 1501.7024, 1545.5785, 1549.6700, 1565.6430, 1729.6508, 1730.5932, 1747.6291, 1761.7506, 1832.7851, 1848.8553, 1866.8770, 1889.8854, 2019.7869, 2046.8381, 2062.8302, 2063.8573, 2079.8709, 2095.8419, 2117.7673, 2133.9402, 2149.9197, 2210.9927, 2264.8464, 2334.0437, 2450.0481, 2466.9528, 2551.9627, 2611.1242, 2644.0843, 2717.0583, 2732.2341, 2789.3006, 3110.3047, 3163.4230, 3664.6062, 3937.5706, 3976.6005, 3993.6333

4. MTBC2_METMA **Mass:** 22660 **Score:** 51 **Expect:** 27 **Queries matched:** 8

Dimethylamine corrinoid protein 2.- Methanosarcina mazei (Methanosarcina frisia).

Observed	Mr(expt)	Mr(calc)	Delta	Start	End	Miss	Peptide
915.4276	914.4203	914.5072	-0.0869	21	- 29	0	K.DAVLAAVEK.A
1501.7024	1500.6952	1500.7394	-0.0443	178	- 192	0	K.VMVGGAPATQAWADK.I
1744.7499	1743.7427	1743.8977	-0.1551	176	- 192	1	R.VKVMVGGAPATQAWADK.I + Oxidation (M)
2046.8381	2045.8308	2046.0204	-0.1896	110	- 128	0	K.SIVSTMLQSAGFEVHDIGR.D
2062.8302	2061.8229	2062.0153	-0.1924	110	- 128	0	K.SIVSTMLQSAGFEVHDIGR.D + Oxidation (M)
2063.8573	2062.8500	2063.0456	-0.1955	2	- 19	1	M.ATKEELIQELSDSIISCK.K + Carbamidomethyl (C)
2210.9927	2209.9855	2210.0810	-0.0955	1	- 19	1	-.MATKEELIQELSDSIISCK.K + Carbamidomethyl (C); Oxidation (M)
3110.3047	3109.2975	3109.4484	-0.1509	178	- 208	1	K.VMVGGAPATQAWADKIGADCYAENASEAVAK.A + Oxidation (M)

No match to: 751.3859, 793.3455, 832.2988, 848.3093, 861.0016, 865.3301, 876.9628, 892.9507, 988.3750, 993.4277, 1005.3922, 1064.4451, 1167.4829, 1216.4928, 1264.5354, 1545.5785, 1549.6700, 1565.6430, 1622.7394, 1729.6508, 1730.5932, 1747.6291, 1761.7506, 1832.7851, 1848.8553, 1866.8770, 1889.8854, 2019.7869, 2079.8709, 2095.8419, 2117.7673, 2133.9402, 2149.9197, 2264.8464, 2334.0437, 2450.0481, 2466.9528, 2519.1418, 2535.1542, 2551.9627, 2589.1406, 2611.1242, 2644.0843, 2717.0583, 2732.2341, 2789.3006, 3163.4230, 3647.6063, 3664.6062, 3937.5706, 3976.6005, 3993.6333

5. Q492F4_BLOPB **Mass:** 18561 **Score:** 51 **Expect:** 28 **Queries matched:** 9

Bacterioferritin comigratory protein.- Blochmannia pennsylvanicus (strain BPEN).

Observed	Mr(expt)	Mr(calc)	Delta	Start	End	Miss	Peptide
751.3859	750.3787	750.4276	-0.0489	76	- 81	1	K.SEKLFK.F
1167.4829	1166.4756	1166.5502	-0.0746	54	- 62	1	K.LRDNMDTFR.K
1264.5354	1263.5281	1263.6109	-0.0828	152	- 161	0	R.YLHDTHVSHR.-
1549.6700	1548.6627	1548.7574	-0.0947	42	- 55	1	K.AMTPGCTIQACKLR.D + Carbamidomethyl (C)
1565.6430	1564.6358	1564.7524	-0.1166	42	- 55	1	K.AMTPGCTIQACKLR.D + Carbamidomethyl (C); Oxidation (M)
1622.7394	1621.7321	1621.7738	-0.0418	42	- 55	1	K.AMTPGCTIQACKLR.D + 2 Carbamidomethyl (C); Oxidation (M)
2133.9402	2132.9329	2132.9758	-0.0429	86	- 102	0	K.EMLNFTLLYDENCQISK.K + Carbamidomethyl (C); Oxidation (M)
2334.0437	2333.0364	2333.1622	-0.1257	34	- 53	1	K.ILIYFYPKAMTPGCTIQACK.L + Carbamidomethyl (C); Oxidation (M)
2551.9627	2550.9554	2551.1974	-0.2420	82	- 102	1	K.FSEKEMLNFTLLYDENCQISK.K

No match to: 793.3455, 832.2988, 848.3093, 861.0016, 865.3301, 876.9628, 892.9507, 915.4276, 988.3750, 993.4277, 1005.3922, 1064.4451, 1216.4928, 1501.7024, 1545.5785, 1729.6508, 1730.5932, 1744.7499, 1747.6291, 1761.7506, 1832.7851, 1848.8553, 1866.8770, 1889.8854, 2019.7869, 2046.8381, 2062.8302, 2063.8573, 2079.8709, 2095.8419, 2117.7673, 2149.9197, 2210.9927, 2264.8464, 2450.0481, 2466.9528, 2519.1418, 2535.1542, 2589.1406, 2611.1242, 2644.0843, 2717.0583, 2732.2341, 2789.3006, 3110.3047, 3163.4230, 3647.6063, 3664.6062, 3937.5706, 3976.6005, 3993.6333

6. MTBC1_METMA **Mass:** 22965 **Score:** 50 **Expect:** 33 **Queries matched:** 9

Dimethylamine corrinoid protein 1.- Methanosarcina mazei (Methanosarcina frisia).

Observed	Mr(expt)	Mr(calc)	Delta	Start	End	Miss	Peptide
915.4276	914.4203	914.5072	-0.0869	21 -	29	0	K.DAVLAAVEK.A
1501.7024	1500.6952	1500.7394	-0.0443	178 -	192	0	K.VMVGGAPATQAWADK.I
1744.7499	1743.7427	1743.8977	-0.1551	176 -	192	1	K.VKVMVGGAPATQAWADK.I + Oxidation (M)
1761.7506	1760.7434	1760.8866	-0.1432	5 -	19	0	K.EELLQELSEAIISCK.K + Carbamidomethyl (C)
1832.7851	1831.7778	1831.9601	-0.1823	5 -	20	1	K.EELLQELSEAIISCKK.D
1889.8854	1888.8781	1888.9815	-0.1034	5 -	20	1	K.EELLQELSEAIISCKK.D + Carbamidomethyl (C)
2046.8381	2045.8308	2046.0204	-0.1896	110 -	128	0	K.SIVSTMLQSAGFEVHDIGR.D
2062.8302	2061.8229	2062.0153	-0.1924	110 -	128	0	K.SIVSTMLQSAGFEVHDIGR.D + Oxidation (M)
3110.3047	3109.2975	3109.4484	-0.1509	178 -	208	1	K.VMVGGAPATQAWADKIGADCYAENASEAVAK.A + Oxidation (M)

No match to: 751.3859, 793.3455, 832.2988, 848.3093, 861.0016, 865.3301, 876.9628, 892.9507, 988.3750, 993.4277, 1005.3922, 1064.4451, 1167.4829, 1216.4928, 1264.5354, 1545.5785, 1549.6700, 1565.6430, 1622.7394, 1729.6508, 1730.5932, 1747.6291, 1848.8553, 1866.8770, 2019.7869, 2063.8573, 2079.8709, 2095.8419, 2117.7673, 2133.9402, 2149.9197, 2210.9927, 2264.8464, 2334.0437, 2450.0481, 2466.9528, 2519.1418, 2535.1542, 2551.9627, 2589.1406, 2611.1242, 2644.0843, 2717.0583, 2732.2341, 2789.3006, 3163.4230, 3647.6063, 3664.6062, 3937.5706, 3976.6005, 3993.6333

7. Q76CL0_9CNID **Mass:** 74138 **Score:** 48 **Expect:** 51 **Queries matched:** 12

FcEP-1 (Fragment).- Favites chinensis.

Observed	Mr(expt)	Mr(calc)	Delta	Start	End	Miss	Peptide
793.3455	792.3382	792.3800	-0.0417	571 -	578	0	K.QGGCTVTK.I
1167.4829	1166.4756	1166.5542	-0.0787	646 -	654	1	K.FIQSWQQKC.-
1549.6700	1548.6627	1548.6952	-0.0325	21 -	32	1	K.MFGDEVKYMEIR.Q + 2 Oxidation (M)
1729.6508	1728.6436	1728.8042	-0.1606	501 -	516	0	K.GGGCPYVLVQDHWGGK.E + Carbamidomethyl (C)
2019.7869	2018.7796	2018.9189	-0.1393	209 -	225	1	K.NPENCKITHEQVAMDFK.R + Oxidation (M)
2095.8419	2094.8347	2095.0255	-0.1909	471 -	488	1	R.EIKNSLYAQCEVQDQTVK.V
2117.7673	2116.7600	2116.9570	-0.1970	501 -	519	1	K.GGGCPYVLVQDHWGGKECR.I + Carbamidomethyl (C)
2210.9927	2209.9855	2210.0025	-0.0171	331 -	350	1	R.QMVIQMQGTKAPGCEDCMLK.C
2644.0843	2643.0770	2643.1371	-0.0601	341 -	364	1	K.APGCEDCMLKCTANPQGMTLQFGR.G + Carbamidomethyl (C); Oxidation (M)
2717.0583	2716.0510	2716.1535	-0.1025	341 -	364	1	K.APGCEDCMLKCTANPQGMTLQFGR.G + 2 Carbamidomethyl (C); 2 Oxidation (M)
3110.3047	3109.2975	3109.5349	-0.2375	538 -	565	1	R.IQSSQESVVINPDQTILIDGHKADCSQK.A + Carbamidomethyl (C)
3647.6063	3646.5990	3646.8776	-0.2786	111 -	144	1	K.ANLNVEPSITSLLKFQTLTGSAEITPCIGAHEHR.Q

No match to: 751.3859, 832.2988, 848.3093, 861.0016, 865.3301, 876.9628, 892.9507, 915.4276, 988.3750, 993.4277, 1005.3922, 1064.4451, 1216.4928, 1264.5354, 1501.7024, 1545.5785, 1565.6430, 1622.7394, 1730.5932, 1744.7499, 1747.6291, 1761.7506, 1832.7851, 1848.8553, 1866.8770, 1889.8854, 2046.8381, 2062.8302, 2063.8573, 2079.8709, 2133.9402, 2149.9197, 2264.8464, 2334.0437, 2450.0481, 2466.9528, 2519.1418, 2535.1542, 2551.9627, 2589.1406, 2611.1242, 2732.2341, 2789.3006, 3163.4230, 3664.6062, 3937.5706, 3976.6005, 3993.6333

8. Q5X4J4_LEGPA **Mass:** 64177 **Score:** 47 **Expect:** 59 **Queries matched:** 11

Hypothetical protein.- Legionella pneumophila (strain Paris).

Observed	Mr(expt)	Mr(calc)	Delta	Start	End	Miss	Peptide
865.3301	864.3228	864.3898	-0.0670	482 -	488	0	R.EALEEMK.M + Oxidation (M)
1005.3922	1004.3850	1004.4451	-0.0601	35 -	42	0	R.ETSNYYTK.E
1264.5354	1263.5281	1263.6202	-0.0921	416 -	426	0	K.VVEVMNALMDK.Y + Oxidation (M)
1729.6508	1728.6436	1728.7746	-0.1310	427 -	440	1	K.YPQRSTTEMVACMR.I + Carbamidomethyl (C)
1747.6291	1746.6218	1746.7849	-0.1631	523 -	536	1	K.EYSTKNFHSQESYK.V
1761.7506	1760.7434	1760.7644	-0.0210	427 -	440	1	K.YPQRSTTEMVACMR.I + Carbamidomethyl (C); 2 Oxidation (M)
2019.7869	2018.7796	2018.9229	-0.1433	244 -	259	1	K.YNCNFEFEVNKLQPMK.F + Oxidation (M)
2644.0843	2643.0770	2643.2638	-0.1868	5 -	28	0	R.YSGFDYDGCVGVTNLINIVAQNPK.L + Carbamidomethyl (C)
2789.3006	2788.2933	2788.3312	-0.0379	528 -	551	1	K.NFHSQESYKVVGAYLCMQSQLTQK.L
3163.4230	3162.4157	3162.4902	-0.0745	312 -	338	0	K.AQQWNVLCDEGQWNNPIFTLQAIMAEK.S + Oxidation (M)
3976.6005	3975.5933	3975.9022	-0.3089	76 -	113	0	K.LCTDLGMEFDPFLLADAYNLVPAGTSLHEATNPANGAK.I

No match to: 751.3859, 793.3455, 832.2988, 848.3093, 861.0016, 876.9628, 892.9507, 915.4276, 988.3750, 993.4277, 1064.4451, 1167.4829, 1216.4928, 1501.7024, 1545.5785, 1549.6700, 1565.6430, 1622.7394, 1730.5932, 1744.7499, 1832.7851, 1848.8553, 1866.8770, 1889.8854, 2046.8381, 2062.8302, 2063.8573, 2079.8709, 2095.8419, 2117.7673, 2133.9402, 2149.9197, 2210.9927, 2264.8464, 2334.0437, 2450.0481, 2466.9528, 2519.1418, 2535.1542, 2551.9627, 2589.1406, 2611.1242, 2717.0583, 2732.2341, 3110.3047, 3647.6063, 3664.6062, 3937.5706, 3993.6333

9. AAM31748 **Mass:** 23736 **Score:** 47 **Expect:** 62 **Queries matched:** 9

AE008384 NID: - Methanosarcina mazei Go1

Observed	Mr(expt)	Mr(calc)	Delta	Start	End	Miss	Peptide
915.4276	914.4203	914.5072	-0.0869	27 -	35	0	K.DAVLAAVEK.A
1501.7024	1500.6952	1500.7394	-0.0443	184 -	198	0	K.VMVGGAPATQAWADK.I
1744.7499	1743.7427	1743.8977	-0.1551	182 -	198	1	K.VKVMVGGAPATQAWADK.I + Oxidation (M)
1761.7506	1760.7434	1760.8866	-0.1432	11 -	25	0	K.EELLQELSEAIISCK.K + Carbamidomethyl (C)
1832.7851	1831.7778	1831.9601	-0.1823	11 -	26	1	K.EELLQELSEAIISCKK.D
1889.8854	1888.8781	1888.9815	-0.1034	11 -	26	1	K.EELLQELSEAIISCKK.D + Carbamidomethyl (C)
2046.8381	2045.8308	2046.0204	-0.1896	116 -	134	0	K.SIVSTMLQSAGFEVHDIGR.D
2062.8302	2061.8229	2062.0153	-0.1924	116 -	134	0	K.SIVSTMLQSAGFEVHDIGR.D + Oxidation (M)
3110.3047	3109.2975	3109.4484	-0.1509	184 -	214	1	K.VMVGGAPATQAWADKIGADCYAENASEAVAK.A + Oxidation (M)

No match to: 751.3859, 793.3455, 832.2988, 848.3093, 861.0016, 865.3301, 876.9628, 892.9507, 988.3750, 993.4277, 1005.3922, 1064.4451, 1167.4829, 1216.4928, 1264.5354, 1545.5785, 1549.6700, 1565.6430, 1622.7394, 1729.6508, 1730.5932, 1747.6291, 1848.8553, 1866.8770, 2019.7869, 2063.8573, 2079.8709, 2095.8419, 2117.7673, 2133.9402, 2149.9197, 2210.9927, 2264.8464, 2334.0437, 2450.0481, 2466.9528, 2519.1418, 2535.1542, 2551.9627, 2589.1406, 2611.1242, 2644.0843, 2717.0583, 2732.2341, 2789.3006, 3163.4230, 3647.6063, 3664.6062, 3937.5706, 3976.6005, 3993.6333

10. Q7NU53_CHRVO **Mass:** 12921 **Score:** 46 **Expect:** 73 **Queries matched:** 6

Probable ferredoxin.- Chromobacterium violaceum.

Observed	Mr(expt)	Mr(calc)	Delta	Start	End	Miss	Peptide
1761.7506	1760.7434	1760.6487	0.0947	8 -	22	0	K.SNPCQSCGACCASFR.V + 4 Carbamidomethyl (C)
2046.8381	2045.8308	2045.9774	-0.1466	67 -	86	0	R.GEVGGSVGCGVYPLRPSPCR.E + Carbamidomethyl (C)
2551.9627	2550.9554	2551.0204	-0.0650	1 -	22	1	-.MLAMSEKSNPCQSCGACCASFR.V + 4 Carbamidomethyl (C)
2589.1406	2588.1334	2588.2773	-0.1439	62 -	86	1	R.CVALRGEVGGSVGCGVYPLRPSPCR.E + Carbamidomethyl (C)
2611.1242	2610.1169	2610.3553	-0.2384	98 -	123	1	K.ARASHGLPALWEAPVAANEAEPLLAG.-
3976.6005	3975.5933	3975.7072	-0.1139	8 -	44	1	K.SNPCQSCGACCASFRVSFYWAEADDGGGLVPGHLTEK.L + 2 Carbamidomethyl (C)

No match to: 751.3859, 793.3455, 832.2988, 848.3093, 861.0016, 865.3301, 876.9628, 892.9507, 915.4276, 988.3750, 993.4277, 1005.3922, 1064.4451, 1167.4829, 1216.4928, 1264.5354, 1501.7024, 1545.5785, 1549.6700, 1565.6430, 1622.7394, 1729.6508, 1730.5932, 1744.7499, 1747.6291, 1832.7851, 1848.8553, 1866.8770, 1889.8854, 2019.7869, 2062.8302, 2063.8573, 2079.8709, 2095.8419, 2117.7673, 2133.9402, 2149.9197, 2210.9927, 2264.8464, 2334.0437, 2450.0481, 2466.9528, 2519.1418, 2535.1542, 2644.0843, 2717.0583, 2732.2341, 2789.3006, 3110.3047, 3163.4230, 3647.6063, 3664.6062, 3937.5706, 3993.6333

11. Q4P4T4_USTMA **Mass:** 15821 **Score:** 46 **Expect:** 87 **Queries matched:** 6

Hypothetical protein.- Ustilago maydis (Smut fungus).

Observed	Mr(expt)	Mr(calc)	Delta	Start	End	Miss	Peptide
1622.7394	1621.7321	1621.8060	-0.0739	57 -	70	0	R.SNNQVYDNLSIVTR.S
2210.9927	2209.9855	2210.1906	-0.2051	85 -	105	1	R.QLVNTAAALGDDKKPQEATIK.E
2264.8464	2263.8392	2263.9772	-0.1380	106 -	124	1	K.EKCPGCGNDEMNFHTLQLR.S + Carbamidomethyl (C); Oxidation (M)
2551.9627	2550.9554	2551.2008	-0.2454	8 -	31	0	K.IGSLLFCPNCGSLLDVPGDEDMIK.C + Oxidation (M)
2589.1406	2588.1334	2588.3050	-0.1716	32 -	56	1	K.CAPCGAVQNAKGSADLLSTLLISSR.S + 2 Carbamidomethyl (C)
3163.4230	3162.4157	3162.5286	-0.1129	2 -	31	1	M.SALPDKIGSLLFCPNCGSLLDVPGDEDMIK.C + Oxidation (M)

No match to: 751.3859, 793.3455, 832.2988, 848.3093, 861.0016, 865.3301, 876.9628, 892.9507, 915.4276, 988.3750, 993.4277, 1005.3922, 1064.4451, 1167.4829, 1216.4928, 1264.5354, 1501.7024, 1545.5785, 1549.6700, 1565.6430, 1729.6508, 1730.5932, 1744.7499, 1747.6291, 1761.7506, 1832.7851, 1848.8553, 1866.8770, 1889.8854, 2019.7869, 2046.8381, 2062.8302, 2063.8573, 2079.8709, 2095.8419, 2117.7673, 2133.9402, 2149.9197, 2334.0437, 2450.0481, 2466.9528, 2519.1418, 2535.1542, 2611.1242, 2644.0843, 2717.0583, 2732.2341, 2789.3006, 3110.3047, 3647.6063, 3664.6062, 3937.5706, 3976.6005, 3993.6333

12. Q766Y7_PIG **Mass:** 12960 **Score:** 44 **Expect:** 1.4e+02 **Queries matched:** 6

Calcitonin receptor-stimulating peptide-2.- Sus scrofa (Pig).

Observed	Mr(expt)	Mr(calc)	Delta	Start	End	Miss	Peptide
1264.5354	1263.5281	1263.5336	-0.0055	80 -	90	0	K.SCNTASCVTHK.M + 2 Carbamidomethyl (C)
1549.6700	1548.6627	1548.7606	-0.0978	104 -	117	1	K.NNFMPTNVDSKILG.-
1565.6430	1564.6358	1564.7555	-0.1197	104 -	117	1	K.NNFMPTNVDSKILG.- + Oxidation (M)
2095.8419	2094.8347	2094.9397	-0.1051	80 -	97	1	K.SCNTASCVTHKMTGWLSR.S + 2 Carbamidomethyl (C)
2732.2341	2731.2268	2731.4923	-0.2655	6 -	29	0	K.FPPFLVLSILVLYQAGMFHTAPVR.L + Oxidation (M)
2789.3006	2788.2933	2788.3112	-0.0179	56 -	79	1	K.DYVQMKATVLEQESEDFSITAQEK.S

No match to: 751.3859, 793.3455, 832.2988, 848.3093, 861.0016, 865.3301, 876.9628, 892.9507, 915.4276, 988.3750, 993.4277, 1005.3922, 1064.4451, 1167.4829, 1216.4928, 1501.7024, 1545.5785, 1622.7394, 1729.6508, 1730.5932, 1744.7499, 1747.6291, 1761.7506, 1832.7851, 1848.8553, 1866.8770, 1889.8854, 2019.7869, 2046.8381, 2062.8302, 2063.8573, 2079.8709, 2117.7673, 2133.9402, 2149.9197, 2210.9927, 2264.8464, 2334.0437, 2450.0481, 2466.9528, 2519.1418, 2535.1542, 2551.9627, 2589.1406, 2611.1242, 2644.0843, 2717.0583, 3110.3047, 3163.4230, 3647.6063, 3664.6062, 3937.5706, 3976.6005, 3993.6333

13. Q2KKS8_9PERO **Mass:** 13440 **Score:** 44 **Expect:** 1.4e+02 **Queries matched:** 7

CG11943-PB-like protein (Fragment).- Siniperca chuatsi (Chinese perch).

Observed	Mr(expt)	Mr(calc)	Delta	Start	End	Miss	Peptide
993.4277	992.4205	992.4783	-0.0578	49 -	56	0	K.SLMEVVCR.D + Carbamidomethyl (C)
1064.4451	1063.4378	1063.5186	-0.0807	25 -	34	0	R.LTAPEDGFSK.L
2019.7869	2018.7796	2018.8972	-0.1175	49 -	66	1	K.SLMEVVCRDACDGHEISR.M
2133.9402	2132.9329	2132.9401	-0.0072	49 -	66	1	K.SLMEVVCRDACDGHEISR.M + 2 Carbamidomethyl (C)
2149.9197	2148.9125	2148.9350	-0.0225	49 -	66	1	K.SLMEVVCRDACDGHEISR.M + 2 Carbamidomethyl (C); Oxidation (M)
2210.9927	2209.9855	2210.1074	-0.1220	38 -	56	1	R.ENLAIIESYGKSLMEVVCR.D + Carbamidomethyl (C)
2789.3006	2788.2933	2788.3853	-0.0920	1 -	24	1	-.YLQIAQKPEEPDTLQSAGKAMWER.L

No match to: 751.3859, 793.3455, 832.2988, 848.3093, 861.0016, 865.3301, 876.9628, 892.9507, 915.4276, 988.3750, 1005.3922, 1167.4829, 1216.4928, 1264.5354, 1501.7024, 1545.5785, 1549.6700, 1565.6430, 1622.7394, 1729.6508, 1730.5932, 1744.7499, 1747.6291, 1761.7506, 1832.7851, 1848.8553, 1866.8770, 1889.8854, 2046.8381, 2062.8302, 2063.8573, 2079.8709, 2095.8419, 2117.7673, 2264.8464, 2334.0437, 2450.0481, 2466.9528, 2519.1418, 2535.1542, 2551.9627, 2589.1406, 2611.1242, 2644.0843, 2717.0583, 2732.2341, 3110.3047, 3163.4230, 3647.6063, 3664.6062, 3937.5706, 3976.6005, 3993.6333

14. AAM13420 **Mass:** 103703 **Score:** 44 **Expect:** 1.4e+02 **Queries matched:** 14

AF443596 NID: - Zea mays

Observed	Mr(expt)	Mr(calc)	Delta	Start	End	Miss	Peptide
751.3859	750.3787	750.4024	-0.0238	821 -	826	1	R.GKIYDR.E
793.3455	792.3382	792.3661	-0.0278	722 -	727	1	K.SQCRSR.Q + Carbamidomethyl (C)
993.4277	992.4205	992.3692	0.0513	737 -	744	0	R.ECDPDVCR.N + Carbamidomethyl (C)
1005.3922	1004.3850	1004.4311	-0.0462	913 -	921	0	K.DDGQPFNGR.A
1167.4829	1166.4756	1166.5163	-0.0408	399 -	409	0	K.SQQSESSSTAR.V
1549.6700	1548.6627	1548.7314	-0.0687	182 -	194	1	R.NQRMTEDQSVLGR.R + Oxidation (M)
1832.7851	1831.7778	1831.8093	-0.0315	745 -	761	0	R.NCWVGCGDGTLGVPNQR.G + Carbamidomethyl (C)
1889.8854	1888.8781	1888.8308	0.0473	745 -	761	0	R.NCWVGCGDGTLGVPNQR.G + 2 Carbamidomethyl (C)
2149.9197	2148.9125	2148.9469	-0.0345	333 -	351	0	K.QPAWSGVDDSVPCGIHCHK.L + 2 Carbamidomethyl (C)
2264.8464	2263.8392	2263.8611	-0.0219	690 -	708	1	K.QCPCLSNGTCCEKYCGCPK.I + 4 Carbamidomethyl (C)

```
2611.1242  2610.1169  2610.1658  -0.0489   236 - 257   1  R.MTVQECGMSDAVLQTLARHMER.A + Carbamidomethyl (C); 3 Oxidation (M)
3647.6063  3646.5990  3646.6755  -0.0765   319 - 351   1  K.LHGCSQDLVFPTEKQPAWSGVDDSVPCGIHCHK.L + Carbamidomethyl (C)
3664.6062  3663.5989  3663.8703  -0.2714    23 -  58   1  R.SRPSSSAAQVTSNSAVRAGEENAASLYVLSVIDSLK.K
3937.5706  3936.5633  3936.7642  -0.2009   352 - 388   1  K.LASEPDAAAGADHMLFDVEEPTHSSDNVMNQPGSNRK.K
```

No match to: 832.2988, 848.3093, 861.0016, 865.3301, 876.9628, 892.9507, 915.4276, 988.3750, 1064.4451, 1216.4928, 1264.5354, 1501.7024, 1545.5785, 1565.6430, 1622.7394, 1729.6508, 1730.5932, 1744.7499, 1747.6291, 1761.7506, 1848.8553, 1866.8770, 2019.7869, 2046.8381, 2062.8302, 2063.8573, 2079.8709, 2095.8419, 2117.7673, 2133.9402, 2210.9927, 2334.0437, 2450.0481, 2466.9528, 2519.1418, 2535.1542, 2551.9627, 2589.1406, 2644.0843, 2717.0583, 2732.2341, 2789.3006, 3110.3047, 3163.4230, 3976.6005, 3993.6333

15. Q31EJ1_THICR **Mass:** 23625 **Score:** 44 **Expect:** 1.4e+02 **Queries matched:** 7

Methionine biosynthesis MetW.- Thiomicrospira crunogena (strain XCL-2).

Observed	Mr(expt)	Mr(calc)	Delta	Start		End	Miss	Peptide
988.3750	987.3677	987.4596	-0.0919	180	-	187	0	R.HEPTFAMR.F
1264.5354	1263.5281	1263.6169	-0.0888	1	-	11	0	-.MTSQPLSPEFK.L
1549.6700	1548.6627	1548.8088	-0.1461	188	-	200	0	R.FAPNWLGEIALYR.V
1744.7499	1743.7427	1743.8627	-0.1200	125	-	139	1	R.NRAQLFFGGHMPQNK.S
2519.1418	2518.1345	2518.2579	-0.1234	180	-	200	1	R.HEPTFAMRFAPNWLGEIALYR.V
2535.1542	2534.1469	2534.2528	-0.1059	180	-	200	1	R.HEPTFAMRFAPNWLGEIALYR.V + Oxidation (M)
3647.6063	3646.5990	3646.7759	-0.1769	23	-	54	1	R.VLDLGCGDGQLLDYLIQHRNITGYGMEIDPQK.N + Carbamidomethyl (C); Oxidation (M)

No match to: 751.3859, 793.3455, 832.2988, 848.3093, 861.0016, 865.3301, 876.9628, 892.9507, 915.4276, 993.4277, 1005.3922, 1064.4451, 1167.4829, 1216.4928, 1501.7024, 1545.5785, 1565.6430, 1622.7394, 1729.6508, 1730.5932, 1747.6291, 1761.7506, 1832.7851, 1848.8553, 1866.8770, 1889.8854, 2019.7869, 2046.8381, 2062.8302, 2063.8573, 2079.8709, 2095.8419, 2117.7673, 2133.9402, 2149.9197, 2210.9927, 2264.8464, 2334.0437, 2450.0481, 2466.9528, 2551.9627, 2589.1406, 2611.1242, 2644.0843, 2717.0583, 2732.2341, 2789.3006, 3110.3047, 3163.4230, 3664.6062, 3937.5706, 3976.6005, 3993.6333

16. C72150 **Mass:** 15663 **Score:** 43 **Expect:** 1.5e+02 **Queries matched:** 6

B3R protein - variola minor virus (strain Garcia-1966)

Observed	Mr(expt)	Mr(calc)	Delta	Start		End	Miss	Peptide
988.3750	987.3677	987.3790	-0.0113	63	-	70	0	R.DIDGMYCR.C + Oxidation (M)
993.4277	992.4205	992.4498	-0.0293	71	-	79	0	R.CSHGYTGIR.C
1848.8553	1847.8480	1847.9711	-0.1231	2	-	16	1	M.SMKYLMLLFAAMIIR.S + 3 Oxidation (M)
2019.7869	2018.7796	2018.8397	-0.0600	63	-	79	1	R.DIDGMYCRCSHGYTGIR.C + Carbamidomethyl (C); Oxidation (M)
2046.8381	2045.8308	2045.9022	-0.0714	44	-	62	0	R.LCGPEGNGYCFHGICIHAR.D
2334.0437	2333.0364	2333.1157	-0.0792	71	-	90	1	R.CSHGYTGIRCQHVVLVDYQR.S

No match to: 751.3859, 793.3455, 832.2988, 848.3093, 861.0016, 865.3301, 876.9628, 892.9507, 915.4276, 1005.3922, 1064.4451, 1167.4829, 1216.4928, 1264.5354, 1501.7024, 1545.5785, 1549.6700, 1565.6430, 1622.7394, 1729.6508, 1730.5932, 1744.7499, 1747.6291, 1761.7506, 1832.7851, 1866.8770, 1889.8854, 2062.8302, 2063.8573, 2079.8709, 2095.8419, 2117.7673, 2133.9402, 2149.9197, 2210.9927, 2264.8464, 2450.0481, 2466.9528, 2519.1418, 2535.1542, 2551.9627, 2589.1406, 2611.1242, 2644.0843, 2717.0583, 2732.2341, 2789.3006, 3110.3047, 3163.4230, 3647.6063, 3664.6062, 3937.5706, 3976.6005, 3993.6333

17. T28439 **Mass:** 15693 **Score:** 43 **Expect:** 1.5e+02 **Queries matched:** 6

hypothetical protein D4R - variola major virus

Observed	Mr(expt)	Mr(calc)	Delta	Start		End	Miss	Peptide
988.3750	987.3677	987.3790	-0.0113	63	-	70	0	R.DIDGMYCR.C + Oxidation (M)
993.4277	992.4205	992.4498	-0.0293	71	-	79	0	R.CSHGYTGIR.C
1848.8553	1847.8480	1847.9711	-0.1231	2	-	16	1	M.SMKYLMLLFAAMIIR.S + 3 Oxidation (M)
2019.7869	2018.7796	2018.8397	-0.0600	63	-	79	1	R.DIDGMYCRCSHGYTGIR.C + Carbamidomethyl (C); Oxidation (M)
2046.8381	2045.8308	2045.9022	-0.0714	44	-	62	0	R.LCGPEGNGYCFHGICIHAR.D
2334.0437	2333.0364	2333.1157	-0.0792	71	-	90	1	R.CSHGYTGIRCQHVVLVDYQR.S

No match to: 751.3859, 793.3455, 832.2988, 848.3093, 861.0016, 865.3301, 876.9628, 892.9507, 915.4276, 1005.3922, 1064.4451, 1167.4829, 1216.4928, 1264.5354, 1501.7024, 1545.5785, 1549.6700, 1565.6430, 1622.7394, 1729.6508, 1730.5932, 1744.7499, 1747.6291, 1761.7506, 1832.7851, 1866.8770, 1889.8854, 2062.8302, 2063.8573, 2079.8709, 2095.8419, 2117.7673, 2133.9402, 2149.9197, 2210.9927, 2264.8464, 2450.0481, 2466.9528, 2519.1418, 2535.1542, 2551.9627, 2589.1406, 2611.1242, 2644.0843, 2717.0583, 2732.2341, 2789.3006, 3110.3047, 3163.4230, 3647.6063, 3664.6062, 3937.5706, 3976.6005, 3993.6333

18. S30979 **Mass:** 10815 **Score:** 43 **Expect:** 1.6e+02 **Queries matched:** 6

gene 34 protein - Mycobacterium phage L5

Observed	Mr(expt)	Mr(calc)	Delta	Start		End	Miss	Peptide
1501.7024	1500.6952	1500.6553	0.0399	64	-	77	1	R.QGERQPADGEGSDR.C
1848.8553	1847.8480	1847.8909	-0.0429	82	-	98	0	R.CNLVPVYSLHCTSSNLV.-
2079.8709	2078.8636	2078.9565	-0.0929	2	-	19	1	M.CHVAGHRYDGGQDQQRPR.V
2210.9927	2209.9855	2209.9970	-0.0115	1	-	19	1	-.MCHVAGHRYDGGQDQQRPR.V
2732.2341	2731.2268	2731.3473	-0.1205	37	-	61	1	R.QAVDHQGHHLCLGHPVGRSQLAESR.E
2789.3006	2788.2933	2788.3687	-0.0754	37	-	61	1	R.QAVDHQGHHLCLGHPVGRSQLAESR.E + Carbamidomethyl (C)

No match to: 751.3859, 793.3455, 832.2988, 848.3093, 861.0016, 865.3301, 876.9628, 892.9507, 915.4276, 988.3750, 993.4277, 1005.3922, 1064.4451, 1167.4829, 1216.4928, 1264.5354, 1545.5785, 1549.6700, 1565.6430, 1622.7394, 1729.6508, 1730.5932, 1744.7499, 1747.6291, 1761.7506, 1832.7851, 1866.8770, 1889.8854, 2019.7869, 2046.8381, 2062.8302, 2063.8573, 2095.8419, 2117.7673, 2133.9402, 2149.9197, 2264.8464, 2334.0437, 2450.0481, 2466.9528, 2519.1418, 2535.1542, 2551.9627, 2589.1406, 2611.1242, 2644.0843, 2717.0583, 3110.3047, 3163.4230, 3647.6063, 3664.6062, 3937.5706, 3976.6005, 3993.6333

19. Q3F6Z7_9BURK **Mass:** 46446 **Score:** 43 **Expect:** 1.7e+02 **Queries matched:** 9

Extracellular ligand-binding receptor.- Burkholderia ambifaria AMMD.

Observed	Mr(expt)	Mr(calc)	Delta	Start		End	Miss	Peptide
848.3093	847.3020	847.3680	-0.0660	38	-	45	0	R.ACPMSSPR.V
915.4276	914.4203	914.4933	-0.0730	305	-	312	1	R.QAREQGVK.A

1545.5785	1544.5712	1544.6197	-0.0484	136 - 148	0	K.FEVVMMDDACEPK.Q + 2 Oxidation (M)
2046.8381	2045.8308	2045.9146	-0.0838	2 - 19	1	M.QDASRCLSEPDVPIGDCK.S + 2 Carbamidomethyl (C)
2063.8573	2062.8500	2062.9122	-0.0621	1 - 19	1	-.MQDASRCLSEPDVPIGDCK.S
2079.8709	2078.8636	2078.9071	-0.0435	1 - 19	1	-.MQDASRCLSEPDVPIGDCK.S + Oxidation (M)
2535.1542	2534.1469	2534.2838	-0.1369	364 - 388	1	R.DANGPFQMPSYAAVKIIADSIAGAK.T
2644.0843	2643.0770	2643.2977	-0.2206	282 - 304	1	K.LKSQGVDFVYFGGYHPEMGLLMR.Q
3110.3047	3109.2975	3109.5146	-0.2172	46 - 72	1	R.VLFDCASHAAFARMTNNIEELLMTLSR.L + Carbamidomethyl (C)

No match to: 751.3859, 793.3455, 832.2988, 861.0016, 865.3301, 876.9628, 892.9507, 988.3750, 993.4277, 1005.3922, 1064.4451, 1167.4829, 1216.4928, 1264.5354, 1501.7024, 1549.6700, 1565.6430, 1622.7394, 1729.6508, 1730.5932, 1744.7499, 1747.6291, 1761.7506, 1832.7851, 1848.8553, 1866.8770, 1889.8854, 2019.7869, 2062.8302, 2095.8419, 2117.7673, 2133.9402, 2149.9197, 2210.9927, 2264.8464, 2334.0437, 2450.0481, 2466.9528, 2519.1418, 2551.9627, 2589.1406, 2611.1242, 2717.0583, 2732.2341, 2789.3006, 3163.4230, 3647.6063, 3664.6062, 3937.5706, 3976.6005, 3993.6333

20. Q2XLE6_PSEPU **Mass:** 48195 **Score:** 42 **Expect:** 1.8e+02 **Queries matched:** 8

Toluene transport protein, putative precursor.- Pseudomonas putida F1.

Observed	Mr(expt)	Mr(calc)	Delta	Start - End	Miss	Peptide
1565.6430	1564.6358	1564.7919	-0.1561	424 - 437	0	R.VQTPSYLAGTEMLR.Q
1866.8770	1865.8697	1865.9305	-0.0608	422 - 437	1	R.ERVQTPSYLAGTEMLR.Q + Oxidation (M)
2063.8573	2062.8500	2063.0112	-0.1611	97 - 114	0	R.GPYVGPQLSYVAQLDDWR.F
2117.7673	2116.7600	2116.9661	-0.2061	133 - 151	0	K.SFLSQTENGIQTSFDNSSR.L
2149.9197	2148.9125	2149.0915	-0.1790	227 - 248	1	R.NSTAGGAVDAVGWGGRLGLTYK.L
2519.1418	2518.1345	2518.2703	-0.1358	350 - 371	1	R.YQDITVASIGTAYKYNNDLTLR.A
2535.1542	2534.1469	2534.2302	-0.0833	93 - 114	1	R.SNNRGPYVGPQLSYVAQLDDWR.F
3110.3047	3109.2975	3109.5502	-0.2527	424 - 451	1	R.VQTPSYLAGTEMLRQSHSQINAVVSYSK.N + Oxidation (M)

No match to: 751.3859, 793.3455, 832.2988, 848.3093, 861.0016, 865.3301, 876.9628, 892.9507, 915.4276, 988.3750, 993.4277, 1005.3922, 1064.4451, 1167.4829, 1216.4928, 1264.5354, 1501.7024, 1545.5785, 1549.6700, 1622.7394, 1729.6508, 1730.5932, 1744.7499, 1747.6291, 1761.7506, 1832.7851, 1848.8553, 1889.8854, 2019.7869, 2046.8381, 2062.8302, 2079.8709, 2095.8419, 2133.9402, 2210.9927, 2264.8464, 2334.0437, 2450.0481, 2466.9528, 2551.9627, 2589.1406, 2611.1242, 2644.0843, 2717.0583, 2732.2341, 2789.3006, 3163.4230, 3647.6063, 3664.6062, 3937.5706, 3976.6005, 3993.6333

21. B36837 **Mass:** 15761 **Score:** 42 **Expect:** 1.8e+02 **Queries matched:** 6

D1L protein - variola virus (strain India-1967)

Observed	Mr(expt)	Mr(calc)	Delta	Start - End	Miss	Peptide
988.3750	987.3677	987.3790	-0.0113	63 - 70	0	R.DIDGMYCR.C + Oxidation (M)
993.4277	992.4205	992.4498	-0.0293	71 - 79	0	R.CSHGYTGIR.C
1848.8553	1847.8480	1847.9711	-0.1231	2 - 16	1	M.SMKYLMLLFAAMIIR.S + 3 Oxidation (M)
2019.7869	2018.7796	2018.8397	-0.0600	63 - 79	1	R.DIDGMYCRCSHGYTGIR.C + Carbamidomethyl (C); Oxidation (M)
2334.0437	2333.0364	2333.1157	-0.0792	71 - 90	1	R.CSHGYTGIRCQHVVLVDYQR.S
3664.6062	3663.5989	3663.7686	-0.1697	17 - 51	1	R.SFANSGNAIETTLSEITNTTTDIPAIRLCGPEGDR.Y

No match to: 751.3859, 793.3455, 832.2988, 848.3093, 861.0016, 865.3301, 876.9628, 892.9507, 915.4276, 1005.3922, 1064.4451, 1167.4829, 1216.4928, 1264.5354, 1501.7024, 1545.5785, 1549.6700, 1565.6430, 1622.7394, 1729.6508, 1730.5932, 1744.7499, 1747.6291, 1761.7506, 1832.7851, 1866.8770, 1889.8854, 2046.8381, 2062.8302, 2063.8573, 2079.8709, 2095.8419, 2117.7673, 2133.9402, 2149.9197, 2210.9927, 2264.8464, 2450.0481, 2466.9528, 2519.1418, 2535.1542, 2551.9627, 2589.1406, 2611.1242, 2644.0843, 2717.0583, 2732.2341, 2789.3006, 3110.3047, 3163.4230, 3647.6063, 3937.5706, 3976.6005, 3993.6333

22. AAU28929 **Mass:** 78546 **Score:** 42 **Expect:** 1.9e+02 **Queries matched:** 10

AE017354 NID: - Legionella pneumophila subsp. pneumophila str. Philadelphia 1

Observed	Mr(expt)	Mr(calc)	Delta	Start - End	Miss	Peptide
793.3455	792.3382	792.4130	-0.0747	359 - 365	0	R.GTFIEAR.A
1264.5354	1263.5281	1263.6459	-0.1178	636 - 646	1	K.KAYDAEELIGR.L
1501.7024	1500.6952	1500.8334	-0.1382	616 - 628	1	K.LMRLILDLGDAQK.Q + Oxidation (M)
1565.6430	1564.6358	1564.7481	-0.1123	425 - 437	1	R.FDNRLSSELNDQK.L
1761.7506	1760.7434	1760.8291	-0.0857	14 - 28	0	R.FIIGDEYSITMTSER.K
1848.8553	1847.8480	1847.9417	-0.0937	337 - 353	0	R.TPTAVYTHGFLTVEGQK.M
1889.8854	1888.8781	1888.9240	-0.0459	14 - 29	1	R.FIIGDEYSITMTSERK.M
2210.9927	2209.9855	2210.1041	-0.1186	337 - 356	1	R.TPTAVYTHGFLTVEGQKMSK.S + Oxidation (M)
2334.0437	2333.0364	2333.0887	-0.0523	63 - 84	0	K.MLGIQCISVCGDDAHGTPIMLK.A + 2 Oxidation (M)
3647.6063	3646.5990	3646.9474	-0.3484	491 - 521	1	K.LNEVHAICTMGINLFRILITYLKPVLPMMAK.A + Carbamidomethyl (C); 3 Oxidation (M)

No match to: 751.3859, 832.2988, 848.3093, 861.0016, 865.3301, 876.9628, 892.9507, 915.4276, 988.3750, 993.4277, 1005.3922, 1064.4451, 1167.4829, 1216.4928, 1545.5785, 1549.6700, 1622.7394, 1729.6508, 1730.5932, 1744.7499, 1747.6291, 1832.7851, 1866.8770, 2019.7869, 2046.8381, 2062.8302, 2063.8573, 2079.8709, 2095.8419, 2117.7673, 2133.9402, 2149.9197, 2264.8464, 2450.0481, 2466.9528, 2519.1418, 2535.1542, 2551.9627, 2589.1406, 2611.1242, 2644.0843, 2717.0583, 2732.2341, 2789.3006, 3110.3047, 3163.4230, 3664.6062, 3937.5706, 3976.6005, 3993.6333

23. Q6DIG7_XENTR **Mass:** 13411 **Score:** 42 **Expect:** 2e+02 **Queries matched:** 5

Cyclin-dependent kinase inhibitor 2B (P15, inhibits CDK4).- Xenopus tropicalis (Western clawed frog) (Silurana tropicalis).

Observed	Mr(expt)	Mr(calc)	Delta	Start - End	Miss	Peptide
1545.5785	1544.5712	1544.6534	-0.0821	1 - 14	0	-.MAFNANTLCSACAR.G + Carbamidomethyl (C); Oxidation (M)
1622.7394	1621.7321	1621.7341	-0.0020	64 - 79	0	K.LPDPCTGACPVHDAAR.E
2334.0437	2333.0364	2333.2266	-0.1902	80 - 100	0	R.EGFLDTLLVLLNNGASLYEPR.D
3110.3047	3109.2975	3109.5293	-0.2318	22 - 50	1	R.QMLQSGIPVNATNSHGRTPIQVMMMGSPK.M
3937.5706	3936.5633	3936.9501	-0.3868	64 - 100	1	K.LPDPCTGACPVHDAAREGFLDTLLVLLNNGASLYEPR.D

No match to: 751.3859, 793.3455, 832.2988, 848.3093, 861.0016, 865.3301, 876.9628, 892.9507, 915.4276, 988.3750, 993.4277, 1005.3922, 1064.4451, 1167.4829, 1216.4928, 1264.5354, 1501.7024, 1549.6700, 1565.6430, 1729.6508, 1730.5932, 1744.7499, 1747.6291, 1761.7506, 1832.7851, 1848.8553, 1866.8770, 1889.8854, 2019.7869, 2046.8381, 2062.8302, 2063.8573, 2079.8709, 2095.8419, 2117.7673, 2133.9402, 2149.9197, 2210.9927, 2264.8464, 2450.0481, 2466.9528, 2519.1418, 2535.1542, 2551.9627, 2589.1406, 2611.1242, 2644.0843, 2717.0583, 2732.2341, 2789.3006, 3163.4230, 3647.6063, 3664.6062, 3976.6005, 3993.6333

24. Q1Z385_PHOPR **Mass:** 33824 **Score:** 42 **Expect:** 2e+02 **Queries matched:** 9

Putative transcriptional activator protein NhaR.- Photobacterium profundum 3TCK.

Observed	Mr(expt)	Mr(calc)	Delta	Start		End	Miss	Peptide
751.3859	750.3787	750.3912	-0.0125	157	-	163	0	K.SPGLYSK.K
1549.6700	1548.6627	1548.7164	-0.0536	143	-	156	0	K.LDMILSDCPVDSGK.S + Carbamidomethyl (C)
1565.6430	1564.6358	1564.7113	-0.0755	143	-	156	0	K.LDMILSDCPVDSGK.S + Carbamidomethyl (C); Oxidation (M)
2117.7673	2116.7600	2116.9651	-0.2051	2	-	17	0	M.SHLNYNHLYYFWMVCK.Q
2133.9402	2132.9329	2132.9600	-0.0271	2	-	17	0	M.SHLNYNHLYYFWMVCK.Q + Oxidation (M)
2264.8464	2263.8392	2264.0005	-0.1613	1	-	17	0	-.MSHLNYNHLYYFWMVCK.Q + Oxidation (M)
2644.0843	2643.0770	2643.3149	-0.2378	122	-	142	1	R.IQLRCYESTHEMLLEQLSLHK.L + Carbamidomethyl (C); Oxidation (M)
3647.6063	3646.5990	3646.7785	-0.1795	74	-	105	0	K.MFDLSYEMLDIVNYTQQDNLLLEVGVADALSK.R
3664.6062	3663.5989	3663.6928	-0.0940	126	-	156	1	R.CYESTHEMLLEQLSLHKLDMILSDCPVDSGK.S + 2 Carbamidomethyl (C); Oxidation (M)

No match to: 793.3455, 832.2988, 848.3093, 861.0016, 865.3301, 876.9628, 892.9507, 915.4276, 988.3750, 993.4277, 1005.3922, 1064.4451, 1167.4829, 1216.4928, 1264.5354, 1501.7024, 1545.5785, 1622.7394, 1729.6508, 1730.5932, 1744.7499, 1747.6291, 1761.7506, 1832.7851, 1848.8553, 1866.8770, 1889.8854, 2019.7869, 2046.8381, 2062.8302, 2063.8573, 2079.8709, 2095.8419, 2149.9197, 2210.9927, 2334.0437, 2450.0481, 2466.9528, 2519.1418, 2535.1542, 2551.9627, 2589.1406, 2611.1242, 2717.0583, 2732.2341, 2789.3006, 3110.3047, 3163.4230, 3937.5706, 3976.6005, 3993.6333

25. C96537 **Mass:** 39973 **Score:** 42 **Expect:** 2e+02 **Queries matched:** 8

hypothetical protein F2J10.5 [imported] - Arabidopsis thaliana

Observed	Mr(expt)	Mr(calc)	Delta	Start		End	Miss	Peptide
1622.7394	1621.7321	1621.8385	-0.1064	235	-	248	0	K.NIEEVSTCNIFILK.G
1832.7851	1831.7778	1831.8848	-0.1070	109	-	124	0	R.LCMTPPSPEQFVEAVK.Q + Carbamidomethyl (C)
1848.8553	1847.8480	1847.8797	-0.0317	109	-	124	0	R.LCMTPPSPEQFVEAVK.Q + Carbamidomethyl (C); Oxidation (M)
2133.9402	2132.9329	2133.0128	-0.0799	26	-	43	0	K.WDELGFALVPTDYMYVAK.C + Oxidation (M)
2535.1542	2534.1469	2534.1967	-0.0498	102	-	124	1	R.MQTGADRLCMTPPSPEQFVEAVK.Q
2644.0843	2643.0770	2643.3400	-0.2629	109	-	132	1	R.LCMTPPSPEQFVEAVKQTVLANNK.W
2789.3006	2788.2933	2788.4316	-0.1383	323	-	347	1	R.TGEEAFSTKLHLILTNIQMGVVEDK.K + Oxidation (M)
3664.6062	3663.5989	3663.8228	-0.2240	285	-	317	1	R.DLSVDELLEAEEVFCTGTAVVIKAVETVTFHDK.R + Carbamidomethyl (C)

No match to: 751.3859, 793.3455, 832.2988, 848.3093, 861.0016, 865.3301, 876.9628, 892.9507, 915.4276, 988.3750, 993.4277, 1005.3922, 1064.4451, 1167.4829, 1216.4928, 1264.5354, 1501.7024, 1545.5785, 1549.6700, 1565.6430, 1729.6508, 1730.5932, 1744.7499, 1747.6291, 1761.7506, 1866.8770, 1889.8854, 2019.7869, 2046.8381, 2062.8302, 2063.8573, 2079.8709, 2095.8419, 2117.7673, 2149.9197, 2210.9927, 2264.8464, 2334.0437, 2450.0481, 2466.9528, 2519.1418, 2551.9627, 2589.1406, 2611.1242, 2717.0583, 2732.2341, 3110.3047, 3163.4230, 3647.6063, 3937.5706, 3976.6005, 3993.6333

26. Q5JMR0_ORYSA **Mass:** 22633 **Score:** 42 **Expect:** 2.1e+02 **Queries matched:** 6

Sperm protein-like.- Oryza sativa (japonica cultivar-group).

Observed	Mr(expt)	Mr(calc)	Delta	Start		End	Miss	Peptide
1216.4928	1215.4856	1215.5918	-0.1062	182	-	193	0	K.CSPPVTSADALR.V
1501.7024	1500.6952	1500.7460	-0.0508	58	-	71	1	R.LSSIFDAAKSDGYK.C
2079.8709	2078.8636	2079.0207	-0.1571	67	-	84	1	K.SDGYKCNVEHLLAVFQTR.L
2133.9402	2132.9329	2133.0854	-0.1524	18	-	37	1	K.QGGGYFNTPGEDPSVLLRVK.E
2334.0437	2333.0364	2333.1615	-0.1250	87	-	108	1	R.ELGIALPLMCCAADMLSVPSRK.Q + Oxidation (M)
2589.1406	2588.1334	2588.2946	-0.1613	85	-	107	1	R.LRELGIALPLMCCAADMLSVPSR.K + 2 Carbamidomethyl (C); Oxidation (M)

No match to: 751.3859, 793.3455, 832.2988, 848.3093, 861.0016, 865.3301, 876.9628, 892.9507, 915.4276, 988.3750, 993.4277, 1005.3922, 1064.4451, 1167.4829, 1264.5354, 1545.5785, 1549.6700, 1565.6430, 1622.7394, 1729.6508, 1730.5932, 1744.7499, 1747.6291, 1761.7506, 1832.7851, 1848.8553, 1866.8770, 1889.8854, 2019.7869, 2046.8381, 2062.8302, 2063.8573, 2095.8419, 2117.7673, 2149.9197, 2210.9927, 2264.8464, 2450.0481, 2466.9528, 2519.1418, 2535.1542, 2551.9627, 2611.1242, 2644.0843, 2717.0583, 2732.2341, 2789.3006, 3110.3047, 3163.4230, 3647.6063, 3664.6062, 3937.5706, 3976.6005, 3993.6333

27. Q4DF81_TRYCR **Mass:** 73363 **Score:** 42 **Expect:** 2.2e+02 **Queries matched:** 11

Hypothetical protein.- Trypanosoma cruzi.

Observed	Mr(expt)	Mr(calc)	Delta	Start		End	Miss	Peptide
793.3455	792.3382	792.3436	-0.0054	58	-	64	0	R.ANSVDCK.H + Carbamidomethyl (C)
865.3301	864.3228	864.3977	-0.0749	471	-	477	0	R.VENFNDK.G
1501.7024	1500.6952	1500.7393	-0.0441	279	-	290	1	R.KQQQQQQQSSR.H
1545.5785	1544.5712	1544.6815	-0.1102	138	-	149	1	K.QQQQREGEEEER.N
1761.7506	1760.7434	1760.8727	-0.1293	155	-	169	1	R.IAVTDECTVVDLDRR.R + Carbamidomethyl (C)
1832.7851	1831.7778	1831.9098	-0.1320	153	-	168	1	R.ARIAVTDECTVVDLDR.R + Carbamidomethyl (C)
1848.8553	1847.8480	1847.7712	0.0768	58	-	73	1	R.ANSVDCKHNEAECCLR.R + Carbamidomethyl (C)
2046.8381	2045.8308	2045.9013	-0.0705	653	-	670	1	R.NSLAPVDANWHCEGYSRA.- + Carbamidomethyl (C)
2519.1418	2518.1345	2518.1077	0.0268	536	-	558	1	K.NDAERAGAAFDQLPSSHTNCMAR.F + Carbamidomethyl (C)
2535.1542	2534.1469	2534.1026	0.0443	536	-	558	1	K.NDAERAGAAFDQLPSSHTNCMAR.F + Carbamidomethyl (C); Oxidation (M)
2589.1406	2588.1334	2588.2275	-0.0941	187	-	214	0	R.MPSESALAAAAGISSGAAASEAIPAEDK.T + Oxidation (M)

No match to: 751.3859, 832.2988, 848.3093, 861.0016, 876.9628, 892.9507, 915.4276, 988.3750, 993.4277, 1005.3922, 1064.4451, 1167.4829, 1216.4928, 1264.5354, 1549.6700, 1565.6430, 1622.7394, 1729.6508, 1730.5932, 1744.7499, 1747.6291, 1866.8770, 1889.8854, 2019.7869, 2062.8302, 2063.8573, 2079.8709, 2095.8419, 2117.7673, 2133.9402, 2149.9197, 2210.9927, 2264.8464, 2334.0437, 2450.0481, 2466.9528, 2551.9627, 2611.1242, 2644.0843, 2717.0583, 2732.2341, 2789.3006, 3110.3047, 3163.4230, 3647.6063, 3664.6062, 3937.5706, 3976.6005, 3993.6333

28. Q1VVY8_9FLAO **Mass:** 7992 **Score:** 42 **Expect:** 2.3e+02 **Queries matched:** 4

Hypothetical protein.- Psychroflexus torquis ATCC 700755.

Observed	Mr(expt)	Mr(calc)	Delta	Start	End	Miss	Peptide
1064.4451	1063.4378	1063.5372	-0.0994	1 -	9	0	-.MTDPVFLNK.G
1565.6430	1564.6358	1564.7256	-0.0899	10 -	23	0	K.GEFDELAGTLQEEK.V
2611.1242	2610.1169	2610.2522	-0.1353	1 -	23	1	-.MTDPVFLNKGEFDELAGTLQEEK.V
2789.3006	2788.2933	2788.4160	-0.1227	46 -	67	0	R.EIMLFLCENLLFFIIFLQFGHW.- + Oxidation (M)

No match to: 751.3859, 793.3455, 832.2988, 848.3093, 861.0016, 865.3301, 876.9628, 892.9507, 915.4276, 988.3750, 993.4277, 1005.3922, 1167.4829, 1216.4928, 1264.5354, 1501.7024, 1545.5785, 1549.6700, 1622.7394, 1729.6508, 1730.5932, 1744.7499, 1747.6291, 1761.7506, 1832.7851, 1848.8553, 1866.8770, 1889.8854, 2019.7869, 2046.8381, 2062.8302, 2063.8573, 2079.8709, 2095.8419, 2117.7673, 2133.9402, 2149.9197, 2210.9927, 2264.8464, 2334.0437, 2450.0481, 2466.9528, 2519.1418, 2535.1542, 2551.9627, 2589.1406, 2644.0843, 2717.0583, 2732.2341, 3110.3047, 3163.4230, 3647.6063, 3664.6062, 3937.5706, 3976.6005, 3993.6333

29. Q8RET8_FUSNN **Mass:** 51567 **Score:** 41 **Expect:** 2.5e+02 **Queries matched:** 9

Adenosylmethionine-8-amino-7-oxononanoate aminotransferase (EC 2.6.1.62).- Fusobacterium nucleatum subsp. nucleatum.

Observed	Mr(expt)	Mr(calc)	Delta	Start	End	Miss	Peptide
1744.7499	1743.7427	1743.8899	-0.1472	438 -	451	1	K.MLHVCKESIEELLK.I + Carbamidomethyl (C); Oxidation (M)
1761.7506	1760.7434	1760.8113	-0.0679	117 -	132	0	K.FLFSDNGSSCIEMALK.L
1832.7851	1831.7778	1831.8424	-0.0646	322 -	337	0	K.SFLHSHTYSGNPLGCR.I + Carbamidomethyl (C)
1848.8553	1847.8480	1847.9419	-0.0939	1 -	16	1	-.MRGVCIMINNLSELQK.K
1889.8854	1888.8781	1888.9795	-0.1014	133 -	148	1	K.LSFQYHLQTGNPQKTK.F
2149.9197	2148.9125	2149.0625	-0.1501	255 -	273	1	K.YNIHLIDDEIAMGFGRTGK.M
3110.3047	3109.2975	3109.4452	-0.1478	292 -	318	0	K.GLSSGYYPIAMLCITTDIFNAFYADYK.E + Carbamidomethyl (C); Oxidation (M)
3163.4230	3162.4157	3162.6099	-0.1942	411 -	437	1	K.KGAFLRPIGNSVYFMPPYVITYEEIDK.M + Oxidation (M)
3664.6062	3663.5989	3663.6220	-0.0231	43 -	73	1	K.GDGLYLIDENGNKYMDCISSWWVNLFGHCNK.R + Carbamidomethyl (C); Oxidation (M)

No match to: 751.3859, 793.3455, 832.2988, 848.3093, 861.0016, 865.3301, 876.9628, 892.9507, 915.4276, 988.3750, 993.4277, 1005.3922, 1064.4451, 1167.4829, 1216.4928, 1264.5354, 1501.7024, 1545.5785, 1549.6700, 1565.6430, 1622.7394, 1729.6508, 1730.5932, 1747.6291, 1866.8770, 2019.7869, 2046.8381, 2062.8302, 2063.8573, 2079.8709, 2095.8419, 2117.7673, 2133.9402, 2210.9927, 2264.8464, 2334.0437, 2450.0481, 2466.9528, 2519.1418, 2535.1542, 2551.9627, 2589.1406, 2611.1242, 2644.0843, 2717.0583, 2732.2341, 2789.3006, 3647.6063, 3937.5706, 3976.6005, 3993.6333

30. Q24161_DROME **Mass:** 47840 **Score:** 41 **Expect:** 2.6e+02 **Queries matched:** 11

CKII alpha subunit interactor 1.- Drosophila melanogaster (Fruit fly).

Observed	Mr(expt)	Mr(calc)	Delta	Start	End	Miss	Peptide
793.3455	792.3382	792.3687	-0.0305	2 -	8	0	M.TEAEICK.L
1005.3922	1004.3850	1004.4531	-0.0682	225 -	233	1	K.HNKTMCGAK.D + Oxidation (M)
1549.6700	1548.6627	1548.7541	-0.0913	213 -	224	1	K.LHELHEHMKTMK.H + Oxidation (M)
1565.6430	1564.6358	1564.7490	-0.1132	213 -	224	1	K.LHELHEHMKTMK.H + 2 Oxidation (M)
1729.6508	1728.6436	1728.7997	-0.1561	228 -	241	1	K.TMCGAKDLEFLCLR.C + 2 Carbamidomethyl (C); Oxidation (M)
1761.7506	1760.7434	1760.7174	0.0260	296 -	309	1	R.FYECPYKDCTSSYR.V
1866.8770	1865.8697	1865.8487	0.0210	190 -	204	0	R.MHSCKPRPNPVYYCR.D + Oxidation (M)
2133.9402	2132.9329	2132.9520	-0.0190	205 -	221	1	R.DCGSEFHKLHELHEHMK.T + Carbamidomethyl (C)
2149.9197	2148.9125	2148.9469	-0.0344	205 -	221	1	R.DCGSEFHKLHELHEHMK.T + Carbamidomethyl (C); Oxidation (M)
3937.5706	3936.5633	3936.7405	-0.1772	249 -	281	1	R.YFDVIRHENSVHNVSDFICVECNMSFANQGSYR.R + Carbamidomethyl (C)
3976.6005	3975.5933	3975.8431	-0.2499	173 -	204	1	R.MLCTCCGHVLEILTHIRMHSCKPRPNPVYYCR.D + 3 Carbamidomethyl (C); 2 Oxidation (M)

No match to: 751.3859, 832.2988, 848.3093, 861.0016, 865.3301, 876.9628, 892.9507, 915.4276, 988.3750, 993.4277, 1064.4451, 1167.4829, 1216.4928, 1264.5354, 1501.7024, 1545.5785, 1622.7394, 1730.5932, 1744.7499, 1747.6291, 1832.7851, 1848.8553, 1889.8854, 2019.7869, 2046.8381, 2062.8302, 2063.8573, 2079.8709, 2095.8419, 2117.7673, 2210.9927, 2264.8464, 2334.0437, 2450.0481, 2466.9528, 2519.1418, 2535.1542, 2551.9627, 2589.1406, 2611.1242, 2644.0843, 2717.0583, 2732.2341, 2789.3006, 3110.3047, 3163.4230, 3647.6063, 3664.6062, 3993.6333

31. CAH17038 **Mass:** 75747 **Score:** 41 **Expect:** 2.8e+02 **Queries matched:** 10

CR628337 NID: - Legionella pneumophila str. Lens

Observed	Mr(expt)	Mr(calc)	Delta	Start	End	Miss	Peptide
751.3859	750.3787	750.3694	0.0093	1 -	6	1	-.MTSERK.M
793.3455	792.3382	792.4130	-0.0747	336 -	342	0	R.GTFIEAR.A
1264.5354	1263.5281	1263.6459	-0.1178	613 -	623	1	K.KAYDAEELIGR.L
1501.7024	1500.6952	1500.8334	-0.1382	593 -	605	1	K.LMRLILDLGDAQK.Q + Oxidation (M)
1545.5785	1544.5712	1544.7147	-0.1435	272 -	284	1	R.GVSFDEFWDKTSK.T
1848.8553	1847.8480	1847.9417	-0.0937	314 -	330	0	R.TPTAVYTHGFLTVEGQK.M
2210.9927	2209.9855	2210.1041	-0.1186	314 -	333	1	R.TPTAVYTHGFLTVEGQKMSK.S + Oxidation (M)
2334.0437	2333.0364	2333.0887	-0.0523	40 -	61	0	K.MLGIQCISVCGDDAHGTPIMLK.A + 2 Oxidation (M)
2789.3006	2788.2933	2788.3932	-0.0999	499 -	522	0	K.ASEEFLNSEPLHWGSIDKPLLNHR.I
3647.6063	3646.5990	3646.9474	-0.3484	468 -	498	1	K.LNEVHAICTMGINLFRILITYLKPVLPMMAK.A + Carbamidomethyl (C); 3 Oxidation (M)

No match to: 832.2988, 848.3093, 861.0016, 865.3301, 876.9628, 892.9507, 915.4276, 988.3750, 993.4277, 1005.3922, 1064.4451, 1167.4829, 1216.4928, 1549.6700, 1565.6430, 1622.7394, 1729.6508, 1730.5932, 1744.7499, 1747.6291, 1761.7506, 1832.7851, 1866.8770, 1889.8854, 2019.7869, 2046.8381, 2062.8302, 2063.8573, 2079.8709, 2095.8419, 2117.7673, 2133.9402, 2149.9197, 2264.8464, 2450.0481, 2466.9528, 2519.1418, 2535.1542, 2551.9627, 2589.1406, 2611.1242, 2644.0843, 2717.0583, 2732.2341, 3110.3047, 3163.4230, 3664.6062, 3937.5706, 3976.6005, 3993.6333

32. Q9VUU3_DROME **Mass:** 47883 **Score:** 40 **Expect:** 2.9e+02 **Queries matched:** 11

CG6215-PA (RE17810p) (RE16596p).- Drosophila melanogaster (Fruit fly).

Observed	Mr(expt)	Mr(calc)	Delta	Start	End	Miss	Peptide
793.3455	792.3382	792.3687	-0.0305	2 -	8	0	M.TEAEICK.L
1005.3922	1004.3850	1004.4531	-0.0682	225 -	233	1	K.HNKTMCGAK.D + Oxidation (M)
1549.6700	1548.6627	1548.7541	-0.0913	213 -	224	1	K.LHELHEHMKTMK.H + Oxidation (M)
1565.6430	1564.6358	1564.7490	-0.1132	213 -	224	1	K.LHELHEHMKTMK.H + 2 Oxidation (M)

1729.6508	1728.6436	1728.7997	-0.1561	228 -	241	1	K.TMCGAKDLEFLCLR.C + 2 Carbamidomethyl (C); Oxidation (M)
1761.7506	1760.7434	1760.7174	0.0260	296 -	309	1	R.FYECPYKDCTSSYR.V
1866.8770	1865.8697	1865.8487	0.0210	190 -	204	0	R.MHSCKPRPNPVYYCR.D + Oxidation (M)
2133.9402	2132.9329	2132.9520	-0.0190	205 -	221	1	R.DCGSEFHKLHELHEHMK.T + Carbamidomethyl (C)
2149.9197	2148.9125	2148.9469	-0.0344	205 -	221	1	R.DCGSEFHKLHELHEHMK.T + Carbamidomethyl (C); Oxidation (M)
3937.5706	3936.5633	3936.7405	-0.1772	249 -	281	1	R.YFDVIRHENSVHNVSDFICVECNMSFANQGSYR.R + Carbamidomethyl (C)
3976.6005	3975.5933	3975.8431	-0.2499	173 -	204	1	R.MLCTCCGHVLEILTHIRMHSCKPRPNPVYYCR.D + 3 Carbamidomethyl (C); 2 Oxidation (M)

No match to: 751.3859, 832.2988, 848.3093, 861.0016, 865.3301, 876.9628, 892.9507, 915.4276, 988.3750, 993.4277, 1064.4451, 1167.4829, 1216.4928, 1264.5354, 1501.7024, 1545.5785, 1622.7394, 1730.5932, 1744.7499, 1747.6291, 1832.7851, 1848.8553, 1889.8854, 2019.7869, 2046.8381, 2062.8302, 2063.8573, 2079.8709, 2095.8419, 2117.7673, 2210.9927, 2264.8464, 2334.0437, 2450.0481, 2466.9528, 2519.1418, 2535.1542, 2551.9627, 2589.1406, 2611.1242, 2644.0843, 2717.0583, 2732.2341, 2789.3006, 3110.3047, 3163.4230, 3647.6063, 3664.6062, 3993.6333

33. HLDD_PSEAE **Mass:** 37165 **Score:** 40 **Expect:** 3.2e+02 **Queries matched:** 7

ADP-L-glycero-D-manno-heptose-6-epimerase (EC 5.1.3.20) (ADP-L- glycero-beta-D-manno-heptose-6-epimerase) (ADP-glyceromanno-heptose 6- epimerase) (ADP-hep 6-ep

Observed	Mr(expt)	Mr(calc)	Delta	Start	End	Miss	Peptide
751.3859	750.3787	750.3660	0.0126	64 -	70	0	R.GDFGTVR.A
1761.7506	1760.7434	1760.8264	-0.0830	71 -	87	0	R.ALFHQGACASTLESNGR.Y
1832.7851	1831.7778	1831.8999	-0.1221	290 -	304	1	R.ARYQSHTCADLELLR.E + Carbamidomethyl (C)
2133.9402	2132.9329	2133.0503	-0.1174	194 -	213	1	R.DGRVELFGEHGGFPPGGHLR.D
2149.9197	2148.9125	2149.0287	-0.1162	23 -	41	1	R.RGETDIIAVDDLTDGEQFR.N
2210.9927	2209.9855	2210.0127	-0.0272	42 -	60	0	R.NLADADIADYLDQNDFLER.Y
2264.8464	2263.8392	2263.9803	-0.1412	305 -	324	1	R.EAGYRDDFQSLEEGVAGYCR.W

No match to: 793.3455, 832.2988, 848.3093, 861.0016, 865.3301, 876.9628, 892.9507, 915.4276, 988.3750, 993.4277, 1005.3922, 1064.4451, 1167.4829, 1216.4928, 1264.5354, 1501.7024, 1545.5785, 1549.6700, 1565.6430, 1622.7394, 1729.6508, 1730.5932, 1744.7499, 1747.6291, 1848.8553, 1866.8770, 1889.8854, 2019.7869, 2046.8381, 2062.8302, 2063.8573, 2079.8709, 2095.8419, 2117.7673, 2334.0437, 2450.0481, 2466.9528, 2519.1418, 2535.1542, 2551.9627, 2589.1406, 2611.1242, 2644.0843, 2717.0583, 2732.2341, 2789.3006, 3110.3047, 3163.4230, 3647.6063, 3664.6062, 3937.5706, 3976.6005, 3993.6333

34. C83228 **Mass:** 37394 **Score:** 40 **Expect:** 3.3e+02 **Queries matched:** 7

ADP-L-glycero-D-mannoheptose 6-epimerase PA3337 [imported] - Pseudomonas aeruginosa (strain PAO1)

Observed	Mr(expt)	Mr(calc)	Delta	Start	End	Miss	Peptide
751.3859	750.3787	750.3660	0.0126	66 -	72	0	R.GDFGTVR.A
1761.7506	1760.7434	1760.8264	-0.0830	73 -	89	0	R.ALFHQGACASTLESNGR.Y
1832.7851	1831.7778	1831.8999	-0.1221	292 -	306	1	R.ARYQSHTCADLELLR.E + Carbamidomethyl (C)
2133.9402	2132.9329	2133.0503	-0.1174	196 -	215	1	R.DGRVELFGEHGGFPPGGHLR.D
2149.9197	2148.9125	2149.0287	-0.1162	25 -	43	1	R.RGETDIIAVDDLTDGEQFR.N
2210.9927	2209.9855	2210.0127	-0.0272	44 -	62	0	R.NLADADIADYLDQNDFLER.Y
2264.8464	2263.8392	2263.9803	-0.1412	307 -	326	1	R.EAGYRDDFQSLEEGVAGYCR.W

No match to: 793.3455, 832.2988, 848.3093, 861.0016, 865.3301, 876.9628, 892.9507, 915.4276, 988.3750, 993.4277, 1005.3922, 1064.4451, 1167.4829, 1216.4928, 1264.5354, 1501.7024, 1545.5785, 1549.6700, 1565.6430, 1622.7394, 1729.6508, 1730.5932, 1744.7499, 1747.6291, 1848.8553, 1866.8770, 1889.8854, 2019.7869, 2046.8381, 2062.8302, 2063.8573, 2079.8709, 2095.8419, 2117.7673, 2334.0437, 2450.0481, 2466.9528, 2519.1418, 2535.1542, 2551.9627, 2589.1406, 2611.1242, 2644.0843, 2717.0583, 2732.2341, 2789.3006, 3110.3047, 3163.4230, 3647.6063, 3664.6062, 3937.5706, 3976.6005, 3993.6333

35. Q8PZQ4_METMA **Mass:** 31906 **Score:** 40 **Expect:** 3.4e+02 **Queries matched:** 7

Methylenetetrahydrofolate reductase (EC 1.5.1.20).- Methanosarcina mazei (Methanosarcina frisia).

Observed	Mr(expt)	Mr(calc)	Delta	Start	End	Miss	Peptide
1264.5354	1263.5281	1263.6459	-0.1178	135 -	146	1	R.KLDTGVDFAGNK.L
1501.7024	1500.6952	1500.8300	-0.1348	14 -	27	1	K.FLVTAEVSPPKGTR.F
1866.8770	1865.8697	1865.9855	-0.1158	227 -	242	1	K.NISGIHVPEEIMLRMK.D
2149.9197	2148.9125	2149.0646	-0.1521	241 -	260	1	R.MKDASSPMEEGLLIASETIK.E
2611.1242	2610.1169	2610.2669	-0.1499	147 -	171	1	K.LDGGTSFCAGAVSGIDPEKTMQLIK.L + Carbamidomethyl (C); Oxidation (M)
2732.2341	2731.2268	2731.3495	-0.1227	217 -	240	1	K.SLGMAEFMNKNISGIHVPEEIMLR.M + Oxidation (M)
3976.6005	3975.5933	3975.9168	-0.3235	101 -	135	1	R.NICLMTGDYPTCGDHPGSKPVYDVDSVQLIELVRK.L + 2 Carbamidomethyl (C)

No match to: 751.3859, 793.3455, 832.2988, 848.3093, 861.0016, 865.3301, 876.9628, 892.9507, 915.4276, 988.3750, 993.4277, 1005.3922, 1064.4451, 1167.4829, 1216.4928, 1545.5785, 1549.6700, 1565.6430, 1622.7394, 1729.6508, 1730.5932, 1744.7499, 1747.6291, 1761.7506, 1832.7851, 1848.8553, 1889.8854, 2019.7869, 2046.8381, 2062.8302, 2063.8573, 2079.8709, 2095.8419, 2117.7673, 2133.9402, 2210.9927, 2264.8464, 2334.0437, 2450.0481, 2466.9528, 2519.1418, 2535.1542, 2551.9627, 2589.1406, 2644.0843, 2717.0583, 2789.3006, 3110.3047, 3163.4230, 3647.6063, 3664.6062, 3937.5706, 3993.6333

36. Q268R7_MYCVN **Mass:** 26517 **Score:** 40 **Expect:** 3.5e+02 **Queries matched:** 6

Regulatory protein, TetR.- Mycobacterium vanbaalenii PYR-1.

Observed	Mr(expt)	Mr(calc)	Delta	Start	End	Miss	Peptide
793.3455	792.3382	792.3687	-0.0305	189 -	195	0	K.TIDDCVK.R
1216.4928	1215.4856	1215.5712	-0.0857	45 -	54	0	K.GAFYHYFPSK.E
1866.8770	1865.8697	1865.9370	-0.0673	27 -	44	0	R.GYENVSLNDLLAVSGTSK.G
2062.8302	2061.8229	2062.0049	-0.1820	222 -	240	0	K.QVEAMMTLFMPSRPAGSLP.-
2589.1406	2588.1334	2588.3454	-0.2120	88 -	112	1	R.LNAGLAASYQVKLGMGVPEPIAAMR.S + 2 Oxidation (M)
2789.3006	2788.2933	2788.4073	-0.1140	100 -	124	1	K.LGMGVPEPIAAMRSLLMPENQALFR.K + 3 Oxidation (M)

No match to: 751.3859, 832.2988, 848.3093, 861.0016, 865.3301, 876.9628, 892.9507, 915.4276, 988.3750, 993.4277, 1005.3922, 1064.4451, 1167.4829, 1264.5354, 1501.7024, 1545.5785, 1549.6700, 1565.6430, 1622.7394, 1729.6508, 1730.5932, 1744.7499, 1747.6291, 1761.7506, 1832.7851, 1848.8553, 1889.8854, 2019.7869, 2046.8381, 2063.8573, 2079.8709, 2095.8419, 2117.7673, 2133.9402, 2149.9197, 2210.9927, 2264.8464, 2334.0437, 2450.0481, 2466.9528, 2519.1418, 2535.1542, 2551.9627, 2611.1242, 2644.0843, 2717.0583, 2732.2341, 3110.3047, 3163.4230, 3647.6063, 3664.6062, 3937.5706, 3976.6005, 3993.6333

37. Q39SP8_GEOMG **Mass:** 5632 **Score:** 40 **Expect:** 3.6e+02 **Queries matched:** 4

Hypothetical protein.- Geobacter metallireducens (strain GS-15 / ATCC 53774 / DSM 7210).

Observed	Mr(expt)	Mr(calc)	Delta	Start	End	Miss	Peptide
865.3301	864.3228	864.3656	-0.0428	10 -	16	0	K.MHMCALK.A + 2 Oxidation (M)
1005.3922	1004.3850	1004.4419	-0.0570	1 -	9	0	-.MACDITSHK.M
1761.7506	1760.7434	1760.7830	-0.0397	2 -	16	1	M.ACDITSHKMHMCALK.A + Carbamidomethyl (C); Oxidation (M)
2611.1242	2610.1169	2610.1797	-0.0628	10 -	34	1	K.MHMCALKAEGCSDEIASLSASPTVK.C + 2 Oxidation (M)

No match to: 751.3859, 793.3455, 832.2988, 848.3093, 861.0016, 876.9628, 892.9507, 915.4276, 988.3750, 993.4277, 1064.4451, 1167.4829, 1216.4928, 1264.5354, 1501.7024, 1545.5785, 1549.6700, 1565.6430, 1622.7394, 1729.6508, 1730.5932, 1744.7499, 1747.6291, 1832.7851, 1848.8553, 1866.8770, 1889.8854, 2019.7869, 2046.8381, 2062.8302, 2063.8573, 2079.8709, 2095.8419, 2117.7673, 2133.9402, 2149.9197, 2210.9927, 2264.8464, 2334.0437, 2450.0481, 2466.9528, 2519.1418, 2535.1542, 2551.9627, 2589.1406, 2644.0843, 2717.0583, 2732.2341, 2789.3006, 3110.3047, 3163.4230, 3647.6063, 3664.6062, 3937.5706, 3976.6005, 3993.6333

38. PN0600 **Mass:** 8559 **Score:** 40 **Expect:** 3.6e+02 **Queries matched:** 4

DNA-directed RNA polymerase (EC 2.7.7.6) III - Pneumocystis carinii (fragment)

Observed	Mr(expt)	Mr(calc)	Delta	Start	End	Miss	Peptide
865.3301	864.3228	864.3800	-0.0572	69 -	75	0	K.TFTHICS.- + Carbamidomethyl (C)
993.4277	992.4205	992.4749	-0.0545	68 -	75	1	R.KTFTHICS.- + Carbamidomethyl (C)
1167.4829	1166.4756	1166.5311	-0.0555	23 -	32	0	R.AEAMELMNVK.N + 2 Oxidation (M)
3664.6062	3663.5989	3663.6279	-0.0290	1 -	32	1	-.PYNADFDGDEMNLHVPQTEEARAEAMELMNVK.N

No match to: 751.3859, 793.3455, 832.2988, 848.3093, 861.0016, 876.9628, 892.9507, 915.4276, 988.3750, 1005.3922, 1064.4451, 1216.4928, 1264.5354, 1501.7024, 1545.5785, 1549.6700, 1565.6430, 1622.7394, 1729.6508, 1730.5932, 1744.7499, 1747.6291, 1761.7506, 1832.7851, 1848.8553, 1866.8770, 1889.8854, 2019.7869, 2046.8381, 2062.8302, 2063.8573, 2079.8709, 2095.8419, 2117.7673, 2133.9402, 2149.9197, 2210.9927, 2264.8464, 2334.0437, 2450.0481, 2466.9528, 2519.1418, 2535.1542, 2551.9627, 2589.1406, 2611.1242, 2644.0843, 2717.0583, 2732.2341, 2789.3006, 3110.3047, 3163.4230, 3647.6063, 3937.5706, 3976.6005, 3993.6333

39. Q8NRN6_CORGL **Mass:** 10266 **Score:** 39 **Expect:** 3.7e+02 **Queries matched:** 4

Hypothetical protein Cgl1012 (Hypothetical protein).- Corynebacterium glutamicum (Brevibacterium flavum).

Observed	Mr(expt)	Mr(calc)	Delta	Start	End	Miss	Peptide
2095.8419	2094.8347	2094.9819	-0.1473	26 -	42	1	R.TDYFTTEEQKLYGMELK.T
2519.1418	2518.1345	2518.1838	-0.0493	2 -	23	0	M.SWAFNGFLSDDALVELMGLDFR.N + Oxidation (M)
2589.1406	2588.1334	2588.3056	-0.1723	36 -	58	1	K.LYGMELKTAIENPDSAAFINHVR.I
2789.3006	2788.2933	2788.3278	-0.0345	2 -	25	1	M.SWAFNGFLSDDALVELMGLDFRNR.T + Oxidation (M)

No match to: 751.3859, 793.3455, 832.2988, 848.3093, 861.0016, 865.3301, 876.9628, 892.9507, 915.4276, 988.3750, 993.4277, 1005.3922, 1064.4451, 1167.4829, 1216.4928, 1264.5354, 1501.7024, 1545.5785, 1549.6700, 1565.6430, 1622.7394, 1729.6508, 1730.5932, 1744.7499, 1747.6291, 1761.7506, 1832.7851, 1848.8553, 1866.8770, 1889.8854, 2019.7869, 2046.8381, 2062.8302, 2063.8573, 2079.8709, 2117.7673, 2133.9402, 2149.9197, 2210.9927, 2264.8464, 2334.0437, 2450.0481, 2466.9528, 2535.1542, 2551.9627, 2611.1242, 2644.0843, 2717.0583, 2732.2341, 3110.3047, 3163.4230, 3647.6063, 3664.6062, 3937.5706, 3976.6005, 3993.6333

40. Q3MHI8_BOVIN **Mass:** 39549 **Score:** 39 **Expect:** 3.7e+02 **Queries matched:** 8

Hypothetical protein MGC128771.- Bos taurus (Bovine).

Observed	Mr(expt)	Mr(calc)	Delta	Start	End	Miss	Peptide
865.3301	864.3228	864.3283	-0.0055	153 -	159	0	K.NMDENDK.G
993.4277	992.4205	992.4233	-0.0028	152 -	159	1	K.KNMDENDK.G
1761.7506	1760.7434	1760.7094	0.0340	277 -	292	0	K.GAVPTDQACESCSHDK.K + 2 Carbamidomethyl (C)
1832.7851	1831.7778	1831.7829	-0.0051	277 -	293	1	K.GAVPTDQACESCSHDKK.F + Carbamidomethyl (C)
1889.8854	1888.8781	1888.8043	0.0738	277 -	293	1	K.GAVPTDQACESCSHDKK.F + 2 Carbamidomethyl (C)
2019.7869	2018.7796	2018.8383	-0.0587	1 -	17	0	-.MSSEMLPAFSETCNVER.Q + Carbamidomethyl (C); 2 Oxidation (M)
2046.8381	2045.8308	2046.0282	-0.1975	221 -	239	0	K.QASDHGIDHAQLPLGNFVK.M
2535.1542	2534.1469	2534.2448	-0.0979	221 -	243	1	K.QASDHGIDHAQLPLGNFVKMNSR.K

No match to: 751.3859, 793.3455, 832.2988, 848.3093, 861.0016, 876.9628, 892.9507, 915.4276, 988.3750, 1005.3922, 1064.4451, 1167.4829, 1216.4928, 1264.5354, 1501.7024, 1545.5785, 1549.6700, 1565.6430, 1622.7394, 1729.6508, 1730.5932, 1744.7499, 1747.6291, 1848.8553, 1866.8770, 2062.8302, 2063.8573, 2079.8709, 2095.8419, 2117.7673, 2133.9402, 2149.9197, 2210.9927, 2264.8464, 2334.0437, 2450.0481, 2466.9528, 2519.1418, 2551.9627, 2589.1406, 2611.1242, 2644.0843, 2717.0583, 2732.2341, 2789.3006, 3110.3047, 3163.4230, 3647.6063, 3664.6062, 3937.5706, 3976.6005, 3993.6333

41. BAB22477 **Mass:** 30402 **Score:** 39 **Expect:** 3.9e+02 **Queries matched:** 7

AK002954 NID: - Mus musculus

Observed	Mr(expt)	Mr(calc)	Delta	Start	End	Miss	Peptide
793.3455	792.3382	792.3687	-0.0305	139 -	144	0	K.MEQLEK.D + Oxidation (M)
988.3750	987.3677	987.4556	-0.0879	98 -	105	1	K.HQDSSMRK.F
1264.5354	1263.5281	1263.5877	-0.0596	198 -	207	1	K.MEQELDARTR.E + Oxidation (M)
1549.6700	1548.6627	1548.7896	-0.1268	70 -	82	1	K.LEARLSDYAEQVR.N
2062.8302	2061.8229	2061.9814	-0.1585	212 -	229	0	R.TQEELVTSNQLSSDLNER.L
2063.8573	2062.8500	2062.9807	-0.1306	167 -	183	1	K.RDEIDELEGFQQQEISK.V
2551.9627	2550.9554	2551.0616	-0.1062	28 -	53	1	R.SVSKESVASMGADSGDDFASDGSSSR.E + Oxidation (M)

No match to: 751.3859, 832.2988, 848.3093, 861.0016, 865.3301, 876.9628, 892.9507, 915.4276, 993.4277, 1005.3922, 1064.4451, 1167.4829, 1216.4928, 1501.7024, 1545.5785, 1565.6430, 1622.7394, 1729.6508, 1730.5932, 1744.7499, 1747.6291, 1761.7506, 1832.7851, 1848.8553, 1866.8770, 1889.8854, 2019.7869, 2046.8381, 2079.8709, 2095.8419, 2117.7673, 2133.9402, 2149.9197, 2210.9927, 2264.8464, 2334.0437, 2450.0481, 2466.9528, 2519.1418, 2535.1542, 2589.1406, 2611.1242, 2644.0843, 2717.0583, 2732.2341, 2789.3006, 3110.3047, 3163.4230, 3647.6063, 3664.6062, 3937.5706, 3976.6005, 3993.6333

42. Q3ISL2_NATPD **Mass:** 6952 **Score:** 39 **Expect:** 4e+02 **Queries matched:** 4

Hypothetical protein.- Natronomonas pharaonis (strain DSM 2160 / ATCC 35678).

Observed	Mr(expt)	Mr(calc)	Delta	Start	End	Miss	Peptide
915.4276	914.4203	914.4570	-0.0366	54 -	61	1	R.RESAPEAR.H

1729.6508	1728.6436	1728.6905	-0.0470	1 -	16	0	-.MSQGDADATFVCPECR.S
2611.1242	2610.1169	2610.2417	-0.1247	27 -	53	0	R.TALLDEGCVVCGAAVSADAFGTVATGR.R + Carbamidomethyl (C)
3647.6063	3646.5990	3646.7792	-0.1802	17 -	53	1	R.SEVPVTPPMRTALLDEGCVVCGAAVSADAFGTVATGR.R

No match to: 751.3859, 793.3455, 832.2988, 848.3093, 861.0016, 865.3301, 876.9628, 892.9507, 988.3750, 993.4277, 1005.3922, 1064.4451, 1167.4829, 1216.4928, 1264.5354, 1501.7024, 1545.5785, 1549.6700, 1565.6430, 1622.7394, 1730.5932, 1744.7499, 1747.6291, 1761.7506, 1832.7851, 1848.8553, 1866.8770, 1889.8854, 2019.7869, 2046.8381, 2062.8302, 2063.8573, 2079.8709, 2095.8419, 2117.7673, 2133.9402, 2149.9197, 2210.9927, 2264.8464, 2334.0437, 2450.0481, 2466.9528, 2519.1418, 2535.1542, 2551.9627, 2589.1406, 2644.0843, 2717.0583, 2732.2341, 2789.3006, 3110.3047, 3163.4230, 3664.6062, 3937.5706, 3976.6005, 3993.6333

43. BAD13409 **Mass:** 14179 **Score:** 39 **Expect:** 4.2e+02 **Queries matched:** 6

AB121202 NID: - Macrothele gigas

Observed	Mr(expt)	Mr(calc)	Delta	Start	End	Miss	Peptide
993.4277	992.4205	992.4563	-0.0358	35 -	42	1	K.DEFKDPSR.S
1064.4451	1063.4378	1063.5298	-0.0919	27 -	34	1	K.NEFEERLK.D
1848.8553	1847.8480	1847.8552	-0.0072	99 -	112	1	R.KLECVETSGYWWYK.R + Carbamidomethyl (C)
2062.8302	2061.8229	2061.7722	0.0507	81 -	96	0	R.CMGYDIECNENLPCCK.H + 4 Carbamidomethyl (C)
2063.8573	2062.8500	2062.8039	0.0462	80 -	96	1	K.RCMGYDIECNENLPCCK.H + Carbamidomethyl (C); Oxidation (M)
2732.2341	2731.2268	2731.4903	-0.2635	1 -	26	0	-.MMTLSPFLLLLIAAVVIGNASEGEVK.N + Oxidation (M)

No match to: 751.3859, 793.3455, 832.2988, 848.3093, 861.0016, 865.3301, 876.9628, 892.9507, 915.4276, 988.3750, 1005.3922, 1167.4829, 1216.4928, 1264.5354, 1501.7024, 1545.5785, 1549.6700, 1565.6430, 1622.7394, 1729.6508, 1730.5932, 1744.7499, 1747.6291, 1761.7506, 1832.7851, 1866.8770, 1889.8854, 2019.7869, 2046.8381, 2079.8709, 2095.8419, 2117.7673, 2133.9402, 2149.9197, 2210.9927, 2264.8464, 2334.0437, 2450.0481, 2466.9528, 2519.1418, 2535.1542, 2551.9627, 2589.1406, 2611.1242, 2644.0843, 2717.0583, 2789.3006, 3110.3047, 3163.4230, 3647.6063, 3664.6062, 3937.5706, 3976.6005, 3993.6333

44. Q963V7_9DIPT **Mass:** 17429 **Score:** 39 **Expect:** 4.4e+02 **Queries matched:** 5

Reverse transcriptase (Fragment).- Chironomus agilis.

Observed	Mr(expt)	Mr(calc)	Delta	Start	End	Miss	Peptide
1501.7024	1500.6952	1500.8275	-0.1323	74 -	85	1	R.VWHNGLLYKMLK.L
1744.7499	1743.7427	1743.8501	-0.1075	59 -	73	1	K.STGMIFLDVEKAFDR.V + Oxidation (M)
1848.8553	1847.8480	1848.0145	-0.1665	88 -	103	1	R.FPQPLIKTVSSFLSER.S
2535.1542	2534.1469	2534.3532	-0.2063	121 -	143	0	K.FGVPQGAVLSPTLYNIFTQDIVR.E
2611.1242	2610.1169	2610.2357	-0.1187	16 -	37	1	R.INEHIDDNNIIPNQQCGFRTGR.S + Carbamidomethyl (C)

No match to: 751.3859, 793.3455, 832.2988, 848.3093, 861.0016, 865.3301, 876.9628, 892.9507, 915.4276, 988.3750, 993.4277, 1005.3922, 1064.4451, 1167.4829, 1216.4928, 1264.5354, 1545.5785, 1549.6700, 1565.6430, 1622.7394, 1729.6508, 1730.5932, 1747.6291, 1761.7506, 1832.7851, 1866.8770, 1889.8854, 2019.7869, 2046.8381, 2062.8302, 2063.8573, 2079.8709, 2095.8419, 2117.7673, 2133.9402, 2149.9197, 2210.9927, 2264.8464, 2334.0437, 2450.0481, 2466.9528, 2519.1418, 2551.9627, 2589.1406, 2644.0843, 2717.0583, 2732.2341, 2789.3006, 3110.3047, 3163.4230, 3647.6063, 3664.6062, 3937.5706, 3976.6005, 3993.6333

45. Q4XGI8_PLACH **Mass:** 3067 **Score:** 39 **Expect:** 4.4e+02 **Queries matched:** 3

Hypothetical protein (Fragment).- Plasmodium chabaudi.

Observed	Mr(expt)	Mr(calc)	Delta	Start	End	Miss	Peptide
1549.6700	1548.6627	1548.7606	-0.0978	10 -	23	0	K.NNNEVIFITCASPK.N
1848.8553	1847.8480	1847.9199	-0.0719	10 -	25	1	K.NNNEVIFITCASPKNK.K + Carbamidomethyl (C)
2079.8709	2078.8636	2078.9877	-0.1241	6 -	23	1	K.ACNKNNNEVIFITCASPK.N + 2 Carbamidomethyl (C)

No match to: 751.3859, 793.3455, 832.2988, 848.3093, 861.0016, 865.3301, 876.9628, 892.9507, 915.4276, 988.3750, 993.4277, 1005.3922, 1064.4451, 1167.4829, 1216.4928, 1264.5354, 1501.7024, 1545.5785, 1565.6430, 1622.7394, 1729.6508, 1730.5932, 1744.7499, 1747.6291, 1761.7506, 1832.7851, 1866.8770, 1889.8854, 2019.7869, 2046.8381, 2062.8302, 2063.8573, 2095.8419, 2117.7673, 2133.9402, 2149.9197, 2210.9927, 2264.8464, 2334.0437, 2450.0481, 2466.9528, 2519.1418, 2535.1542, 2551.9627, 2589.1406, 2611.1242, 2644.0843, 2717.0583, 2732.2341, 2789.3006, 3110.3047, 3163.4230, 3647.6063, 3664.6062, 3937.5706, 3976.6005, 3993.6333

46. Q9JI50_MOUSE **Mass:** 27603 **Score:** 39 **Expect:** 4.4e+02 **Queries matched:** 7

Dendritic cell-associated C-type lectin-1.- Mus musculus (Mouse).

Observed	Mr(expt)	Mr(calc)	Delta	Start	End	Miss	Peptide
751.3859	750.3787	750.3660	0.0126	82 -	87	0	K.DNFLSR.N
993.4277	992.4205	992.5039	-0.0834	82 -	89	1	K.DNFLSRNK.E
1167.4829	1166.4756	1166.5428	-0.0672	72 -	81	1	R.HNSGRNPEEK.D
1832.7851	1831.7778	1831.7875	-0.0097	129 -	144	0	K.SCYLFSFSGNSWYGSK.R
1889.8854	1888.8781	1888.8090	0.0691	129 -	144	0	K.SCYLFSFSGNSWYGSK.R + Carbamidomethyl (C)
2732.2341	2731.2268	2731.2091	0.0177	185 -	207	0	R.NQSEGPWFWEDGSAFFPNSFQVR.N
2789.3006	2788.2933	2788.3424	-0.0491	103 -	128	1	K.VAPSKASQTTGGFSQSCLPNWIMHGK.S + Carbamidomethyl (C)

No match to: 793.3455, 832.2988, 848.3093, 861.0016, 865.3301, 876.9628, 892.9507, 915.4276, 988.3750, 1005.3922, 1064.4451, 1216.4928, 1264.5354, 1501.7024, 1545.5785, 1549.6700, 1565.6430, 1622.7394, 1729.6508, 1730.5932, 1744.7499, 1747.6291, 1761.7506, 1848.8553, 1866.8770, 2019.7869, 2046.8381, 2062.8302, 2063.8573, 2079.8709, 2095.8419, 2117.7673, 2133.9402, 2149.9197, 2210.9927, 2264.8464, 2334.0437, 2450.0481, 2466.9528, 2519.1418, 2535.1542, 2551.9627, 2589.1406, 2611.1242, 2644.0843, 2717.0583, 3110.3047, 3163.4230, 3647.6063, 3664.6062, 3937.5706, 3976.6005, 3993.6333

47. Q8GXA4_ARATH **Mass:** 53868 **Score:** 39 **Expect:** 4.4e+02 **Queries matched:** 8

Hypothetical protein At4g26450/M3E9_120 (At4g26450).- Arabidopsis thaliana (Mouse-ear cress).

Observed	Mr(expt)	Mr(calc)	Delta	Start	End	Miss	Peptide
793.3455	792.3382	792.3878	-0.0496	429 -	435	0	K.AANFTNR.A
1501.7024	1500.6952	1500.6692	0.0260	223 -	235	1	K.ENSYSSLESDSRK.Q
1744.7499	1743.7427	1743.8387	-0.0961	349 -	363	1	K.ESHIRDLESTTNQSK.H
1761.7506	1760.7434	1760.8614	-0.1181	436 -	451	0	R.AQDLQNDCIEITGTIK.K
1889.8854	1888.8781	1888.9564	-0.0783	436 -	452	1	R.AQDLQNDCIEITGTIKK.R
2062.8302	2061.8229	2061.8302	-0.0073	236 -	253	1	K.QSGRMMDYNGENGETSMR.K

```
2264.8464  2263.8392  2263.9716  -0.1324   215 - 234   1  R.GESVDFEKENSYSSLESDSR.K
3110.3047  3109.2975  3109.5277  -0.2302    45 -  75   0  K.LLSTSNSVELGKPMSFDSPGDGGGAYSPVLK.G
```

No match to: 751.3859, 832.2988, 848.3093, 861.0016, 865.3301, 876.9628, 892.9507, 915.4276, 988.3750, 993.4277, 1005.3922, 1064.4451, 1167.4829, 1216.4928, 1264.5354, 1545.5785, 1549.6700, 1565.6430, 1622.7394, 1729.6508, 1730.5932, 1747.6291, 1832.7851, 1848.8553, 1866.8770, 2019.7869, 2046.8381, 2063.8573, 2079.8709, 2095.8419, 2117.7673, 2133.9402, 2149.9197, 2210.9927, 2334.0437, 2450.0481, 2466.9528, 2519.1418, 2535.1542, 2551.9627, 2589.1406, 2611.1242, 2644.0843, 2717.0583, 2732.2341, 2789.3006, 3163.4230, 3647.6063, 3664.6062, 3937.5706, 3976.6005, 3993.6333

48. Q9D0J3_MOUSE **Mass:** 12279 **Score:** 39 **Expect:** 4.5e+02 **Queries matched:** 5

10 days embryo whole body cDNA, RIKEN full-length enriched library, clone:2610010G17 product:GRAF homolog.- Mus musculus (Mouse).

Observed	Mr(expt)	Mr(calc)	Delta	Start	End	Miss	Peptide
1889.8854	1888.8781	1888.9642	-0.0861	86 -	101	1	R.SLQEFAAVLRNLEDER.S
2046.8381	2045.8308	2045.8129	0.0179	69 -	85	0	K.FQCIGDAETDDEMCIAR.S + 2 Carbamidomethyl (C); Oxidation (M)
2063.8573	2062.8500	2062.9240	-0.0740	2 -	19	0	M.GLPALEFSDCCLDSPHFR.E + Carbamidomethyl (C)
2210.9927	2209.9855	2209.9594	0.0260	1 -	19	0	-.MGLPALEFSDCCLDSPHFR.E + Carbamidomethyl (C); Oxidation (M)
2535.1542	2534.1469	2534.1933	-0.0464	2 -	23	1	M.GLPALEFSDCCLDSPHFRETLK.S + Carbamidomethyl (C)

No match to: 751.3859, 793.3455, 832.2988, 848.3093, 861.0016, 865.3301, 876.9628, 892.9507, 915.4276, 988.3750, 993.4277, 1005.3922, 1064.4451, 1167.4829, 1216.4928, 1264.5354, 1501.7024, 1545.5785, 1549.6700, 1565.6430, 1622.7394, 1729.6508, 1730.5932, 1744.7499, 1747.6291, 1761.7506, 1832.7851, 1848.8553, 1866.8770, 2019.7869, 2062.8302, 2079.8709, 2095.8419, 2117.7673, 2133.9402, 2149.9197, 2264.8464, 2334.0437, 2450.0481, 2466.9528, 2519.1418, 2551.9627, 2589.1406, 2611.1242, 2644.0843, 2717.0583, 2732.2341, 2789.3006, 3110.3047, 3163.4230, 3647.6063, 3664.6062, 3937.5706, 3976.6005, 3993.6333

49. Q4DDU3_TRYCR **Mass:** 29352 **Score:** 38 **Expect:** 4.6e+02 **Queries matched:** 6

Mucin-associated surface protein (MASP), putative.- Trypanosoma cruzi.

Observed	Mr(expt)	Mr(calc)	Delta	Start	End	Miss	Peptide
1264.5354	1263.5281	1263.5942	-0.0661	206 -	217	1	K.QDGKDTTSVGEK.N
2535.1542	2534.1469	2534.2113	-0.0644	9 -	31	1	R.VLLVCALCVLLCGAGGRCDEEER.A + 2 Carbamidomethyl (C)
2551.9627	2550.9554	2551.1244	-0.1690	26 -	51	1	R.CDEEERAALGSDGGPPPGSAEPGTPR.K
2611.1242	2610.1169	2610.2758	-0.1589	2 -	25	1	M.AMMMAGRVLLVCALCVLLCGAGGR.C + 3 Carbamidomethyl (C); 2 Oxidation (M)
2644.0843	2643.0770	2643.1631	-0.0860	233 -	258	1	R.DEGSEGTGEDTKATTVTANTTDTTNK.Q
2717.0583	2716.0510	2716.2846	-0.2336	1 -	25	1	-.MAMMMAGRVLLVCALCVLLCGAGGR.C + 2 Carbamidomethyl (C); 4 Oxidation (M)

No match to: 751.3859, 793.3455, 832.2988, 848.3093, 861.0016, 865.3301, 876.9628, 892.9507, 915.4276, 988.3750, 993.4277, 1005.3922, 1064.4451, 1167.4829, 1216.4928, 1501.7024, 1545.5785, 1549.6700, 1565.6430, 1622.7394, 1729.6508, 1730.5932, 1744.7499, 1747.6291, 1761.7506, 1832.7851, 1848.8553, 1866.8770, 1889.8854, 2019.7869, 2046.8381, 2062.8302, 2063.8573, 2079.8709, 2095.8419, 2117.7673, 2133.9402, 2149.9197, 2210.9927, 2264.8464, 2334.0437, 2450.0481, 2466.9528, 2519.1418, 2589.1406, 2732.2341, 2789.3006, 3110.3047, 3163.4230, 3647.6063, 3664.6062, 3937.5706, 3976.6005, 3993.6333

50. T43206 **Mass:** 43182 **Score:** 38 **Expect:** 4.6e+02 **Queries matched:** 9

probable aminopeptidase (EC 3.4.11.-) - fission yeast (Schizosaccharomyces pombe)

Observed	Mr(expt)	Mr(calc)	Delta	Start	End	Miss	Peptide
1622.7394	1621.7321	1621.7981	-0.0660	180 -	195	0	R.LDNLGMTSGASQALTK.S + Oxidation (M)
1747.6291	1746.6218	1746.7851	-0.1633	345 -	359	0	R.TLDLGNPMLSMHSCR.E + Carbamidomethyl (C); Oxidation (M)
2046.8381	2045.8308	2046.0204	-0.1896	1 -	19	0	-.MIGNGFSIIATHTDSPTLR.L + Oxidation (M)
2063.8573	2062.8500	2062.9598	-0.1097	325 -	344	1	R.NDSPCGSTIGPKLAAMTGMR.T + Carbamidomethyl (C)
2079.8709	2078.8636	2078.9547	-0.0911	325 -	344	1	R.NDSPCGSTIGPKLAAMTGMR.T + Carbamidomethyl (C); Oxidation (M)
2095.8419	2094.8347	2094.9496	-0.1149	325 -	344	1	R.NDSPCGSTIGPKLAAMTGMR.T + Carbamidomethyl (C); 2 Oxidation (M)
2611.1242	2610.1169	2610.1844	-0.0675	337 -	359	1	K.LAAMTGMRTLDLGNPMLSMHSCR.E + Carbamidomethyl (C); 3 Oxidation (M)
2644.0843	2643.0770	2643.2532	-0.1762	366 -	387	0	K.DFEYAVVLFSSFFQNFANLEEK.I
2789.3006	2788.2933	2788.3397	-0.0463	27 -	50	1	K.SAYGYLQVGVEKYGGGIWHTWFDR.D

No match to: 751.3859, 793.3455, 832.2988, 848.3093, 861.0016, 865.3301, 876.9628, 892.9507, 915.4276, 988.3750, 993.4277, 1005.3922, 1064.4451, 1167.4829, 1216.4928, 1264.5354, 1501.7024, 1545.5785, 1549.6700, 1565.6430, 1729.6508, 1730.5932, 1744.7499, 1761.7506, 1832.7851, 1848.8553, 1866.8770, 1889.8854, 2019.7869, 2062.8302, 2117.7673, 2133.9402, 2149.9197, 2210.9927, 2264.8464, 2334.0437, 2450.0481, 2466.9528, 2519.1418, 2535.1542, 2551.9627, 2589.1406, 2717.0583, 2732.2341, 3110.3047, 3163.4230, 3647.6063, 3664.6062, 3937.5706, 3976.6005, 3993.6333

Search Parameters

Type of search : Peptide Mass Fingerprint
Enzyme : Trypsin
Variable modifications : Carbamidomethyl (C),Oxidation (M)
Mass values : Monoisotopic
Protein Mass : Unrestricted
Peptide Mass Tolerance : ± 100 ppm
Peptide Charge State : 1+
Max Missed Cleavages : 1
Number of queries : 60

Mascot: http://www.matrixscience.com/
